# Interferometer techniques for gravitational-wave detection

**DOI:** 10.1007/s41114-016-0002-8

**Published:** 2017-02-17

**Authors:** Charlotte Bond, Daniel Brown, Andreas Freise, Kenneth A. Strain

**Affiliations:** 1 School of Physics and Astronomy, University of Birmingham, Birmingham, B15 2TT UK; 2 School of Physics and Astronomy, University of Glasgow, Glasgow, G12 8QQ UK

**Keywords:** Gravitational waves, Gravitational-wave detectors, Laser interferometry, Optics, Simulations, Finesse

## Abstract

Several km-scale gravitational-wave detectors have been constructed worldwide. These instruments combine a number of advanced technologies to push the limits of precision length measurement. The core devices are laser interferometers of a new kind; developed from the classical Michelson topology these interferometers integrate additional optical elements, which significantly change the properties of the optical system. Much of the design and analysis of these laser interferometers can be performed using well-known classical optical techniques; however, the complex optical layouts provide a new challenge. In this review, we give a textbook-style introduction to the optical science required for the understanding of modern gravitational wave detectors, as well as other high-precision laser interferometers. In addition, we provide a number of examples for a freely available interferometer simulation software and encourage the reader to use these examples to gain hands-on experience with the discussed optical methods.

## Introduction

### The scope and style of the review

The historical development of laser interferometers for application as gravitational-wave detectors (Pitkin et al. 2011) has involved the combination of relatively simple optical subsystems into more and more complex assemblies. The individual elements that compose the interferometers, including mirrors, beam splitters, lasers, modulators, various polarising optics, photo detectors and so forth, are individually well described by relatively simple, mostly-classical physics. Complexity arises from the combination of multiple mirrors, beam splitters etc. into optical cavity systems that have narrow resonant features, and the consequent requirement to stabilise relative separations of the various components to sub-wavelength accuracy, and indeed in many cases to very small fractions of a wavelength.

Thus, classical physics describes the interferometer techniques and the operation of current gravitational-wave detectors. However, we note that at signal frequencies above a couple of hundreds of Hertz, the sensitivity of current detectors is limited by the photon counting noise at the interferometer readout, also called shot-noise. The next generation systems such as Advanced LIGO (Fritschel 2003; Aasi 2015), Advanced Virgo (Acernese 2015) and KAGRA (Aso et al. 2013) are expected to operate in a regime where the quantum physics of both light and mirror motion couple to each other. Then, a rigorous quantum-mechanical description is certainly required. Sensitivity improvements beyond these ‘Advanced’ detectors necessitate the development of *non-classical* techniques; a comprehensive discussion of such techniques is provided in Danilishin and Khalili (2012). This review provides a brief introduction to quantum noise in Sect. 6 but otherwise focusses on the non-quantum aspects of interferometry that play an important role in overcoming other limits to current detectors, due to, for example, thermal effects and feedback control systems. At the same time these classical techniques will provide the means for implementing new, non-classical schemes and just remain as important as ever.

The optical components employed tend to behave in a linear fashion with respect to the optical field, i.e., nonlinear optical effects need hardly be considered. Indeed, almost all aspects of the design of laser interferometers are dealt with in the linear regime. Therefore the underlying mathematics is relatively simple and many standard techniques are available, including those that naturally allow numerical solution by computer models. Such computer models are in fact necessary as the exact solutions can become quite complicated even for systems of a few components. In practice, workers in the field rarely calculate the behaviour of the optical systems from first principles, but instead rely on various well-established numerical modelling techniques. An example of software that enables modelling of interferometers and their component systems is Finesse (Freise et al. 2004; Freise 2015). This was developed by some of us (AF, DB), has been validated in a wide range of situations, and was used to prepare the examples included in the present review.

The target readership we have in mind is the student or researcher who desires to get to grips with practical issues in the design of interferometers or component parts thereof. For that reason, this review consists of sections covering the basic physics and approaches to simulation, intermixed with some practical examples. To make this as useful as possible, the examples are intended to be realistic with sensible parameters reflecting typical application in gravitational wave detectors. The examples, prepared using Finesse, are designed to illustrate the methods typically applied in designing gravitational wave detectors. We encourage the reader to obtain Finesse and to follow the examples (see “Appendix A”).

### Overview of the goals of interferometer design for gravitational-wave detection

**Fig. 1:**
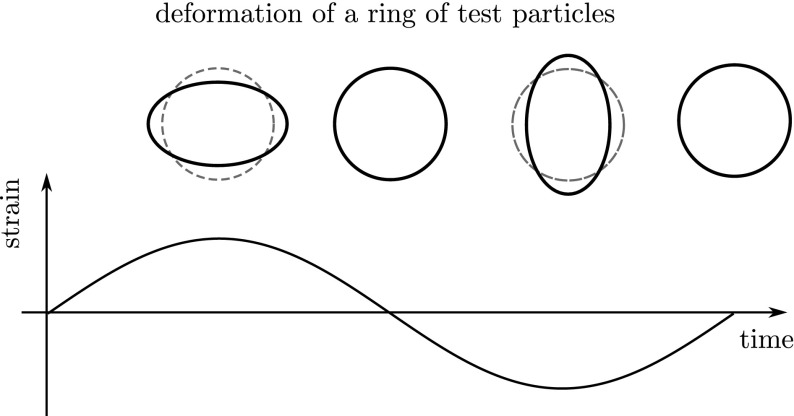
Gravitational waves are transverse quadrupole waves. If a wave passes through the ring of test particles that is oriented perpendicular to the direction of wave propagation, the distances between the particles would change periodically as shown in this *sketch*

**Fig. 2:**
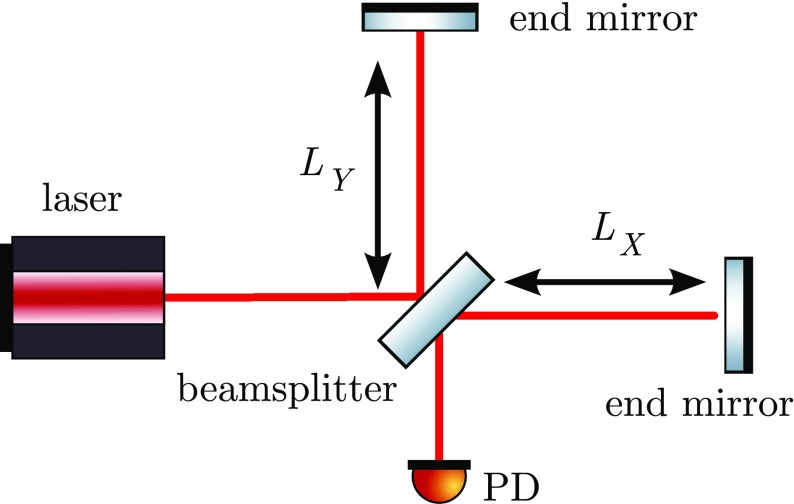
Simplified layout of a Michelson interferometer. The laser provides the input light, which is split into two beams by the central beamsplitters. The beams reflect off the end mirrors and recombine at the beamsplitter. The light power on the main photo detector (PD) changes when the difference between the arm length $$\varDelta L = L_X -
											L_Y$$ changes

Gravitational waves are transverse quadrupole waves travelling at the speed of light. They are distortions in space-time that can be detected by measuring the distance between test masses, see Fig. 1. A Michelson interferometer presents an ideal detector geometry, it is designed to measure relative length changes of two perpendicular directions in a plane, see Fig. 2. The end mirrors of the Michelson interferometer represent the test masses and any change in the relative distance between the central beam splitter and the end mirrors will produce a change in the light power detected in the output port.

The measurable length change induced by a gravitational-wave depends on the total length being measured. For gravitational waves with wavelength much larger than the detector size we get:

1.1 $$\begin{aligned}
								\varDelta L = h~L, \end{aligned}$$

with *L* the length of the detetor and *h* the strain amplitude of the gravitational wave. This scaling of the change with the base length led to the construction of interferometers with arm length of several kilometres.

Gravitational-wave detectors strive to pick out signals carried by passing gravitational waves from a background of self-generated noise. This is challenging because of the extremely small effects produces by the gravitational waves. For example, the first gravitational wave detected in September 2015 by the LIGO detectors (Abbott et al. 2016b), which is considered to be a strong event, reached a strain amplitude of $10^{-21}$. This signal could not have been measured with a simple Michelson interferometer. The performance of an interferometric detector is limited by its various internal noise sources, which includes quantum noise, the inherent quantum fluctuations of the laser beam used to generate the output signal. We show later [Eq. (6.21)] that the amplitude spectral sensitivity of a simple Michelson interferometer limited only by quantum noise^1^ would be given by:

1.2 $$\begin{aligned}
								\mathrm{NSR} = \sqrt{\frac{2\hbar }{P_0 \omega _0}} \frac{c}{L},
								\end{aligned}$$

with $$P_0$$ and $$\omega
								_0$$ the power and angular frequency of the laser light. The LIGO lasers have a wavelength of $$\lambda
								_0=1064$$ nm. If the LIGO instruments would be simple Michelson interferometers, to reach a sensitivity better than $$10^{-22}$$ would require a laser power of

1.3 $$\begin{aligned}
								P_0 > \frac{2\hbar }{10^{-44} \omega _0} \frac{c^2}{L^2}
								\approx 70\,\mathrm {kW}. \end{aligned}$$

However the LIGO laser system can deliver only several hundred watts of power. More powerful lasers exist but not with the required stability in amplitude and phase. Transmitting this many kilowatts of power through the injection optics and the central beam splitter would also cause undesirable and significant thermal deformations of the optics due to absorption. Instead we can use alternative interferometer configurations to increase the signal-to-noise ratio regarding quantum noise. In other words, we improve on the known concept of the Michelson interferometer and in the process invent new interferometer configurations, sometimes referred to as advanced interferometers.

Quantum noise is just one example of the challenges that need to be overcome to reach the desired sensitivity. Many new technologies and concepts have been—and are still being—invented, tested and refined to further develop these laser-interferometric gravitational wave detectors. It was this endeavour that finally resulted in the spectacular first detections of gravitational waves in 2015 (Abbott et al. 2016a, b). In this review we focus on those ideas that affect the optical layout and that use new interferometer configurations.

The evolution of gravitational-wave detectors can be seen by following their development from prototypes and early observing systems towards the so-called ‘Advanced detectors’, which are currently under construction, or in the case of Advanced LIGO, in the first phase of scientific observing (as of late 2015). Starting from the simplest Michelson interferometer (Forward 1978), then by the application of techniques to increase the number of photons stored in the arms: delay lines (Herriott et al. 1964), Fabry–Perot arm cavities (Fabry and Perot 1899; Fattaccioli et al. 1986) and power recycling (Billing et al. 1983; Drever et al. 1983). The final step in the development of classical interferometry was the inclusion of signal recycling (Meers 1988; Heinzel et al. 1998), which, among other effects, allows the signal from a gravitational-wave signal of approximately-known spectrum to be enhanced above the noise.

Reading out a signal from even the most basic interferometer requires minimising the coupling of local environmental effects to the detected output. Thus, the relative positions of all the components must be stabilised. This is commonly achieved by suspending the mirrors etc. as pendulums, often multi-stage pendulums in series, and then applying closed-loop control to maintain the desired operating condition. The careful engineering required to provide low-noise suspensions with the correct vibration isolation and low-noise actuation is described in many works, for example, Braccini et al. (1996), Plissi et al. (2000), Barriga et al. (2009) and Aston et al. (2012).

As the interferometer optics become more complicated the resonance conditions become more narrowly defined, i.e., the allowed combinations of inter-component path lengths required to allow the photon number in the interferometer arms to reach a maximum. It is likewise necessary to maintain angular alignment of all components so that beams required to interfere are correctly co-aligned. Typically the beams need to be aligned within a small fraction, and sometimes a very small fraction, of the far-field diffraction angle: the requirement can be in the low nano-radian range for km-scale detectors (Morrison et al. 1994; Freise et al. 2007). Therefore, for each optical component there is typically one longitudinal, i.e., along the direction of light propagation, plus two angular degrees of freedom: pitch and yaw about the longitudinal axis. A complex interferometer consists of up to around seven highly sensitive components and so there can be of order 20 degrees of freedom to be measured and controlled (Acernese 2006; Winkler et al. 2007).

Although the light fields are linear in their behaviour the coupling between the position of a mirror and the complex amplitude of the detected light field typically shows strongly nonlinear dependence on mirror positions due to the sharp resonance features exhibited by cavity systems. The fields do vary linearly, or at least they vary smoothly close to the desired operating point. So, while well-understood linear control theory suffices to design the control system needed to maintain the optical configuration at its operating point, the act of bringing the system to that operating condition is often a separate and more challenging nonlinear problem. In the current version of this work we consider only the linear aspects of sensing and control.

Control systems require actuators, and those employed are typically electrical-force transducers that act on the suspended optical components, either directly or—to provide enhanced noise rejection—at upper stages of multi-stage suspensions. The transducers are normally coil-magnet actuators, with the magnets on the moving part, or, less frequently, electrostatic actuators of varying design. The actuators are frequently regarded as part of the mirror suspension subsystem and are not discussed in the current work.

To give order to our review we consider the main physics describing the operation of the basic optical components: mirrors, beam splitters, modulators, etc., required to construct interferometers. Although all of the relevant physics is generally well known and not new, we take it as a starting point that permits the introduction of notation and conventions. It is also true that the interferometry employed for gravitational-wave detection has a different emphasis than other interferometer applications. As a consequence, descriptions or examples of a number of crucial optical properties for gravitational wave detectors cannot be found in the literature.

The purpose of this review is especially to provide a coherent theoretical framework for describing such effects. With the basics established, it can be seen that the interferometer configurations that have been employed in gravitational-wave detection may be built up and simulated in a relatively straightforward manner.

### Plane-wave analysis

The main optical systems of interferometric gravitational-wave detectors are designed such that all system parameters are well known and stable over time. The stability is achieved through a mixture of passive isolation systems and active feedback control. In particular, the light sources are some of the most stable, low-noise continuous-wave laser systems so that electromagnetic fields can be assumed to be essentially monochromatic. Additional frequency components can be modelled as small modulations in amplitude or phase. The laser beams are well collimated, propagate along a well-defined optical axis and remain always very much smaller than the optical elements they interact with. Therefore, these beams can be described as *paraxial* and the well-known paraxial approximations can be applied.

**Fig. 3:**
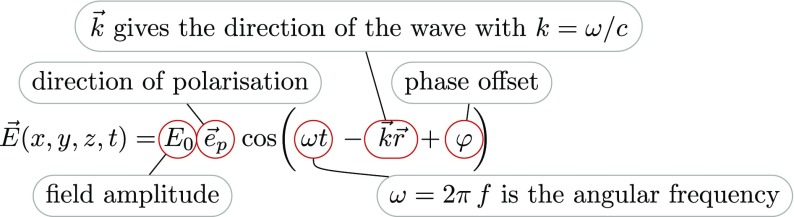
The electric field component of an electromagnetic wave

It is useful to first derive a mathematical model based on monochromatic, scalar, plane waves. As it turns out, a more detailed model including the polarisation and the shape of the laser beam as well as multiple frequency components, can be derived as an extension to the plane-wave model. A plane electromagnetic wave is typically described by its electric field component (Fig. 3):

with $$E_0$$ as the (constant) field amplitude in V/m, $$ \mathbf
								{e}_p$$ the unit vector in the direction of polarisation, such as, for example, $$\mathbf
								{e}_y$$ for $$\mathscr
								{S}$$-polarised light, $$\omega
								$$ the angular oscillation frequency of the wave, and $$\mathbf
								{k}=\mathbf {e}_k \omega /c$$ the wave vector pointing in the direction of propagation. The absolute phase $$\varphi
								$$ only becomes meaningful when the field is superposed with other light fields.

In this document we will consider waves propagating along the optical axis given by the *z*-axis, so that $$\mathbf
								{k}\mathbf {r}=kz$$. For the moment we will ignore the polarisation and use scalar waves, which can be written as

1.4 $$\begin{aligned}
								E(z,t)=E_0 \cos (\omega t - kz +\varphi ).
								\end{aligned}$$

Further, in this document we use complex notation, i.e.,

1.5 $$\begin{aligned}
								E={\mathfrak {R}}\left\{ E' \right\} \quad \text {with} \quad E'=
								E'_0 \exp \big (\mathrm {i}\,(\omega t - kz)\big ).
								\end{aligned}$$

This has the advantage that the scalar amplitude and the phase $$\varphi
								$$ can be given by one, now complex, amplitude $$E'_0=E_0 \exp
								(\mathrm {i}\,\varphi )$$. We will use this notation with complex numbers throughout. For clarity we will simply use the unprimed letters for the auxiliary field. In particular, we will use the letter *E* and also *a* and *b* to denote complex electric-field amplitudes. But remember that, for example, in $$E=E_0 \exp
								(-\mathrm {i}\,kz)$$ neither *E* nor $$E_0$$ are physical quantities. Only the real part of *E* exists and deserves the name field amplitude.

### Frequency domain analysis

In most cases we are either interested in the fields *at one particular location*, for example, on the surface of an optical element, or we want to know the fields at all places in the interferometer but *at one particular point in time*. The latter is usually true for the *steady state* approach: assuming that the interferometer is in a steady state, all solutions must be independent of time so that we can perform all computations at $t=0$ without loss of generality. In that case, the scalar plane wave can be written as

1.6 $$\begin{aligned}
								E=E_0 \exp (-\mathrm {i}\,kz).
								\end{aligned}$$

The frequency domain is of special interest as numerical models of gravitational-wave detectors tend to be much faster to compute in the frequency domain than in the time domain.

## Optical components: coupling of field amplitudes

**Fig. 4:**
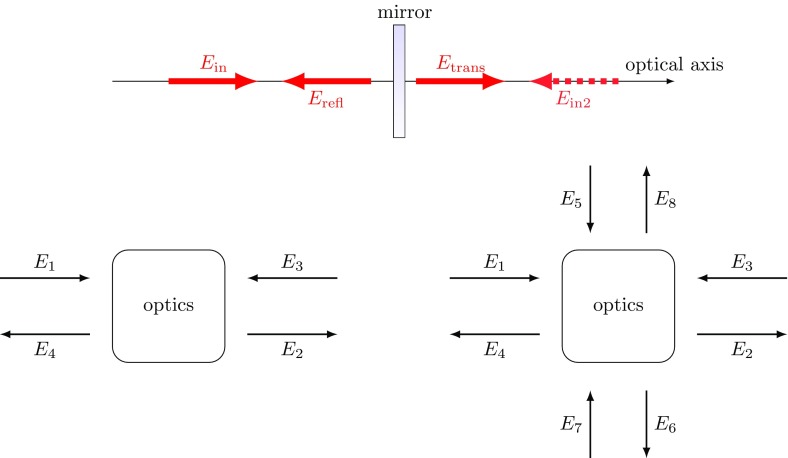
This set of figures introduces an abstract form of illustration, which will be used in this document. The *top figure* shows a typical example taken from the analysis of an optical system: an incident field $$E_{\mathrm
										{in}}$$ is reflected and transmitted by a semi-transparent mirror; there might be the possibility of second incident field $$E_{in2}$$. The *lower left figure* shows the abstract form we choose to represent the same system. The *lower right figure* depicts how this can be extended to include a beam splitter object, which connects two optical axes

When an electromagnetic wave interacts with an optical system, all of its parameters can be changed as a result. Typically optical components are designed such that, ideally, they only affect one of the parameters, i.e., either the amplitude *or* the polarisation *or* the shape. Therefore, it is convenient to derive separate descriptions concerning each parameter. This section introduces the coupling of the complex field amplitude at optical components. Typically, the optical components are described in the simplest possible way, as illustrated by the use of abstract schematics such as those shown in Fig. 4.

### Mirrors and spaces: reflection, transmission and propagation

The core optical systems of current interferometric gravitational interferometers are composed of two building blocks: a) resonant optical cavities, such as Fabry–Perot resonators, and b) beam splitters, as in a Michelson interferometer. In other words, the laser beam is either propagated through a vacuum system or interacts with a partially-reflecting optical surface.

The term *optical surface* generally refers to a boundary between two media with possibly different indices of refraction *n*, for example, the boundary between air and glass or between two types of glass. A real fused silica mirror in an interferometer features two surfaces, which interact with a reflected or transmitted laser beam. However, in some cases, one of these surfaces has been treated with an anti-reflection (AR) coating to minimise the effect on the transmitted beam.

The terms *mirror* and *beam splitter* are sometimes used to describe a (theoretical) optical surface in a model. We define real *amplitude coefficients* for reflection and transmission *r* and *t*, with $$0\le r,t \le
								1$$, so that the field amplitudes can be written as (Fig. 5)

**Fig. 5:**
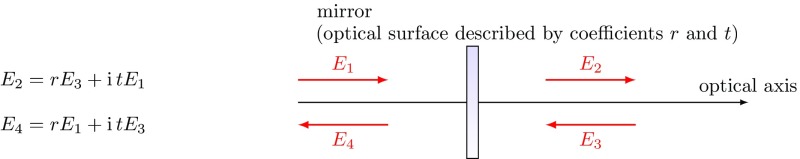
The coupling of field amplitudes at a mirror component

**Fig. 6:**
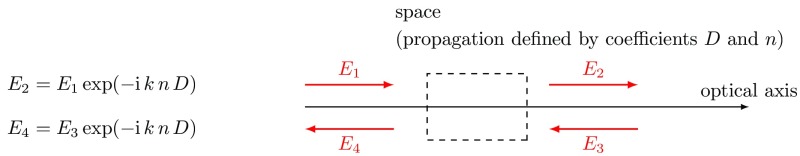
Coupling of field amplitudes for free propagation

The $$\pi
								/2$$ phase shift upon transmission (here given by the factor $$\mathrm
								{i}\,$$) refers to a phase convention explained in Sect. 2.4.

The free propagation of a distance *D* through a medium with index of refraction *n* can be described with the following set of equations (Fig. 6):

In the following we use $n=1$ for simplicity.

Note that we use above relations to demonstrate various mathematical methods for the analysis of optical systems. However, refined versions of the coupling equations for optical components, including those for spaces and mirrors, are also required, see, for example, Sect. 2.6.

### The two-mirror resonator

**Fig. 7:**
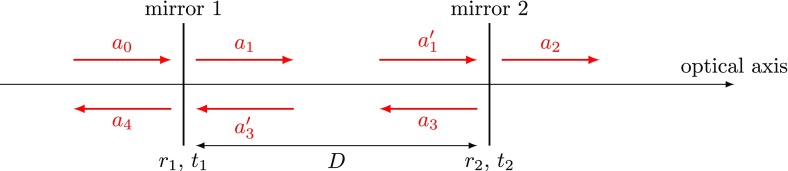
Simplified schematic of a two mirror cavity. The two mirrors are defined by the amplitude coefficients for reflection and transmission. Further, the resulting cavity is characterised by its length *D*. Light field amplitudes are shown and identified by a variable name, where necessary to permit their mutual coupling to be computed

The linear optical resonator, also called a *cavity* is formed by two partially-transparent mirrors, arranged in parallel as shown in Fig. 7. This simple setup makes a very good example with which to illustrate how a mathematical model of an interferometer can be derived, using the equations introduced in Sect. 2.1. A more detailed description of the two-mirror cavity is provided in Sect. 5.1.

The cavity is defined by a propagation length *D* (in vacuum), the amplitude reflectivities $$r_1,
								r_2$$ and the amplitude transmittances $$t_1,
								t_2$$. The amplitude at each point in the cavity can be computed simply as the superposition of fields. The entire set of equations can be written as

2.1 $$\begin{aligned}
								a_1= & {} \mathrm {i}\,t_1 a_0 + r_1 a_3'\nonumber \\ a_1'=
								& {} \exp (-\mathrm {i}\,k D)~ a_1\nonumber \\ a_2=
								& {} \mathrm {i}\,t_2 a_1'\nonumber \\ a_3= & {} r_2
								a_1'\nonumber \\ a_3'= & {} \exp (-\mathrm {i}\,k D)
								~a_3\nonumber \\ a_4= & {} r_1 a_0 + \mathrm {i}\,t_1 a_3'
								\end{aligned}$$

The circulating field impinging on the first mirror (surface) $a_3'$ can now be computed as

2.2 $$\begin{aligned}
								a_3'= & {} \exp (-\mathrm {i}\,k D) ~a_3=\exp (-\mathrm
								{i}\,k D)~ r_2 a_1' =\exp (-\mathrm {i}\,2 k D)~ r_2 a_1 \nonumber
								\\= & {} \exp (-\mathrm {i}\,2 k D)~ r_2~ (\mathrm {i}\,t_1
								a_0 + r_1 a_3'). \end{aligned}$$

This then yields

2.3 $$\begin{aligned}
								a_3'=a_0\frac{\mathrm {i}\,r_2 t_1 \exp (-\mathrm {i}\,2 k
								D)}{1-r_1r_2\exp (-\mathrm {i}\,2 k D)}.
								\end{aligned}$$

We can directly compute the reflected field to be

2.4 $$\begin{aligned}
								a_4=a_0\left( r_1 - \frac{r_2 t_1^2 \exp (-\mathrm {i}\,2 k
								D)}{1-r_1 r_2 \exp (- \mathrm {i}\,2 k D)}\right) = a_0\left(
								\frac{r_1-r_2(r_1^2+t_1^2)\exp (-\mathrm {i}\,2 k D)}{1-r_1 r_2 \exp
								(- \mathrm {i}\,2 k D)}\right) ,
								\end{aligned}$$

while the transmitted field becomes

2.5 $$\begin{aligned}
								a_2=a_0 \frac{-t_1 t_2 \exp (-\mathrm {i}\,k D)}{1-r_1 r_2 \exp (-
								\mathrm {i}\,2 k D)}. \end{aligned}$$

The properties of two mirror cavities will be discussed in more detail in Sect. 5.1.

### Coupling matrices

**Fig. 8:**
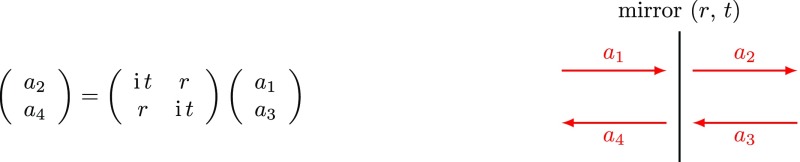
Coupling matrix for field amplitudes at a mirror

**Fig. 9:**
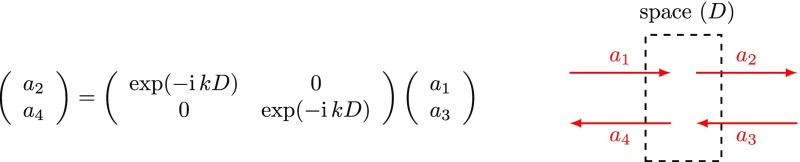
Coupling matrix for field amplitudes at a space

Computations that involve sets of linear equations as shown in Sect. 2.2 can often be done or written efficiently with matrices. Two methods of applying matrices to coupling field amplitudes are demonstrated below, using again the example of a two mirror cavity. First of all, we can rewrite the coupling equations in matrix form. The mirror coupling as given in Fig. 5 becomes (Fig. 8)

and the amplitude coupling at a ‘space’, as given in Fig. 6, can be written as (Fig. 9)

In these examples the matrix simply transforms the impinging amplitudes into the outgoing amplitudes.

*Coupling matrices for numerical computations*

The matrices introduced above are useful for storing and displaying the coupling coefficients for the light fields. However, if we want to compute the fields in an optical system a different approach is required. An obvious application of linear coupling equations is to construct a large matrix representing extended optical system appropriate with one equation for each field amplitude. The matrix represents a set of linear equations whose solution is a vector with all light fields in the optical system. For example, the set of linear equations for a mirror would be written as

2.6 $$\begin{aligned}
								\left( \begin{array}{c@{\quad }c@{\quad }c@{\quad }c} 1 &{}0
								&{} 0 &{} 0 \\ -\mathrm {i}\,t &{}
								1&{} -r &{} 0\\ 0 &{}0 &{} 1
								&{} 0\\ -r &{} 0 &{} -\mathrm {i}\,t
								&{} 1 \end{array} \right) \left( \begin{array}{c} a_1\\
								a_2\\ a_3 \\ a_4\end{array} \right) = \left( \begin{array}{c} a_1\\
								0\\ a_3 \\ 0\end{array} \right) =M_{\mathrm {system}}~\mathbf
								{a}_{\mathrm {sol}}\,=\,\mathbf {a}_{\mathrm {input}},
								\end{aligned}$$

where the input vector^2^ $$\mathbf
								{a}_{\mathrm {input}}$$ has non-zero values for the impinging fields and $$\mathbf
								{a}_{\mathrm {sol}}$$ is the ‘solution’ vector, i.e., after solving the system of equations the amplitudes of the impinging as well as those of the outgoing fields are stored in that vector.

As an example we apply this method to the two mirror cavity. The system matrix for the optical setup shown in Fig. 7 becomes

2.7 $$\begin{aligned}
								\left( \begin{array}{c@{\quad }c@{\quad }c@{\quad }c@{\quad
								}c@{\quad }c@{\quad }c} 1 &{} 0 &{} 0 &{} 0
								&{} 0 &{} 0 &{} 0\\ -\mathrm {i}\,t_1
								&{} 1 &{} 0 &{} -r_1 &{} 0
								&{} 0 &{} 0\\ -r_1 &{} 0 &{} 1
								&{} -\mathrm {i}\,t_1 &{} 0 &{} 0
								&{} 0\\ 0 &{} 0 &{} 0 &{} 1
								&{} 0 &{} 0 &{} -e^{-\mathrm {i}\,k D}\\ 0
								&{} -e^{-\mathrm {i}\,k D} &{} 0 &{} 0
								&{} 1 &{} 0 &{} 0\\ 0 &{} 0
								&{} 0 &{} 0 &{} -\mathrm {i}\,t_2
								&{} 1 &{} 0\\ 0 &{} 0 &{} 0
								&{} 0 &{} -r_2 &{} 0 &{} 1
								\end{array} \right) \left( \begin{array}{c} a_0\\ a_1\\ a_4 \\
								a'_3\\ a'_1\\ a_2\\ a_3 \end{array} \right) = \left(
								\begin{array}{c} a_0\\ 0\\ 0 \\ 0\\ 0\\ 0\\ 0 \end{array} \right)
								\end{aligned}$$

This is a *sparse* matrix. Sparse matrices are an important subclass of linear algebra problems and many efficient numerical algorithms for solving sparse matrices are freely available (see, for example, Davis 2006). The advantage of this method of constructing a single matrix for an entire optical system is the direct access to all field amplitudes. It also stores each coupling coefficient in one or more dedicated matrix elements, so that numerical values for each parameter can be read out or changed after the matrix has been constructed and, for example, stored in computer memory. The obvious disadvantage is that the size of the matrix quickly grows with the number of optical elements (and with the degrees of freedom of the system, see, for example, Sect. 9).

*Coupling matrices for a compact system descriptions*

The following method is probably most useful for analytic computations, or for optimisation aspects of a numerical computation. The idea behind the scheme, which is used for computing the characteristics of dielectric coatings (Hecht 2002; Matuschek et al. 1997) and has been demonstrated for analysing gravitational wave detectors (Mizuno and Yamaguchi 1999), is to rearrange equations as in Figs. 8 and 9 such that the overall matrix describing a series of components can be obtained by multiplication of the component matrices. In order to achieve this, the coupling equations have to be re-ordered so that the input vector consists of two field amplitudes *at one side of the component*. For the mirror, this gives a coupling matrix of

2.8 $$\begin{aligned}
								\left( \begin{array}{c} a_1\\ a_4\end{array} \right) =\frac{\mathrm
								{i}\,}{t} \left( \begin{array}{c@{\quad }c} -1&{} r \\ -r
								&{} r^2+t^2 \end{array} \right) \left( \begin{array}{c}
								a_2\\ a_3\end{array} \right) .
								\end{aligned}$$

In the special case of the lossless mirror this matrix simplifies as we have $r^2+t^2=R+T=1$. The space component would be described by the following matrix:

2.9 $$\begin{aligned}
								\left( \begin{array}{c} a_1\\ a_4\end{array} \right) = \left(
								\begin{array}{c@{\quad }c} \exp (\mathrm {i}\,k D) &{} 0\\ 0
								&{} \exp (-\mathrm {i}\,k D)\end{array} \right) \left(
								\begin{array}{c} a_2\\ a_3\end{array} \right) .
								\end{aligned}$$

With these matrices we can very easily compute a matrix for the cavity with two lossless mirrors as

2.10 $$\begin{aligned}
								M_{\mathrm {cav}}= & {} M_{\mathrm {mirror1}}\times
								M_{\mathrm {space}}\times M_{\mathrm
								{mirror2}}\end{aligned}$$

2.11 $$\begin{aligned}=
								& {} \frac{-1}{t_1 t_2}\left( \begin{array}{c@{\quad }c}
								e^{+}-r_1 r_2 e^{-} &{} -r_2e^{+}+ r_1 e^{-} \\ -r_2e^{-}+
								r_1 e^{+} &{} e^{-}-r_1 r_2 e^{+} \end{array}\right) ,
								\end{aligned}$$

with $$e^{+}=\exp
								(\mathrm {i}\,k D)$$ and $$e^{-}=\exp
								(-\mathrm {i}\,k D)$$. The system of equation describing a cavity shown in Eq. (2.1) can now be written more compactly as

2.12 $$\begin{aligned}
								\left( \begin{array}{c} a_0\\ a_4\end{array} \right) = \frac{-1}{t_1
								t_2}\left( \begin{array}{c@{\quad }c} e^{+}-r_1 r_2 e^{-}
								&{} -r_2e^{+}+ r_1 e^{-} \\ -r_2e^{-}+ r_1 e^{+} &{}
								e^{-}-r_1 r_2 e^{+} \end{array}\right) \left( \begin{array}{c} a_2\\
								0\end{array} \right) . \end{aligned}$$

This allows direct computation of the amplitude of the transmitted field resulting in

2.13 $$\begin{aligned}
								a_2=a_0\frac{-t_1t_2\exp (-\mathrm {i}\,k D)}{1-r_1 r_2 \exp
								(-\mathrm {i}\,2 k D)}, \end{aligned}$$

which is the same as Eq. (2.5).

The advantage of this matrix method is that it allows compact storage of any series of mirrors and propagations, and potentially other optical elements, in a single $$2 \times
								2$$ matrix. The disadvantage inherent in this scheme is the lack of information about the field amplitudes inside the group of optical elements.

### Phase relation at a mirror or beam splitter

Throughout this article we use a slightly unintuitive definition for how the phase of a light field changes when in interacts with a mirror or beam splitter. In this section we motivate this convention and show in detail that it is, for our purposes, mathematical equivalent to other definitions.

**Fig. 10:**
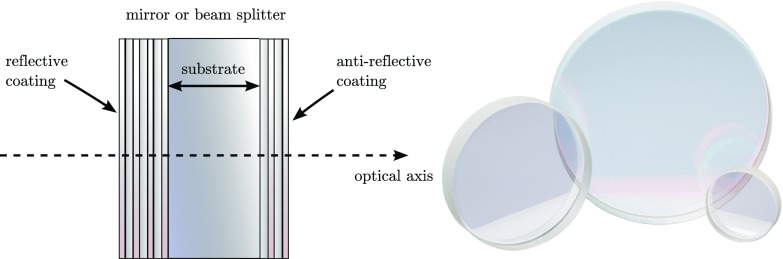
This sketch shows a mirror or beam splitter component with dielectric coatings and the photograph shows some typical commercially available examples (Newport Corporation 2008). Most mirrors and beam splitters used in optical experiments are of this type: a substrate made from glass, quartz or fused silica is coated on both sides. The *reflective coating* defines the overall reflectivity of the component (anything between $R\approx 1$ and $R\approx 0$, while the *anti-reflective coating* is used to reduce the reflection at the second optical surface as much as possible so that this surface does not influence the light. Please note that the drawing is not to scale, the coatings are typically only a few microns thick on a several millimetre to centimetre thick substrate

The magnitude and phase of reflection at a single optical surface can be derived from Maxwell’s equations and the electromagnetic boundary conditions at the surface, and in particular the condition that the field amplitudes tangential to the optical surface must be continuous. The results are called *Fresnel’s equations* (Kenyon 2008). Thus, for a field impinging on an optical surface under normal incidence we can give the reflection coefficient as

2.14 $$\begin{aligned}
								r=\frac{n_1-n_2}{n_1+n_2}, \end{aligned}$$

with $$n_1$$ and $$n_2$$ the indices of refraction of the first and second medium, respectively. The transmission coefficient for a lossless surface can be computed as $$t^2=1-r^2$$. We note that the phase change upon reflection is either 0 or 180°, depending on whether the second medium is optically thinner or thicker than the first. It is not shown here but Fresnel’s equations can also be used to show that the phase change for the transmitted light at a lossless surface is zero. This contrasts with the definitions given in Sect. 2.1 (see Fig. 5), where the phase shift upon any reflection is defined as zero and the transmitted light experiences a phase shift of $$\pi
								/2$$. The following section explains the motivation for the latter definition having been adopted as the common notation for the analysis of modern optical systems.

*Composite optical surfaces*

Modern mirrors and beam splitters that make use of dielectric coatings are complex optical systems, see Fig. 10 whose reflectivity and transmission depend on the multiple interference inside the coating layers and thus on microscopic parameters. The phase change upon transmission or reflection depends on the details of the applied coating and is typically not known. In any case, the knowledge of an absolute value of a phase change is typically not of interest in laser interferometers because the absolute positions of the optical components are not known to sub-wavelength precision. Instead the *relative* phase between the incoming and outgoing beams is of importance. In the following we demonstrate how constraints on these relative phases, i.e., the phase relation between the beams, can be derived from the fundamental principle of power conservation. To do this we consider a Michelson interferometer, as shown in Fig. 11, with perfectly-reflecting mirrors. The beam splitter of the Michelson interferometer is the object under test. We assume that the magnitude of the reflection *r* and transmission *t* are known. The phase changes upon transmission and reflection are unknown. Due to symmetry we can say that the phase change upon transmission $$\varphi
								_t$$ should be the same in both directions. However, the phase change on reflection might be different for either direction, thus, we write $$\varphi
								_{r1}$$ for the reflection at the front and $$\varphi
								_{r2}$$ for the reflection at the back of the beam splitter.

**Fig. 11:**
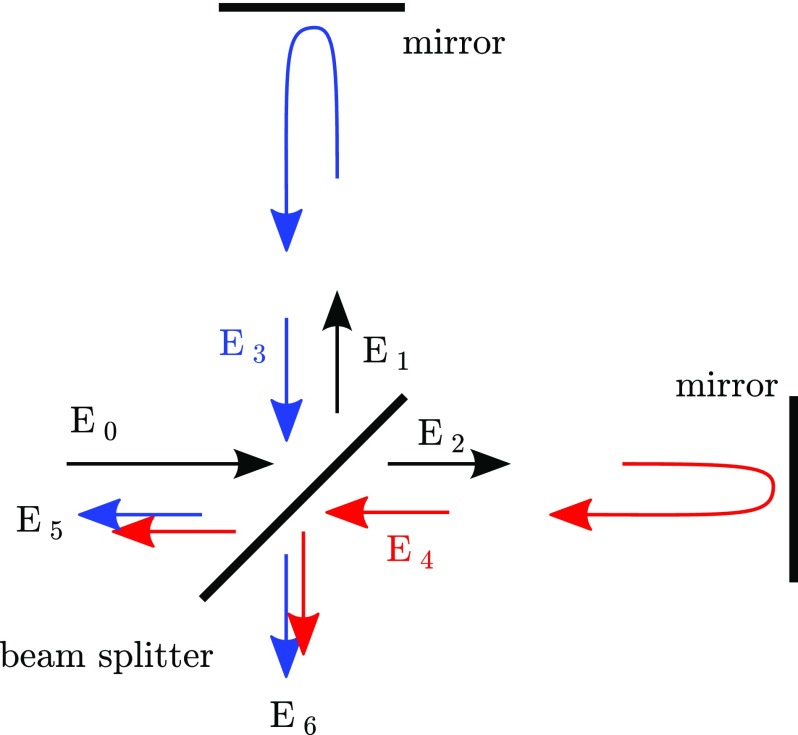
The relation between the phase of the light field amplitudes at a beam splitter can be computed assuming a Michelson interferometer, with arbitrary arm length but perfectly-reflecting mirrors. The incoming field $E_0$ is split into two fields $E_1$ and $E_2$ which are reflected at the end mirrors and return to the beam splitter, as $E_3$ and $E_4$, to be recombined into two outgoing fields. These outgoing fields $E_5$ and $E_6$ are depicted by *two arrows* to highlight that these are the sum of the transmitted and reflected components of the returning fields. We can derive constraints for the phase of $E_1$ and $E_2$ with respect to the input field $E_0$ from the conservation of energy: $|E_0|^2=|E_5|^2+|E_6|^2$

Then the electric fields can be computed as

2.15 $$\begin{aligned}
								E_1=r~ E_0~ e^{\mathrm {i}\,\varphi _{r1}};\quad E_2=t~ E_0~
								e^{\mathrm {i}\,\varphi _{t}}.
								\end{aligned}$$

We do not know the length of the interferometer arms. Thus, we introduce two further unknown phases: $$\varPhi
								_1$$ for the total phase accumulated by the field in the vertical arm and $$\varPhi
								_2$$ for the total phase accumulated in the horizontal arm. The fields impinging on the beam splitter compute as

2.16 $$\begin{aligned}
								E_3=r~ E_0~ e^{\mathrm {i}\,(\varphi _{r1}+\varPhi _1)};\quad E_4=t~
								E_0~ e^{\mathrm {i}\,(\varphi _{t}+\varPhi _2)}.
								\end{aligned}$$

The outgoing fields are computed as the sums of the reflected and transmitted components:

2.17 $$\begin{aligned}
								E_5= & {} E_0\left( R~e^{\mathrm {i}\,(2\varphi
								_{r1}+\varPhi _1)}~+~T~e^{\mathrm {i}\,(2\varphi _{t}+\varPhi
								_2)}\right) \nonumber \\ E_6= & {} E_0~rt\left( e^{\mathrm
								{i}\,(\varphi _{t}+\varphi _{r1}+\varPhi _1)}~+~e^{\mathrm
								{i}\,(\varphi _{t}+\varphi _{r2}+\varPhi _2)}\right) ,
								\end{aligned}$$

with $$R=r^2$$ and $$T=t^2$$.

It will be convenient to separate the phase factors into common and differential ones. We can write

2.18 $$\begin{aligned}
								E_5=E_0~e^{\mathrm {i}\,\alpha _{+}}\left( R~e^{\mathrm {i}\,\alpha
								_{-}}~+~T~e^{-\mathrm {i}\,\alpha _{-}}\right) ,
								\end{aligned}$$

with

2.19 $$\begin{aligned}
								\alpha _{+}=\varphi _{r1}+\varphi _t+\frac{1}{2}\left( \varPhi
								_1+\varPhi _2\right) ;\quad \alpha _{-}=\varphi _{r1}-\varphi
								_t+\frac{1}{2}\left( \varPhi _1-\varPhi _2\right) ,
								\end{aligned}$$

and similarly

2.20 $$\begin{aligned}
								E_6=E_0~rt~e^{\mathrm {i}\,\beta _{+}}~2\cos (\beta _{-}),
								\end{aligned}$$

with

2.21 $$\begin{aligned}
								\beta _{+}=\varphi _t+\frac{1}{2}\left( \varphi _{r1}+\varphi
								_{r2}+\varPhi _1+\varPhi _2\right) ;\quad \beta
								_{-}=\frac{1}{2}\left( \varphi _{r1}-\varphi _{r2}+\varPhi
								_1-\varPhi _2\right) . \end{aligned}$$

For simplicity we now limit the discussion to a 50:50 beam splitter with $r=t=1/\sqrt{2}$, for which we can simplify the field expressions even further:

2.22 $$\begin{aligned}
								E_5=E_0~e^{\mathrm {i}\,\alpha _{+}}\cos (\alpha _{-});\quad
								E_6=E_0~e^{\mathrm {i}\,\beta _{+}}~\cos (\beta _{-}).
								\end{aligned}$$

Conservation of energy requires that $|E_0|^2=|E_5|^2+|E_6|^2$, which in turn requires

2.23 $$\begin{aligned}
								\cos ^2(\alpha _{-})+\cos ^2(\beta _{-})=1,
								\end{aligned}$$

which is only true if

2.24 $$\begin{aligned}
								\alpha _{-}-\beta _{-}=(2N+1)\frac{\pi }{2},
								\end{aligned}$$

with *N* as in integer (positive, negative or zero). This gives the following constraint on the phase factors

2.25 $$\begin{aligned}
								\frac{1}{2}\left( \varphi _{r1}+\varphi _{r2}\right) -\varphi
								_{t}=(2N+1)\frac{\pi }{2}. \end{aligned}$$

One can show that exactly the same condition results in the case of arbitrary (lossless) reflectivity of the beam splitter (Rüdiger 1998).

We can test whether two known examples fulfil this condition. If the beam-splitting surface is the front of a glass plate we know that $$\varphi _t=0,
								\varphi _{r1}=\pi , \varphi _{r2}=0$$, which conforms with Eq. (2.25). A second example is the two-mirror resonator, see Sect. 2.2. If we consider the cavity as an optical ‘black box’, it also splits any incoming beam into a reflected and transmitted component, like a mirror or beam splitter. Further we know that a symmetric resonator must give the same results for fields injected from the left or from the right. Thus, the phase factors upon reflection must be equal $$\varphi
								_r=\varphi _{r1}=\varphi _{r2}$$. The reflection and transmission coefficients are given by Eqs. (2.4) and (2.5) as

2.26 $$\begin{aligned}
								r_{\mathrm {cav}}=\left( r_1 - \frac{r_2 t_1^2 \exp (-\mathrm {i}\,2
								k D)}{1-r_1 r_2 \exp (- \mathrm {i}\,2 k D)}\right) ,
								\end{aligned}$$

and

2.27 $$\begin{aligned}
								t_{\mathrm {cav}}=\frac{-t_1 t_2 \exp (-\mathrm {i}\,k D)}{1-r_1 r_2
								\exp (- \mathrm {i}\,2 k D)}.
								\end{aligned}$$

We demonstrate a simple case by putting the cavity on resonance ($$k D=N\pi
								$$). This yields

2.28 $$\begin{aligned}
								r_{\mathrm {cav}}=\left( r_1 - \frac{r_2 t_1^2}{1-r_1 r_2}\right)
								;\quad t_{\mathrm {cav}}=\frac{\mathrm {i}\,~t_1 t_2}{1-r_1 r_2},
								\end{aligned}$$

with $$r_{\mathrm
								{cav}}$$ being purely real and $$t_{\mathrm
								{cav}}$$ imaginary and thus $$\varphi _t=\pi
								/2$$ and $$\varphi
								_r=0$$ which also agrees with Eq. (2.25).

In most cases we neither know nor care about the exact phase factors. Instead we can pick any set which fulfils Eq. (2.25). For this document we have chosen to use phase factors equal to those of the cavity, i.e., $$\varphi _t=\pi
								/2$$ and $$\varphi
								_r=0$$, which is why we write the reflection and transmission at a mirror or beam splitter as

2.29 $$\begin{aligned}
								E_{\mathrm {refl}}=r ~E_0\quad \mathrm {and}\quad E_{\mathrm
								{trans}}=\mathrm {i}\,~t~ E_0.
								\end{aligned}$$

In this definition *r* and *t* are positive real numbers satisfying $r^2+t^2=1$ for the lossless case. This definition is convenient due to its symmetry, for example, it allows to specify mirrors and beamsplitters without defining a front and back face.

Please note that we only have the freedom to chose convenient phase factors when we do not know or do not care about the details of the coating, which performs the beam splitting. If instead the details are important, for example, when computing the properties of a thin coating layer, such as anti-reflex coatings, the proper phase factors for the respective interfaces must be computed and used. Similarly, for a simple glass plate this convention cannot be used.

### Lengths and tunings: numerical accuracy of distances

The resonance condition inside an optical cavity and the operating point of an interferometer depends on the optical path lengths modulo the laser wavelength, i.e., for light from an Nd:YAG laser length differences of less than $$1~\upmu \hbox
								{m}$$ are of interest, not the full magnitude of the distances between optics. On the other hand, several parameters describing the general properties of an optical system, like the finesse or free spectral range of a cavity (see Sect. 5.1) depend on the macroscopic distance and do not change significantly when the distance is changed on the order of a wavelength. This illustrates that the distance between optical components might not be the best parameter to use for the analysis of optical systems. Furthermore, it turns out that in numerical algorithms the distance may suffer from rounding errors. Let us use the Virgo (2015) arm cavities as an example to illustrate this. The cavity length is approximately 3 km, the wavelength is on the order of $$1~\upmu \hbox
								{m}$$, the mirror positions are actively controlled with a precision of 1 pm and the detector sensitivity can be as good as $$10^{-18}$$ m, measured on $${\sim
								}10$$ ms timescales (i.e., many samples of the data acquisition rate). The floating point accuracy of common, fast numerical algorithms is typically not better than $$10^{-15}$$. If we were to store the distance between the cavity mirrors as such a floating point number, the accuracy would be limited to 3 pm, which does not even cover the accuracy of the control systems, let alone the sensitivity (Fig. 12).

**Fig. 12:**
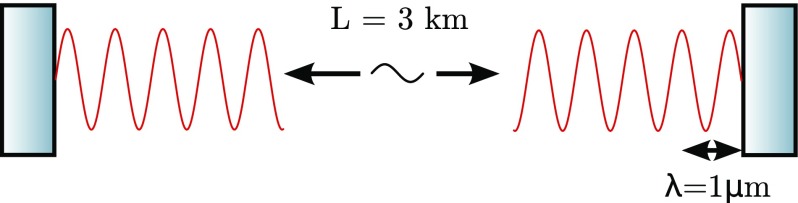
Illustration of an arm cavity of the Virgo gravitational-wave detector (Virgo 2015): the macroscopic length *L* of the cavity is approximately 3 km, while the wavelength of the Nd:YAG laser is $$\lambda \approx 1\,\upmu \mathrm
											{m}$$. The resonance condition is only affected by the microscopic position of the wave nodes with respect to the mirror surfaces and not by the macroscopic length, i.e., displacement of one mirror by $$\varDelta x=\lambda
											/2$$ re-creates exactly the same condition. However, other parameters of the cavity, such as the finesse, only depend on the macroscopic length *L* and not on the microscopic tuning

A simple and elegant solution to this problem is to split a distance *D* between two optical components into two parameters (Heinzel 1999): one is the macroscopic ‘length’ *L*, defined as the multiple of a constant wavelength $$\lambda
								_0$$ yielding the smallest difference to *D*. The second parameter is the microscopic *tuning* *T* that is defined as the remaining difference between *L* and *D*, i.e., $$D=L+T$$. Typically, $$\lambda
								_0$$ can be understood as the wavelength of the laser in vacuum, however, if the laser frequency changes during the experiment or multiple light fields with different frequencies are used simultaneously, a default constant wavelength must be chosen arbitrarily. Please note that usually the term $$\lambda
								$$ in any equation refers to the actual wavelength at the respective location as $$\lambda =\lambda
								_0/n$$ with *n* the index of refraction at the local medium.

We have seen in Sect. 2.1 that distances appear in the expressions for electromagnetic waves in connection with the wavenumber, for example,

2.30 $$\begin{aligned}
								E_2=E_1~\exp (-\mathrm {i}\,k z).
								\end{aligned}$$

Thus, the difference in phase between the field at $z=z_1$ and $z=z_1+D$ is given as

2.31 $$\begin{aligned}
								\varphi =- k D. \end{aligned}$$

We recall that $$k=2\pi /\lambda
								=\omega /c$$. We can define $$\omega _0=2\pi
								~c/\lambda _0$$ and $$k_0=\omega
								_0/c$$. For any given wavelength $$\lambda
								$$ we can write the corresponding frequency as a sum of the default frequency and a difference frequency $$\omega =\omega
								_0+\varDelta \omega $$. Using these definitions, we can rewrite Eq. (2.31) with length and tuning as

2.32 $$\begin{aligned}
								-\varphi = k D = \frac{\omega _0 L}{c} + \frac{\varDelta \omega
								L}{c} + \frac{\omega _0 T}{c}+ \frac{\varDelta \omega T}{c}.
								\end{aligned}$$

The first term of the sum is always a multiple of $$2\pi
								$$, which is equivalent to zero. The last term of the sum is the smallest, approximately of the order $$\varDelta \omega
								\cdot 10^{-14}$$. For typical values of $$L\approx
								1\mathrm {\ m}$$, $$T<1\
								\upmu \mathrm {m}$$ and $$\varDelta \omega
								<2\pi \cdot 100\mathrm {\ MHz}$$ we find that

2.33 $$\begin{aligned}
								\frac{\omega _0 L}{c} =0,\quad \frac{\varDelta \omega
								L}{c}\lessapprox 2,\quad \frac{\omega _0 T}{c}\lessapprox 6,\quad
								\frac{\varDelta \omega T}{c} \lessapprox 2~10^{-6},
								\end{aligned}$$

which shows that the last term can often be ignored.

We can also write the tuning directly as a phase. We define as the dimensionless tuning

2.34 $$\begin{aligned}
								\phi =\omega _0 T/c. \end{aligned}$$

This yields

2.35 $$\begin{aligned}
								\exp \left( \mathrm {i}\,\frac{\omega }{c}T\right) =\exp \left(
								\mathrm {i}\,\frac{\omega _0}{c}T\frac{\omega }{\omega _0}\right) =
								\exp \left( \mathrm {i}\,\frac{\omega }{\omega _0} \phi \right) .
								\end{aligned}$$

The tuning $$\phi
								$$ is given in radian with $$2\pi
								$$ referring to a microscopic distance of one wavelength^3^ $$\lambda
								_0$$.

Finally, we can write the following expression for the phase difference between the light field taken at the end points of a distance *D*:

2.36 $$\begin{aligned}
								\varphi =- k D =-\left( \frac{\varDelta \omega L}{c} + \phi
								\frac{\omega }{\omega _0}\right) ,
								\end{aligned}$$

or if we neglect the last term from Eq. (2.33) we can approximate ($$\omega /\omega
								_0\approx 1$$) to obtain

2.37 $$\begin{aligned}
								\varphi \approx -\left( \frac{\varDelta \omega L}{c} + \phi \right)
								. \end{aligned}$$

This convention provides two parameters *L* and $$\phi
								$$, that can describe distances with a markedly improved numerical accuracy. In addition, this definition often allows simplification of the algebraic notation of interferometer signals. By convention we associate a length *L* with the propagation through free space, whereas the tuning will be treated as a parameter of the optical components. Effectively the tuning then represents a microscopic *displacement* of the respective component. If, for example, a cavity is to be resonant to the laser light, the tunings of the mirrors have to be the same whereas the length of the space in between can be arbitrary.

### Revised coupling matrices for space and mirrors

**Fig. 13:**
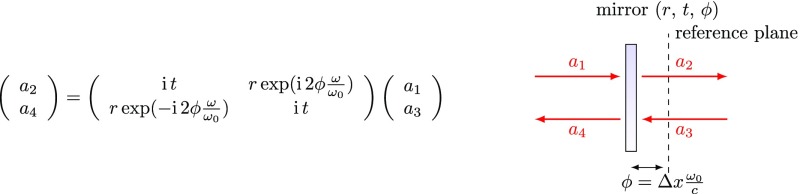
Revised coupling matrix for field amplitudes at a mirror

**Fig. 14:**
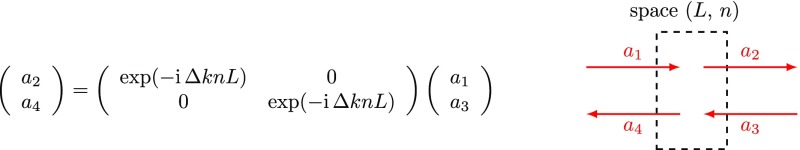
Revised coupling matrix for field amplitudes at a space

Using the definitions for length and tunings we can rewrite the coupling equations for mirrors and spaces introduced in Sect. 2.1 as follows. The mirror coupling becomes (Fig. 13)

(compare this to Fig. 8), and the amplitude coupling for a ‘space’, formally written as in Fig. 9, is now written as (Fig. 14).

### Finesse examples

#### Mirror reflectivity and transmittance

**Fig. 15:**
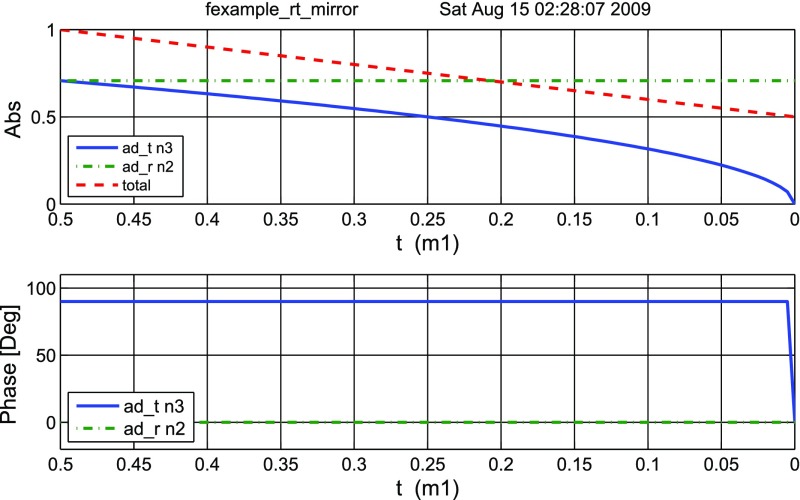
Finesse example: mirror reflectivity and transmittance

We use Finesse to plot the amplitudes of the light fields transmitted and reflected by a mirror (given by a single surface). Initially, the mirror has a power reflectance and transmittance of $R=T=0.5$ and is, thus, lossless. For the plot in Fig. 15 we tune the transmittance from 0.5 to 0. Since we do not explicitly change the reflectivity, *R* remains at 0.5 and the mirror loss increases instead, which is shown by the trace labelled ‘total’ corresponding to the sum of the reflected and transmitted light power. The plot also shows the phase convention of a 90° phase shift for the transmitted light.

**Finesse input file for ‘Mirror reflectivity and transmittance’** 
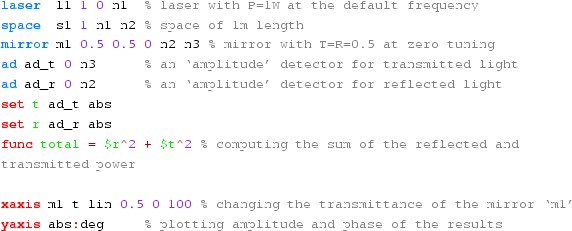


#### Length and tunings

**Fig. 16:**
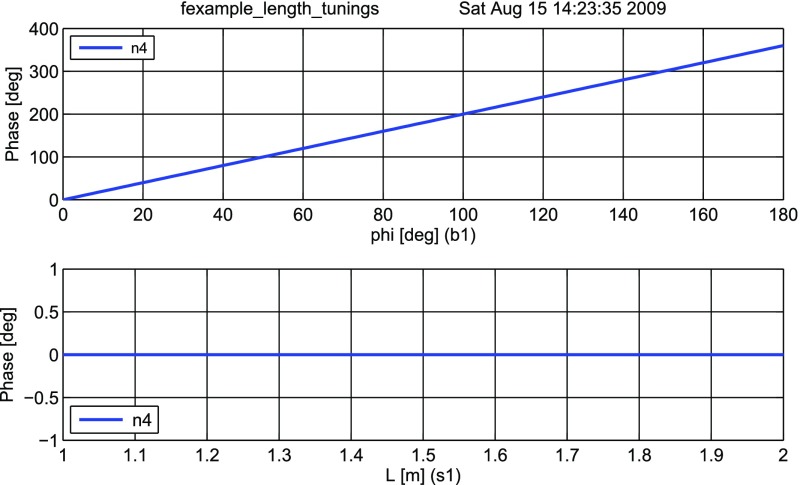
Finesse example: Length and tunings

These Finesse files demonstrate the conventions for lengths and microscopic positions introduced in Sect. 2.5. The top trace in Fig. 16 depicts the phase change of a beam reflected by a beam splitter as the function of the beam splitter tuning. By changing the tuning from 0 to 180° the beam splitter is moved forward and shortens the path length by one wavelength, which by convention increases the light phase by 360°. On the other hand, if a length of a space is changed, the phase of the transmitted light is unchanged (for the default wavelength $\varDelta k=0$), as shown in the lower trace.

**Finesse input files for ‘Length and tunings’** 
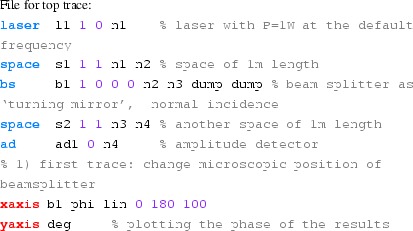
 
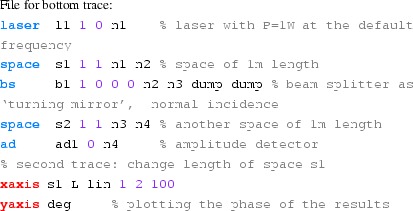


## Light with multiple frequency components

So far we have considered the electromagnetic field to be monochromatic. This has allowed us to compute light-field amplitudes in a quasi-static optical setup. In this section, we introduce the frequency of the light as a new degree of freedom. In fact, we consider a field consisting of a finite and discrete number of frequency components. We write this as

3.1 $$\begin{aligned}
							E(t,z)=\sum _{j}~a_{j}~\exp {\left( \mathrm {i}\,(\omega _j
							\,t-k_jz)\right) }, \end{aligned}$$

with complex amplitude factors $$a_{j}$$, $$\omega
							_j$$ as the angular frequency of the light field and $$k_j=\omega
							_j/c$$. In many cases the analysis compares different fields at one specific location only, in which case we can set $$z=0$$ and write

3.2 $$\begin{aligned}
							E(t)=\sum _{j}~a_{j}~\exp {\left( \mathrm {i}\,\omega _j \,t\right) }.
							\end{aligned}$$

In the following sections the concept of light modulation is introduced. As this inherently involves light fields with multiple frequency components, it makes use of this type of field description. Again we start with the two-mirror cavity to illustrate how the concept of modulation can be used to model the effect of mirror motion.

### Modulation of light fields

Laser interferometers typically use three different types of light fields: the laser with a frequency of, for example, $$f\approx
								2.8\cdot 10^{14}\mathrm {\ Hz}$$, *radio frequency* (RF) sidebands used for interferometer control with frequencies (offset to the laser frequency) of $$f\approx 1\cdot
								10^{6}$$ to $$150\cdot
								10^{6}\mathrm {\ Hz}$$, and the *signal* sidebands at frequencies of 1–10 000 Hz.^4^ As these modulations usually have as their origin a change in optical path length, they are often phase modulations of the laser frequency, the RF sidebands are utilised for optical readout purposes, while the signal sidebands carry the signal to be measured (the gravitational-wave signal plus noise created in the interferometer).

Figure 17 shows a *time domain* representation of an electromagnetic wave of frequency $$\omega
								_0$$, whose amplitude or phase is modulated at a frequency $$\varOmega
								$$. One can easily see some characteristics of these two types of modulation, for example, that amplitude modulation leaves the zero crossing of the wave unchanged whereas with phase modulation the maximum and minimum amplitude of the wave remains the same. In the *frequency domain* in which a modulated field is expanded into several unmodulated field components, the interpretation of modulation becomes even easier: any sinusoidal modulation of amplitude or phase generates new field components, which are shifted in frequency with respect to the initial field. Basically, light power is shifted from one frequency component, the *carrier*, to several others, the *sidebands*. The relative amplitudes and phases of these sidebands differ for different types of modulation and different modulation strengths. This section demonstrates how to compute the sideband components for amplitude, phase and frequency modulation.

**Fig. 17:**
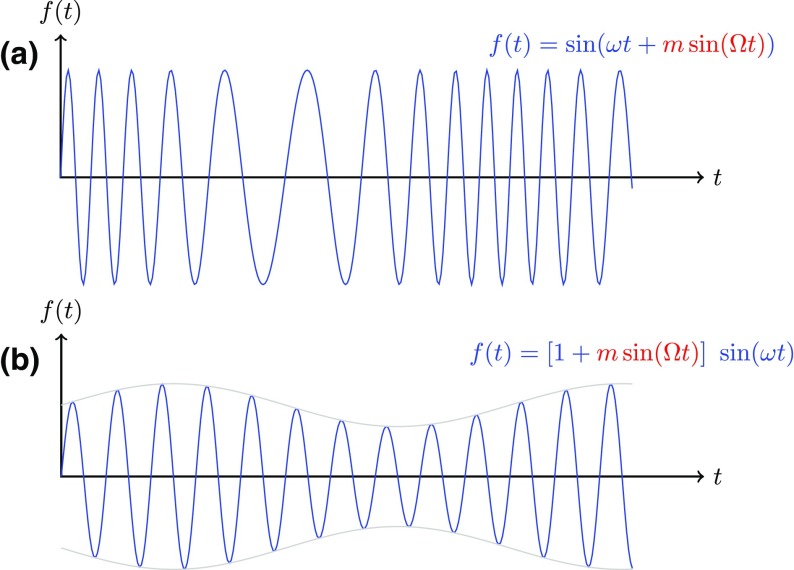
Example traces for phase and amplitude modulation: the *upper plot* **a** shows a phase-modulated sine wave and the *lower plot* **b** depicts an amplitude-modulated sine wave. Phase modulation is characterised by the fact that it mostly affects the zero crossings of the sine wave. Amplitude modulation affects mostly the maximum amplitude of the wave. The equations show the modulation terms in *red* with *m* the modulation index and $\varOmega$ the modulation frequency

### Phase modulation

Phase modulation can create a large number of sidebands. The number of sidebands with noticeable power depends on the modulation strength (or depth) given by the *modulation index* *m*. Assuming an input field

3.3 $$\begin{aligned}
								E_{\mathrm {in}}=E_0~\exp {\left( \mathrm {i}\,\omega _0 \,t\right)
								}, \end{aligned}$$

a sinusoidal phase modulation of the field can be described as

3.4 $$\begin{aligned}
								E=E_0~\exp {\Bigl (\mathrm {i}\,(\omega _0 \,t+ m \cos {\left(
								\varOmega \,t\right) })\Bigr )}.
								\end{aligned}$$

This equation can be expanded using the identity (Gradshteyn and Ryzhik 1994)

3.5 $$\begin{aligned}
								\exp (\mathrm {i}\,z \cos \varphi )=\sum _{k=-\infty }^\infty
								\mathrm {i}\,^kJ_k(z)\exp (\mathrm {i}\,k \varphi ),
								\end{aligned}$$

**Fig. 18:**
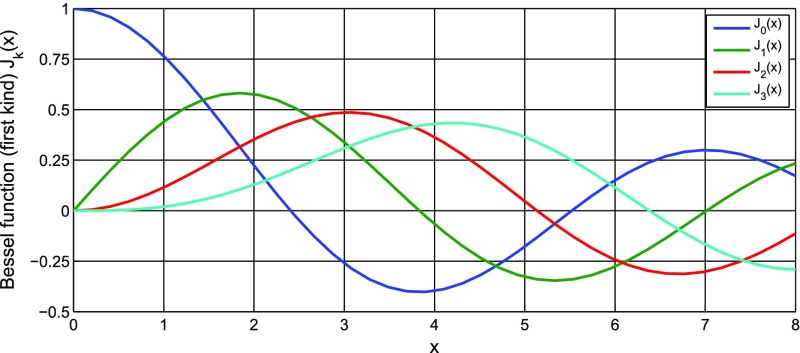
Some of the lowest-order Bessel functions $J_k(x)$ of the first kind. For small *x* the expansion shows a simple $x^k$ dependency and higher-order functions can often be neglected

with *Bessel functions of the first kind* $J_k(m)$. We can write

3.6 $$\begin{aligned}
								E=E_0~\exp {\left( \mathrm {i}\,\omega _0 \,t\right) }~\sum
								_{k=-\infty }^{\infty }\mathrm {i}\,^{k}~J_k(m)~\exp {\left( \mathrm
								{i}\,k \varOmega \,t\right) }.
								\end{aligned}$$

The field for $$k=0$$, oscillating with the frequency of the input field $$\omega
								_0$$, represents the carrier. The sidebands can be divided into *upper* ($$k>0$$) and *lower* ($$k<0$$) sidebands. These sidebands are light fields that have been shifted in frequency by $$k\, \varOmega
								$$. The upper and lower sidebands with the same absolute value of *k* are called a pair of sidebands of order *k*. Equation (3.6) shows that the carrier is surrounded by an infinite number of sidebands. However, for small modulation indices ($$m<1$$) the Bessel functions rapidly decrease with increasing *k* (the lowest orders of the Bessel functions are shown in Fig. 18). For small modulation indices we can use the approximation (Abramowitz and Stegun 1965)

3.7 $$\begin{aligned}
								J_k(m)~=\left( \frac{m}{2}\right) ^k\sum _{n=0}^\infty \frac{\left(
								-\frac{m^2}{4}\right) ^n}{n! (k+n)!}=\frac{1}{k!}\left(
								\frac{m}{2}\right) ^k+O\left( m^{k+2}\right) .
								\end{aligned}$$

In which case, only a few sidebands have to be taken into account. For $$m\ll
								1$$ we can write

3.8 $$\begin{aligned}
								E= & {} E_0~\exp {\left( \mathrm {i}\,\omega _0 \,t\right)
								}\nonumber \\&\times \,\Bigl (J_0(m)-\mathrm
								{i}\,J_{-1}(m)~\exp {\left( -\mathrm {i}\,\varOmega \,t\right)
								}+\mathrm {i}\,J_{1}(m)~\exp {\left( \mathrm {i}\,\varOmega
								\,t\right) }\Bigr ), \end{aligned}$$

and with

3.9 $$\begin{aligned}
								J_{-k}(m)=(-1)^kJ_k(m), \end{aligned}$$

we obtain

3.10 $$\begin{aligned}
								E=E_0~\exp {\left( \mathrm {i}\,\omega _0 \,t\right) }~\left(
								1+\mathrm {i}\,\frac{m}{2}\Bigl (\exp {\left( -\mathrm
								{i}\,\varOmega \,t\right) }+\exp {\left( \mathrm {i}\,\varOmega
								\,t\right) }\Bigr )\right) ,
								\end{aligned}$$

as the first-order approximation in *m*. In the above equation the carrier field remains unchanged by the modulation, therefore this approximation is not the most intuitive. It is clearer if the approximation up to the second order in *m* is given:

3.11 $$\begin{aligned}
								E=E_0~\exp {\left( \mathrm {i}\,\omega _0 \,t\right) }~\left(
								1-\frac{m^2}{4}+\mathrm {i}\,\frac{m}{2}\Bigl (\exp {\left( -\mathrm
								{i}\,\varOmega \,t\right) }+\exp {\left( \mathrm {i}\,\varOmega
								\,t\right) }\Bigr )\right) ,
								\end{aligned}$$

which shows that power is transferred from the carrier to the sideband fields.

Higher-order expansions in *m* can be performed simply by specifying the highest order of Bessel function, which is to be used in the sum in Eq. (3.6), i.e.,

3.12 $$\begin{aligned}
								E=E_0~\exp {\left( \mathrm {i}\,\omega _0 \,t\right) }~\sum
								_{k=-order}^{order}i^{\,k}~J_k(m)~\exp {\left( \mathrm {i}\,k
								\varOmega \,t\right) }. \end{aligned}$$

### Frequency modulation

For small modulation, indices, phase modulation and frequency modulation can be understood as different descriptions of the same effect (Heinzel 1999). Following the same spirit as above we would assume a modulated frequency to be given by

3.13 $$\begin{aligned}
								\omega =\omega _0+m'\cos {\left( \varOmega \,t\right) },
								\end{aligned}$$

and then we might be tempted to write

3.14 $$\begin{aligned}
								E=E_0~\exp {\Bigl (\mathrm {i}\,(\omega _0 + m' \cos {\left(
								\varOmega \,t\right) })\,t\Bigr )},
								\end{aligned}$$

which would be wrong. The frequency of a wave is actually defined as $$\omega /(2\pi
								)=f= d\varphi /dt$$. Thus, to obtain the frequency given in Eq. (3.13), we need to have a phase of

3.15 $$\begin{aligned}
								\omega _0\,t+ \frac{m'}{\varOmega } \sin {\left( \varOmega
								\,t\right) }. \end{aligned}$$

For consistency with the notation for phase modulation, we define the modulation index to be

3.16 $$\begin{aligned}
								m=\frac{m'}{\varOmega }=\frac{\varDelta \omega }{\varOmega },
								\end{aligned}$$

with $$\varDelta \omega
								$$ as the frequency swing—how *far* the frequency is shifted by the modulation—and $$\varOmega
								$$ the modulation frequency—how *fast* the frequency is shifted. Thus, a sinusoidal frequency modulation can be written as

3.17 $$\begin{aligned}
								E=E_0\exp {\left( \mathrm {i}\,\varphi \right) }=E_0~\exp {\left(
								\mathrm {i}\,\left( \omega _0\,t+ \frac{\varDelta \omega }{\varOmega
								} \cos {\left( \varOmega \,t\right) }\right) \right) },
								\end{aligned}$$

which is exactly the same expression as Eq. (3.4) for phase modulation. The practical difference is the typical size of the modulation index, with phase modulation having a modulation index of $m<10$, while for frequency modulation, typical numbers might be $m>10^4$. Thus, in the case of frequency modulation, the approximations for small *m* are not valid. The series expansion using Bessel functions, as in Eq. (3.6), can still be performed; however, very many terms of the resulting sum need to be taken into account.

### Amplitude modulation

In contrast to phase modulation, (sinusoidal) amplitude modulation always generates exactly two sidebands. Furthermore, a natural maximum modulation index exists: the modulation index is defined to be one ($m=1$) when the amplitude is modulated between zero and the amplitude of the unmodulated field.

If the amplitude modulation is performed by an active element, for example by modulating the current of a laser diode, the following equation can be used to describe the output field:

3.18 $$\begin{aligned}
								E= & {} E_0~\exp {\left( \mathrm {i}\,\omega _0 \,t\right)
								}~\Bigl (1+m\cos {\left( \varOmega \,t\right) }\Bigr )\nonumber \\=
								& {} E_0~\exp {\left( \mathrm {i}\,\omega _0 \,t\right)
								}~\Bigl (1+\frac{m}{2}~\exp {\left( \mathrm {i}\,\varOmega
								\,t\right) }+\frac{m}{2}~\exp {\left( -\mathrm {i}\,\varOmega
								\,t\right) }\Bigr ). \end{aligned}$$

However, passive amplitude modulators (like acousto-optic modulators or electro-optic modulators with polarisers) can only reduce the amplitude. In these cases, the following equation is more useful:

3.19 $$\begin{aligned}
								E= & {} E_0~\exp {\left( \mathrm {i}\,\omega _0 \,t\right)
								}~\left( 1-\frac{m}{2}\Bigl (1-\cos {\left( \varOmega \,t\right)
								}\Bigr )\right) \nonumber \\= & {} E_0~\exp {\left( \mathrm
								{i}\,\omega _0 \,t\right) }~\Bigl (1-\frac{m}{2}+\frac{m}{4}~\exp
								{\left( \mathrm {i}\,\varOmega \,t\right) }+\frac{m}{4}~\exp {\left(
								-\mathrm {i}\,\varOmega \,t\right) }\Bigr ).
								\end{aligned}$$

### Sidebands as phasors in a rotating frame

A common method of visualising the behaviour of sideband fields in interferometers is to use *phase diagrams* in which each field amplitude is represented by an arrow in the complex plane.

We can think of the electric field amplitude $$E_0\exp (\mathrm
								{i}\,\omega _0 t)$$ as a vector in the complex plane, rotating around the origin with angular velocity $$\omega
								_0$$. To illustrate or to help visualise the addition of several light fields it can be useful to look at this problem using a *rotating reference frame*, defined as follows. A complex number shall be defined as $$z=x+\mathrm
								{i}\,y$$ so that the real part is plotted along the *x*-axis, while the *y*-axis is used for the imaginary part. We want to construct a new coordinate system ($$x^{\prime
								}$$, $$y^{\prime
								}$$) in which the field vector is at a constant position. This can be achieved by defining

3.20 $$\begin{aligned}
								x= & {} x^{\prime }\ \cos \omega _0 t - y^{\prime }\ \sin
								\omega _0 t\nonumber \\ y= & {} x^{\prime }\ \sin \omega _0
								t + y^{\prime }\ \cos \omega _0 t,
								\end{aligned}$$

or

3.21 $$\begin{aligned}
								x^{\prime }= & {} x\ \cos \left( -\omega _0 t\right) - y\
								\sin \left( -\omega _0 t \right) \nonumber \\ y^{\prime }= &
								{} x\ \sin \left( -\omega _0 t \right) + y\ \cos \left( -\omega _0 t
								\right) . \end{aligned}$$

**Fig. 19:**
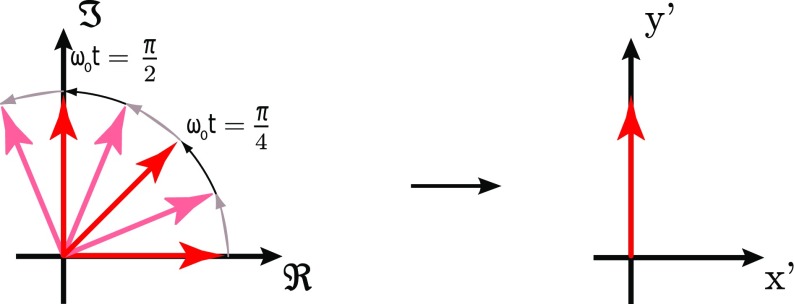
Electric field vector $$E_0\exp (\mathrm {i}\,\omega _0
											t)$$ depicted in the complex plane and in a rotating frame ($$x^{\prime }$$, $$y^{\prime }$$) rotating at $$\omega _0$$ so that the field vector appears stationary

**Fig. 20:**
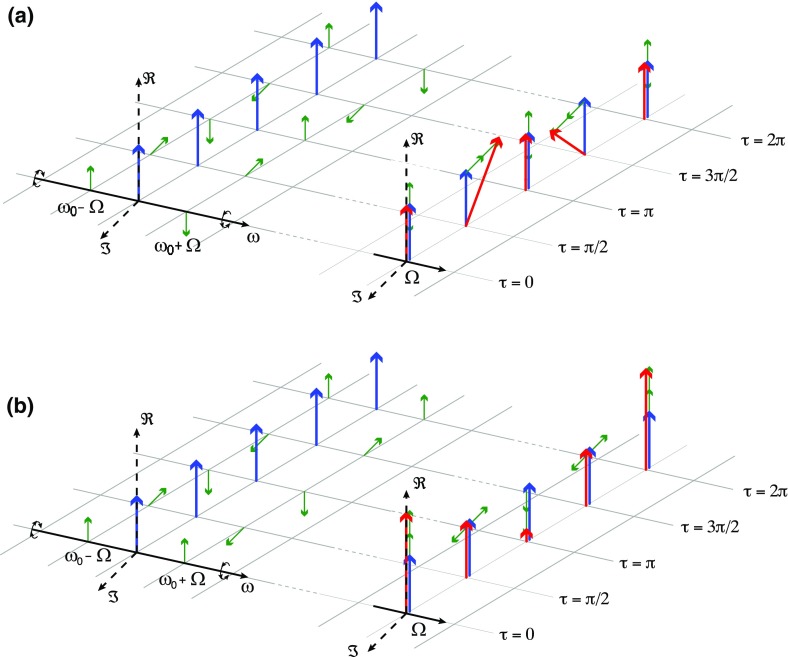
Amplitude and phase modulation in the ‘phasor’ picture. The *upper plots* **a** illustrate how a phasor diagram can be used to describe phase modulation, while the *lower plots* **b** do the same for amplitude modulation. In both cases the *left hand plot* shows the carrier in *blue* and the modulation sidebands in *green* as snapshots at certain time intervals. One can see clearly that the upper sideband ($$\omega _0+\varOmega
											$$) rotates faster than the carrier, while the lower sideband rotates slower. The *right plot* in both cases shows how the total field vector at any given time can be constructed by adding the three field vectors of the carrier and sidebands. [Drawing courtesy of Simon Chelkowski]

Figure 19 illustrates how the transition into the rotating frame makes the field vector to appear stationary. The angle of the field vector in a rotating frame depicts the phase offset of the field. Therefore these vectors are also called *phasors* and the illustrations using phasors are called *phasor diagrams*. Two more complex examples of how phasor diagrams can be employed is shown in Fig. 20 (Chelkowski 2007).

Phasor diagrams can be especially useful to see how frequency coupling of light field amplitudes can change the type of modulation, for example, to turn phase modulation into amplitude modulation. An extensive introduction to this type of phasor diagram can be found in Malec (2006).

### Phase modulation through a moving mirror

Several optical components can modulate transmitted or reflected light fields. In this section we discuss in detail the example of phase modulation by a moving mirror. Mirror motion does not change the transmitted light; however, the phase of the reflected light will be changed as shown in Eq. (13).

**Fig. 21:**
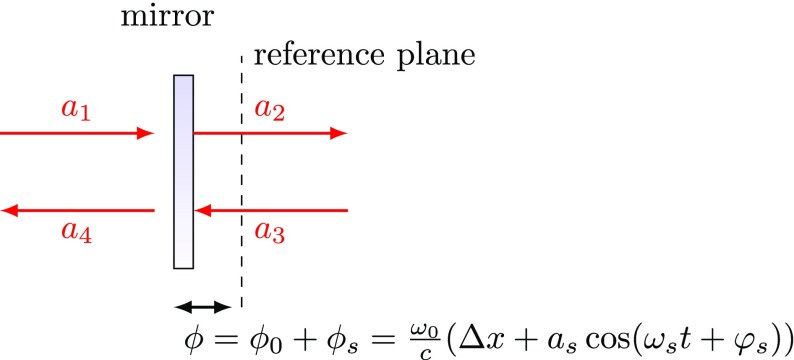
A sinusoidal signal with amplitude $$a_s$$ frequency $$\omega _s$$ and phase offset $$\varphi _s$$ is applied to a mirror position, or to be precise, to the mirror tuning. The equation given for the tuning $$\phi $$ assumes that $$\omega _s/\omega _0 \ll
											1$$, see Sect. 2.5

We assume sinusoidal change of the mirror’s tuning as shown in Fig. 21. The position modulation is given as $$x_{\mathrm
								{m}}=a_{\mathrm {s}}\cos (\omega _{\mathrm {s}}t+\varphi _{\mathrm
								{s}})$$, and thus the reflected field at the mirror becomes (assuming $$a_4=0$$)

3.22 $$\begin{aligned}
								a_3=r\, a_1 \exp (-\mathrm {i}\,2\phi _0)\,\exp {\left( \mathrm
								{i}\,2k x_{\mathrm {m}}\right) }\approx r a_1 \exp (-\mathrm
								{i}\,2\phi _0)\,\exp {\Bigl (\mathrm {i}\,2k_{\mathrm {0}}
								a_{\mathrm {s}}\cos (\omega _{\mathrm {s}}t+\varphi _{\mathrm
								{s}})\Bigr )},\nonumber \\ \end{aligned}$$

setting $$m=2k_{\mathrm
								{0}} a_{\mathrm {s}}$$. This can be expressed as

3.23 $$\begin{aligned}
								a_3= & {} r a_1 \exp (-\mathrm {i}\,2\phi _0)\,\Bigl
								(1+\mathrm {i}\,\frac{m}{2}\exp {\Bigl (-\mathrm {i}\,(\omega
								_{\mathrm {s}}t+\varphi _{\mathrm {s}})\Bigr )}+ \mathrm
								{i}\,\frac{m}{2}\exp {\Bigl (\mathrm {i}\,(\omega _{\mathrm
								{s}}t+\varphi _{\mathrm {s}})\Bigr )}\Bigr )\nonumber \\= &
								{} r a_1 \exp (-\mathrm {i}\,2\phi _0)\,\Bigl (1+\frac{m}{2}\exp
								{\Bigl (-\mathrm {i}\,(\omega _{\mathrm {s}}t+\varphi _{\mathrm
								{s}}-\pi /2)\Bigr )}~\Bigr .\nonumber \\&+\,\Bigl
								.\frac{m}{2}\exp {\Bigl (\mathrm {i}\,(\omega _{\mathrm
								{s}}t+\varphi _{\mathrm {s}}+\pi /2)\Bigr )}\Bigr ).
								\end{aligned}$$

### Coupling matrices for beams with multiple frequency components

The coupling between electromagnetic fields at optical components introduced in Sect. 2 referred only to the amplitude and phase of a simplified monochromatic field, ignoring all the other parameters of the electric field of the beam given in Eq. (3). However, this mathematical concept can be extended to include other parameters provided that we can find a way to describe the total electric field as a sum of components, each of which is characterised by a discrete value of the related parameters. In the case of the frequency of the light field, this means we have to describe the field as a sum of monochromatic components. In the previous sections we have shown how this could be done in the special case of an initial monochromatic field that is subject to modulation: if the modulation index is small enough we can limit the number of frequency components that we need to consider. In many cases it is actually sufficient to describe a modulation only by the interaction of the carrier at $$\omega
								_0$$ (the unmodulated field) and two sidebands with a frequency offset of $$\pm \omega
								_m$$ to the carrier. A beam given by the sum of three such components can be described by a complex vector:

3.24 $$\begin{aligned}
								\mathbf {a}= \left( \begin{array}{c} a(\omega _0)\\ a( \omega
								_0-\omega _m)\\ a( \omega _0+\omega _m)\\ \end{array}\right) =
								\left( \begin{array}{c} a_{\omega 0}\\ a_{\omega 1}\\ a_{\omega 2}\\
								\end{array}\right) \end{aligned}$$

with $$\omega _0=\omega
								0$$, $$\omega _0-\omega
								_m=\omega 1$$ and $$\omega _0+\omega
								_m=\omega 2$$. In the case of a phase modulator that applies a modulation of small modulation index *m* to an incoming light field $$\mathbf
								{a}_1$$, we can describe the coupling of the frequency component as follows:

3.25 $$\begin{aligned}
								\begin{array}{l} a_{2,\omega 0}=J_0(m) a_{1,\omega 0} +J_{1}(m)
								a_{1,\omega 1} +J_{-1}(m) a_{1,\omega 2}\\ a_{2,\omega
								1}=J_{0}(m)a_{1,\omega 1}+J_{-1}(m) a_{1,\omega 0} \\ a_{2,\omega
								2}=J_{0}(m)a_{1,\omega 2} + J_1(m) a_{1,\omega 0 },\\ \end{array}
								\end{aligned}$$

which can be written in matrix form:

3.26 $$\begin{aligned}
								\mathbf {a}_2=\left( \begin{array}{c@{\quad }c@{\quad }c} J_0(m)
								&{} J_{1}(m) &{} J_{-1}(m)\\ J_{-1}(m) &{}
								J_0(m) &{} 0 \\ J_{1}(m) &{} 0 &{} J_0(m) \\
								\end{array}\right) \mathbf {a}_1 .
								\end{aligned}$$

And similarly, we can write the complete coupling matrix for the modulator component, for example, as

3.27 $$\begin{aligned}
								\left( \begin{array}{c} a_{2,w0}\\ a_{2,w1}\\ a_{2,w2}\\ a_{4,w0}\\
								a_{4,w1}\\ a_{4,w2}\\ \end{array}\right) \left(
								\begin{array}{c@{\quad }c@{\quad }c@{\quad }c@{\quad }c@{\quad }c}
								J_0(m) &{} J_{1}(m) &{} J_{-1}(m) &{} 0
								&{} 0 &{} 0\\ J_{-1}(m) &{} J_0(m)
								&{} 0 &{} 0 &{} 0 &{} 0\\ J_{1}(m)
								&{} 0 &{} J_0(m) &{} 0 &{} 0
								&{} 0\\ 0 &{} 0 &{} 0 &{} J_0(m)
								&{} J_{1}(m) &{} J_{-1}(m)\\ 0 &{} 0
								&{} 0 &{} J_{-1}(m) &{} J_0(m) &{} 0
								\\ 0 &{} 0 &{} 0 &{} J_{1}(m) &{} 0
								&{} J_0(m)\\ \end{array}\right) \left( \begin{array}{c}
								a_{1,w0}\\ a_{1,w1}\\ a_{1,w2}\\ a_{3,w0}\\ a_{3,w1}\\ a_{3,w2}\\
								\end{array}\right) \end{aligned}$$

**Fig. 22:**
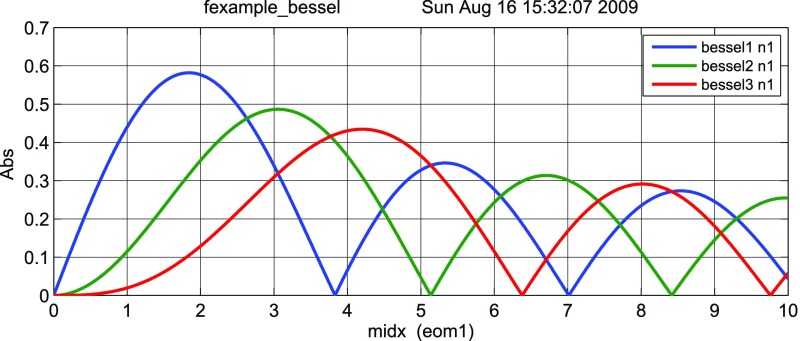
Finesse example: modulation index

### Finesse examples

#### Modulation index

This file demonstrates the use of a modulator. Phase modulation (with up to five higher harmonics is applied to a laser beam and amplitude detectors are used to measure the field at the first three harmonics. Compare this to Fig. 18 as well (Fig. 22).

**Finesse input file for ‘Modulation index’** 
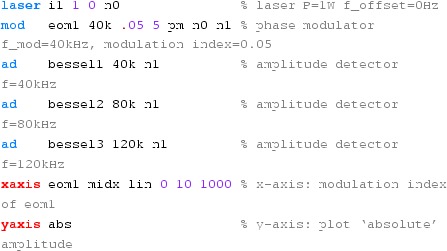


#### Mirror modulation

**Fig. 23:**
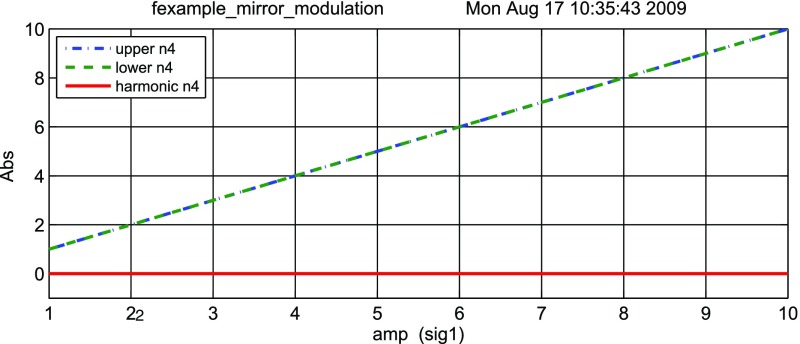
Finesse example: mirror modulation

Finesse offers two different types of modulators: the ‘modulator’ component shown in the example above, and the ‘fsig’ command, which can be used to apply a *signal modulation* to existing optical components. The main difference is that ‘fsig’ is meant to be used for transfer function computations. Consequently Finesse discards all nonlinear terms, which means that the sideband amplitude is proportional to the signal amplitude and harmonics are not created (Fig. 23).

**Finesse input file for ‘Mirror modulation’** 
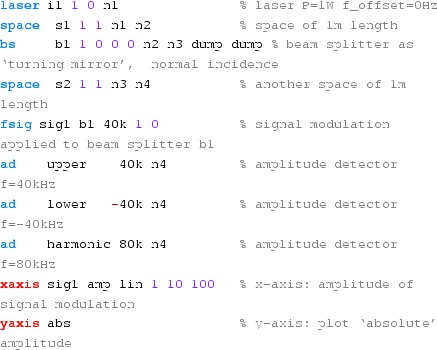


## Optical readout

In previous sections we have dealt with the amplitude of light fields directly and also used the *amplitude detector* in the Finesse examples. This is the advantage of a mathematical analysis versus experimental tests, in which only light intensity or light power can be measured directly. This section gives the mathematical details for modelling photo detectors.

The intensity of a field impinging on a photo detector is given as the magnitude of the Poynting vector, with the Poynting vector given as (Yariv 1989)

4.1 $$\begin{aligned}
							\mathbf {S}=\mathbf {E}\times \mathbf {H}=\frac{1}{\mu _0}\mathbf
							{E}\times \mathbf {B}. \end{aligned}$$

Inserting the electric and magnetic components of a plane wave, we obtain

4.2 $$\begin{aligned}
							|\mathbf {S}|=\frac{1}{\mu _0 c}E^2=c\epsilon _0E^2_0\cos ^2(\omega
							t)=\frac{c\epsilon _0}{2} E_0^2\left( 1+\cos (2\omega t)\right) ,
							\end{aligned}$$

with $$\epsilon
							_0$$ the electric permeability of vacuum and *c* the speed of light.

The response of a photo detector is given by the total flux of effective radiation^5^ during the response time of the detector. For example, in a photodiode a photon will release a charge in the n-p junction. The response time is given by the time it takes for the charge to travel through the detector (and further time may be taken up in the electronic processing of the signal). The size of the photodiode and the applied bias voltage determine the travel time of the charges with typical values of approximately 10 ns. Thus, frequency components faster than perhaps 100 MHz are not resolved by a standard photodiode. For example, a laser beam with a wavelength of $$\lambda
							$$ = 1064 nm has a frequency of $$f=c/\lambda \approx
							282~10^{12}\mathrm {\ Hz}=282\mathrm {\ THz}$$. Thus, the $$2\omega
							$$ component is much too fast for the photo detector; instead, it returns the average power

4.3 $$\begin{aligned}
							|\overline{\mathbf {S}}|=\frac{c\epsilon _0}{2} E_0^2.
							\end{aligned}$$

In complex notation we can write

4.4 $$\begin{aligned}
							|\overline{\mathbf {S}}|=\frac{c\epsilon _0}{2}E E^*.
							\end{aligned}$$

However, for more intuitive results the light fields can be given in converted units, so that the light power can be computed as the square of the light field amplitudes. Unless otherwise noted, throughout this work the unit of light field amplitudes is $$\sqrt{\mathrm
							{watt}}$$. Thus, the notation used in this document to describe the computation of the light power of a laser beam is

4.5 $$\begin{aligned} P=E
							E^*. \end{aligned}$$

**Fig. 24:**
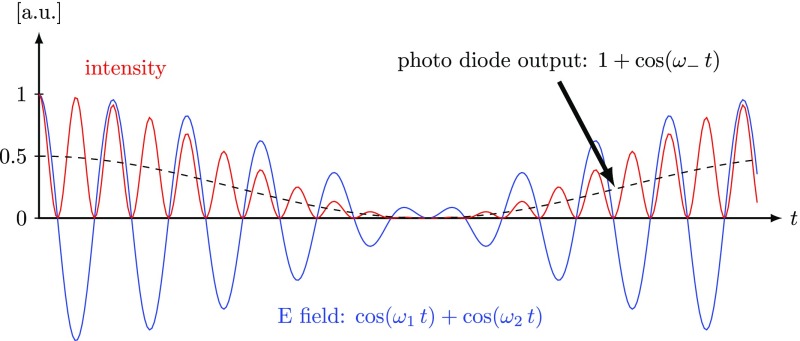
A beam with two frequency components hits the photo diode. Shown in this plot are the field amplitude, the corresponding intensity and the electrical output of the photodiode

### Detection of optical beats

What is usually called an *optical beat* or simply a *beat* is the sinusoidal behaviour of the intensity of two overlapping and coherent fields. For example, if we superpose two fields of slightly different frequency, we obtain

4.6 $$\begin{aligned}
								E= & {} E_0\cos (\omega _1 t)+ E_0 \cos (\omega _2
								t)\nonumber \\ P= & {} E^2=E_0^2\left( \cos ^2(\omega _1 t)
								+ \cos ^2(\omega _2 t) + 2\cos (\omega _{1} t)\cos (\omega _{2}
								t)\right) \nonumber \\= & {} E_0^2\left( \cos ^2(\omega _1
								t) + \cos ^2(\omega _2 t) + \cos (\omega _{+} t) + \cos (\omega _{-}
								t)\right) , \end{aligned}$$

with $$\omega
								_{+}=\omega _{1}+\omega _{2}$$ and $$\omega
								_{-}=\omega _{1}-\omega _{2}$$. In this equation the frequency $$\omega
								_{-}$$ can be very small and can then be detected with the photodiode as illustrated in Fig. 24.

4.7 $$\begin{aligned}
								P_{\mathrm {diode}}=E_0^2\left( 1+\cos (\omega _{-} t)\right)
								\end{aligned}$$

Using the same example photodiode as before: in order to be able to detect an optical beat $$\omega
								_{-}$$ would need to be smaller than 100 MHz. If we take two, sightly detuned Nd:YAG lasers with $$f=282$$ THz, this means that the relative detuning of these lasers must be smaller than $$10^{-7}$$.

In general, for a field with several frequency components, the photodiode signal can be written as

4.8 $$\begin{aligned}
								\begin{array}{rcl} |E|^2&{}=&{}E\cdot E^*=\sum
								\limits _{i=0}^N\sum \limits _{j=0}^N a_ia_j^*~e^{\mathrm
								{i}\,(\omega _i-\omega _j)\,t}.\\ \end{array}
								\end{aligned}$$

For example, if the photodiode signal is filtered with a low-pass filter, such that only the DC part remains, we can compute the resulting signal by looking for all components without frequency dependence. The frequency dependence vanishes when the frequency becomes zero, i.e., in all parts of Eq. (4.8) with $$\omega _i =
								\omega _j$$. The output is a real number, calculated like this:

4.9 $$\begin{aligned}
								x=\sum \limits _i\sum \limits _j a_ia_j^*\quad \mathrm {with}\quad
								\{i,j~|~i,j\in \{0,\ldots ,N\}~\wedge ~\omega _i=\omega _j\}.
								\end{aligned}$$

### Signal demodulation

A typical application of light modulation, is its use in a modulation-demodulation scheme, which applies an electronic demodulation to a photodiode signal. A ‘demodulation’ of a photodiode signal at a user-defined frequency $$\omega
								_{x}$$, performed by an electronic mixer and a low-pass filter, produces a signal, which is proportional to the amplitude of the photo current at DC and at the frequency $$\omega _0\pm
								\omega _x$$. Interestingly, by using two mixers with different phase offsets one can also reconstruct the phase of the signal, or to be precise the phase difference of the light at $$\omega _0 \pm
								\omega _x$$ with respect to the carrier light. This feature can be very powerful for generating interferometer control signals.

Mathematically, the demodulation process can be described by a multiplication of the output with a cosine: $$\cos (\omega
								_x+\varphi _x)$$, where $$\varphi
								_x$$ is the *demodulation phase*. This cosine is also called the ‘local oscillator’. The signal is

4.10 $$\begin{aligned}
								S_0=|E|^2=E\cdot E^*=\sum \limits _{i=0}^N\sum \limits _{j=0}^N
								a_ia_j^*~e^{\mathrm {i}\,(\omega _i-\omega _j)\,t}.
								\end{aligned}$$

Multiplied with the local oscillator it becomes

4.11 $$\begin{aligned}
								S_{1}= & {} S_0\cdot \cos (\omega _xt+\varphi
								_x)=S_0\frac{1}{2}\left( e^{\mathrm {i}\,(\omega _xt+\varphi _x)} +
								e^{-\mathrm {i}\,(\omega _xt+\varphi _x)}\right) \nonumber \\=
								& {} \frac{1}{2}\sum \limits _{i=0}^N\sum \limits _{j=0}^N
								a_ia_j^*~e^{\mathrm {i}\,(\omega _i-\omega _j)\,t}\cdot \left(
								e^{\mathrm {i}\,(\omega _xt+\varphi _x)} + e^{-\mathrm {i}\,(\omega
								_xt+\varphi _x)}\right) . \end{aligned}$$

With $$A_{ij}=a_ia_j^*$$ and $$e^{\mathrm
								{i}\,\omega _{ij}\,t}=e^{\mathrm {i}\,(\omega _i-\omega
								_j)\,t}$$ we can write

4.12 $$\begin{aligned}
								S_{1}\!=\!\frac{1}{2}\left( \sum \limits _{i=0}^NA_{ii}+\sum \limits
								_{i=0}^N \sum \limits _{j=i+1}^N (A_{ij}~e^{\mathrm {i}\,\omega
								_{ij}\,t}\!+\!A_{ij}^*~e^{-\mathrm {i}\,\omega _{ij}\,t})\right)
								\cdot \left( e^{\mathrm {i}\,(\omega _xt+\varphi _x)}+e^{-\mathrm
								{i}\,(\omega _xt+\varphi _x)}\right) .
								\end{aligned}$$

When looking for the DC components of $S_1$ we get the following (Freise 2003):

4.13 $$\begin{aligned}
								S_{\mathrm {1,DC}}= & {} \sum \limits _{ij}
								\frac{1}{2}(A_{ij}~e^{-\mathrm {i}\,\varphi _x}+A_{ij}^*~e^{\mathrm
								{i}\,\varphi _x})\quad \mathrm {with}\quad \{i,j~|~i,j\in \{0,\ldots
								,N\}~\wedge ~\omega _{ij}=\omega _x\}\nonumber \\= & {} \sum
								\limits _{ij}{\mathfrak {R}}\left\{ A_{ij}~e^{-\mathrm {i}\,\varphi
								_x} \right\} . \end{aligned}$$

This would be the output of a mixer and a subsequent low-pass filter. The results for $$\varphi
								_x=0$$ and $$\varphi _x=\pi
								/2$$ are called *in-phase* and *in-quadrature*, respectively (or also *first* and *second quadrature*). They are given by

4.14 $$\begin{aligned}
								S_{\mathrm {1,DC,phase}}= & {} \sum \limits _{ij}{\mathfrak
								{R}}\left\{ A_{ij} \right\} ,\nonumber \\ S_{\mathrm {1,DC,quad}}=
								& {} \sum \limits _{ij}{\mathfrak {I}}\left\{
								{A_{ij}}\right\} . \end{aligned}$$

If only one mixer is used, the output is always real and is determined by the demodulation phase. However, with two mixers generating the in-phase and in-quadrature signals, it is possible to construct a complex number representing the signal amplitude and phase:

4.15 $$\begin{aligned}
								z=\sum \limits _{ij}a_{i}a^*_{j}\quad \mathrm {with}\quad
								\{i,j~|~i,j\in \{0,\ldots ,N\}~\wedge ~\omega _{ij}=\omega _x\}.
								\end{aligned}$$

Often several sequential demodulations are applied in order to measure very specific phase information. For example, a double demodulation can be described as two sequential multiplications of the signal with two local oscillators and taking the DC component of the result. First looking at the whole signal, we can write:

4.16 $$\begin{aligned}
								S_{2}=S_0\cdot \cos (\omega _x t +\varphi _x)\cos (\omega _y t
								+\varphi _y). \end{aligned}$$

This can be written as

4.17 $$\begin{aligned}
								S_{2}= & {} S_0\frac{1}{2}( \cos (\omega _y t +\omega _x t
								+\varphi _y+\varphi _x)+\cos (\omega _y t -\omega _x t +\varphi
								_y-\varphi _x))\nonumber \\= & {} S_0\frac{1}{2}( \cos
								(\omega _+ t +\varphi _+)+\cos (\omega _- t +\varphi _-)),
								\end{aligned}$$

and thus reduced to two single demodulations. Since we now only care for the DC component we can use the expression from above [Eq. (4.15)]. These two demodulations give two complex numbers:

4.18 $$\begin{aligned}
								z1= & {} \sum \limits _{ij}A_{ij}\quad \mathrm {with}\quad
								\{i,j~|~i,j\in \{0,\ldots ,N\}~\wedge ~\omega _i-\omega _j=\omega
								_+\},\nonumber \\ z2= & {} \sum \limits _{ij}A_{kl}\quad
								\mathrm {with}\quad \{k,l~|~k,l\in \{0,\ldots ,N\}~\wedge ~\omega
								_k-\omega _l=\omega _-\}. \end{aligned}$$

The demodulation phases are applied as follows to get a real output (two sequential mixers)

4.19 $$\begin{aligned}
								x={\mathfrak {R}}\left\{ (z_1~e^{-\mathrm {i}\,\varphi
								_x}+z_2~e^{\mathrm {i}\,\varphi _x})~e^{-\mathrm {i}\,\varphi _y}
								\right\} . \end{aligned}$$

In a typical setup, a user-defined demodulation phase for the first frequency (here $$\varphi
								_x$$) is given. If two mixers are used for the second demodulation, we can reconstruct the complex number

4.20 $$\begin{aligned}
								z=z_1~e^{-\mathrm {i}\,\varphi _x}+z_2~e^{\mathrm {i}\,\varphi _x}.
								\end{aligned}$$

More demodulations can also be reduced to single demodulations as above.

**Fig. 25:**
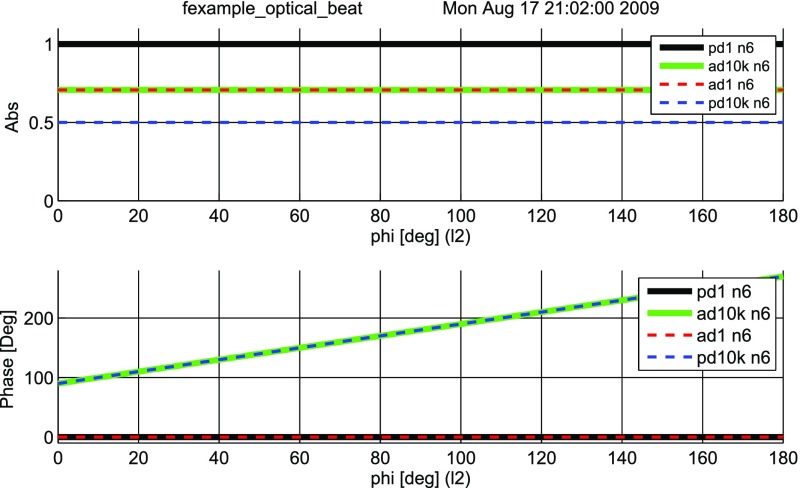
Finesse example: optical beat

### Finesse examples

#### Optical beat

In this example two laser beams are superimposed at a 50:50 beam splitter. The beams have a slightly different frequency: the second beam has a 10 kHz offset with respect to the first (and to the default laser frequency). The plot illustrates the output of four different detectors in one of the beam splitter output ports, while the phase of the second beam is tuned from 0° to 180°. The photodiode ‘pd1’ shows the total power remaining constant at a value of 1. The amplitude detectors ‘ad1’ and ‘ad10k’ detect the laser light at 0 Hz (default frequency) and 10 kHz respectively. Both show a constant absolute of $\sqrt{1/2}$ and the detector ‘ad10k’ tracks the tuning of the phase of the second laser beam. Finally, the detector ‘pd10k’ resembles a photodiode with demodulation at 10 kHz. In fact, this represents a photodiode and two mixers used to reconstruct a complex number as shown in Eq. (4.15). One can see that the phase of the resulting electronic signal also directly follows the phase difference between the two laser beams (Fig. 25).

**Finesse input file for ‘Optical beat’** 
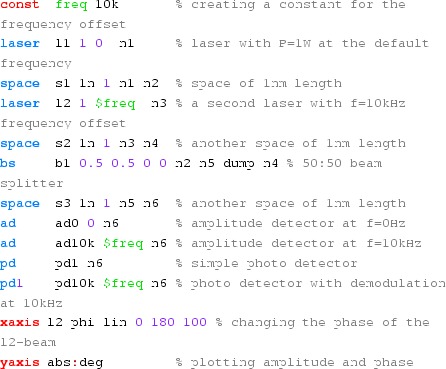


## Basic interferometers

The large interferometric gravitational-wave detectors currently in operation are based on two fundamental interferometer topologies: the *Fabry–Perot* interferometer and the *Michelson* interferometer. The main instrument is very similar to the original interferometer concept used in the famous experiment by Michelson and Morley (1887). The main difference is that modern instruments use laser light to illuminate the interferometer to achieve much higher accuracy. Already an early prototype in 1971 has thus achieved a sensitivity a million times better than Michelson’s original instrument (Moss et al. 1971). In addition, the Michelson interferometer used in current gravitational-wave detectors has been enhanced by resonant cavities, which in turn have been derived from the original idea for a spectroscopy standard published by Fabry and Perot (1899). The following section will describe the fundamental properties of the Fabry–Perot interferometer and the Michelson interferometer. A thorough understanding of these basic instruments is essential for the study of the high-precision interferometers used for gravitational-wave detection.

### The two-mirror cavity: a Fabry–Perot interferometer

**Fig. 26:**
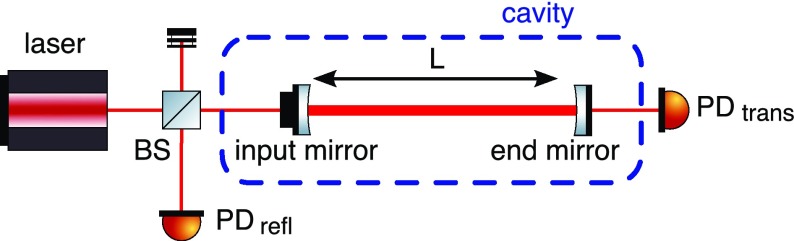
Typical optical layout of a two-mirror cavity, also called a Fabry–Perot interferometer. Two mirrors form the Fabry–Perot interferometer, a laser beam is injected through one of the mirrors and the reflected and transmitted light can be detected by photo detectors

We have computed the field amplitudes in a linear two-mirror cavity, also called a *Fabry–Perot* interferometer, in Sect. 2.2. In order to understand the features of this optical instrument it is interesting to have a closer look at the power circulating in the cavity. A typical optical layout is shown in Fig. 26; two parallel mirrors form the Fabry–Perot cavity. A laser beam is injected through the first mirror (at normal incidence).

The behaviour of the (ideal) cavity is determined by the length of the cavity *L*, the wavelength of the laser $$\lambda
								$$ and the reflectivity and transmittance of the mirrors. Using the mathematical description introduced in Sect. 2.2 and assuming an input power of $$|a_0|^2=1$$, we obtain the following equation for the circulating power:

5.1 $$\begin{aligned}
								P_1=|a_1|^2 =\frac{T_1}{1+R_1R_2-2r_1r_2\cos \left( 2 k L\right) },
								\end{aligned}$$

with $$k=2\pi /\lambda
								$$, *P*, $$T=t^2$$ and $$R=r^2$$, as defined in Sect. 1.3. Similarly we could compute the transmission of the optical system as the input–output ratio of the field amplitudes. For example, with $$a_0$$ the field injected into the cavity and $$a_2$$ the field transmitted by the cavity,

5.2 $$\begin{aligned}
								\frac{a_2}{a_0}=\frac{-t_1 t_2 \exp (-\mathrm {i}\,k L)}{1-r_1 r_2
								\exp (- \mathrm {i}\,2 k L)}
								\end{aligned}$$

is the frequency-dependent transfer function of the cavity in transmission (the frequency dependence is hidden inside the $$k=2\pi
								f/c$$).

**Fig. 27:**
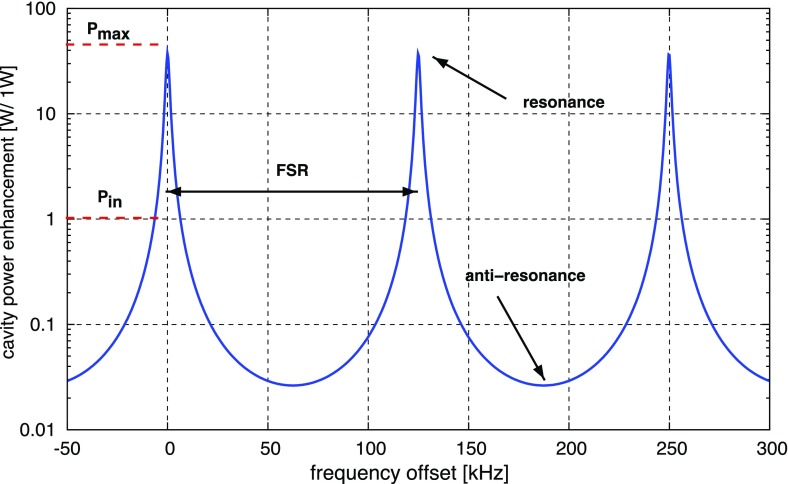
Power enhancement in a two-mirror cavity as a function of the laser-light frequency. The *peaks* marks the resonances of the cavity, i.e., modes of operation in which the injected light is resonantly enhanced. The frequency distance between two peaks is called *free-spectral range* (FSR)

Figure 27 shows a plot of the circulating light power $$P_1$$ over the laser frequency. The maximum power is reached when the cosine function in the denominator becomes equal to one, i.e., at $$k L = N \pi
								$$ with *N* an integer. This occurs when the round-trip length is an integer multiple of the wavelength of the injected light: $$2L = N 2\pi /k=N
								\lambda $$. This is called the cavity *resonance*. The lowest power values are reached at *anti-resonance* when $$k L = (N
								+1/2)\pi $$. We can also rewrite

5.3 $$\begin{aligned}
								2 k L=\omega \frac{2L}{c} = 2 \pi f \frac{2 L}{c} = \frac{2 \pi
								f}{\mathrm {FSR}}, \end{aligned}$$

with FSR being the *free-spectral range* of the cavity as shown in Fig. 27. Thus, it becomes clear that resonance is reached for laser frequencies

5.4 $$\begin{aligned}
								f_{r}= N \cdot \mathrm {FSR},
								\end{aligned}$$

where *N* is an integer.

Another characteristic parameter of a cavity is its linewidth, usually given as its *full width at half maximum* (FWHM) or its *pole frequency*, $f_p$. In order to compute the linewidth we have to ask at which frequency the circulating power becomes half the maximum:

5.5 $$\begin{aligned}
								\begin{array}{l} |a_1(f_p)|^2 \mathop {=}\limits ^{!}\frac{1}{2}
								|a_{1,\mathrm {max}}|^2.\\ \end{array}
								\end{aligned}$$

This results in the following expression for the full linewidth:

5.6 $$\begin{aligned}
								\mathrm {FWHM}=2f_p=\frac{2 \mathrm {FSR}}{\pi }\arcsin \left(
								\frac{1-r_1r_2}{2\sqrt{r_1r_2}}\right) .
								\end{aligned}$$

The ratio of the linewidth to the free spectral range is called the *finesse* of a cavity:

5.7 $$\begin{aligned}
								F=\frac{\mathrm {FSR}}{\mathrm {FWHM}}=\frac{\pi }{2\arcsin \left(
								\frac{1-r_1r_2}{2\sqrt{r_1r_2}}\right) }.
								\end{aligned}$$

In the case of high finesse, i.e., when $$r_1$$ and $$r_2$$ are close to 1, we can use the fact that the argument of the $$\arcsin
								$$ function is small and make the approximation

5.8 $$\begin{aligned}
								F\approx \frac{\pi \sqrt{r_1r_2}}{1-r_1r_2}\approx \frac{\pi
								}{1-r_1r_2}. \end{aligned}$$

The behaviour of a two mirror cavity depends on the length of the cavity (with respect to the frequency of the laser) and on the reflectivities of the mirrors. Regarding the mirror parameters, one distinguishes three cases^6^:

- when $T_1<T_2$ the cavity is *undercoupled*
- when $T_1=T_2$ the cavity is *impedance matched*
- when $T_1>T_2$ the cavity is *overcoupled*

**Fig. 28:**
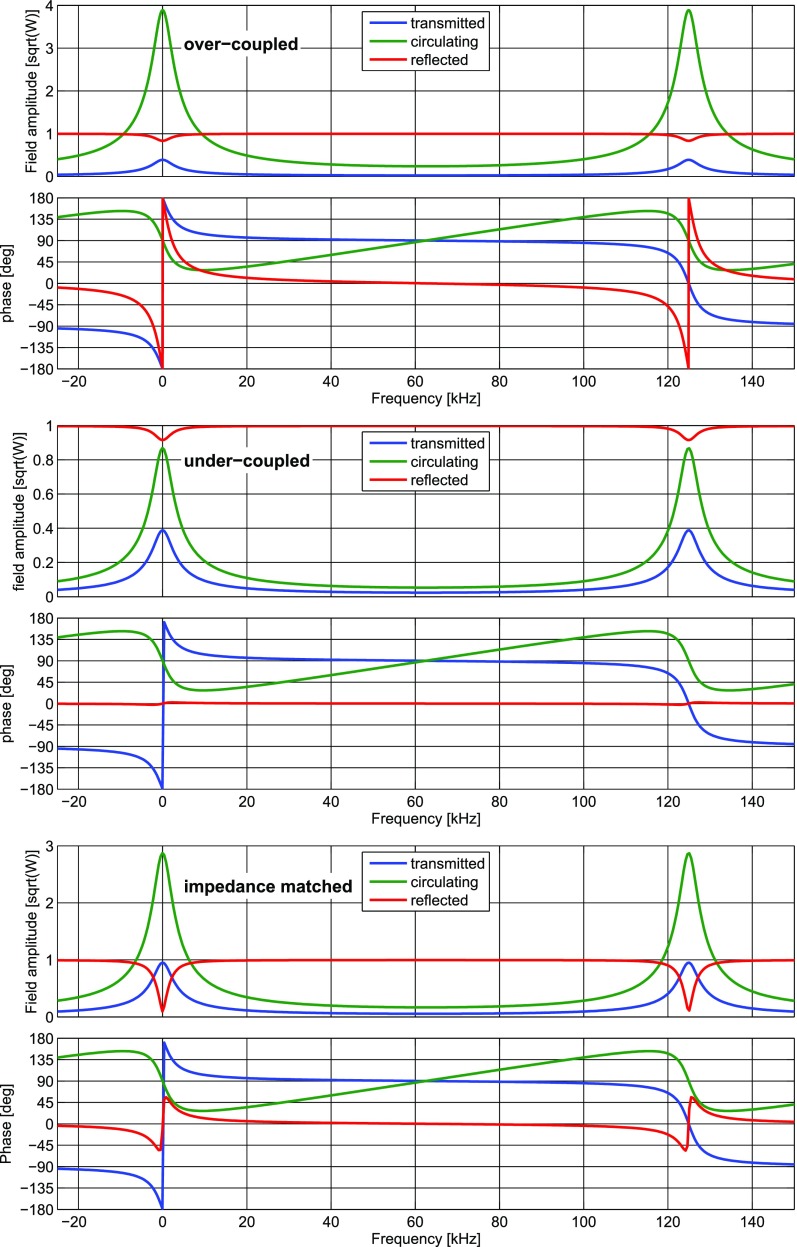
This figure compares the fields reflected by, transmitted by and circulating in a Fabry–Perot cavity for the three different cases: over-coupled, under-coupled and impedance matched cavity (in all cases $T_1+T_2=0.2$ and the round-trip loss is 1 %). The traces show the phase and amplitude of the electric field as a function of laser frequency detuning

**Fig. 29:**
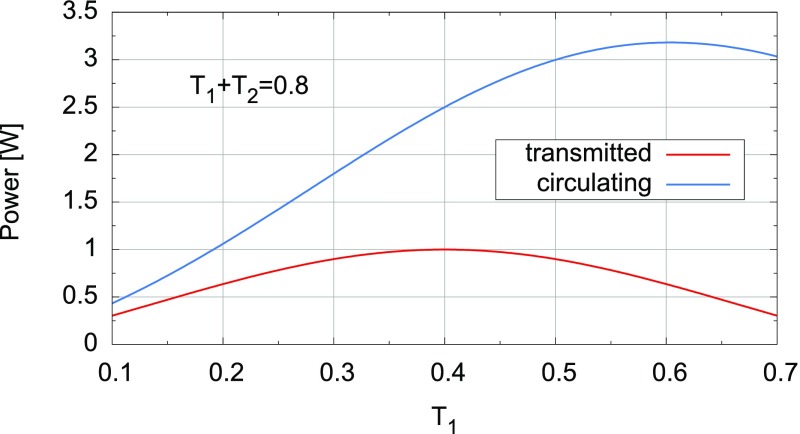
Power transmitted and circulating in a two mirror cavity with input power 1 W. The mirror transmissions are set such that $T_1+T_2=0.8$ and the reflectivities of both mirrors are set as $R=1-T$. The cavity is undercoupled for $T_1<0.4$, impedance matched at $T_1=T_2=0.4$, and overcoupled for $T_1>0.4$. The transmission is maximised in the impedance-matched case and falls similarly for over or undercoupled settings. However, the circulating power (and any resonance performance of the cavity) is much larger in the overcoupled case

The differences between these three cases can seem subtle mathematically but have a strong impact on the application of cavities in laser systems. One of the main differences is the phase evolution of the light fields, as shown in Fig. 28. The circulating power shows that the resonance effect is better used in over-coupled cavities; this is illustrated in Fig. 29, which shows the transmitted and circulating power for the three different cases. Only in the impedance-matched case can the cavity transmit (on resonance) *all* the incident power. Given the same total transmission $T_1+T_2$, the overcoupled case allows for the largest circulating power and thus a stronger ‘resonance effect’ of the cavity, which is useful, for example, when the cavity is used as a mode filter. Hence, most commonly used cavities are impedance matched or overcoupled.

### Michelson interferometer

**Fig. 30:**
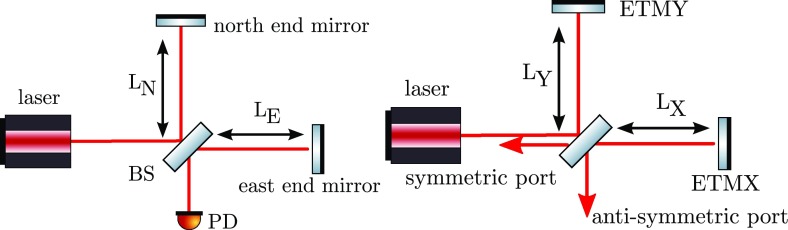
Optical layout and two common naming convention for a Michelson interferometer: a laser beam is split into two and sent along two perpendicular interferometer arms. We will sometimes label the directions in a Michelson interferometer as North, East, West and South, as shown in the *left plot*. The end mirrors or end test masses (ETMs), reflect the beams towards the beam splitter, where they recombine. The South and West ports of the beam splitter are possible output port; however in many cases only the South port is used. The plot on the *right* shows an alternative naming scheme commonly used, in which the two arms are labelled X and Y, the output towards the laser is called the *symmetric port* and the other output is referred to as the *anti-symmetric port*

We came across the Michelson interferometer in Sect. 2.4 when we discussed the phase relation at a beam splitter. The typical optical layout of the Michelson interferometer is shown again in Fig. 30, a laser beam is split by a beam splitter and sent along two perpendicular *interferometer arms*. The four directions seen from the beam splitter are often labelled North, East, West and South. Another common naming scheme, also shown in Fig. 30 refers to the interferometer arms as X and Y; the two outputs are labelled as the symmetric port (towards the laser input) and anti-symmetric port respectively. Both conventions are common in the literature and we will make use of both in this article.

The ends of the interferometer arms (North and East or Y and X) are marked by highly reflective *end mirrors*, sometimes called *end test masses* (ETM), The laser beams are reflected by the end mirrors and then recombined at the central beam splitter. Generally, the Michelson interferometer has two outputs, namely the so far unused beam splitter port (*South port* or *anti-symmetric port*) and the input port (*West port * or *symmetric port*). Both output ports can be used to obtain interferometer signals; however most setups are designed such that the main signals are detected in the South port.^7^

The Michelson interferometer output signal is determined by the laser wavelength $$\lambda
								$$, the reflectivity and transmittance of the beam splitter and the end mirrors, and the relative length of the interferometer arms. In many cases the end mirrors are highly reflective and the beam splitter is ideally a 50:50 beam splitter. In this case, we can compute the output for a monochromatic field as shown in Sect. 2.4. Using Eq. (2.17) we can write the field in the South port as

5.9 $$\begin{aligned}
								E_S=E_0~\frac{\mathrm {i}\,}{2}\left( e^{\mathrm {i}\,2 k
								L_N}~+~e^{\mathrm {i}\,2 k L_E}\right) .
								\end{aligned}$$

We define the common and differential arm lengths as

5.10 $$\begin{aligned}
								\bar{L}= & {} \frac{L_N+L_E}{2}\nonumber \\ \varDelta L=
								& {} L_N-L_E, \end{aligned}$$

which yield $$2L_N=2\bar{L}+\varDelta
								L$$ and $$2L_E=2\bar{L}-\varDelta
								L$$. Thus, we can further simplify to get

5.11 $$\begin{aligned}
								E_S=E_0~\frac{\mathrm {i}\,}{2} e^{\mathrm {i}\,2 k \bar{L}} \left(
								e^{\mathrm {i}\,k \varDelta L}~+~e^{-\mathrm {i}\,k \varDelta
								L}\right) =E_0~\mathrm {i}\,e^{\mathrm {i}\,2 k \bar{L}} \cos (k
								\varDelta L). \end{aligned}$$

The photo detector then produces a signal proportional to

5.12 $$\begin{aligned}
								S=E_SE^*_S=P_0 \cos ^2(k \varDelta L)=P_0 \cos ^2(2\pi \varDelta
								L/\lambda ). \end{aligned}$$

This signal is depicted in Fig. 31; it shows that the power in the South port changes between zero and the input power with a period of $$\varDelta
								L/\lambda =0.5$$. The tuning at which the output power drops to zero is called the *dark fringe*. Current interferometric gravitational-wave detectors operate their Michelson interferometer at or near the dark fringe.

**Fig. 31:**
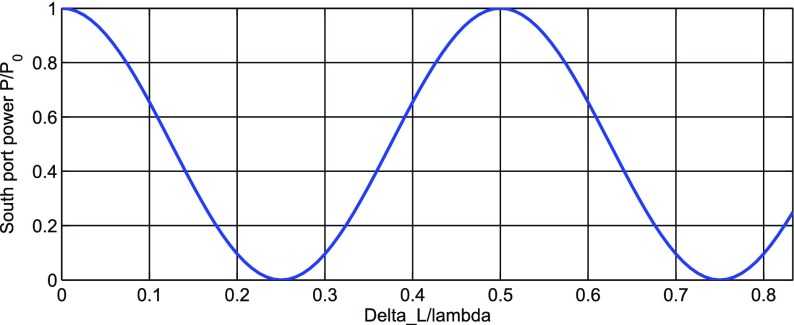
Power in the South port of a symmetric Michelson interferometer as a function of the arm length difference $$\varDelta L$$. When the interferometer is set to $$\varDelta L/\lambda
											=0.25$$ the input light is not transmitted into the South port: this condition is called the *dark fringe*

The above seems to indicate that the macroscopic arm-length difference plays no role in the Michelson output signal. However, this is only correct for a monochromatic laser beam with infinite coherence length. In real interferometers care must be taken that the arm-length difference is well below the coherence length of the light source. In gravitational-wave detectors the macroscopic arm-length difference is an important design feature; it is kept very small in order to reduce coupling of laser noise into the output but needs to retain a finite size to allow the transfer of phase modulation sidebands from the input to the output port; this is illustrated in the Finesse example below and will be covered in detail in Sect. 8.11.

### Michelson interferometer and the sideband picture

In the context of gravitational wave detection the Michelson interferometer is used for measuring a very small differential change in the length of one arm versus the other. The very small amplitude of gravitational waves, or the equivalent small differential change of the arm lengths, requires additional optical techniques to increase the sensitivity of the interferometer. In this section we briefly introduce the interferometer configurations and review their effect on the detector sensitivity.

The Michelson interferometer can achieve its best sensitivity when operated in a quasi stationary mode, i.e., when the positions of mirrors and beamsplitters are carefully controlled so that the key parameters, for example the light power inside the interferometer and at the output ports, are nearly constant. We call such an interferometer state, described by a unique set of the key parameters, an *operating point* of the interferometer (see Sect. 8 for a discussion of the control systems involved to reach and maintain an operating point). For an interferometer in a steady state it is possible to describe and analyse the behaviour using a *steady state model*, describing the light field coupling in the frequency domain and making use of the previously introduced concept of sidebands, see Sect. 3.1.

**Fig. 32:**
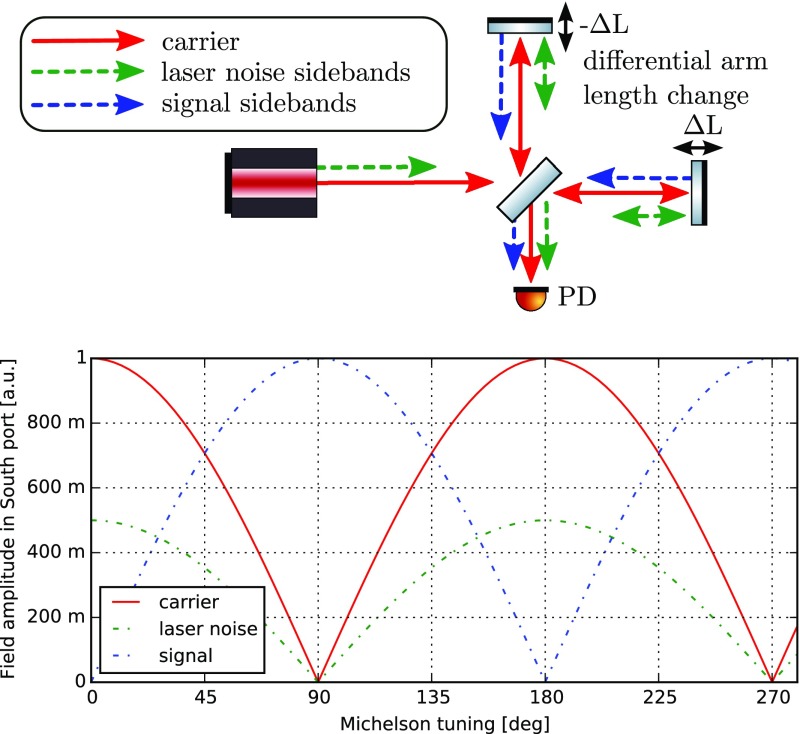
A Michelson interferometer shown with three types of light field: the ‘carrier’, representing the undistorted laser input field, ‘laser phase noise sidebands’, which enter the interferometer with the carrier, and ‘signal sidebands’, which are phase modulation sidebands caused by differential arm length motion. All three fields leave the interferometer through both output ports (here only the detector in the South port is shown). The *graph* shows the amplitude of the three light fields in the South port as a function of the Michelson tuning (differential arm length change). At 0$^{\circ }$ the Michelson is on a *bright fringe* and at 90$^{\circ }$ on a *dark fringe*

Consider a Michelson interferometer which is to be used to measure a differential arm length change. As an example for a signal to noise comparison we consider the phase noise of the injected laser light. For this example the noise can be represented by a sinusoidal modulation with a small amplitude at a single frequency, say 100 Hz. Therefore we can describe the phase noise of the laser by a pair of sidebands superimposed on the main carrier light field entering the Michelson interferometer. Equally the change of an interferometer arm represents a phase modulation of the light reflected back from the end mirrors and the generated optical signal can be represented by a pair of phase modulation sidebands, see Sect. 5.5.

In order to get an estimation of the signal to noise ratio we can trace the individual sidebands through the interferometer and compute their amplitude in the output port. Figure 32 shows the setup of a basic Michelson interferometer, indicating the insertion of the noise and signal sidebands. It also provides a plot of the sideband amplitude in the South output port as a function of the differential arm length of the Michelson interferometer. We can see that a tuning of 90$$^{\circ
								}$$ corresponds to the dark fringe, the state of the interferometer in which the injected light (the carrier and laser noise) is reflected back towards the laser and is not transmitted into the South port. The plot reveals two advantages of the dark fringe as an operating point: first of all the transmission of the signal sidebands to the photo detector is maximised while the laser phase noise is minimised. More generally at the dark fringe, all common mode effects, such as laser noise, or common length changes of the arms, produce a minimal optical signal at the output port, whereas differential effects in the arms are maximised. Furthermore at the dark fringe the least amount of carrier light is transmitted to the photo detector. This is an advantage because it is technically often easier to make an accurate light power measurement when the total detected power is low.

It should be noted that with the interferometer on the dark fringe, only the signal sidebands would leave the interferometer. Typically these alone cannot create a strong signal during detection. In the case of gravitational wave detection these sidebands are many orders of magnitude smaller than the amplitude of the carrier. We require a beat between the signal sidebands and another field, a so-called *local oscillator*, to generate a strong electronic signal proportional to the amplitude of the signal sidebands. The local oscillator can be created in different ways, the most common are:

- Apply an RF modulation to the laser beam, either before injecting it into the interferometer or inside the interferometer. A small macroscopic length asymmetry between the two arms (Schnupp asymmetry, see Sect. 8.13) allows a significant amount of the RF sidebands to reach the South port when the interferometer is operating on the dark fringe for the carrier. The RF sideband fields can be used as a local oscillator.
- Set the Michelson such that it is close to, but not exactly on, the dark fringe. The carrier leaking into the South port can thus be used as a local oscillator. This scheme preserves the advantages of the dark fringe but relies on very good power stability of the carrier light.
- Superimpose an auxiliary beam onto the output before the photodetector. For example, a pick-off beam from the main laser can be used for this. The main disadvantage of this concept is that it requires a very stable auxiliary beam (in phase as well as position) thus creating new control problems.

### Michelson interferometer signal readout with DC offset, or RF modulation

As discussed in Sect. 4.1, one method for providing a local oscillator is to use a small microscopic DC offset to tune the Michelson interferometer slightly away from the dark fringe. This allows a small amount of carrier to leak through to the output port to beat with the signal sidebands. The differential arm length difference required is

5.13 $$\begin{aligned}
								\varDelta L = \frac{\pi }{2 k_0} + \delta _{\mathrm {off}},
								\end{aligned}$$

where $$k_0 = \omega
								_0/c$$ is the wavenumber of the carrier field and the DC offset is $$\delta _{\mathrm
								{off}} \ll 1$$. The field at the output port of a Michelson (as shown in Fig. 11) for a single carrier field and one pair of signal sidebands is:

5.14 $$\begin{aligned}
								E_{6}= & {} itr E_0 e^{-i2k_0\bar{L}}\left( 2\cos (k_0
								\varDelta L) + s^+ + s^-\right) e^{\mathrm {i}\,\omega _0
								t},\nonumber \\= & {} itr E_0 e^{-i2k_0\bar{L}}\left( 2\cos
								\left( \frac{\pi }{2} + k_0\delta _{\mathrm {off}}\right) + s^+ +
								s^-\right) e^{\mathrm {i}\,\omega _0 t}, \nonumber \\= & {}
								itr E_0 e^{-i2k_0\bar{L}}\left( 2\sin \left( k_0\delta _{\mathrm
								{off}}\right) + s^+ + s^-\right) e^{\mathrm {i}\,\omega _0 t},
								\end{aligned}$$

where $$s^\pm
								$$ are the complex amplitudes (magnitude and phase) of the upper and lower sidebands that reach the output port, for example, sidebands generated by a gravitational wave signal or via the modulation of a mirror position. The power in this field as measured by a photodiode will then contain the beats between the carrier and both sidebands. As the magnitude of any signal sideband is assumed to be very small, $$|s^\pm | \ll
								1$$, we only need to consider terms linear in $$s^\pm
								$$. The DC power and terms linear in the $$s^\pm
								$$ are then given by:

5.15 $$\begin{aligned}
								E_{6}E^*_{6}= & {} TR |E_0|^2 \left( 4\sin ^2\left(
								k_0\delta _{\mathrm {off}}\right) + 2\sin \left( k_0\delta _{\mathrm
								{off}}\right) (s^+ + s^-) + O(s^2) \right) . \qquad
								\end{aligned}$$

As expected the signal sideband terms are not visible in the power if $$\sin (k_0\delta
								_{\mathrm {off}}) = 0$$, because, if we operate purely at the dark fringe for the carrier field, no local oscillator is present to beat with the signal. The signal amplitude and phase can then be read out by demodulating the photocurrent at the signal frequency. In practice the choice of $$\delta _{\mathrm
								{off}}$$ depends on a number of technical issues, in particular the laser power in the main output port and the transfer of common mode noise into the output.

Another option for providing a local oscillator is by phase modulating the input laser light, which is typically done at radio-frequencies (RF). This method of readout is also referred to as a *heterodyne readout scheme*. When the Michelson interferometer is set up with a small, macroscopic arm length difference (Schnupp asymmetry) the RF sidebands will have a different interference condition at the beam splitter compared to the carrier, and the inteferometer can be setup so that the RF sidebands are present at the output port, to be used as a local oscillator, whilst the carrier field is at a dark fringe.

Consider a phase modulated beam with modulation index *b* and modulation frequency $$\omega
								_b$$, the input field will be:

5.16 $$\begin{aligned}
								E_0 = E'_{0}e^{\mathrm {i}\,\omega _0 t}(1 + \mathrm {i}\,b
								(e^{\mathrm {i}\,\omega _{b}t} + e^{-\mathrm {i}\,\omega _{b}t})).
								\end{aligned}$$

The propagation of these three input fields to the output port can be treated separately and is similar to Eq. (5.14), except that we must keep track of their different frequencies: $$k_0 = \omega
								_0/c$$ and $$k_{b} = \omega
								_b/c$$ for the upper and lower RF sidebands. Ignoring the signal sidebands the fields present at the output port are

5.17 $$\begin{aligned}
								E_6= & {} E'_6(\omega _0) + E'_6(\omega _0+\omega _b) +
								E'_6(\omega _0-\omega _b) \nonumber \\ E'_6(\omega _0)= & {}
								\mathrm {i}\,2 rt E'_0 \cos \left( k_0 \varDelta L \right) \nonumber
								\\ E'_6(\omega _0\pm \omega _b)= & {} \mathrm {i}\,2 b
								E'_{0}e^{-\mathrm {i}\,2(k_0 \pm k_b) \bar{L}} \cos \left( (k_0 \pm
								k_b)\varDelta L\right) e^{\mathrm {i}\,(\omega _0\pm \omega _{b})t}
								\end{aligned}$$

For using an RF readout scheme we want to set the Michelson to be on the dark fringe for $$E'_6(\omega _0)
								= 0$$. This is done by using a differential arm length difference of $$\varDelta L =
								(2N+1)\frac{\pi }{2 k_0}$$ so that $$\cos \left( k_0
								\varDelta L \right) =0$$, where *N* is any integer. The condition for the RF sidebands is now:

5.18 $$\begin{aligned}
								\cos \left( (k_0 \pm k_b)\varDelta L\right)= & {} \cos
								\left( (k_0 \pm k_b)(2N+1)\frac{\pi }{2 k_0}\right) \nonumber \\=
								& {} \cos \left( \frac{\pi }{2} + N\pi \pm (2N+1)\frac{\pi
								k_b}{2 k_0}\right) \nonumber \\= & {} \sin \left( N\pi \pm
								(2N+1)\frac{\pi k_b}{2 k_0}\right) \nonumber \\= & {} \pm
								(-1)^N\sin \left( k_b \varDelta L_N\right)
								\end{aligned}$$

5.19 $$\begin{aligned}
								\varDelta L_N\equiv & {} (2N+1)\frac{\lambda _0}{4},
								\end{aligned}$$

where $$\lambda
								_0$$ is the wavelength of the carrier light field. Thus the $$\sin \left( k_b
								\varDelta L_N\right) $$ term now determines the amplitude of the RF sidebands that will be present at the output port, where *N* is our free variable to choose. Although $$\varDelta
								L_0$$ is a microscopic distance the actual differential arm length difference required to allow a reasonable amount of sidebands through requires a large choice of *N* as $$k_b \varDelta
								L_N \ll 1$$ for radio frequency modulations. For example, the GEO 600 detector, which uses such an RF modulation scheme, operates with $$\varDelta L =
								13.5$$ cm (Lück et al. 2010). The final step of including the signal sidebands is not elaborated on here but can be included with some careful algebra, remembering that there will be signal sidebands created around the carrier and both RF sidebands that could be present at the output port.

See Sect. 8.13 for an more detailed comparison of the DC and RF techniques to produce control signals and Sect. 8.16 for detailed arguments for the advantages and disadvantages of both techniques.

### Response of the Michelson interferometer to a gravitational waves signal

In this section we derive how the sideband picture can be used to describe how the length modulation caused by a gravitational wave affects a laser beam travelling through space. This method can then be applied to any interferometer setup, for example to compute how the signal readout of a Michelson interferometer when using a DC offset. Modulating a space of proper length *L* will induce a phase modulation to any laser beam travelling along it. The phase such a beam accumulates along a path modulated by a gravitational wave signal *h*(*t*) is (Mizuno 1995)

5.20 $$\begin{aligned}
								\varphi =-k_0 L \mp \frac{\omega _0}{2}\int _{t-L/c}^{t} h(t) = -k_0
								L\mp \delta \varphi , \end{aligned}$$

with $$k_{c} = \omega
								_{0}/c$$ being the wavenumber of the light field and $$\delta \varphi
								$$ being the additional phase accumulated due to the modulation of the path. For our analysis here we can assume the gravitational wave signal is a simple sinusoidal function

5.21 $$\begin{aligned}
								h(t)=h_0\cos \left( \omega _{gw} t + \varphi _{gw}\right) ,
								\end{aligned}$$

where $$\omega
								_{gw}$$ and $$\varphi
								_{gw}$$ are the frequency and phase of the gravitational wave. The phase accumulated from propagating along the space is then^8^

5.22 $$\begin{aligned}
								\delta \varphi = \frac{\omega _0 h_0}{\omega _{gw}}\cos \left(
								\omega _{gw} t +\varphi _{gw} -\omega _{gw}\frac{L}{2c}\right) \sin
								\left( \omega _{gw}\frac{L}{2c}\right) .
								\end{aligned}$$

Thus an oscillating, time dependent phase is present in the light fields travelling along the space. Section 3.2 describes how such a modulation generates sideband fields; the respective modulation index and phase are

5.23 $$\begin{aligned}
								m= & {} -\frac{\omega _0 h_0}{\omega _{gw}} \sin {\left(
								\frac{k_{gw} L}{2}\right) },
								\end{aligned}$$

5.24 $$\begin{aligned}
								\varphi= & {} - \frac{k_{gw} L}{2} + \varphi _{gw},
								\end{aligned}$$

with $$k_{gw} = \omega
								_{gw}/c$$ being the wavenumber for the gravitational wave signal sidebands. Using Eq. (3.10) the unscaled amplitude and phase of the upper, $$\alpha
								^+_{gw}$$, and lower, $$\alpha
								^-_{gw}$$, sidebands generated by a gravitational wave are then

5.25 $$\begin{aligned}
								A_{gw}= & {} - \frac{w_0 h_0}{2\omega _{gw}} \sin {\left(
								\frac{k_{gw} L}{2}\right) },
								\end{aligned}$$

5.26 $$\begin{aligned}
								\varPhi _{gw}^\pm= & {} \frac{\pi }{2} - L\left( k_0 \pm
								\frac{k_{gw}}{2}\right) \pm \varphi _{gw},
								\end{aligned}$$

5.27 $$\begin{aligned}
								\alpha ^\pm _{gw}= & {} A_{gw} e^{i\varPhi ^\pm _{gw}}
								e^{\pm i\omega _{gw}t}. \end{aligned}$$

Note that $$\alpha ^\pm
								_{gw}$$ must be scaled by the carrier field that is propagating into the space for the complete sideband amplitude.

**Fig. 33:**
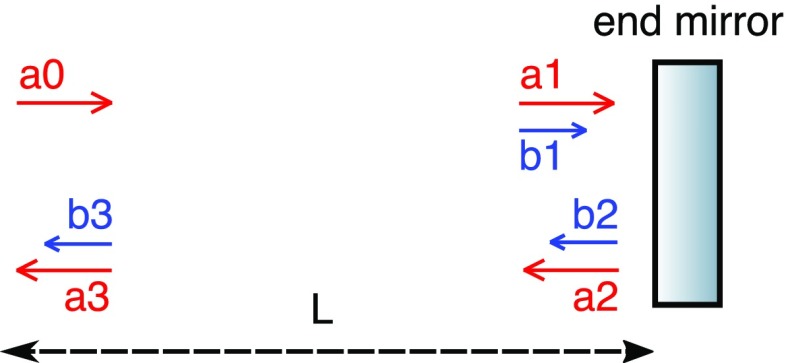
Simplified sketch of a single arm of the Michelson interferometer, the *arrows* show the carrier fields, denoted by *a*, and the signal sidebands *b* at the locations where they are computed

To compute how a Michelson responds to a gravitational wave we must first consider the modulation of the carrier field travelling in both directions along the arms. Both the carrier and the created signal sideband fields propagate along each arm, as shown in Fig. 33, and are reflected by a mirror with amplitude reflectivity $r_{etm}$. The relevant carrier fields are

5.28 $$\begin{aligned}
								a_3= & {} a_2 \exp {(-\mathrm {i}\,k_0 L)},
								\end{aligned}$$

5.29 $$\begin{aligned}
								a_2= & {} r_{etm} a_1,
								\end{aligned}$$

5.30 $$\begin{aligned}
								a_1= & {} a_0 \exp {(-\mathrm {i}\,k_0 L)}.
								\end{aligned}$$

The sidebands that are generated along such an arm are

5.31 $$\begin{aligned}
								b^\pm _1= & {} a_0 \alpha _{gw}^{\pm }, \nonumber \\ b^\pm
								_2= & {} r_{etm} b^\pm _1, \nonumber \\ b^\pm _3= &
								{} b^\pm _2 \exp {(-\mathrm {i}\,(k_0\pm k_{gw})L)} + a_2 \alpha
								_{gw}^{\pm }, \nonumber \\= & {} 2 r_{etm} a_0 \alpha
								_{gw}^{\pm } \exp \left( -\mathrm {i}\,k_0 L\mp \mathrm
								{i}\,\frac{k_{gw}L}{2}\right) \cos \left( \mp \mathrm
								{i}\,\frac{k_{gw}L}{2}\right)
								\end{aligned}$$

and by substituting the sideband amplitude $$\alpha
								_{gw}^{\pm }$$, see Eq. (5.27), we find that:

5.32 $$\begin{aligned}
								b^\pm _3= & {} - \mathrm {i}\,\frac{r_{etm} a_0 w_0
								h_0}{2\omega _{gw}}\sin \left( k_{gw}L\right) \exp \left( -\mathrm
								{i}\,2k_0 L\right) \exp \left( \pm (\omega _{gw}t - \mathrm
								{i}\,k_{gw}L + \varphi _{gw})\right) .\nonumber \\
								\end{aligned}$$

These are the sidebands that will leave the arm due to some gravitational wave modulating the space of an arm. One point to note is that the gravitational wave induced sidebands can cancel themselves out for frequencies $$f_{gw} =
								\frac{Nc}{2L}$$.

Now we assume the Michelson interferometer is operated with a DC offset for the signal readout, see Sect. 5.4. For such a setup the field at the output port is given by Eq. (5.14) which when applied here gives:

5.33 $$\begin{aligned}
								E_{\mathrm {out}}= & {} \mathrm {i}\,2 r t E_0 \cos
								(k_0\varDelta L) + b^+_{N} + b^-_{N} + b^+_{E} + b^-_{E}
								\end{aligned}$$

The gravitational wave signal sidebands created in the North and East arms with perfect end mirrors, $$r_{etm}=1$$, is given by 5.32 where care should be taken to use the correct lengths and carrier term: $$b^\pm _N \equiv
								b^\pm _3$$ with $$L=L_N,\,a_0=rE_0$$ and $$b^\pm _E \equiv
								b^\pm _3$$ with $$L=L_E,\,a_0=itE_0$$. These sidebands at the output port, once transmitted or reflected at the central beam splitter again, are

5.34 $$\begin{aligned}
								b^\pm _N= & {} \frac{rt E_0 w_0 h_0}{2\omega _{gw}}\sin
								\left( k_{gw}L_N\right) \exp \left( -\mathrm {i}\,2k_0 L_N\right)
								\exp \left( \pm (\omega _{gw}t - \mathrm {i}\,k_{gw}L_N + \varphi
								_{gw})\right) , \nonumber \\
								\end{aligned}$$

5.35 $$\begin{aligned}
								b^\pm _E= & {} -\frac{rt E_0 w_0 h_0}{2\omega _{gw}}\sin
								\left( k_{gw}L_E\right) \exp \left( -\mathrm {i}\,2k_0 L_E\right)
								\exp \left( \pm (\omega _{gw}t - \mathrm {i}\,k_{gw}L_E + \varphi
								_{gw})\right) .\nonumber \\ \end{aligned}$$

Note that an extra minus sign is included for the East-arm sidebands because the gravitational wave modulate the North and East arms differentially. Next we will write the arm lengths in terms of a macroscopic differential $$\varDelta
								L$$, and common mode $$\bar{L}$$, lengths: $$L_N = \bar{L} +
								\varDelta L/2$$ and $$L_E = \bar{L} -
								\varDelta L/2$$. Along with this we also assume that the central beam splitter has a 50:50 splitting ration $$r = t =
								1/\sqrt{2}$$, that the common mode length is an integer number of wavelengths for the carrier light $$\exp (\mathrm
								{i}\,k_0\bar{L}) = 1$$, that $$\bar{L} \gg
								\varDelta L$$, and that the gravitational wave’s wavelength is much larger than $$\varDelta
								L$$, so $$k_{gw}(\bar{L} +
								\varDelta L/2) \approx k_{gw}\bar{L}$$. Taking these assumptions into account the sideband terms become

5.36 $$\begin{aligned}
								b^\pm _N= & {} \frac{E_0 w_0 h_0}{4\omega _{gw}}\sin \left(
								k_{gw}\bar{L}\right) \exp \left( -\mathrm {i}\,k_0\varDelta L\right)
								\exp \left( \pm (\omega _{gw}t - \mathrm {i}\,k_{gw}\bar{L} +
								\varphi _{gw})\right) , \nonumber \\
								\end{aligned}$$

5.37 $$\begin{aligned}
								b^\pm _E= & {} -\frac{E_0 w_0 h_0}{4\omega _{gw}}\sin \left(
								k_{gw}\bar{L}\right) \exp \left( \mathrm {i}\,k_0\varDelta L\right)
								\exp \left( \pm (\omega _{gw}t - \mathrm {i}\,k_{gw}\bar{L} +
								\varphi _{gw})\right) .\nonumber \\
								\end{aligned}$$

Finally the sum of the sidebands at the output is

5.38 $$\begin{aligned}
								b^+_{N} \!+\! b^-_{N} \!+ \!b^+_{E} \!+\! b^-_{E} = \frac{\mathrm
								{i}\,E_0 w_0 h_0}{\omega _{gw}}\sin \left( k_{gw}\bar{L}\right) \sin
								\left( k_0\varDelta L\right) \cos \left( \omega _{gw}t -
								k_{gw}\bar{L} \!+\! \varphi _{gw}\right) .\nonumber \\
								\end{aligned}$$

Now that we know the signal sideband fields at the output port, we can combine them with the carrier field that is also present:

5.39 $$\begin{aligned}
								E_{\mathrm {out}}= & {} \mathrm {i}\,E_0 \cos (k_0\varDelta
								L) + b^+_{N} + b^-_{N} + b^+_{E} + b^-_{E} \nonumber \\= &
								{} \mathrm {i}\,E_0 \left[ \cos (k_0\varDelta L) \!+ \!\frac{w_0
								h_0}{\omega _{gw}}\sin \left( k_{gw}\bar{L}\right) \sin \left(
								k_0\varDelta L\right) \cos \left( \omega _{gw}t - k_{gw}\bar{L}
								\!+\! \varphi _{gw}\right) \!\right] \!.\nonumber \\
								\end{aligned}$$

A photodiode placed at the output of the Michelson will then measure the power in this beam from which we want to extract the gravitational wave amplitude, $$h_0$$ and phase, $$\varphi
								_{gw}$$. The power in the beam contains multiple beat frequencies between all the carrier and signal sidebands, with the terms oscillating at the frequency $$\omega
								_{gw}$$, are those linearly proportional to $$h_0$$:

5.40 $$\begin{aligned}
								P_{gw}= & {} |E_0|^2\frac{w_0 h_0}{\omega _{gw}}\sin \left(
								k_{gw}\bar{L}\right) \sin \left( 2k_0\varDelta L\right) \cos \left(
								\omega _{gw}t - k_{gw}\bar{L} + \varphi _{gw}\right) .\qquad
								\end{aligned}$$

As we are using DC readout, the differential arm length is chosen to operate slightly away from the dark fringe of the carrier field $$\varDelta L =
								\frac{\pi }{2 k_0} + \delta _{\mathrm
								{off}}$$ as discussed in Sect. 5.4. The choice of DC offset is typically $$\delta _{\mathrm
								{off}} \ll \lambda _0$$, the wavelength of the carrier light. So for small DC offset the power signal can be approximated as

5.41 $$\begin{aligned}
								P_{gw}\approx & {} 2k_0\delta _{\mathrm
								{off}}|E_0|^2\frac{w_0 h_0}{\omega _{gw}}\sin \left(
								k_{gw}\bar{L}\right) \cos \left( \omega _{gw}t - k_{gw}\bar{L} +
								\varphi _{gw}\right) . \end{aligned}$$

As described before, we now see that some DC offset is required to measure the signal; the DC offset provides the local oscillator field for the signal sidebands to beat with. Finally, the transfer function from a gravitational wave signal to the output photodiode, $$T_{gw\rightarrow
								P}$$, shows that the diode measures $$T_{gw\rightarrow
								P}$$ Watts per unit $$h_0$$ at frequency $$\omega
								_{gw}$$:

5.42 $$\begin{aligned}
								T_{gw\rightarrow P}(\omega _{gw})\approx & {} k_0\delta
								_{\mathrm {off}}|E_0|^2\frac{w_0}{\omega _{gw}}\sin \left(
								k_{gw}\bar{L}\right) e^{ - \mathrm {i}\,k_{gw}\bar{L}}.
								\end{aligned}$$

For an example on how to model the response of a Michelson to a gravitational wave modelled using Finesse see Sect. 5.6.3.

### Finesse examples

#### Cavity power

**Fig. 34:**
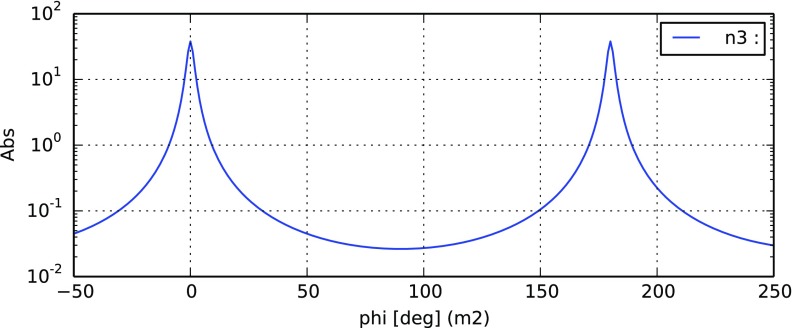
Finesse example: cavity power

This is a simple Finesse example showing the power enhancement in a two-mirror cavity as a function of the microscopic tuning of a mirror position (the position is given in degrees with 360$^{\circ }$ referring to a change of longitudinal position by one wavelength). Compare this plot to the one shown in Fig. 27, which instead shows the power enhancement as a function of the laser frequency detuning (Fig. 34).

**Finesse input file for ‘Cavity power’** 
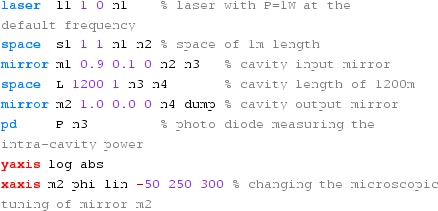


#### Michelson power

**Fig. 35:**
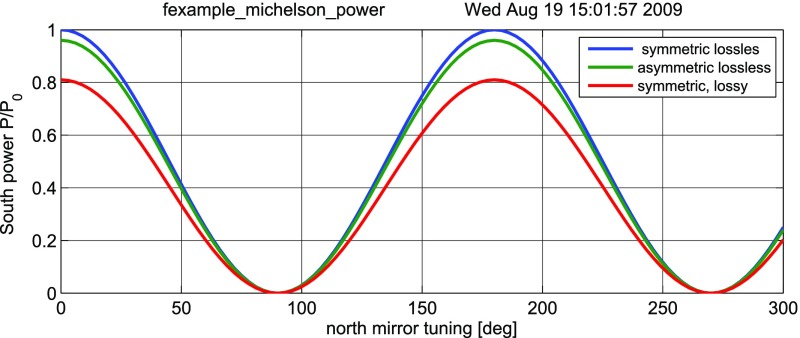
Finesse example: Michelson power

The power in the South port of a Michelson detector varies as the cosine squared of the microscopic arm length difference. The maximum output can be equal to the input power, but only if the Michelson interferometer is symmetric and lossless. The tuning for which the South port power is zero is referred to as the *dark fringe* (Fig. 35).

**Finesse input file for ‘Michelson power’** 
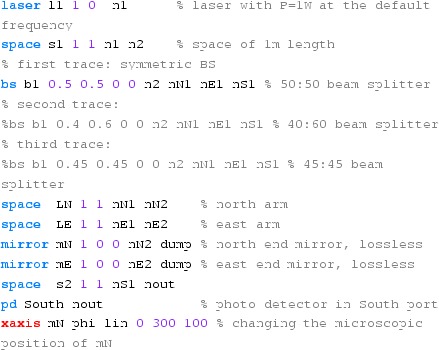


#### Michelson gravitational wave response

**Fig. 36:**
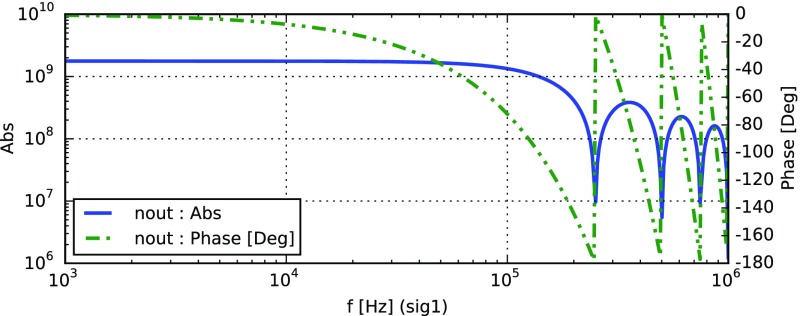
Finesse example: Michelson sideband output from a gravitational wave

This is a simple Finesse example showing how the arm spaces can be modulated to produce the effect a gravitational wave would have on it. It outputs the amplitude and phase of the upper sideband that reaches the output port. The dips in amplitude occur when the travel time of the photons along the interferometer arms equals one gravitational wave period and hence the signal accumulated in the first and second half of the travel time cancel each other (the plot above does not have enough resolution to show that the dips indicate zero signal, the non-zero amplitudes are an artefact of the numerical plotting routine). In this example the frequencies of the dips are given as $$f=N\,c/1200\,\mathrm{m} =
									N\,250\,\mathrm{kHz}$$, with *N* a positive integer (Fig. 36).

**Finesse input file for ‘Michelson gravitational wave response’** 
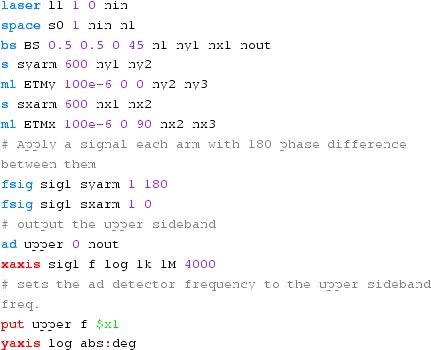


## Radiation pressure and quantum fluctuations of light

Once classical noise sources are sufficiently reduced, the quantum fluctuations of light become one of the limiting noise sources for interferometric gravitational-wave detectors (Braginskii and Vorontsov 1975; Jaekel and Reynaud 1990; Meers and Strain 1991; Niebauer et al. 1991). To reduce this quantum noise the basic Michelson interferometer has been significantly altered over time, as we discuss in Sect. 7. This section aims to outline what quantum noise is and how its effects can be calculated.

The coupling of the quantum fluctuations of light into the output signal of the detector has traditionally been described as two separate effects: shot noise in the output current of the photodiodes and radiation pressure effects due to the use of suspended optics. Caves has shown that both noise components can be understood as originating from vacuum fluctuations coupling into the dark port of the Michelson interferometer (Caves 1981) and the two-photon formalism suggested by Caves and Schumaker (1985) has led to a large body of work towards understanding and reducing quantum noise in gravitational wave interferometers (Miao et al. 2014; McClelland et al. 2011; Chen et al. 2010; Müller-Ebhard et al. 2009; Corbitt et al. 2005; Buonanno et al. 2003).

In the following we outline a method to compute quantum noise in interferometer output ports using sidebands and the classical framework presented in Sects. 2, 3 and 4. We apply this method to investigate the quantum noise limits of several interferometer readout schemes and finally discuss how suspended optics effect the quantum noise.

The interested reader can explore this topic further with a modern and comprehensive treatment of quantum noise in the review provided in Danilishin and Khalili (2012) and the following references: the standard quantum limit (Caves 1981; Jaekel and Reynaud 1990) squeezing (Loudon and Knight 1987; Vahlbruch et al. 2007) and quantum non-demolition interferometry (Braginsky et al. 2000; Giovannetti et al. 2004).

### Quantum noise sidebands

The two quadratures of the light field, its amplitude and phase, form an observable conjugate pair thus both cannot be measured simultaneously without some uncertainty in the result (Caves and Schumaker 1985). This quantum noise of a single mode laser can be depicted as a phasor with the coherent carrier field and the addition of some stochastic Gaussian-distributed noise which affects both its phase and amplitude (Bachor and Manson 1990; Meers and Strain 1991). The quantities $$\sigma ^2_{\phi
								}$$ and $$\sigma
								^2_{a}$$ are the variances that characterise fluctuations in phase and amplitude respectively. The noise present in a light field with an equal, minimum $$\sigma _\phi
								$$ and $$\sigma
								_a$$ is known as *vacuum fluctuations* or *vacuum noise*. Vacuum noise can be understood as the photon at all frequencies being incoherently created and annihilated. Therefore vacuum noise is all-pervasive, existing at all locations in space, at every frequency and in every spatial mode. Such photons also enter our interferometer and limit the sensitivity of any measurement of a field’s amplitude or phase.

**Fig. 37:**
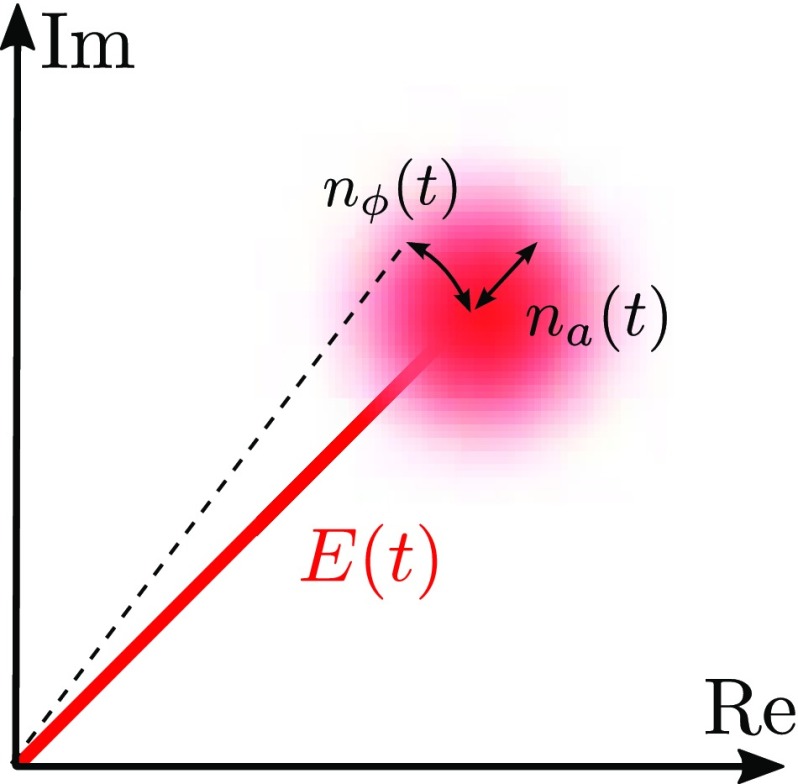
Phasor diagram of Eq. (6.4) depicting the Gaussian random amplitude and phase fluctuations due to vacuum noise. Here $$n_{a,\phi
											}(t)$$ are random gaussian noises in either the phase or amplitude of the carrier. Shown is only the positive frequency part of the carrier field, as *E*(*t*) is real a conjugate negative frequency term also exists

Consider a carrier field at one location with amplitude $$a_0$$ and frequency $$\omega
								_0$$ along with a continuum of noise fields (the positive frequency spectrum):

6.1 $$\begin{aligned}
								E(t) = \frac{a_0}{2} e^{\mathrm {i}\,\omega _0 t} + \frac{1}{2}\int
								_0^\infty q(\omega )e^{\mathrm {i}\,\omega t}\,d\omega +
								\mathrm{c.c}. \end{aligned}$$

where $$q(\omega
								)$$ is the Fourier component of a stochastic process, representing the vacuum fluctuation of the electric field.

We can rewrite the continuum of noise in reference to the carrier field frequency:

6.2 $$\begin{aligned}
								E(t) = \frac{a_0}{2} e^{\mathrm {i}\,\omega _0 t} + \frac{e^{\mathrm
								{i}\,\omega _0 t}}{2}\int _{-\omega _0}^\infty q(\omega _0+\omega
								)e^{\mathrm {i}\,\omega t} \,d\omega + \mathrm{c.c}.
								\end{aligned}$$

where we can view our quantum noise fields as sidebands of the carrier instead. For gravitational-wave detectors the bandwidth *B* of the signals induced by a gravitational wave is of the order of several kHz and thus $$B \ll \omega
								_0$$. Hence, we can focus on a small range of the noise sidebands that will actually affect our sensitivity:

6.3 $$\begin{aligned}
								E(t) = \frac{1}{2}\left[ a_0 + \int _{-B}^B q(\omega _0+\varOmega
								)e^{\mathrm {i}\,\varOmega t} \,d\varOmega \right] e^{\mathrm
								{i}\,\omega _0 t} + \mathrm{c.c}.
								\end{aligned}$$

Here $$\varOmega
								$$ will be used in notation to refer to frequencies in the signal bandwidth with $$-B <
								\varOmega \le B \ll \omega _0$$. We can also represent the quantum fluctuations as noise in both amplitude and phase:

6.4 $$\begin{aligned}
								E(t) = [a_0 + n_a(t)] e^{\mathrm {i}\,\omega _0 t + n_\phi (t)/a_0}
								+ \mathrm{c.c}= [a_0 + n_a(t) + \mathrm {i}\,n_\phi (t)]e^{\mathrm
								{i}\,\omega _0 t} + \mathrm{c.c}. ,
								\end{aligned}$$

with $$n_a$$, $$n_\phi
								$$ being real amplitudes of the amplitude and phase fluctuations (of the stochastic process) with $$n_a$$, $$n_\phi \ll
								1$$. This equation is represented in the phasor diagram in Figs. 37, and 38 shows how to position such the noise phasors in a sideband spectrum.

We can now relate the amplitude and phase fluctuation to the complex quantum noise $$q(\omega
								)$$:

6.5 $$\begin{aligned}
								q(\omega ) = n_a(\omega ) + \mathrm {i}\,n_\phi (\omega )
								\end{aligned}$$

Both $$n_{a,\phi
								}(\omega )$$ of a vacuum noise sideband are characterised by a Gaussian probability density function with a mean $$\mu _{a,\phi
								}=0$$ and variance $$\sigma
								^2_{a,\phi }$$. Note that the sidebands for the quantum noise are not representing a coherent and deterministic signal. This semi-classical approach is sufficient to motivate the design choices in laser interferometers for gravitational wave detection. A rigorous approach would require to use operators instead of sidebands. This approach is beyond the scope of this article, and instead fully covered in the review article by Danilishin and Khalili (2012).

**Fig. 38:**
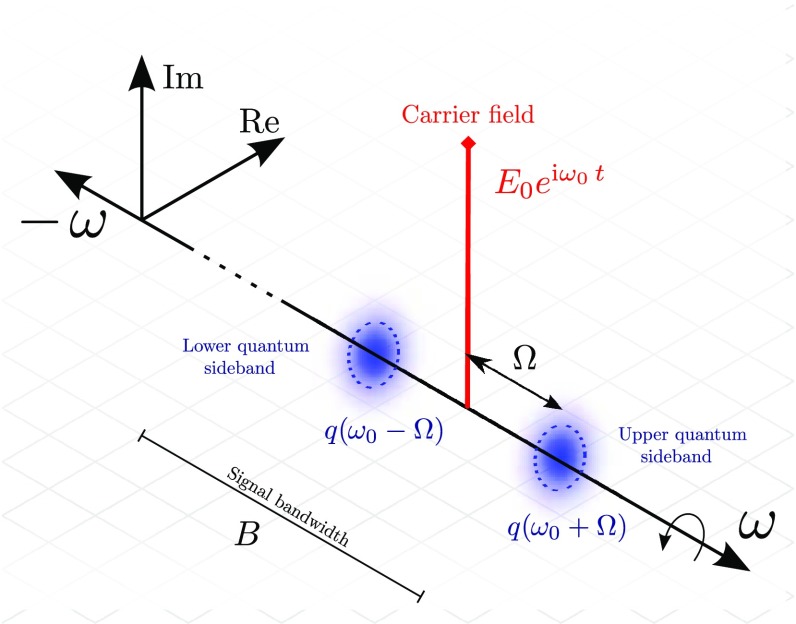
This diagram depicts a carrier field as shown in Fig. 37 but expanded to show the vacuum noise sideband phasors that contribute towards the noise. The amplitude and phase of each sideband is a stochastic Gaussian noise so that its real and imaginary parts are described by some probability distribution depicted by the *blue faded region*, the *dashed circle* represents the standard deviation of such fluctuations. The signal bandwidth *B* can be imagined as containing an infinite number of such vacuum noise sidebands, each oscillating with a random phase and amplitude. Pictured are two upper and lower sidebands selected from this continuum of vacuum noise. The negative frequency phasors are not shown, they would be the mirrored conjugate versions of the positive phasors

The variances $$n_{a,\phi
								}(\omega )$$ are limited by the minimum uncertainty in the relation

6.6 $$\begin{aligned}
								\sigma _\phi \sigma _a \ge \frac{\hbar \omega }{2},
								\end{aligned}$$

which gives for an integration time of one second, $$\sigma ^2_{\phi
								} = \sigma ^2_{a} = \hbar \omega /2$$. As the phase and amplitude of $$q(\omega
								)$$ is random we can only compute its *expected value* or *ensemble value* at a particular frequency:

6.7 $$\begin{aligned}
								\langle q(\omega ) \rangle = \langle \mu _a \rangle + \mathrm
								{i}\,\langle \mu _\phi \rangle = 0,
								\end{aligned}$$

which is zero as the mean of the noise is zero, hence on average no sidebands are actually observed. We can also consider the covariance between any two sidebands at frequency $$\omega
								$$ and $$\omega
								'$$. As $$q(\omega
								)$$ is a complex value there are multiple ways the covariance can be taken when considering the conjugates of either sideband, for example $$\langle q(\omega
								)q^*(-\omega ') \rangle $$, $$\langle
								q^*(\omega )q(-\omega ') \rangle $$, etc.. However as the fluctuations in amplitude and phase at different frequencies are independent, the covariance between any two vacuum noise sidebands is:

6.8 $$\begin{aligned}
								\langle q(\omega )q^*(\omega ') \rangle= & {} \frac{\hbar
								\omega }{2}\delta (\omega -\omega ') ,
								\end{aligned}$$

6.9 $$\begin{aligned}
								\langle q(\omega )q(\omega ') \rangle= & {} 0 ,
								\end{aligned}$$

The delta function in the covariance signifies there is no correlation between different frequencies. The auto-covariance is then $$\langle q(\omega
								)q^*(\omega ) \rangle \propto \delta (\omega -\omega ) = \infty
								$$, which may seem odd at a first glance. However, this can be better understood in the time domain picture, as we are measuring our signal over an idealistic infinite time span and as our noise is Markovian (and therefore also ergodic), the time average of the power of a signal will be infinitely large.

**Noise power spectral densities**

Noise, i.e., a random signal, can be quantified using a *power spectral density* (PSD) which is a measure of the power in a signal per frequency. The definition of a single-sided PSD of some frequency domain value $$x(\omega
								)$$ is:

6.10 $$\begin{aligned}
								S_{xx}(\omega )\delta (\omega -\omega ')= & {} 2\langle
								x(\omega )x^*(\omega ') \rangle ,
								\end{aligned}$$

with units $$[x]^2/\mathrm{Hz}$$. The cross-spectral-density between two values $$x(\omega
								)$$ and $$y(\omega
								)$$ is similarly:

6.11 $$\begin{aligned}
								S_{xy}(\omega )\delta (\omega -\omega ')= & {} 2\langle
								x(\omega )y^*(\omega ') \rangle .
								\end{aligned}$$

The eventual physical noise we wish to compute is the noise in the demodulated photocurrent of the photodiode measuring the interferometer output signal, here we will consider only photodiodes with 100 % quantum efficiency.^9^ The photocurrent *I* is proportional to the detected light power $$I(t){\sim }
								P(t)$$ and the PSD of the noise in the photocurrent is:

6.12 $$\begin{aligned}
								S_I(\omega )\delta (\omega -\omega ') = 2\langle I(\omega
								)I^*(\omega ') \rangle \end{aligned}$$

The DC and $$\omega \pm
								\varOmega $$ terms of the power on a photodiode for a single carrier with quantum noise sidebands is:

6.13 $$\begin{aligned}
								P(t) = E(t)E^*(t)= & {} |a_0|^2 + a_0^*\int _{-B}^{B}
								q(\omega _0+\varOmega ) e^{\mathrm {i}\,\varOmega t} \,d\varOmega
								\nonumber \\&+\, a_0 \int _{-B}^{B} q^{*}(\omega
								_0+\varOmega ) e^{-\mathrm {i}\,\varOmega t} \,d\varOmega + O(q^2),
								\end{aligned}$$

terms of the order $$q^2$$ are assumed to be a negligibly small contribution. The positive half of the photocurrent spectrum for $$0<\varOmega \le
								B$$ is given by its Fourier transform:

6.14 $$\begin{aligned}
								I(\varOmega )\equiv & {} \mathcal {F}[I(t)]= a_0^*q(\omega
								_0+\varOmega ) + a_0 q^*(\omega _0-\varOmega ).
								\end{aligned}$$

The spectrum for frequencies in the signal bandwidth is thus defined by just quantum noise scaled by the carrier field. From this point on for the sake of brevity we will define the following notation without the carrier frequency, as we are only using a single carrier for this derivation:

6.15 $$\begin{aligned}
								q(\omega _0\pm \varOmega ) \Rightarrow q_\pm \,\,\,
								\mathrm{and}\,\,\,q(\omega _0\pm \varOmega ') \Rightarrow q'_\pm .
								\end{aligned}$$

Using Eqs. (6.12) and (6.14), the PSD of the photocurrent is:

6.16 $$\begin{aligned}
								S_I(\varOmega )\delta (\varOmega -\varOmega ')= & {}
								2P_0\bigl (\bigl< q_+q'^*_+ \bigr> + \bigl<
								q_-q'^*_- \bigr>\bigr ) + 2a_0^2\bigl<q_-q'_+
								\bigr>^*+ 2{a_0^2}^*\bigl <q_+q'_- \bigr
								>.\nonumber \\ \end{aligned}$$

Now applying Eqs. (6.8) and (6.9) in Eq. (6.16) the noise PSD for a single carrier field with vacuum noise is:

6.17 $$\begin{aligned}
								S_I(\omega _0\pm \varOmega )\delta (\varOmega -\varOmega ')=
								& {} 2 P_0 \left( \bigl<q_+ q_+'^*\bigr> +
								\bigl <q_- q_-'^*\bigr >\right) , \nonumber \\=
								& {} P_0 \left( \hbar (\omega _0+\varOmega ) + \hbar (\omega
								_0-\varOmega )\right) \delta (\varOmega -\varOmega ') \nonumber \\
								S_I(\omega _0\pm \varOmega )= & {} 2 P_0 \hbar \omega _0 .
								\end{aligned}$$

Here we see that the quantum noise of a single carrier field does not depend on the sideband frequency $$\varOmega
								$$. The vacuum fluctuations interfering with our carrier field produces a broadband frequency-independent noise source proportional to the carrier power and frequency. It should also be noted that Eq. (6.17) is the same result as the semi-classical Schottky shot-noise equation, Eq. (6.44). An interesting aspect to note here are the differing reasons for the presence of this quantum or shot noise. The Schottky formula derives this noise from the Poisson statistics of electrons generated in the photocurrent due to the light field power. Whereas the quantum approach reasons that such fluctuations in the photocurrent are in fact due to vacuum noise superimposing itself onto our light fields introducing a noise into our measurements.

The description of quantum noise with semi-classical sidebands has the advantage that the propagation of a stochastic signal through a linear system is described by the same transfer functions as for a deterministic signal. Therefor we can use the classical model of the optical system to compute the propagation of the quantum noise as well as any signal.

### Vacuum noise and gravitational-wave detector readout schemes

**Fig. 39:**
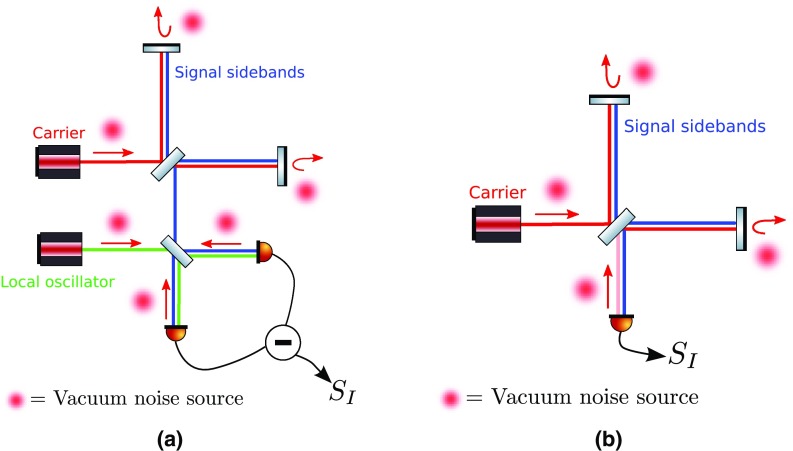
Shown are two possible readout schemes that can be used to extract signal sideband information from a Michelson along with the various classical fields and sources of vacuum noise, **a** balanced homodyne, **b** DC offset

Let us now consider a Michelson interferometer, as described in Sect. 5.2, and the limiting sensitivity due to vacuum noise leaking into the detector. Figure 39 depicts two example readout schemes for measuring the gravitational wave signal. For both schemes we can identify the sources of vacuum noise that will enter the interferometer. The input is assumed to be a perfect single-mode laser whose noise is purely vacuum noise. The end mirrors in the arms are taken to be perfectly reflective thus no vacuum noise enters through them; however if $r<1$ any vacuum noise leaking out would be replaced with an equal amount of uncorrected noise injected back in. The output port is fully open and thus allow vacuum noise to enter into the system proving the primary contribution of noise in Michelson setup used for gravitational-wave detectors. This is due to the fact that such the Michelson is operated on the dark fringe for the input carrier, meaning any laser noise will leave the system back towards the laser, whereas the noise entering through the output port will return to the output port.

When no non-linear optical effects (effects proportional to the beam’s power) are present in an interferometer and the only quantum noise present is uncorrelated vacuum noise, there will always be the same amount of vacuum noise incident on any photodiode. This is irrespective of the topology of the interferometer or components used because noise can never be effectively lost from the system; an equivalent amount of uncorrelated noise is always injected back in. In such cases propagation of the noise sidebands through the interferometer do not need to be computed. Instead, when computing $S_I$ at any of the photodiodes shown in Fig. 39 we only need to consider pure vacuum noise sidebands and the local oscillator field, $E_{LO}$; the source of location of the vacuum noise sources is not of importance. This is why for early generation gravitational-wave detectors, which had negligible non-linear optical effects, the semi-classical Schottky expression could be used to estimate the quantum noise correctly.

The detailed computation of quantum noise limited sensitivity of a detector depends on the readout scheme used. Early generations of gravitational wave detectors such as LIGO, Virgo, GEO 600 and TAMA300 used heterodyne readout schemes, where RF modulation sidebands applied to the input field are used as local oscillators at the output (see Sect. 5.4). However, such schemes included some technical challenges, the oscillator noise of the RF modulator being one of them, and also increase the shot-noise level when demodulating the photocurrent (Meers and Strain 1991; Niebauer et al. 1991; Rakhmanov 2001; Buonanno et al. 2003). Thus the next generation of detectors opted for a DC readout scheme (Fricke et al. 2012; Hild et al. 2009), see Sect. 8.16. Both schemes depicted in Fig. 39 use a form of DC readout, which we will analyse in more detail in the following sections. We do not cover the computation of quantum noise with RF modulation readout schemes, the interested reader should see Harms et al. (2007), Rakhmanov (2001) and Buonanno et al. (2003).

*Noise-to-signal ratio for DC offset*

A DC offset in the main Michelson interferometer (see Sect. 5.4) provides a local oscillator by making the interferometer operate slightly away from the dark fringe for the carrier, and hence allowing some to leak through to the output port along with any signal sideband fields. The sources of vacuum noise that will contribute to the quantum noise are shown in Fig. 39b; however, as stated previously the total amount of noise present at the photodiode will be just pure vacuum noise as it is assumed that there are no non-linear optical effects. The local oscillator field at the output is given by Eq. (5.11) and along with the vacuum noise sidebands the output field is:

6.18 $$\begin{aligned}
								E_{\mathrm {out}}= & {} \left[ \mathrm {i}\,E_0
								e^{-i2k\bar{L}}\sin (k_0 \delta _{\mathrm {off}}) + q_+e^{\mathrm
								{i}\,\varOmega t} + q_-e^{-\mathrm {i}\,\varOmega t}\right]
								e^{\mathrm {i}\,\omega _0 t}.
								\end{aligned}$$

where we have used the dark fringe offset as stated in Eq. (5.13). The quantum noise PSD when using a DC offset is now essentially the same scenario as when deriving Eq. (6.17), where a single carrier and noise sidebands were considered; except that the carrier power now depends on $$\delta _{\mathrm
								{off}}$$:

6.19 $$\begin{aligned}
								S_{\mathrm {P,DC}}= & {} \langle |P_{\mathrm
								{out}}(\varOmega )|^2\rangle = 2 P_{0}\sin ^2\left( k_0\delta
								_{\mathrm {off}}\right) \hbar \omega _0 \approx 2P_{0}(k_0\delta
								_{\mathrm {off}})^2 \hbar \omega _0 ,
								\end{aligned}$$

where $$P_0$$ is the power of the laser injected into the Michelson and $$k_0\delta
								_{\mathrm {off}} \ll 1$$.

To compute the noise-to-signal (NSR) ratio, which is used to describe the sensitivity of our Michelson, the transfer function from a signal we want to measure to the photodiode output is required. Here we will use the gravitational wave signal transfer function from Eq. (5.42) which describes the Watts of power per unit of strain, *h*, at the output detector

6.20 $$\begin{aligned}
								\left| T_{gw\rightarrow P}(\omega _{gw})\right|\approx & {}
								k_0\delta _{\mathrm {off}}P_0\frac{w_0}{\omega _{gw}}\sin \left(
								\frac{\omega _{gw}\bar{L}}{c}\right) \, \mathrm{W}/\mathrm{h}.
								\end{aligned}$$

We note that $$T_{gw\rightarrow
								P}$$ refers to the *amplitude* of the differential length modulation that the arms experience. Thus the NSR should be computed with the *amplitude spectral density* given as $$\mathrm{ASD} =
								\sqrt{\mathrm{PSD}}$$:

6.21 $$\begin{aligned}
								\mathrm{NSR} = \frac{{\sqrt{S_{\mathrm{P,DC}}}}}{T_{gw\rightarrow
								P}} = \sqrt{\frac{2\hbar }{P_0 \omega _0}} \frac{\omega _{gw}}{\sin
								(\omega _{gw}\bar{L}/c)} \,\, \frac{\mathrm{h}}{\sqrt{\mathrm{Hz}}},
								\end{aligned}$$

and example plot of such a sensitivity is shown in Fig. 40. The displacement sensitivity does not depend on the DC offset and can be improved, for example, by increasing the laser power. Eventually, building a more powerful laser is not possible without sacrificing stability in power and frequency. Instead we can also use a Fabry–Perot cavity (power recycling) to increase the effective power inside the interferometer, see Sect. 7.

*Noise-to-signal ratio for balanced homodyne*

Balanced homodyne readout involves the use of an external local oscillator whose optical frequency is the same as the main carrier light in the interferometer. The main Michelson interferometer is operated on the dark fringe for the carrier so no carrier light is present at the output port. This local oscillator is mixed with the signal sidebands using a beam splitter, such a setup is depicted in Fig. 39a and in more detail for the readout in Fig. 41. As the signal sidebands are now split into two optical paths we require two photodiodes to measure the signal, otherwise half the signal will be lost instantly. The balanced aspect of this readout scheme refers to the fact that the two photocurrents $I_a$ and $I_b$ are combined in such a way that the noise from either the local oscillator port or the signal port can be completely removed from the measurement.

**Fig. 40:**
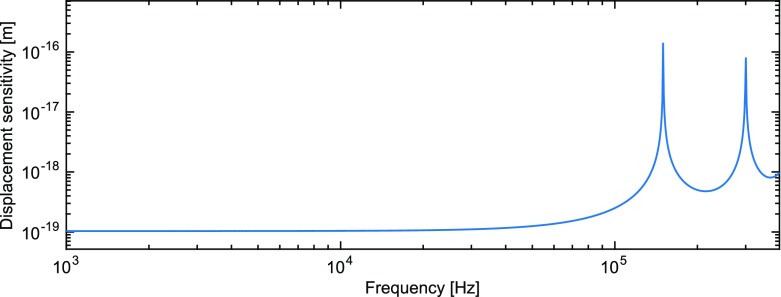
Shot-noise limited sensitivity of a Michelson, see Eq. (6.21), with $\bar{L}=1000$ m, $R=T=0.5$ and $P_0=1$ W

**Fig. 41:**
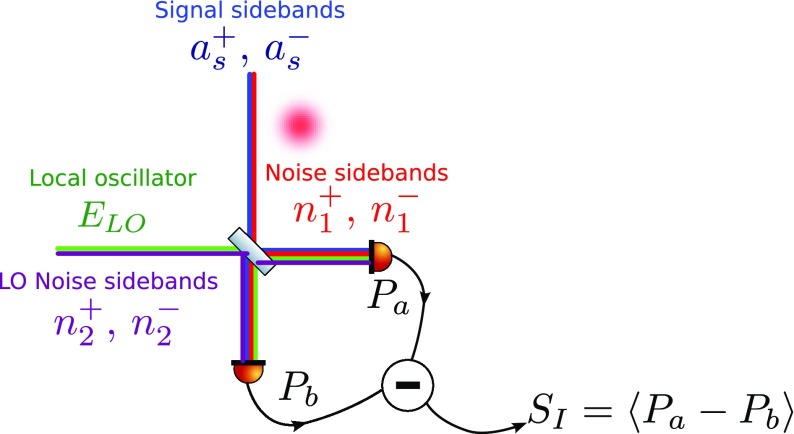
The signal and noise fields in the homodyne detector as used in the balanced homodyne readout scheme

No current generation gravitational-wave detector uses this form of homodyne readout for extracting gravitational wave signals. This has been due to the additional technical challenges which are not present when using DC readout. It is however used extensively for quantum noise measurements when non-vacuum states are injected into interferometers (Stefszky et al. 2012; Chua et al. 2014) and offers potential benefits over DC readout if the technical challenges can be overcome, as we show later in this section. Although not currently used, such a readout scheme is a current topic of investigation for future generations of detectors for extracting gravitational-wave signals (Fritschel et al. 2014).

There are two possible sources for the local oscillator field when using balanced homodyne detection: a separate laser system or a pick-off of the same carrier field used in the interferometer. The former is technically challenging as the separate system must be locked to the input laser to ensure temporal coherence when beating with the signal sidebands. The latter option of using a pick-off beam does not have this issue as it is from the same laser. Other technical challenges that exist for both options are (McKenzie et al. 2007) that the beam splitter is exactly 50:50; that the signal sidebands and local oscillator fields have a particularly good spatial overlap, also referred to as *mode-matching* and that the local oscillator does not back-scatter into the output port of the interferometer.

Assuming a perfect 50:50 beam splitter and a coherent local oscillator which is well aligned to some signal beam we want to measure, both the incoming signal and local oscillator include vacuum noise. To calculate the photocurrent noise $S_I$ we describe the noise sidebands as shown in Fig. 41. In the following we ignore the signal sidebands $a_s^+$, $a_s^-$ and compute just the noise floor of the detector:

6.22 $$\begin{aligned}
								E_a = \left[ r(n_1^+e^{\mathrm {i}\,\varOmega t} + n_1^-e^{-\mathrm
								{i}\,\varOmega t}) + \mathrm {i}\,t(E_{LO} + n_2^+e^{\mathrm
								{i}\,\varOmega t} + n_2^-e^{-\mathrm {i}\,\varOmega t})\right]
								e^{\mathrm {i}\,\omega _0 t} \nonumber \\ E_b = \left[ r (E_{LO} +
								n_2^+e^{\mathrm {i}\,\varOmega t} + n_2^-e^{-\mathrm {i}\,\varOmega
								t}) + \mathrm {i}\,t(n_1^+e^{\mathrm {i}\,\varOmega t} +
								n_1^-e^{-\mathrm {i}\,\varOmega t})\right] e^{\mathrm {i}\,\omega _0
								t} \end{aligned}$$

The photocurrent noise PSD is then proportional to:

6.23 $$\begin{aligned}
								S_I \propto \left\langle \left| E_aE^*_a - E_bE^*_b\right| ^2
								\right\rangle \end{aligned}$$

For the incident power on each photodiode we ignore noise terms that are not scaled by the local oscillator as negligible:

6.24 $$\begin{aligned}
								P_a(t)= & {} -\mathrm {i}\,t E^*_{LO} \left[
								r(n_1^+e^{\mathrm {i}\,\varOmega t} + n_1^-e^{-\mathrm
								{i}\,\varOmega t}) + \mathrm {i}\,t(n_2^+e^{\mathrm {i}\,\varOmega
								t} + n_2^-e^{-\mathrm {i}\,\varOmega t})\right] +\mathrm{c.c}
								\nonumber \\ P_b(t)= & {} r E^*_{LO} \left[ \mathrm {i}\,t
								(n_1^+e^{\mathrm {i}\,\varOmega t} + n_1^-e^{-\mathrm {i}\,\varOmega
								t}) + r (n_2^+e^{\mathrm {i}\,\varOmega t} + n_2^-e^{-\mathrm
								{i}\,\varOmega t})\right] +\mathrm{c.c}\nonumber \\
								\end{aligned}$$

Assuming that each photodiode is identical in its response to the power, the photocurrents proportional to these two powers can then be subtracted or summed:

6.25 $$\begin{aligned}
								P_a(t) \pm P_b(t)= & {} E^*_{LO}\left[ \mathrm {i}\,rt
								(n_1^+e^{\mathrm {i}\,\varOmega t} + n_1^-e^{-\mathrm {i}\,\varOmega
								t})(-1\pm 1) \right. \nonumber \\&\left. +\,
								(n_2^+e^{\mathrm {i}\,\varOmega t} + n_2^-e^{-\mathrm {i}\,\varOmega
								t})(T\pm R) \right] +\mathrm{c.c} .
								\end{aligned}$$

This shows that either the noise from the local oscillator, $$n_2^\pm
								$$, or that coming along with the signal, $$n_1^\pm
								$$, can be removed. Typically the local oscillator noise will be larger than that accompanying the signal thus we can compute $$P_a -
								P_b$$ to remove it. It can also be seen here if the beam splitter is not 50:50, $$R \ne
								T$$, the local oscillator noise cannot be fully removed. Finally the subtracted photocurrent for the sideband frequency $$\varOmega
								$$ is:

6.26 $$\begin{aligned}
								P_{a-b}(\varOmega ) \equiv \mathcal {F}[P_a(t) - P_b(t)](\varOmega
								)= & {} -\mathrm {i}\,E^*_{LO} (n_1^+ + {n_1^-}^*),
								\end{aligned}$$

where $$r=t=1/\sqrt{2}$$. For pure vacuum noise, $$n_{1}^\pm
								\Rightarrow q_\pm $$, the resulting photocurrent noise PSD for this is that given by Eq. (6.17):

6.27 $$\begin{aligned}
								S_I= & {} \langle \left| P_{a-b}(\varOmega ) \right|
								^2\rangle = 2P_{LO} \left( \langle q_+ q_+^*\rangle + \langle
								q_-q_-^*\rangle \right) ,\nonumber \\= & {} 2 P_{LO} \hbar
								\omega _{0} \end{aligned}$$

Therefore if correctly balanced the quantum noise is no greater than what is present for a DC offset readout. If the local oscillator power $P_{LO}$ is identical to the carrier power in the DC offset scheme, then the sensitivity for the balanced homodyne detection is the same as that for the DC offset detection, stated in Eq. (6.21), because the transfer function from signal to the two photodiodes is essentially the same. One aspect where it differs however is the phase of the local oscillator relative to the signal sidebands which is now a free parameter, which is known as the *homodyne angle* or the *readout phase*. When using a DC offset readout scheme the readout phase is essentially fixed. Having the ability to vary this readout phase provides an extra degree of freedom for optimising the quantum noise-to-signal ratio for gravitational wave signals. This is an assumed feature in some *quantum non-demolition* schemes (Braginsky et al. 1980; Braginsky and Khalili 1996) which introduce new methods for reducing the quantum noise (Purdue and Chen 2002; Kimble et al. 2002; Khalili and Levin 1996; Chen 2003; Chen et al. 2010).

### Quantum noise with non-linear optical effects or squeezed states

**Fig. 42:**
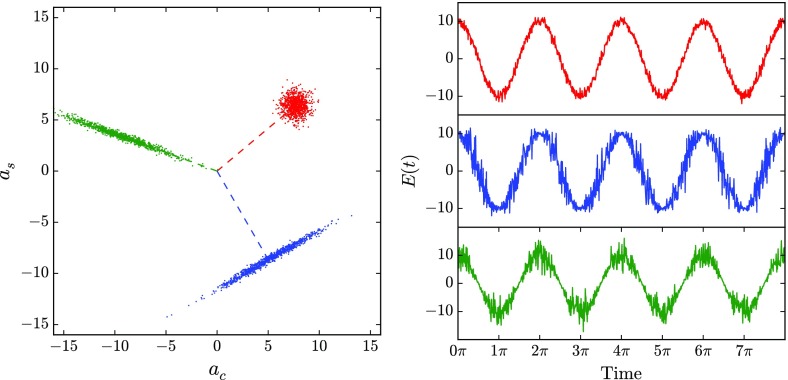
Depicted are the phasor and the time signals of pure vacuum noise (*red*), amplitude-squeezed (*blue*) and phase-squeezed (*green*) noise. The effect here is greatly exaggerated to produce a visible noise thus the scaling on the axes do not represent any realistic values. The phasor diagram shows *E*(*t*) at some arbitrary time value and each point is a sample retrieved from the probability density function of the noise. The squeezed states show clearly a correlation between phase and amplitude fluctuations; the area of each state is equal representing the minimum given by the uncertainty relation 6.6

Up to this point we have only considered pure vacuum noise and linear optical effects, both of which are valid approximations for previous generations of gravitational wave detectors. As the effective laser power in the interferometer is increased to reduce shot noise, the radiation pressure exerted on suspended optics by the circulating laser beams will increase another noise significantly, the *radiation pressure noise*. As the suspended mirrors are free to move under the influence of radiation pressure, any fluctuation in the laser’s power will couple back into itself as phase modulation. This is a non-linear process as the amplitude of the motion is proportional to the power in the beam, which leads to the upper and lower sidebands becoming correlated with one another. As explained in Sect. 6.4, such noise is prominent at low frequencies.

Then there is also the possibility of *squeezing* the vacuum noise, whereby still satisfying the relationship 6.6, the uncertainty in either the phase or amplitude is increased whilst being and decreased in the other. This squeezed noise can be represented by correlated noise sidebands (Caves and Schumaker 1985; Danilishin and Khalili 2012), Fig. 42 shows qualitatively the effect of squeezed vacuum noise on a coherent field. Injecting squeezed noise into the output port of the interferometer can thus reduce the dominant vacuum noise. Upon returning to the output port the noise should still be squeezed, but to a slightly lesser degree due to various optical losses which degrade the amount of squeezing. If squeezing is implemented effectively, the noise can be reduced below the typical shot-noise level of $$2P_0\hbar \omega
								_0$$, thus providing a broad improvement in shot-noise limited regions of the detectors sensitivity (Caves 1981). Although we will not cover squeezing in detail in this article, squeezed light injection has been used routinely by the GEO 600 since 2010 (LIGO Scientific Collaboration 2011), and further upgrades to advanced gravitational-wave detectors using squeezed light sources are actively being developed (Oelker et al. 2014).

One important aspect to note here is that when either non-linear optical effects or non-vacuum states of light are significant, the correlations introduced in the propagation of light fields through the interferometer have to be considered. This is due to the fact that the noise sidebands will be altered in amplitude and phase and the correlation between sidebands introduced as they propagate becomes an important feature. Such a calculation involves constructing the full interferometer matrix, see Sect. 2.3, for the noise sideband frequencies and including, if so required, the radiation pressure coupling at suspended mirrors as discussed in the next section.

### Radiation pressure coupling at a suspended mirror

As the laser power is increased to reduce the shot-noise, the higher power results in a significant radiation pressure force being exerted on the interferometer mirrors. The frequency spectrum of the force exerted on a perfectly reflectivity mirror by a single beam with power $$P(\varOmega
								)$$ is given by (Meystre et al. 1985)

6.28 $$\begin{aligned}
								F_{rp}(\varOmega ) = \frac{2 P(\varOmega )}{c}.
								\end{aligned}$$

In order to attenuate seismic vibrations and produce free-masses as probes for gravitational waves, the mirrors in gravitational-wave detectors are suspended via a series of active and passive suspension systems. At frequencies well above the resonances of the suspension systems the mirrors can be considered to be free (or quasi-free). Any fluctuation in the light power induces a motion in the suspended mirrors. This process converts power fluctuations into phase fluctuations, and this coupling can lead to optomechanical effects such as *optical springs*, which couple the motion of multiple suspended optics together (Sheard et al. 2004; Aspelmeyer et al. 2014).

The induced longitudinal motion of a suspended mirror due to $N_f$ separate forces being applied to it is:

6.29 $$\begin{aligned}
								\delta z(\varOmega ) = H(\varOmega ) \sum _{n=0}^{N_f} F_n(\varOmega
								), \end{aligned}$$

where $$H(\varOmega
								)$$ is the *mechanical susceptibility* or *mechanical transfer function* from a force applied to motion parallel to the mirrors surface normal. Similar relationships are possible for rotational motions considering torques applied to the mirror.

*Mechanical transfer functions*

The transfer function $$H(\varOmega
								)$$ is determined by the specific setup of the suspension systems. The various resonances and features of the system can be represented with an expansion into *poles and zeros*:

6.30 $$\begin{aligned}
								H(\varOmega )= & {} \frac{\prod _{k=1}^{N_z} (\mathcal
								{Z}_{k} - \varOmega ^2)}{ M\prod _{j=1}^{N_p}(\mathcal {P}_j -
								\varOmega ^2)} \nonumber \\ \mathcal {P}_j= & {} \varOmega
								_{p,j}^2-\frac{\mathrm {i}\,\varOmega _{p,j}\varOmega }{Q_{p,j}},
								\quad \mathcal {Z}_k = \varOmega _{z,k}^2-\frac{\mathrm
								{i}\,\varOmega _{z,k}\varOmega }{Q_{z,k}}
								\end{aligned}$$

where *M* is the mass of the mirror in kg, $$\{\varOmega
								_{p/z,i}\}_{i=1}^{N_{p/z}}$$ is a set of frequencies for each pole and zero and $$\{Q_{p/z,i}\}_{i=1}^{N_{p/z}}$$ the respective quality factors. When the frequencies of interest (signal frequencies) are much higher than any pole or zero frequency, $$\varOmega \gg
								\varOmega _{p/z}$$, we can assume a *free mass*, $$N_z =
								0$$ and one pole of infinite Q at 0 Hz:

6.31 $$\begin{aligned}
								H(\varOmega ) = -\frac{1}{M \varOmega ^2}.
								\end{aligned}$$

*Approximations for radiation pressure*

With the mirror position change being proportional to the laser power, $$\delta z \propto
								P$$, the problem is non-linear in terms of the complex field amplitudes. Solving such a problem in a complex interferometer setup is challenging and not possible using the methods outlined in Sect. 2, as the frequency domain model is assuming a linear system. However, for gravitational-wave detectors we can make some assumptions about the system:

- the motion of any optic is small, $$|\delta z| \ll \lambda
											$$, when the interferometer is controlled and well-behaved, and we can linearise equations in $$\delta z$$,
- any high-frequency fluctuations in the beam are negligible due to $$H(\varOmega ) \propto 1/\varOmega
											^2$$ and we ignore the effects of RF sidebands on the optics,
- any low-frequency fluctuations are very small, such that the magnitude of any sidebands is much less than the magnitude of its carrier field, which allows us to identify a well defined carrier field in our calculations.

These are all valid assumptions for gravitational-wave detectors once they are operating in a steady state and have well controlled optics. For a single carrier with amplitude $$E_0$$ and frequency $$\omega
								_0$$ and noise sidebands at frequency $$\varOmega
								$$, the incident field on a suspended mirror is:

6.32 $$\begin{aligned}
								E_i= & {} (E_0 + q_+e^{\mathrm {i}\,\varOmega
								t}+q_-e^{-\mathrm {i}\,\varOmega t}) e^{\mathrm {i}\,\omega _0 t} +
								\mathrm{c.c}. \end{aligned}$$

As with the approximations listed above we can assume $$|q_\pm | \ll
								|E_0|$$ and $$\omega _0 \gg
								\varOmega $$. The fluctuation in the beam power is then given by:

6.33 $$\begin{aligned}
								P(\varOmega )= & {} q_+ E^*_0 + q_-^*E_0
								\end{aligned}$$

where we only consider sideband-carrier product terms and those with a frequency $$\varOmega
								$$. Substituting the fluctuating power 6.33 into the radiation pressure force 6.28 to compute the displacement 6.29 the motion of the mirror can be found. The amplitude of the motion at frequency $$\varOmega
								$$ induced via radiation pressure for a perfectly reflective, free-mass mirror is

6.34 $$\begin{aligned}
								\delta z = -\frac{2}{Mc\varOmega ^2} \left( q_+ E^*_{0} +
								q_-^*E_{0}\right) . \end{aligned}$$

Such a moving mirror, as discussed in Sect. 3.2, creates phase modulation sidebands around any carrier that is reflected from it. The reflected field, using Eq. (3.10), is:

6.35 $$\begin{aligned}
								E_r= E_i\left( 1+\frac{\mathrm {i}\,k_0}{2}\Bigl (\delta z^{+}
								e^{-\mathrm {i}\,\varOmega t} + \delta z^{-} e^{\mathrm
								{i}\,\varOmega t}\Bigr )\right) e^{\mathrm {i}\,\omega _0 t},
								\end{aligned}$$

where to keep notation simpler, $$\delta
								z^{+}\equiv \delta z(+\varOmega )$$ and $$\delta z^{-}
								\equiv \delta z(-\varOmega )^*$$.

Take the simple example of vacuum noise and a single carrier, with amplitude $$E_0$$, incident on a free mass mirror of mass *M* and calculate the noise after being reflected. The amplitude of the reflected upper and lower noise sidebands, $$q_{r,\pm
								}$$, using Eqs. (6.29) and (6.35), are:

6.36 $$\begin{aligned}
								q_{r,\pm }= & {} v_\pm + \mathrm {i}\,E_0 k_0\frac{\delta
								z^\pm }{2}, \nonumber \\= & {} v_\pm - \mathrm {i}\,E_0
								k_0\frac{v_{\pm } E^*_0 + v_{\mp }^*E_0}{Mc\varOmega ^2}.
								\end{aligned}$$

where $$v_\pm
								$$ are the incident pure vacuum noise sidebands. (6.36) shows that the reflected upper and lower noise sidebands are now a mix of the incident upper and lower sidebands, i.e., after reflection they are correlated. There is additional phase noise present, and it scales as $$\propto
								|E_0|/\varOmega ^2$$. Thus, this noise is relevant at low frequencies and when the beam power to mass ratio is significant.

The noise PSD for the reflected beam is computed using Eq. (6.16). This requires computing the various covariance and auto-covariances of the reflected noise sidebands 6.36 and their beating with the carrier field:

6.37 $$\begin{aligned}
								\langle q_{r,\pm }q_{r,\mp }'\rangle= & {} -\dfrac{\hbar
								\omega _0 E_0^2}{M\varOmega ^2 c}\left( k_0 + \dfrac{|E_0|^2
								k_0^2}{M \varOmega ^2 c} \right)
								\end{aligned}$$

6.38 $$\begin{aligned}
								\langle q_{r,\pm }q_{r,\pm }'^*\rangle= & {} \dfrac{\hbar
								(\omega _0\pm \varOmega )}{2} + \dfrac{\hbar \omega _0 |E_0|^4
								k_0^2}{M^2 c^2 \varOmega ^4}.
								\end{aligned}$$

To simplify the above we will also assume the carrier has zero phase, $$E_0^*=
								E_0$$ and that $$E_0 =
								\sqrt{P_0}$$. The power noise PSD using Eq. (6.16) is then

6.39 $$\begin{aligned}
								S_I(\varOmega )= & {} 2P_0\bigl (\bigl<
								q_{r,+}q'^*_{r,+} \bigr> + \bigl< q_{r,-}q'^*_{r,-}
								\bigr>\bigr ) + 2P_0\bigl<q_{r,-}q'_{r,+}
								\bigr>^*+ 2P_0^*\bigl <q_{r,+}q'_{r,-} \bigr
								> \nonumber \\= & {} 2 \hbar \omega _0 P_0.
								\end{aligned}$$

Thus the noise is still just a flat shot noise limit, as expected. However, this only shows the amplitude noise in the beam, not any phase noise. To compute the phase quadrature the local oscillator must have an additional $$\pi
								/2$$ phase relative to the sidebands. Experimentally this could be achieved using the balanaced homodyne readout as mentioned in previous sections. Here we can simply add an additional phase to the carrier to the beam *after* reflection, i.e., compute the PSD of a power fluctuation

6.40 $$\begin{aligned}
								P(\varOmega ) = q_+ E^*_0 e^{-\mathrm {i}\,\phi } + q_-^*E_0
								e^{\mathrm {i}\,\phi } \end{aligned}$$

where $$\phi
								$$ is our additional homodyne phase. Computing the PSD of this with $$\phi =\pi
								/2$$ to compute the phase fluctuations we see:

6.41 $$\begin{aligned}
								S_\phi (\varOmega )= & {} 2 P_0 \hbar \omega _0 +
								\dfrac{8\hbar P_0^3 \omega _0 k_0^2}{M^2 c^2 \varOmega ^4}.
								\end{aligned}$$

This shows a flat shot noise fluctuation plus additional phase noise due to the vacuum noise perturbing the mirror. In the limit of an infinitely heavy mirror we can see this radiation pressure noise is removed and we are left with the vacuum noise fluctuations in phase. It is these phase fluctuations that are converted from phase to amplitude noise at the Michelson dark port that then lead to quantum noise limited sensitivity of the detector at the output photodiode.

### Semi-classical Schottky shot-noise formula

Shot noise historically has been described as the noise arising from the statistical distribution of electrons in photo detectors. The Schottky formula for the (single-sided) power spectral density of the fluctuation of the photocurrent for a given mean current $\bar{I}$ is:

6.42 $$\begin{aligned}
								S_I(f) =2\,e\,\bar{I}, \end{aligned}$$

with *e* the electron charge. Here $$S_X
								(f)$$ denotes the single-sided power spectral density of *X* over the Fourier frequency *f*. The link between (mean) photocurrent $$\bar{I}$$ and (mean) light power $$\bar{P}$$ is given by the relation:

6.43 $$\begin{aligned}
								\bar{I}= e N= \frac{e~\eta ~\lambda }{\hbar 2 \pi c} \bar{P},
								\end{aligned}$$

with *N* as the number of photons and $$\eta
								$$ the quantum efficiency of the diode. Instead of Planck’s constant we write $$\hbar \,2\,\pi
								$$ to avoid confusion with the typical use of *h*(*t*) for the strain of a gravitational wave. We can now give a power spectral density for the fluctuations of the photocurrent:

6.44 $$\begin{aligned}
								S_P(f)=2\,\frac{2\pi \,\hbar \,c~}{\lambda }\bar{P}=2\,\hbar
								\,\omega _0\,\bar{P}. \end{aligned}$$

As stated above this equation estimates the shot noise correctly when the interferometer does not contain any non-linear effects or squeezed input fields.

### Optical springs

Optical springs are a result of an optomechanical feedback process that couples the intensity fluctuations in an optical field and the motion of a suspended mirror being restored by gravity. In the following we show how this feedback process introduces a force analogous to that of a damped spring with resonance frequency and damping coefficient defined by the optical properties of the interferometer and mechanical properties of the suspended mirrors, using the properties of the optomechanical coupling, introduced in the previous section.

At high circulating powers optical springs can significantly alter the behaviour of a suspended interferometer. Layouts such as optical bars (Braginsky et al. 1997) and techniques such as detuned signal-recycling (Buonanno and Chen 2002) use optical springs to improve the sensitivity of detectors (Rehbein et al. 2008). Due to their potential impact on current and future generations of gravitational-wave detectors, there have been several efforts to experimentally characterise their behaviour (Virgilio et al. 2006; Sheard et al. 2004; Corbitt et al. 2006). In this article only the longitudinal motion of a mirror along the axis of the optical beam axis is considered. However, rotational optical springs from torques (Sidles and Sigg 2006; Hirose et al. 2010; Dooley et al. 2013) or couplings to higher-order elastic vibrational modes of the mirror, known as *parametric instabilities* (Braginsky et al. 2001; Evans et al. 2015; Brown 2016), also exist and can pose significant challenges for controlling the interferometer at high laser powers.

*Adiabatic optical spring*

A Fabry–Perot cavity with a suspended end mirror is the simplest system which can feedback the sidebands created to the mirror. Firstly the case when the mirror is moving slowly compared to the round-trip time of the cavity is considered. In this situation the optical response to a mirror moving is effectively instantaneous throughout the interferometer. As shown in Eq. (5.1), the power circulating in a Fabry–Perot cavity, hence the power incident on the suspended mirror, as a function of a cavity length change *z* in meters is

6.45 $$\begin{aligned}
								P_c(z) = \dfrac{P_0\,T_1}{1 + R_1 R_2 - 2 r_1 r_2 \cos (2k z)}
								\end{aligned}$$

and shown in Fig. 43. As the radiation pressure force is $$\propto
								P_c$$ the force varies with respect to the end mirror’s position. A position dependent force is the definition of a spring constant, thus for our optical spring we find:

6.46 $$\begin{aligned}
								k _{opt} = -\dfrac{\mathop {}\!\mathrm {d}F(z)}{\mathop {}\!\mathrm
								{d}z} = \dfrac{\mathop {}\!\mathrm {d}}{\mathop {}\!\mathrm {d}z}
								\left[ \dfrac{-2 P_c(z)}{c}\right] = \dfrac{-8 P_0 r_1 r_2 k T_1
								\sin (2k z)}{c(1+R_1 R_2-2r_1 r_2 \cos (2 k z))^2}.
								\end{aligned}$$

Plotting the $$k_{opt}$$ in Fig. 43, when the cavity is perfectly resonant for the carrier field there is no optical spring, for positive $$\delta
								z$$ we have a restoring force, $$k_{opt}
								< 0$$, and anti-restoring force, $$k_{opt}
								> 0$$, with negative detunings.

**Fig. 43:**
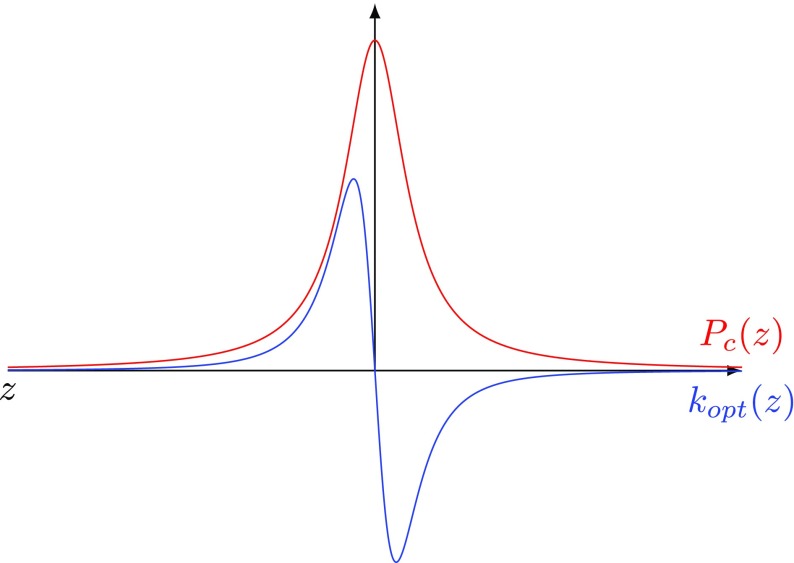
Illustrative example of the circulating power (*red*) in a Fabry–Perot cavity. The *blue line* shows the spring constant (*blue*). Not to scale

**Fig. 44:**
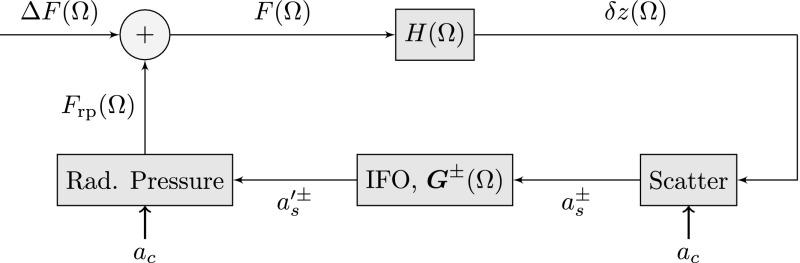
A generic view of a closed-loop optomechanical transfer function for a suspended mirror with mechanical susceptibility $$H(\varOmega
											)$$. Due to some motion $$\delta z(\varOmega
											)$$ of a mirror the light is scattered from the carrier. The IFO plant describes the optical transfer function of the sidebands propagating through the interferometer and back to the mirror in question. The the interference of these then creates some radiation pressure force which is fed back into the mirror. Here $${a}_c$$ is the carrier field at the mirror in question

*Full steady state optical spring*

To compute the full response of a suspended mirror we have to consider the propagation and transformation of the sidebands generated by a moving mirror through the rest of the interferometer and finally back to the mirror in question. This process of scattering and feedback is represented by the block diagram in Fig. 44. A sinusoidal force $$F(\varOmega
								)$$ is acting on a mirror with mechanical susceptibility $$H(\varOmega
								)$$; the motion $$\delta
								z(\varOmega )$$ combined with the incident carrier field $${a}_c$$ scatters light into the upper, $${a}_s^+$$, and lower, $${a}_s^-$$, sidebands leaving the mirror. The IFO plant, $$ G(\pm \varOmega
								) \equiv G^\pm $$, is the optical transfer functions for either the upper or lower sidebands leaving the mirror to those returning to it. Lastly the incident upper and lower sidebands, $${a}'^\pm _s =
								G^\pm {a}_s^\pm $$, are combined again with the carrier field to compute the radiation pressure force $$F_\mathrm
								{rp}(\varOmega )$$ along with an external excitation $$\varDelta
								F(\varOmega )$$ to feedback into the mirror.

To illustrate this in more detail a single carrier with a pair of sidebands describing some amplitude modulation at a frequency $$\varOmega
								$$ will be considered. This optical field is incident on a free mass mirror with a high reflectivity, $$R = r^2\approx
								1$$. The incident field, $$a^{'\pm
								}_s$$, and reflected, $$a^{\pm
								}_s$$, fields using the small phase modulation approximation 3.10 are:

6.47 $$\begin{aligned}
								a^{'\pm }_s&= G^\pm a_s^\pm ,
								\end{aligned}$$

6.48 $$\begin{aligned}
								a^\pm _s&= (a'^\pm _s + \mathrm {i}\,k \,\delta z^\pm a_c)
								\end{aligned}$$

Solving for the incident field using both 6.47 and 6.48 we find:

6.49 $$\begin{aligned}
								a^{'\pm }_s = \dfrac{\mathrm {i}\,k G^\pm \,\delta z^\pm a_c}{1 -
								G^\pm }. \end{aligned}$$

To compute the total force acting on the mirror we must consider the intensity fluctuations of all incident and reflected beams, as the total momentum of the beams and the suspended mirror must be conserved (Meystre et al. 1985). In this case there are only beams on one side of the mirror, the intensity fluctuations of the reflected beams are nearly the same as those of the incident beam as the mirror imprints only phase modulation and $$R\approx
								1$$. The total force the mirror experiences is then:

6.50 $$\begin{aligned}
								F_\mathrm {rp}(\varOmega ) \approx \dfrac{4}{c}\left[ a_s^{'+}a_c^*+
								a_s^{'-*} a_c\right] . \end{aligned}$$

Now, considering the incoming beams 6.49, the force is:

6.51 $$\begin{aligned}
								F_\mathrm {rp}(\varOmega )&\approx \dfrac{4\mathrm {i}\,k
								P_c}{c} \left[ \dfrac{G^+ - G^{-*}}{ 1 - G^+ - G^{-*} + G^+ G^{-*}}
								\right] \,\delta z(\varOmega ) \equiv \kappa (\varOmega ) \,\delta
								z(\varOmega ). \end{aligned}$$

This shows that the radiation pressure force is linearly dependent on $$\delta
								z$$ for an arbitrary interferometer layout described by $$G^\pm
								$$. The complex valued scaling factor, $$\kappa
								(\varOmega )$$, represents how the dynamic response of the suspended mirror is altered. Those terms independent of $$\varOmega
								$$ define the stiffness of the optical spring. Terms $$\propto
								\varOmega $$ describe any damping, $$c_\mathrm
								{opt}$$ being the optical damping coefficient:

6.52 $$\begin{aligned}
								\kappa (\varOmega ) = k_\mathrm {opt} + \mathrm {i}\,\varOmega
								c_\mathrm {opt}(\varOmega ) + \mathcal {O}(\varOmega ^2).
								\end{aligned}$$

Higher order terms can also be significant, depending on the optical feedback, and can alter the inertial behaviour by introducing terms $$\propto
								\varOmega ^2$$. Such manipulation of the optomechanical coupling here can be exploited to improve the sensitivity of gravitational-wave detectors (Ma et al. 2014).

The above analysis is applicable in the case of a single optical field. If there are multiple optical fields of comparable amplitude, the sum of the multiple radiation pressure forces must be considered to compute the overall value of $$\kappa
								$$. The result (6.51) is also only applicable for optical fields with dominating radiation pressure on one single side of a near perfectly reflective mirror. The analytical calculation of other cases, such as, suspended beam splitters, multiple suspended optics, or multiple carrier frequencies with higher order spatial modes, can become very complicated. Tools such as Finesse take all these effects into account to ease studying such systems.

*Optical spring in a cavity*

The simplest case of an optical spring we can consider is that of an two-mirror optical cavity with a single suspended mirror. The optical spring constant for this setup can be determined using Eq. (6.51). Here $$G^\pm
								$$ are the optical transfer functions for the upper and lower sidebands through one round-trip of the cavity after being created at the suspended mirror. For this example we will take the input mirror to be fixed and the end mirror to be a suspended free mass. We determine $$G^\pm
								$$ by starting from where the sidebands are created at the suspended mirror, whose reflectivity is $${\approx }
								1$$. These then propagate along the cavity length *L* twice with a reflection from the fixed input mirror, with reflectivity $$r_1$$, before returning to the end mirror. In total the propagation is

6.53 $$\begin{aligned}
								G^\pm = r_1 e^{\mp \mathrm {i}\,2 \frac{\varOmega }{c}L} e^{\mathrm
								{i}\,2\phi }. \end{aligned}$$

Here $$\phi
								$$ is some detuning of the input mirror position. Substituting this into (6.51) we find:

6.54 $$\begin{aligned}
								\dfrac{G^+ - G^{-*}}{ 1 - G^+ - G^{-*} + G^+ G^{-*}} =
								\dfrac{\mathrm {i}\,2 r_1 e^{-\mathrm {i}\,2 \frac{\varOmega }{c}L}
								\sin (2\phi )}{1 + R_1 e^{-\mathrm {i}\,4 \frac{\varOmega }{c}L} - 2
								r_1 e^{-\mathrm {i}\,2 \frac{\varOmega }{c}L} \cos (2\phi )}
								\end{aligned}$$

and:

6.55 $$\begin{aligned}
								\kappa (\varOmega ) = -\dfrac{8 k P_c r_1 \sin (2\phi )}{c}
								\dfrac{e^{-\mathrm {i}\,2 \frac{\varOmega }{c}L}}{1 + R_1
								e^{-\mathrm {i}\,4 \frac{\varOmega }{c}L} - 2 r_1 e^{-\mathrm {i}\,2
								\frac{\varOmega }{c}L} \cos (2\phi )} .
								\end{aligned}$$

When the cavity is on resonance, $$\phi
								=0$$, we see no optical spring, as expected. Likewise, in the DC limit $$\varOmega
								\rightarrow 0$$, we find an agreement with (6.45). Shown in Fig. 45 is and example of a force-to-displacement transfer function for the suspended end mirror when a force is applied.

### Finesse examples

#### Optical spring

A simple example for an optical spring in a two mirror cavity with suspended mirrors. The cavity is slightly detuned which is required for creating the spring. The output is the motion of a mirror while it is excited with a force of constant amplitude. The resonance feature shown in Fig. 46 is the result of the optomechanical coupling of the mirror with the cavity field.

**Fig. 45:**
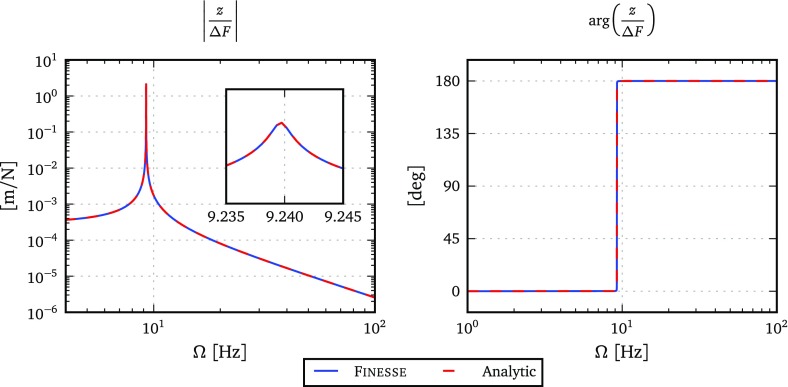
Analytical versus numerical (Finesse) comparison of an optical spring. This is for a fixed input field and suspended (free-mass) end mirror. A force is applied to the end mirror and shown is the force-to-displacement of the end mirror transfer function. *Inset plot* shows zoomed region around the peak which shows a good agreement with the peak shape and position

**Finesse input file for ‘optical spring’** 
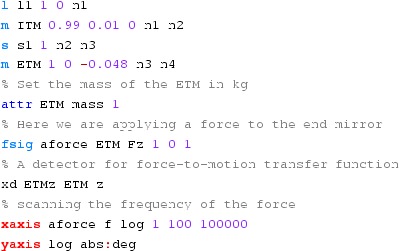


**Fig. 46:**
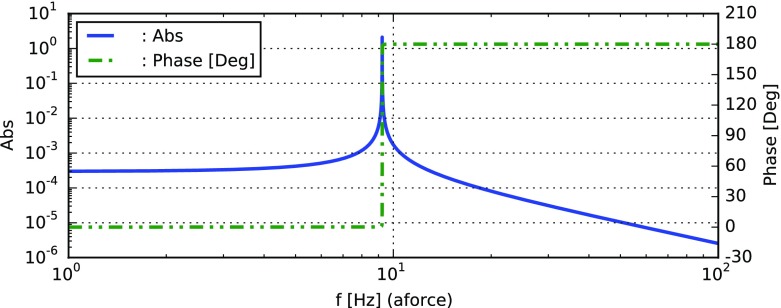
Finesse example: Fabry–Perot cavity with an optical spring. The two traces show the amplitude and phase of the mechanical response of one cavity mirror to an exciting force. The resonance feature close to 10 Hz is the result of the optomechanical coupling of the mirror with the cavity field

**Fig. 47:**
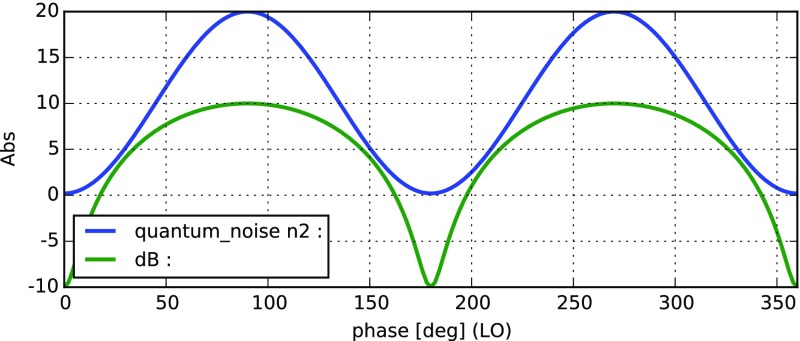
Finesse example: Homodyne detector with a squeezed light input. The *blue trace* shows the quantum noise in units of $$\hbar f$$ with a range of 0.2–20, compared to a quantum noise of $$2 \hbar
												f$$ for an unsqueezed source. The *green trace* instead has the units ‘dB’ and shows an effective squeezing level, inferred from the detected quantum noise

#### Homodyne detector and squeezed light

A laser and a squeezed light source are mixed with a beam splitter and then detected with a homodyne detector. The nominal quantum noise of an un-squeezed light field in the units of the blue trace are $2 \hbar f$. The squeezing level of the squeezed light source is 10 dB, which means that the noise in one quadrature is 10 times lower than this whereas the other quadrature should be 10 times higher. With the phase of the local oscillator the homodyne detector can be tuned to measure the different quadratures. The green trace shows a computation of an effective squeezing level from the detected quantum noise using the Schottky equation (Fig. 47).

**Finesse input file for ‘homodyne detector and squeezed light’** 
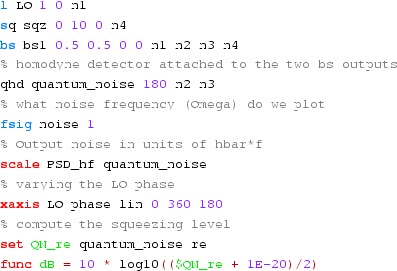


#### Quantum-noise limited interferometer sensitivity

**Fig. 48:**
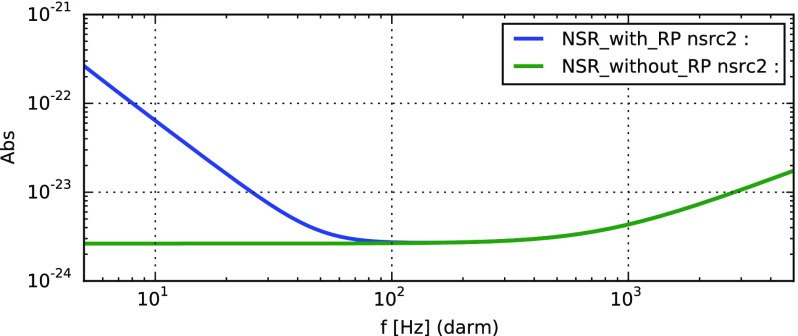
Finesse example: quantum limited sensitivity of a simplified model of an Advanced LIGO interferometer. The interferometer setup is similar to a broadband RSE configuration of LIGO. The *blue trace* shows the full quantum-noise-limited sensitivity. For comparison the *green trace* shows the shot-noise-limited sensitivity

This example shows the quantum-noise limited sensitivity of an advanced detectors. See “Appendix B” for the optical layout of the detector and Sect. 8.12 for more details about the interferometer operation. The model is loosely based on the Advanced LIGO design file and thus we expect to see the peak sensitivity around 100 Hz at a sensitivity of about $10^{-23}/\sqrt{\mathrm{Hz}}$. We can see the both the ‘qnoised’ and ‘qshot’ detectors agree at high frequencies, where the sensitivity is purely limited by shot noise. At low frequencies the two traces differ because only ‘qnoised’ takes into account the radiation pressure effects (Fig. 48).

The Finesse input file for this example is more complex than for other examples because it contains a more complex interferometer setup and uses relatively advanced concepts such as setting mechanical transfer function. See “Appendix A” for more information on Finesse and where to find the documentation, such as the syntax reference, required to follow this example.

**Finesse input file for ‘quantum-limited interferometer sensitivity’** 
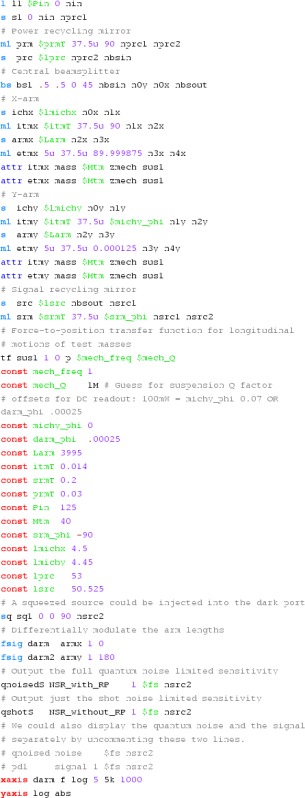

## Advancing the interferometer layout

The first generation of interferometric gravitational-wave detectors was limited in the upper-frequency band by shot noise, one manifestation of the quantum noise of the laser light, see Sect. 6. We can improve the ratio between gravitational-wave signal and shot noise in several ways, for example, by increasing the arms’ length or by increasing the injected laser power. The lengths of the arms is typically limited by the associated costs of the building the infrastructure. For example, due to the curvature of the Earth’s surface, a 40 km long interferometer arm would require a trench or tunnel approximately 30 m below the surface in the middle of the arm.

High-power lasers are used; however do not come near the power levels required for the anticipated sensitivity. For example, the design sensitivity of Advanced LIGO requires a light power or several hundred kilowatts in the interferometer arms. The Advanced LIGO laser can provide up to 200 W of power, and represents a state of the art system (for a CW laser with the required stability in frequency, amplitude and beam profile) (Kwee et al. 2012).

In order to increase the laser power inside the arms further we can utilise the concept of resonant light enhancement in the Fabry–Perot cavity: so-called advanced interferometer topologies are created by introducing optical cavities to the Michelson interferometer. In the following we will briefly introduce the most common concepts, which are used by modern gravitational-wave detectors today.

We have shown in Sect. 5.2 how the dark fringe operating point allows to maximise the throughput of differential signals (with respect to common mode noise), using the sideband picture. Similarly we can compute the transfer functions of the signal sidebands to illustrate the concepts behind the advanced interferometer layout. The motivation for all the advanced concepts shown below is the improvement of the ratio between signal and shot noise. However, we will ignore here the detailed computation of the shot noise and quantum noise discussed in Sect. 6. Instead we will compute only the transfer functions of the signal to the photo detector using the sideband picture. We will ignore radiation pressure noise and shot-noise contributions from any light field but the local oscillator. Thus the amplitude of the signal sidebands in the detection port give a good figure of merit for the shot-noise limited sensitivity of the detector.

### Michelson interferometers with power recycling

**Fig. 49:**
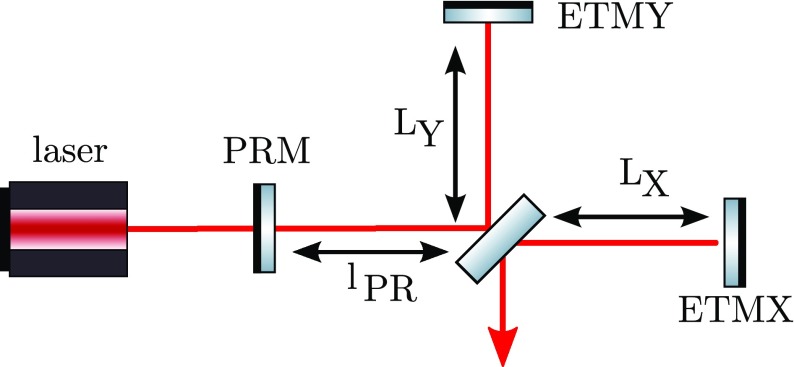
Optical layout of a Michelson interferometer with arm power recycling

**Fig. 50:**
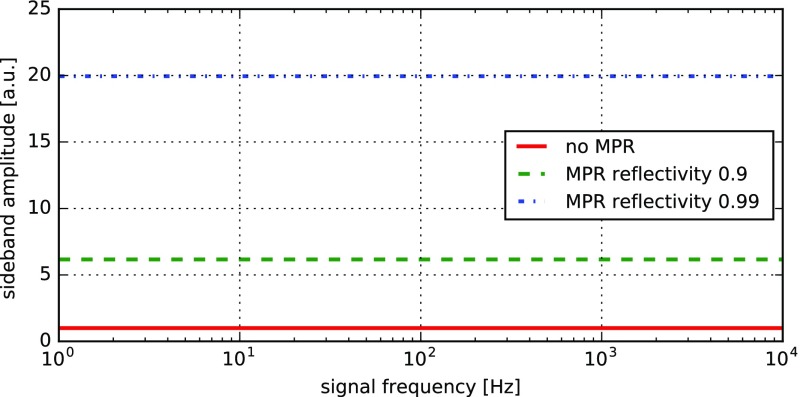
This graph shows the signal sideband amplitude for a differential arm length change, as detected in the anti-symmetric output port, as a function of the frequency of the signal. The *solid red trace* at an amplitude of 1 refers to the case without power recycling. The other two traces show the increased amplitude for different reflectivity’s of the power-recoiling mirror. Compare this plot also with Figs. 52 and 54

The Michelson interferometer, when held on the dark fringe and ignoring internal losses, reflects all the incoming light back into the laser port; seen from the laser it acts like a highly reflective mirror. It was soon realised we can utilise this fact to increase the light power inside the interferometer: an additional mirror inter the input port, the so-called power-recycling mirror (PRM), will generate an optical cavity with the Michelson interferometer acting as a second ‘mirror’. This scheme which is now called *power recycling* was first proposed in 1983 independently by Billing et al. (1983) and Drever et al. (1983). The newly formed cavity is often called *power-recycling cavity*. The optical layout of a power-recycled Michelson interferometer is shown in Fig. 49. Figure 50 shows the amplitude of signal sidebands for different levels of power recycling, as a function of the frequency of the signal. We will compare this to similar plots for other techniques described below.

As we have discussed in Sect. 5.1, the power circulating inside a cavity can be much higher than the injected light power. The power enhancement is given by the finesse of the cavity which is given by the optical losses in the interferometer and the reflectivity of the power-recycling mirror. When the losses inside the Michelson interferometer are negligible the cavity formed by the Michelson and the power-recycling mirror is over-coupled and the power enhancement in the interferometer arms, also called *power-recycling gain* computes as

7.1 $$\begin{aligned}
								G_\mathrm{PR}=\frac{4}{T_\mathrm{PRM}}\approx \frac{2\mathcal
								{F}}{\pi } \end{aligned}$$

with $$\mathcal
								{F}$$ the finesse of the power-recycling cavity.

When the optical losses can not be ignored the maximum power-recycling gain can be reached by impedance matching, i.e., setting the transmission of the power-recycling mirror equal to the round trip losses of the power-recycling cavity and the gain becomes

7.2 $$\begin{aligned}
								G_\mathrm{PR}=\frac{1}{T_\mathrm{PRM}}\approx \frac{\mathcal
								{F}}{\pi } \end{aligned}$$

The power in the signal sidebands is proportional to the carrier power and thus scales with the power-recycling gain as well. The amplitudes plotted in Fig. 50 thus show values of $$\sqrt{4/0.1}\approx
								6.32$$ and $$\sqrt{4/0.01}=20$$.

Power-recycling has further advantages: the cavity effect can be used to reduce beam jitter and to filter laser frequency noise. The disadvantage is that another mirror position needs to be carefully maintained by a feedback control system. In addition, the increase in circulating power also increases the laser power within the substrate of the beam splitter which can cause thermal distortions leading to higher-optical losses. In practise this often limits the achievable power-recycling gain.

**Fig. 51:**
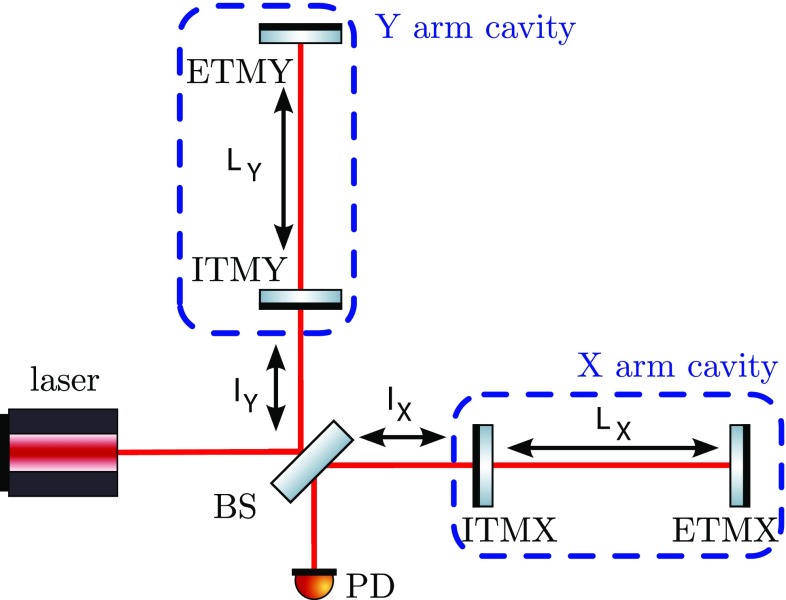
Optical layout of a Michelson interferometer with arm cavities

### Michelson interferometers with arm cavities

Another way to employ cavities to enhance the light power circulating in the interferometer arms is to place optical cavities into these arms, as so-called *arm cavities*, as shown in Fig. 51. This optical configuration sometimes referred to as *Fabry–Perot–Michelson interferometer*. Similar to power-recycling the finesse of the cavity determines the enhancement of the light power.

**Fig. 52:**
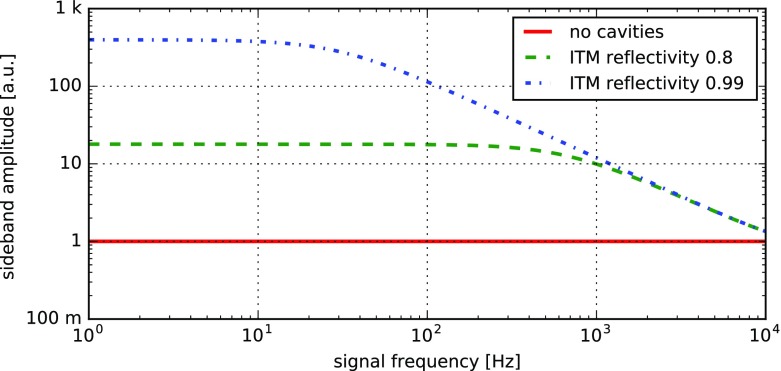
This graph shows the signal sideband amplitude for a Fabry–Perot–Michelson interferometer. The signal is a differential arm length change detected in the anti-symmetric output port, as a function of the frequency of the signal. The *solid red trace* at an amplitude of 1 refers to the case without arm cavities. The other two traces show the increased amplitude for different reflectivities of the cavities’ input mirrors. Compare this also with Figs. 50 and 54

The arm cavities have another effect on the detector sensitivity: they affect not only the power of the circulating carrier field, but also that sidebands generated by a length change. This results in a further increase of the sensitivity for signals with a frequency within the linewidth of the arm cavities but to a decrease in sensitivity regarding signals with frequencies that fall outside the linewidth of the cavities. This can be shown again very clearly with the sideband amplitudes detected at the interferometer output as shown in Fig. 52. We can compare this results to the power-recycling case (Fig. 50): when the reflectivity of the PRM and ITMs is set to $R=0.99$, the expected gain for the carrier field inside the cavities must be the same and equal to 400, assuming an over-coupled case. At low frequencies the signal sidebands will experience the same enhancement, namely by a factor of 400 in power. Thus the total enhancement for the signal sidebands in the Michelson with arm cavities is 16 000, which gives the amplitude of 400 shown for sideband amplitude in Fig. 52. Therefore the arm cavities also change the detector response function in a way that limits the possible sensitivity increase.

The limited bandwidth of the arm cavities is a disadvantage when compared to the power-recycling technique; however, the arm cavities have the significant advantage of not increasing the light power in the beam splitter substrate. In practise the two techniques are commonly used together, with the finesse of the arm cavities and the reflectivity of the power-recycling mirror the result of a trade-off analysis between the bandwidth reduction of the arm cavities and the light power increase in the beam splitter substrate. Such an optical layout is also called *power-recycled Fabry–Perot–Michelson interferometer*.

### Signal recycling, dual recycling and resonant sideband extraction

**Fig. 53:**
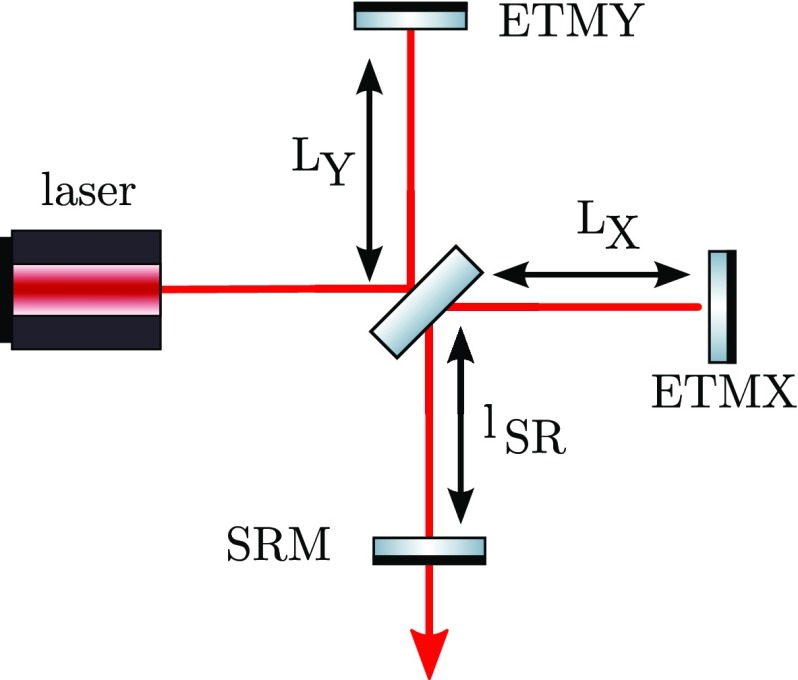
Optical layout of a Michelson interferometer with signal recycling

Soon after the development of power recycling in which an additional mirror is used to ‘recycle’ the laser light leaving the Michelson interferometer through the symmetric port, Brian Meers recognised that it would be of interest to employ a similar technique in the anti-symmetric port. In the ideal Michelson interferometer on the dark fringe, the carrier light and the signal sidebands become separated at the central beam splitter and leave the interferometer though different ports. Meers (1988) suggested the addition of a *signal-recycling mirror* at the anti-symmetric port, to form a signal-recycling cavity with the Michelson interferometer. In a similar manner to the power-recycling cavity the signal-recycling cavity could resonantly enhance the light circulating within, i.e., the signal sidebands. The optical layout of a signal-recycled Michelson interferometer is shown in Fig. 53.

It is somewhat counterintuitive that placing a highly-reflective mirror in front of the photo detector would increase the power detected on the same photo detector. This is because the signal sidebands are created within the interferometer, and thus within the signal recycling cavity, by a parametric effect, in which light is transferred from a much larger reservoir, the carrier field. Gerhard Heinzel provides, in Appendix D of his thesis (Heinzel 1999), a clear and compact mathematical overview of a two-mirror cavity including this effect.

When both recycling techniques are used together, power recycling for enhancing the carrier power and signal recycling for increasing the signal interaction time, the combination of the two methods is called *dual recycling*. It was actually the concept of dual recycling which Meers (1988) proposed, and this was demonstrated first as a table-top experiment by the Glasgow group in 1991 (Strain and Meers 1991).

**Fig. 54:**
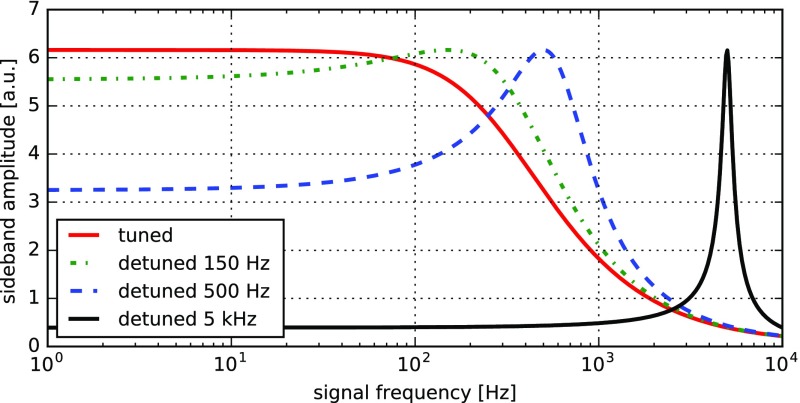
This graph shows the signal sideband amplitude for a Michelson interferometer with different signal-recycling configurations. For all 4 traces the reflectivity of the signal-recycling mirror was set to $R=0.9$, the interferometer arm length is 4 km. The *red trace* shows the *tuned* case in which the signal-recycling cavity is resonant for the carrier light and thus maximises signals around DC. The other *red traces* show different detunings, microscopic offsets to the longitudinal positions of the signal-recalling mirror. The maximum amplitude and bandwidth of the trace is the same in all four cases, just the frequency of the peak sensitivity is shifted by the detuning. Compare this the plots for arm cavities in Fig. 52 and power recycling, Fig. 50

The combination of arm cavities and a signal-recycling mirror is sometimes also called *resonant sideband extraction* (Mizuno et al. 1993). The difference between signal-recycling and resonant sideband extraction is that in the latter case the arm cavities have a very high finesse and the signal-recycling mirror is tuned to or near the anti-resonant operating point, thus effectively increasing the bandwidth of the detector for the signal sidebands. An analysis of the different techniques can be found in the thesis of Mizuno (1995). It is interesting to note that for all variants of the signal recycling the total integrated gain remains constant. For example, the areas under curves for the different detunings shown in Fig. 54 are constant.^10^ This means that signal-recycling is used to *shape* the response function of the detector with respect to the signal-to-shot-noise ratio.

The main interferometer of an Advanced LIGO detector is based on a Michelson interferometer with arm cavities plus power and signal recycling. This configuration is most commonly called *dual-recycled Fabry–Perot–Michelson interferometer* even though the signal recycling mirror is here used in the resonant sideband extraction mode, see Fig. 130 for a schematic of this layout.

**Fig. 55:**
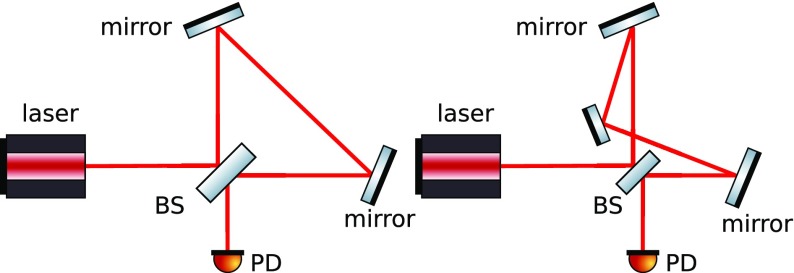
The *left sketch* shows a typical layout of the original Sagnac interferometer: similar to the Michelson interferometer the injected light is split and recombined at a central beam splitter. However, unlike the Michelson the Sagnac has not two different interferometer arms, but the two split beams travel along the same path, in different direction. This makes the Sagnac interferometer insensitive to the actual path length, instead is sensitivity to rotation of the whole interferometer. The *sketch* on the *right* shows a so-called *zero-area* Sagnac interferometer: an additional mirror is used so that the beam path is folded reducing the effective circulated area (see text)

### Sagnac interferometer

**Fig. 56:**
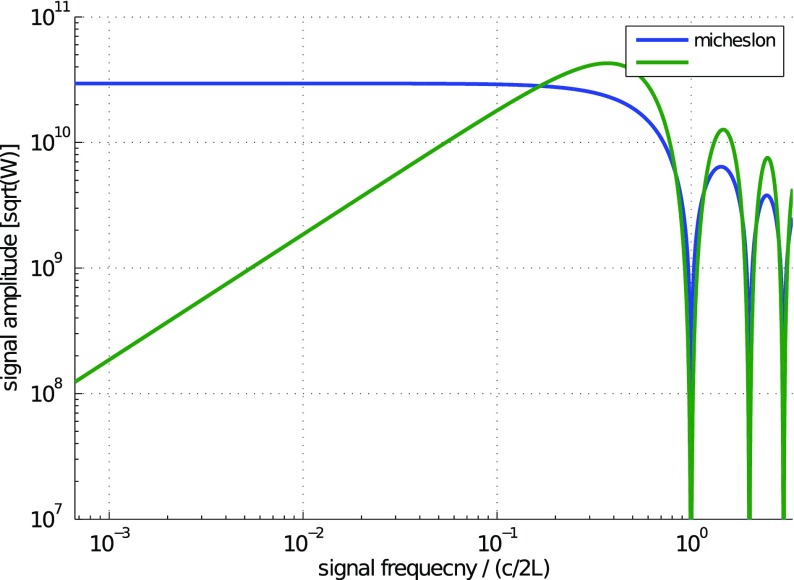
Transfer function of a Michelson and Sagnac interferometer for a gravitational-wave signal to the main output channel. It can be seen that the Sagnac response falls off for lower frequencies and reached twice the peak response; however at relatively large frequency, in the case of Advanced LIGO the peak would be at $$\hbox {f}=c/2L\approx
											37.5$$ kHz, i.e., above the measurement window

Another interferometer type which has a similar-looking optical layout to the Michelson interferometer is the *Sagnac* interferometer, see Fig. 55. Originally proposed by Sagnac (1913a, b) it became of interested to the gravitational-wave community as a possible alternative to the Michelson interferometer: in 1995 successful experimental tests of a zero-area Sagnac demonstrated a different mode of operation, in which it becomes insensitive to rotation but sensitive to mirror motion (Sun et al. 1996). Further investigations into the performance and technical limitations of a Sagnac interferometric gravitational-wave detector have been undertaken (Mizuno et al. 1997; Petrovichev et al. 1998; Beyersdorf et al. 2002) and the community interest was renewed after understanding that the Sagnac topology can be used as a speed-meter with the potential to suppress radiation pressure noise in future detectors (Chen 2003; Danilishin 2004; Wang et al. 2013; Voronchev et al. 2014; Danilishin et al. 2015; Fig. 56).

**Fig. 57:**
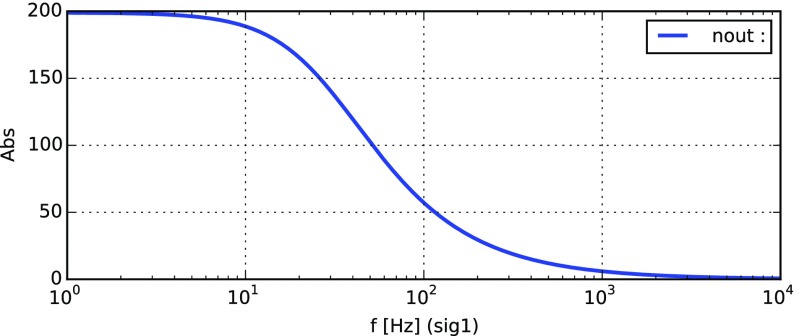
Finesse example: Michelson interferometer with arm cavities. The *trace* shows the signal sideband amplitude in the anti-symmetric port as a function of signal frequency

### Finesse examples

#### Michelson interferometer with arm cavities

This example shows how to setup a Michelson interferometer, tune it to the dark fringe and compute a transfer function from the differential length change to the output signal, using the sideband amplitude for simplicity (Fig. 57).

**Finesse input file for ‘Michelson interferometer with arm cavities’** 
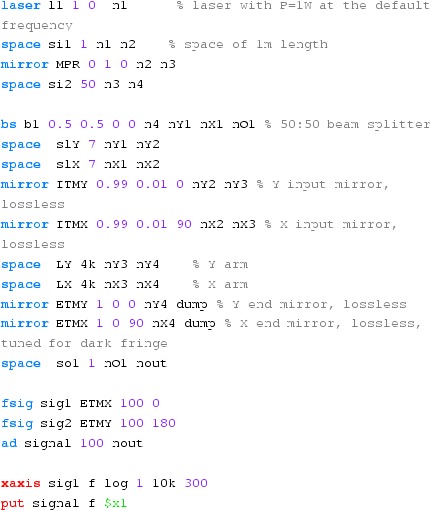


#### Michelson interferometer with signal recycling

**Fig. 58:**
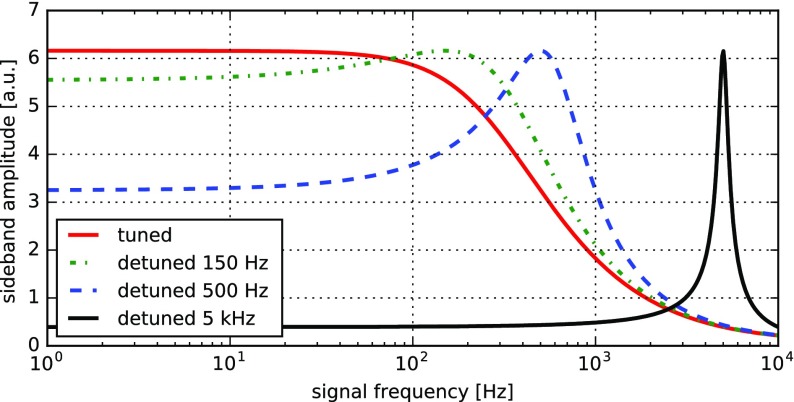
Finesse example: this graph shows the signal sideband amplitude for a Michelson interferometer with different signal-recycling configurations, see Fig. 54

This example recreates the plot shown in Fig. 54, the four traces show the transfer function for a Michelson interferometer with different signal recycling tunings (Fig. 58).

**Finesse input file for ‘Michelson with signal recycling’** 
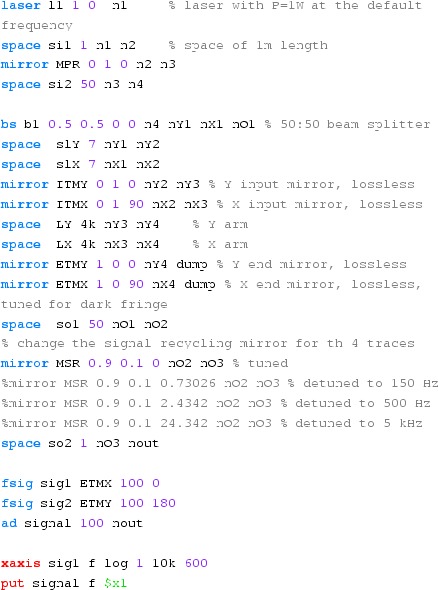


#### Sagnac interferometer

**Fig. 59:**
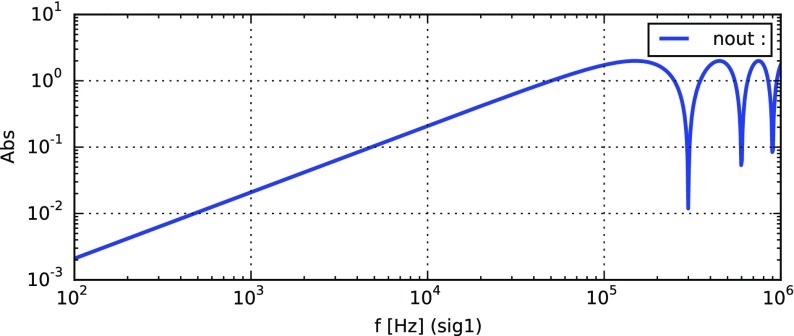
Finesse example: frequency response of a Sagnac interferometer: transfer function from differential mirror position change to signal sideband amplitude in the main output port

This example demonstrates how compute the frequency response of a simple Sagnac interferometer (Fig. 59).

**Finesse input file for Sagnac interferometer** 
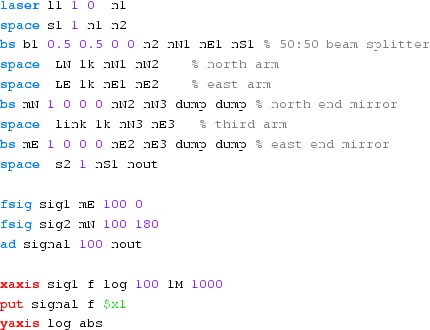


## Interferometric length sensing and control

In this section we introduce interferometers as length sensing devices. In particular, we explain how the Fabry–Perot interferometer and the Michelson interferometer can be used for high-precision measurements and that both require a careful control of the base length (which is to be measured) in order to yield their large sensitivity. In addition, we briefly introduce the general concepts of *error signals*, *transfer functions* and relevant elements of *control theory*, which are used to describe most essential features of length sensing and control.

In addition to sensing and controlling the distances between the components of an interferometer, alignment sensing and control is required for correct operation. While we do not deal with this aspect in detail, all of the ideas we develop for length sensing and control can be applied. The essential differences are that split photo-detectors are required to sense the relative angles of optical wavefronts, and control is be means of actuators that are able to adjust the angles of optical components. For an introduction to the essential ideas see Sect. 9.2 for an introduction to the relevant theory and Morrison et al. (1994), for details of a practical implementation.

### An overview of the control problem

A complete interferometer can have a large number of control loops for the various mirrors and beam splitters, their suspension systems and many other components, such as the laser, active vibration-isolation systems etc. For practical purposes these are usually divided into two broad classes that are often considered separately in the design process. These divisions reflect a degree of independence of the various categories of control and simplify the design process by allowing the problem to be split into a number of more easily tractable design elements.

The set of control loops that obtain signals from the detection of interference conditions or other properties of the light within the interferometer, and act on the major optical components of the interferometer to control those properties, is usually called *global control*. As an example, a description of the global control system of Virgo can be found in Arnaud et al. (2005). On the other hand, loops that sense properties associated with a single component, and act on that component are called *local* loops. A good example of local control is the system employed to damp the rigid-body modes of a mirror suspension, for an example from Advanced LIGO see Aston et al. (2012). By ‘cooling’ or quieting the motion of individual mirrors, the task faced by the global control system can be simplified. Further division of global interferometer control is frequently made between systems that control longitudinal degrees of freedom, i.e., relative positions of the mirrors and beam splitters along the direction of propagation of the light, and angular (alignment) control systems that are designed to stabilise the pointing of components.

Due to the presence of strong nonlinearity throughout much of the phase-space volume, there has been no attempt thus far to solve the multi-dimensional control problem as a whole. At least up to the present, the problem has been divided into several smaller parts, with methods developed to deal with the particular details of each facet of the system, and each stage of operation from completely uncontrolled to held at the operating point—a condition that is called ‘locked’.

This leads to yet another division: it is normal to separate the start-up phase i.e., the process called *acquisition of lock* from the stable running condition (‘in lock’). This split is motivated, at least in part, by the consideration that signal sizes can differ greatly between the two stages. During acquisition electronic signals tend to be large—corresponding to adjusting mirror positions by of order wavelengths, or more. By contrast, in operation the signals representing residual motion in the sensitive frequency band may be 12 or more orders of magnitude smaller than the wavelength of the light, to deliver the required measurement sensitivity.^11^

The jump in signal size between these two states is often dealt with by switching gain levels or even substituting large parts of the control system: starting with large-range but noisy methods for acquisition and switching to low-noise, but small-range controls for operation. A good example of a more substantial transition is the *arm length stabilization* (ALS) scheme in Advanced LIGO, which employs additional lasers, mirror coatings and interferometric methods to provide wavelength-range sensing during the acquisition phase (Evans et al. 2009). When the long cavities (see “Appendix B”) are locked, the control systems are switched over to the high-sensitivity, low-noise signals derived from the main interferometer systems.

During acquisition of lock, the instantaneous operating point frequently lies in a non-linear region of the control space. Several methods have been developed to cope with this problem.

The simplest approach, employed in the early interferometer prototypes, was to wait for a random co-incidence of suitable values to occur then to catch the system quickly enough to hold it in the desired state. As the complexity of the interferometer topologies increased, and with that the number of degrees of freedom, the probability of the desired state occurring in a conveniently short time became rare. This led to the development of more sophisticated techniques for the first long-baseline interferometers.

As a first step it was realised that digital logic, implemented directly in electronics or as software, could be employed to identify when one or more degrees of freedom happened to fall close to the desired operating point, and to activate the relevant control loop. This prevents false signals, frequently present in regions of phase space close to the desired operating point, being fed back to the actuators and perturbing the system. In Pound–Drever–Hall locking, for example, when the phase modulation sidebands pass through resonance in the cavity, the error signal has the opposite sign from that produced by a carrier resonance. The acquisition process can be improved by enabling the control system only when the circulating power within the cavity has exceeded the maximum possible power that a sideband can produce. By this means the control system is only activated close to the desired operating point, improving the chances of a successful lock.

A second way to improve matters is to linearise the behaviour, and so to increase the capture range: i.e., the volume of phase space within which the various control signals are valid. This improvement can be accomplished by normalising the relevant error signal according to some estimate of its slope, as measured by another signal such as the circulating optical power. As an example, the linear range in the Pound–Drever–Hall signal for locking a cavity may be extended by normalising with respect to the power within the cavity, as measured by probing the light transmitted by the cavity.

Another approach, is to arrange for the first locking to be in a region of phase space that is relatively smooth, compared to the region in phase space surrounding the final operating point. This was an enabling technique for GEO 600 when it was first operated in dual-recycled mode. It was found that by locking with signal recycling detuned by a few kHz, an initial lock was possible. The tuning was then stepped towards the target value, in steps chosen to be small enough to avoid perturbing the lock. By this means it was possible to reach a location in phase space which would essentially never have occurred by chance (Grote 2003).

After lock has been achieved by one or more of the above means, the control task is generally managed by linear control systems that may be analysed using standard *linear time invariant* (LTI) control theory. Two generic approaches have been employed with success. In one approach there is a set of separate single-input single output (SISO) controllers, one for each degree of freedom. The alternative is to deal with several degrees of freedom in a single multi-input multi-output (MIMO) controller. Recently, since the advent of computer-based, digital control systems, the MIMO approach has become much more practical than it would be if implemented in analogue electronics. An important difference between the SISO and MIMO approaches concerns how cross-coupling between the degrees of freedoms can dealt with.

Cross-coupling is commonly seen in both sensing and actuation, and considerable effort is needed to develop control systems that operate correctly in the presence of undesired mixing of signals. The main approaches to solving these problems with MIMO controllers is described in Sect. 8.7. Otherwise we discuss SISO controllers to provide illustrative examples of control in idealised interferometers where there is no mixing of degrees of freedom at the point of sensing or actuation.

We introduce standard terminology from control theory. In each control loop a point of reference is taken, called the *error point* at which we measure how the gain of the loop acts to suppress deviations from the desired operating point. Since a loop has no end, the selection of this point is somewhat arbitrary, but it is usually convenient to take the output from a photo-detector or its associated demodulator.

Likewise, we choose an actuation or *feedback point* at the interface between the control electronics and the interferometer—again the precise division is somewhat arbitrary, but the electronic signal input to an actuator is frequently employed as the point of reference.

With these points defined, the part of the loop from error-point to feedback-point is called the *controller* or just the feedback, and the rest of the loop from feedback point to the error point, in the causal direction, is called the *plant*.

Before a loop is activated, the signal that would be measured at the error point is called the *error signal*. In interferometry this is usually derived as an output from the optical system and its photo-detectors, as explained in Sect. 8.5.

### Linear time-invariant control theory: introductory concepts

A full description of linear time-invariant (LTI) theory is beyond the scope of this article, therefore we restrict our description to a short summary of the essential concepts, with some relevant examples presented in the following sections.

In LTI models the superposition principle applies, frequencies do not mix and it is possible to represent any physical time-domain signals in the frequency domain through their Fourier transforms. The time-invariance means that the response of a system to an input does not depend on the time at which that input is applied. This implies that the differential equations describing the system are linear and homogeneous with coefficients that are constant in time. In this case it is common to solve these equations by employing methods based on the Laplace transform. The response of the system is represented by its *transfer function* which is the Laplace transform of its *impulse response*—i.e., the output produced in the time domain when the input is a Dirac-delta function. We look at transfer functions in mode detail in Sect. 8.5.

For LTI systems the eigenfunctions or basis functions of the solution are the complex exponentials. Consider an input of the form

8.1 $$\begin{aligned}
								S_I=Ae^{st}, \end{aligned}$$

where *A* is a complex factor, *s* the Laplace transform variable and *t* is time, to a particular system. If the output is

8.2 $$\begin{aligned}
								S_O=Be^{st}, \end{aligned}$$

with different complex factor *B*, the system is described in full by the transfer function

8.3 $$\begin{aligned}
								T=\frac{S_O}{S_I}=\frac{Be^{st}}{Ae^{st}}=\frac{B}{A},
								\end{aligned}$$

which is not a function of time.

By implication, if the input to the system is a sinusoid (a single Fourier component) the output is also a sinusoid with, generally, different amplitude and phase as described by the transfer function. If the system consists of a series of optical, electronic and mechanical stages or sub-systems, the overall transfer function is the product of the individual transfer functions. If there is feedback, then *loop-algebra* can be applied, and this represents summation of the signal from one point in a loop into an earlier point (feedback), or in the case of feed-forward to a later point. This is also a linear operation, and so the system with feedback or feed-forward retains its LTI property. The most complicated case that need be considered is when there are two or more nested feedback loops (or equivalently two or more actuators that adjust one degree of freedom (Shapiro et al. 2015)), in this case there are loop-algebraic techniques that allow the system to be reduced to a single equivalent loop, such that nested loops of any practical topology may be considered step by step.

In modelling LTI systems it is common to pick a *representation* from one of a mathematically-equivalent set of options. The alternatives are described in, e.g., Franklin et al. (1998). To give some examples, individual loops or blocks with one input and one output (called SISO—single input, single output systems), are commonly represented by their transfer functions.

For LTI systems, the transfer functions can be written as rational polynomials in the complex frequency variable *s*. The polynomials on both the numerator and denominator of the transfer function can be factorised. This leads to an equivalent mathematical representation of the transfer function as a list of zeros (zeros of the numerator) and poles (zeros of the denominator). In addition an overall gain factor, independent of frequency, is usually required. The poles and zeros may be represented by their coordinates in the complex (*s*)-plane, or more phenomenologically by their resonant frequencies and damping factors. This leads to the so-called (*z*, *p*, *k*)-notation, where *z* represents one or more zeros, *p* the poles, and *k* represents an overall frequency-independent gain factor.

For MIMO systems arrays of transfer functions can be employed to describe all possible input–output relations, but it is more common to use the *state-space* representation. Here a single set of matrices encapsulates the behaviour of the entire system. A description of this important method is beyond the scope, but full detail is given in Franklin et al. (1998).

In briefest summary, the state-space method involves writing the set of *N* second order differential equations representing the internal dynamics of a system which has *N* degrees of freedom. These are reduced to 2*N* first-order equations by introducing the time derivatives, i.e., generalised velocities, of the displacement-like coordinates. The solution of the resulting set of equations is then usually carried out numerically, using matrix methods.

### Digital signal processing for control

In the past two decades digital control systems have been introduced into the control of interferometers, in modern instruments the majority of control systems contain digital processing elements, although the interfaces with the interferometer remain analogue in almost all cases. The essential principles remain the same as in the continuous-time systems, and a common approach to the design of digital control systems starts by designing and simulating a continuous-time analogue model. When this model operates as required in simulation, the result is transformed to the discrete-time mathematics of digital control. The resulting filters are then implemented in a combination of software and hardware. In discrete-time models only a finite set of frequencies exist, limited at high frequencies by the Nyquist frequency, i.e., half of the sampling rate in the digital system.

Digital models also have finite amplitude resolution, with the practical resolution limits occurring at the analogue-digital and digital-analogue interfaces (ADC, DAC respectively), rather than in the digital signal processing. These limitations are generally handled by *whitening* the signal at the input and *de-whitening* at the output. For example, input signals with predominant low-frequency content may be high-pass filtered to render their spectral content relatively uniform, i.e., white, before sampling to make best use of the available resolution. The converse process can be applied at the output, with sufficient low-pass filtering applied to ensure that white noise resulting from the output conversion (DAC) is suppressed within the gravitational wave band.

In the description of digital controllers, the discrete mathematics of the *z*-plane replaces the continuous nature of the *s*-plane (Franklin et al. 1998). Transforming from one space to the other is something of an art, mainly due to the consequences of finite precision in the associated calculations. A bilinear transformation is commonly employed. To avoid problems of numerical accuracy in the associated calculations, complicated systems are broken down in to a series of second-order sections. These subsystems have up to two poles and two zeros, i.e., the transfer functions have no higher order than quadratic numerator and denominator. Such subsystems can be transformed more reliably.

**Fig. 60:**
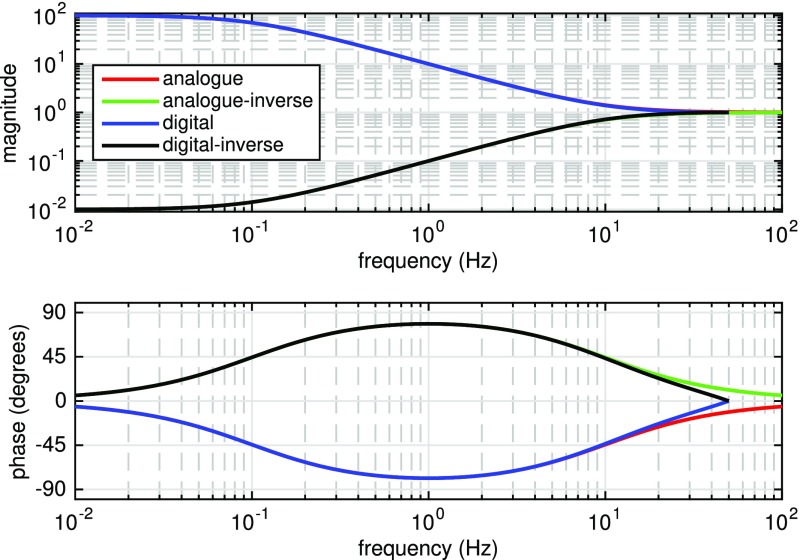
Bode plots to compare a digital representations with a first-order analogue transfer function and its inverse. The analogue system has a single real pole at 0.1 Hz and single real zero at 10 Hz. When the transfer function is inverted the pole becomes a zero, and the zero a pole. The bilinear transform is employed to produce the digital equivalents, with a sampling frequency of 100 Hz. The highest frequency that can be represented in this case is 50 Hz and it can be seen that the digital response becomes a poor approximation to the analogue one at frequencies approaching this limit. Note that, in a practical digital system, there would be a finite time delay and corresponding phase-lag, not included here, and that further delays may be present from anti-aliasing and anti-imaging filters—see text

**Fig. 61:**
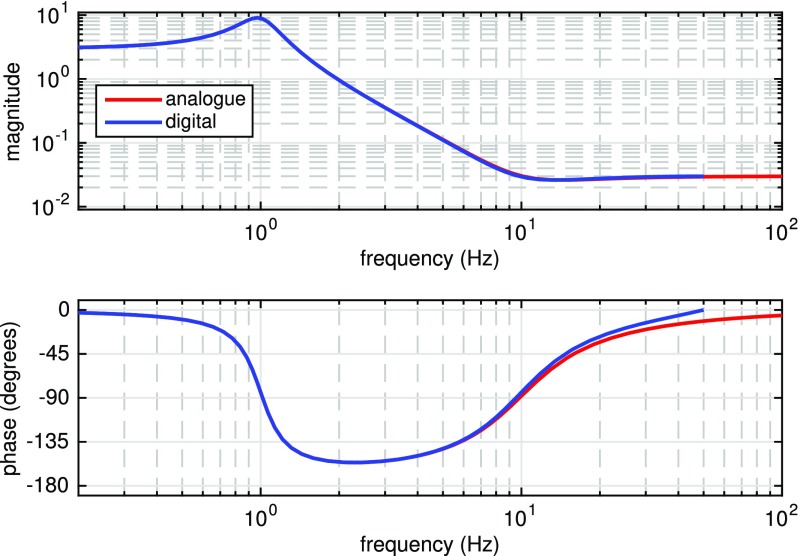
Bode plots for a second-order analogue system, and a digital approximation thereto. Here there is a complex pair of poles at 1 Hz with a resonance quality factor $Q=3$, and a pair of zeros at 10 Hz, $Q=1$. There is an overall gain factor of 3. The digital sampling frequency is 100 Hz, so signal frequencies are limited to 50 Hz

The finite time-steps in digital processing limit the filter transfer functions that may be produced at frequencies approaching the Nyquist limit—see Figs. 60 and 61. Note however that with modern computers the sampling rate is often limited by the analogue interfaces rather than the speed of processing. Where this is true the data stream can be up-sampled (interpolated) to a higher sampling rate for filtering, and then down-sampled (decimated) to the original sampling rate before conversion back to the analogue domain. This process can be used to improve the high-frequency response of digital filters.

One further consequence of discrete time is that there is a finite delay associated with the analogue to digital, signal processing and digital to analogue steps. This must be considered in the development of feedback loops based on digital controls. In practice, even more severe limits to high-frequency performance often arise from anti-aliasing or anti-imaging filters that may be required on the analogue input and outputs, respectively.

Aliasing occurs when the ingoing signal contains significant amplitude components at Fourier frequencies above the Nyquist limit. If these are not filtered out, they are incorrectly recorded their beat frequencies with the nearest harmonic of the sampling frequency. At the output, the digital signal has discrete steps from one sample to the next. To properly reconstruct the required analogue signal these steps require to be removed by the low-pass action of an anti-imaging (or reconstruction) filter. Further detail of the sampling and reconstruction processes is found in Franklin et al. (1998).

### Degrees of freedom and operating points

We consider the optical components to be rigid bodies, each with six degrees of freedom. With practical, high-quality spherical surfaces, only three degrees of freedom per component are important: position along the direction of propagation of the light, referred to as the *longitudinal* coordinate, the *yaw* angle with respect to that direction, i.e., in the horizontal plane, and the *pitch* angle in the vertical plane. The other three degrees of freedom (vertical, horizontal normal to the beam and roll around the axis of the beam) may be important with respect to noise coupling into the length measurement in the case of imperfect mirrors. As discussed in Sect. 11.5, the mirrors typically have only small deviations from ideal spheres, so the coupling factors are small and do not significantly affect the control of the interferometer.

In the interferometer as a whole, one component, for example, a mirror or beam splitter, may be chosen as the origin for the coordinate system. This allows one position and a pair of angles to be pre-defined. The positions and angles of the other components may then be described with respect to this origin. Note, however, that the longitudinal degrees of freedom are measured with an optical ‘ruler’ that is based on the wavelength of the light, and so the wavelength should be counted as one longitudinal degree of freedom in the system as a whole (in the sense that the light has the same frequency everywhere which is usually true to a good approximation in the ultra-stable environment of a gravitational-wave detector). Similarly the direction of the light beam entering the interferometer defines two angles.

To take an example, a simple cavity that is to be held on resonance with in-going light has two meaningful longitudinal degrees of freedom. For a cavity in isolation it would be usual to consider the position of one mirror relative to the other and the frequency, or wavelength, of the light as the important parameters. Mathematically there are other equivalent choices, but in the control and operation of interferometers the point is to find a convenient set of control variables.

Similarly, a simple Michelson interferometer has three components and three longitudinal degrees of freedom. Again it would be usual to consider one component as a reference. If the beam splitter is fixed, the three degrees of freedom are the two arm-lengths and the optical wavelength, or frequency.

If a pair of cavities were to be placed, one each, into the arms of the simple Michelson, the single degree of freedom of each mirror is replaced with the two of the cavity, for a total of five: once again, the same as the number of components. Fixing the beam splitter, these are the laser wavelength, the two distances from the beam splitter to the near mirrors of the cavities and the two lengths of the cavities.

An example of the degrees of freedom relevant to longitudinal control of a more complex system is shown in Sect. 8.12.

The choice between employing absolute or relative coordinates for the positions (and angles) of interferometer components is reflected in differences of approach in the available modelling software. In a Finesse model of a two-mirror cavity, for example, the longitudinal positions of the two mirrors are specified, and adjusting either of them changes the resonant condition of the cavity (see, for example, Sect. 5.6.1). Likewise, adjusting the position of the input mirror changes the phase of both the light in the cavity, and the light reflected from the cavity. See Sect. 2.1 for a discussion of this point.

An operating interferometer requires various interference conditions to be maintained, e.g., cavities should be kept on resonance, the dark-fringe condition in a Michelson interferometer must be met, and so forth. For each degree of freedom this implies that there is an optimum value for best sensitivity or an *operating point* in the multi-dimensional space representing the degrees of freedom. This point is not usually unique: for example, signals repeat modulo one round-trip wavelength, see below.

As most or all degrees of freedom are subject to movement or drift, they must be controlled, generally by designing and implementing a separate control loop for each one. These loops must be designed to hold the value of the degree of freedom close to such an operating point, where ‘close’ is determined by tolerance bounds that must be determined by calculation.

In most cases it is possible to evaluate a tolerance interval around the operating point. The limits usually arise in the consideration of the coupling of some kind of noise into the sensitive measurement (frequency noise, power noise, beam direction noise, etc.). For example, in the case of the dark fringe, sensitivity to laser power noise is at a minimum at the perfectly dark condition, and the tolerable increase in this coupling may be used to set bounds on deviations from the operating point.

Bounds may also be set by considering the required linearity of signals. Non-linearity can lead to beating, which mixes noise into the measurement band. For example if there is a narrow spectral feature or ‘line’, such as a calibration line that may be applied to monitor instrumental sensitivity, or a suspension violin mode^12^ in the measurement band, beating this with low frequency motion of suspended components will produce sidebands on either side of the narrow feature, and these may be of higher amplitude than the noise background at the frequencies of interest near the line. Non-linear operation may also cause problems for control systems, as its presence implies that the gain of control loops will depend on the magnitude of deviations from the nominal operating point. In the following (Sect. 8.5) it will be seen that the normal process of sensing the length of a cavity is reasonably linear only within a very narrow range, in comparison to the wavelength of light, around the operating point, at least for a cavity of high finesse—a range of distance of order $$\lambda /{ F
								}$$ (see Sect. 5.1), or smaller.

In designing a control system for an interferometer, one can in principle consider the space of possible values of all the degrees of freedom in an interferometer, but it is more usual to work with a sub-space, e.g., only the longitudinal degrees. In the angular case there is usually a unique operating point per degree of freedom corresponding to one optimal alignment, but in the longitudinal case operating points are repeated as the relevant round-trip phase change steps in multiples of $$2\pi
								$$, i.e., one wavelength change in round-trip optical path distance. In this case it is usual to consider the (hyper-)volume containing one repeat in each dimension: for km-long interferometers there is very little difference between adjacent volumes. In a typical interferometer designed for gravitational wave detection, the number of degrees of freedom in combination with requirements on noise coupling and linearity mean that only a vanishingly small volume within the phase space corresponds to the acceptable region around the desired operating point. This suggests one of the important questions in operating a gravitational wave detector: how to bring every degree of freedom to the desired operating point.

**Fig. 62:**
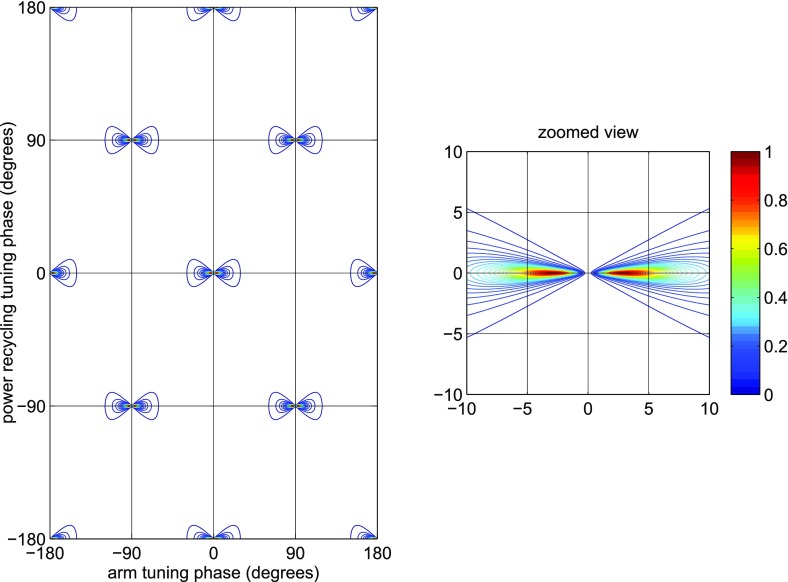
Simplified example illustrating the restricted phase space volume in which interferometer control signals are expected to achieve significant magnitudes. In the *left panel* we show the modulus of the light amplitude emerging at the detection/output port of a power recycled Michelson interferometer as a function of both the length difference between the arms (in the plot, the two end mirrors have their tuning phase shifted by the amount shown, but in opposition) and the tuning of the power recycling (the mirror has its tuning phase shifted by the amount shown). The normalised amplitude is shown on a linear scale from zero to one. These two degrees of freedom are swept over two cycles from the nominal operating point at (0,0). The other possible longitudinal degrees of freedom, namely the common mode arm length and the optical frequency are kept constant. The size of a signal designed to sense the differential degree of freedom, and allow the interferometer to be locked at the operating point, would have significant magnitude only in the region of the features like the one near (0,0) where there is a significant gradient in the horizontal direction in the plot. In this example, the power recycling mirror has a transmission of $1\,\%$, and other components have no loss. In a practical detector there would be several other degrees of freedom. Typically there would also be cavities of higher finesse, leading to even narrower features. The right panel shows a magnified view of one of the ‘islands’ of useful signal, the operating point is a small region at coordinates (0,0) where the light power is low

The diminutive scale of the useful volume in phase space can be illustrated by means of a simplified example. Here we consider the case of two degrees of freedom in a power recycled Michelson interferometer. Even fixing the location of the beam splitter, there are three degrees of freedom (i.e., common arm length, differential arm length and longitudinal position of the power recycling mirror. However, we choose to produce a contour plot showing signal sizes as a function of just two degrees of freedom, Fig. 62. Here we vary the difference in the lengths of the two arms while keeping the average (or common mode) arm length fixed, and also to vary the position of the power recycling mirror. In a practical interferometer there would be several other degrees of freedom associated with, for example, arm cavities, signal recycling and control of the common-mode arm length (or laser frequency), and in most cases the cavities would be of higher finesse producing even narrower features—see, for example, the parameters for Advanced LIGO in “Appendix B”.

The complexity of sensing and control becomes apparent when one considers that, in the common case of the freely-suspended optical components in a ground-based interferometric gravitational wave detector, the initial condition, at the point of ‘switching on’ the controls can be any random point within the space, with—in addition—a wide range of initial velocities associated with each degree of freedom: up to perhaps of order one wavelength per second, in a typical ground-based instrument. How this is dealt with is summarised in Sect. 8.1.

### Error signals and transfer functions

In general, we will call an *error signal* any measured signal suitable for stabilising a certain experimental parameter *p* with a servo loop. The aim is to maintain the variable *p* at a user-defined value, the *operating point*, $p_0$. Therefore, the error signal must be a function of the parameter *p*. In most cases it is preferable to have a bipolar signal with a zero crossing at the operating point. The slope of the error signal at the operating point is a measure of the ‘gain’ of the sensor, which in the case of interferometers is a combination of optics and electronics.

*Transfer functions* describe the propagation of a periodic signal through a *plant* and are usually given as plots of amplitude and phase over frequency, e.g., as Bode plots (see the following section). By definition a transfer function describes only the linear coupling of signals inside a system. This means a transfer function is independent of the actual signal size. For small signals or small deviations, most systems can be linearised and correctly described by transfer functions.

Experimentally, network analysers are commonly used to measure a transfer function: one connects a periodic signal (the *source*) to an actuator of the plant (which is to be analysed) and to an input of the analyser. A signal from a sensor that monitors a certain parameter of the plant is connected to the second analyser input. By mixing the source with the sensor signal the analyser can determine the amplitude and phase of the input signal with respect to the source (amplitude equals one and the phase equals zero when both signals are identical).

Mathematically, transfer functions can be modelled similarly: applying a sinusoidal signal $$~\sin (\omega _s
								t)$$ to the interferometer, e.g., as a position modulation of a cavity mirror, will create phase modulation sidebands with a frequency offset of $$\pm \omega
								_s$$ to the carrier light. If such light is detected in the right way by a photodiode, it will include a signal at the frequency component $$\omega
								_s$$, which can be extracted, for example, by means of demodulation (see Sect. 4.2).

**Fig. 63:**
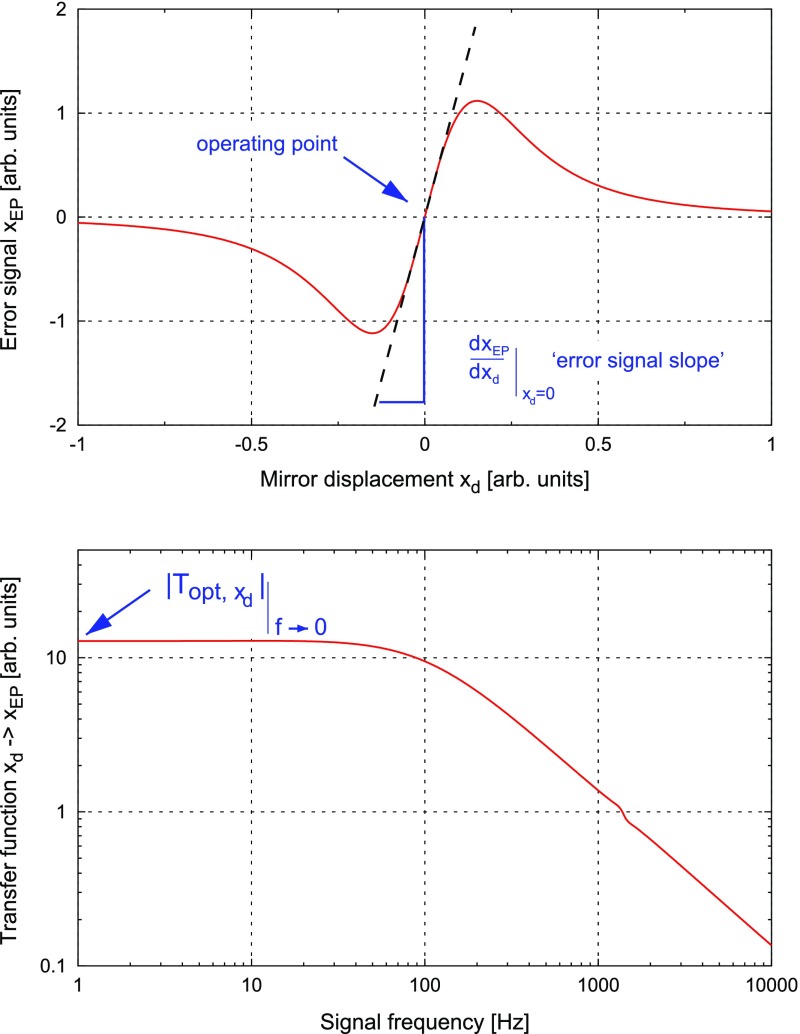
Example of an error signal: the *top graph* shows the electronic interferometer output signal as a function of mirror displacement. The operating point is given as the zero crossing, and the *error-signal slope* is defined as the slope at the operating point. The *bottom graph* shows the magnitude of the transfer function *mirror displacement* $$\rightarrow
											$$ *error signal*. The slope of the error signal (*top graph*) is equal to the low frequency limit of the transfer function magnitude [see Eq. (8.6)]

Transfer functions are of particular interest in relation to error signals. Typically a transfer function of the error signal is required for the design of the respective electronic servo. A ‘transfer function of the error signal’ usually refers to a very specific setup: the system is held at its operating point, such that, on average, $\bar{p}=p_0$. A signal is applied to the system in the form of a very small sinusoidal disturbance of *p*. The transfer function is then constructed by computing for each signal frequency the ratio of the error signal and the injected signal. Figure 63 shows an example of an error signal and its corresponding transfer function. The operating point shall be at

8.4 $$\begin{aligned}
								x_{\mathrm {d}} = 0 \quad \text {and} \quad x_{\scriptscriptstyle
								\mathrm {EP}}(x_{\mathrm {d}}=0) = 0.
								\end{aligned}$$

The optical transfer function $$T_{\mathrm {opt,
								x_{\mathrm {d}}}}$$ with respect to this error signal is defined by

8.5 $$\begin{aligned}
								\widetilde{x}_{\scriptscriptstyle \mathrm {EP}}(f) = T_{\mathrm
								{opt, x_{\mathrm {d}}}} T_{\mathrm {det}} \widetilde{x}_d(f),
								\end{aligned}$$

with $$T_{\mathrm
								{det}}$$ as the transfer function of the sensor. In the following, $$T_{\mathrm
								{det}}$$ is assumed to be unity. At the zero crossing the slope of the error signal represents the magnitude of the transfer function for low frequencies:

8.6 $$\begin{aligned}
								\left| \frac{d x_{\scriptscriptstyle \mathrm {EP}}}{d x_{\mathrm
								{d}}}\right| _{~\bigl |x_{\mathrm {d}}=0\bigr .}=|T_{\mathrm {opt,
								x_{\mathrm {d}}}}|_{~\bigl |f\rightarrow 0\bigr .}
								\end{aligned}$$

The quantity above will be called the *error-signal slope* in the following text. It is proportional to the *optical gain* $$|T_{\mathrm
								{opt}, x_{\mathrm {d}}}|$$, which describes the amplification of the gravitational-wave signal by the optical instrument.

### Bode plots: traditional control theory for SISO loops

An essential feature of a control system is stability, i.e., for a finite input the output should always be bounded. This is equivalent to requiring all of the transfer function poles to correspond to decaying exponentials, so their real parts must be strictly negative.

Prior to the routine application of computers, a number of tools (plots) were developed to facilitate control system design. Although the root-locus, Nyquist and Bode plots continue to be applied, computer models remove the practical (calculational) advantages of one over another. All of these methods present essentially equivalent information, and the choice of one over another is a matter of convenience or familiarity. Since Bode plots provide a complete description of minimum-phase, single-input single-output (SISO) LTI control loops we choose to describe that approach as an example.

Throughout this section, the system is described in continuous time, i.e., as an analogue model. When the technique is applied to digital control systems discrete-time models are needed as discussed in Sect. 8.3.

**Fig. 64:**
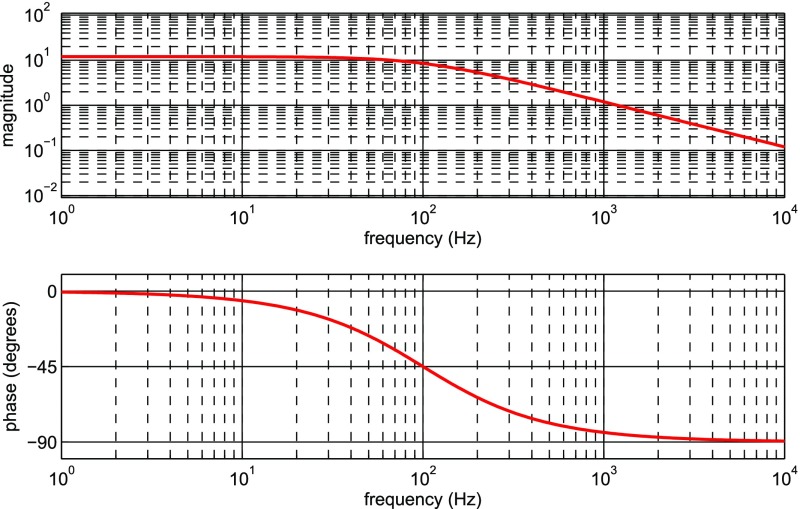
Example Bode plot for a simple first order low-pass transfer function with a gain of 12 at low-frequency and a single pole at 100 Hz. The *upper panel* shows the magnitude of the response, presented on a logarithmic scale, as a function of frequency (here in Hz, may also be radian/s). The *lower panel* shows the corresponding phase in degrees. As noted in the text, for minimum-phase systems such as this, the magnitude and phase are not independent, but both provide useful information in the design of control loops. Where the response is flat with frequency, the phase is asymptotically zero; where the response varies as 1 / *f*, there is a phase lag approaching 90$$^{\circ }$$. As explained in the text, if this plot were to represent the open-loop transfer function of a negative feedback loop, important properties of the loop can be read-off by inspection. For example, stability can be assessed by checking the phase margin at the unity gain frequency (here the gain is unity at 1200 Hz). At this frequency the phase is about $$-85^{\circ
											}$$ and the phase margin is given by the difference of this from $$-180^{\circ
											}$$, or $$95^{\circ }$$. This is far from zero, and so the closed-loop response is predicted to be stable

A Bode plot of a system shows its transfer function in the form of log-magnitude and linear-phase graphs against a logarithmic frequency axis—conventionally in vertically-stacked plots with matching, aligned frequency axes, see Fig. 64 for a simple example. In the context of the design of complete negative feedback loops, it is common, though not universal, to add $$\pi
								$$ to the phase to represent the overall negative sign—this convention is assumed here. The standard procedure starts with consideration of the open-loop Bode plot. In this, the loop is broken (in the model) at a convenient point, and the transfer function from there back to just before the break is calculated and plotted. Remember that the total transfer function is computed as the product of individual transfer functions of parts of the loop that are connected in series. Particular attention is paid to the regions close to points where the transfer function magnitude crosses unity (i.e., zero on the log scale), called the unity gain point(s), and where the phase crosses $$-\pi
								$$ in absolute terms, *not* modulo $$2\pi
								$$. The transfer function is then characterised by the phase margin and the gain margin. The phase margin is the phase of the transfer function plus $$\pi
								$$ at the frequency where the gain is unity, and the gain margin is the inverse of the gain where the phase is $$-\pi
								$$ (or the negative of the log gain). If there are multiple unity gain points the smallest phase margin, and the smallest gain margin, dominate. If the smallest gain and phase margins are both positive, the system is stable. Note that if there are multiple paths or ‘loops’, these are dealt with by applying loop algebra to reduce the system to a single feedback loop without subsidiary loops.

Traditionally these methods were extended to reveal properties of the closed-loop system, i.e., of the original model without any break. This was done because, for transfer functions of low order (one, two or three poles), there are simple expressions that relate the phase margin to the ringing, or equivalently damping, of the closed-loop response. When computer models are employed for systems of greater complexity there is no need for these rules or guidelines and it is common to transform back to the time domain, calculate the impulse response of the closed loop system, and characterise its resonant frequencies and damping without reliance on rules.

As a concrete example of a Bode plot, we include one representing a system that approximates the transfer function shown in Fig. 63). The system consists of a gain factor (12) and a single pole at 100 Hz (or $$2\pi \times
								100$$ radian/s). The transfer function may be written

8.7 $$\begin{aligned}
								G(s) = \frac{12}{s+200\pi },
								\end{aligned}$$

such that there is a single real pole at complex frequency $$s=-200\pi
								$$. Further explanation of the mathematics of transfer functions is given in Sect. 8.2, while Bode plots of higher order systems in both continuous and discrete time are found in Sect. 8.3.

The construction and utility of the Bode plot originates in part from the properties of a common subset of transfer functions that represents stable, causal systems. Such systems are called *minimum phase* as a consequence of the locations of their zeros in the *s*-plane. In a causal system the output lags the input. Stable, causal LTI systems are also invertible, i.e., the transfer function numerator and denominator can be swapped, or equivalently all the poles and zeros may be exchanged resulting in another stable, causal system. For this to work the zeros of the system must have negative real parts, so that when they become poles in the inverse system they are damped. It can be shown that in such a system there is a strict relationship between the phase and the slope of the log-magnitude, as shown on a Bode plot—one is a Hilbert transform of the other. In practice this is equivalent to writing that the when the magnitude graph has a slope of $$f^{-n}$$, where *f* is the frequency, the phase approaches $$-n\pi
								/2$$. This method allows the loop to be designed to meet various goals that are usually expressed in terms of gain (or attenuation) that must be achieved in one or more range of frequencies, with stability checked by reading off the phase and gain margins.

In interferometry the *optical transfer function* is usually a significant aspect of control loops. Such transfer functions may be measured or found by calculation (e.g., with Finesse). The corresponding transfer functions can be found by applying the techniques described in Sect. 8.5.

### Separating mixtures of the degrees of freedom: control matrices

In practice, each error signal intended to represent a particular degree of freedom of the optical arrangement also contains some information about other degrees of freedom. To give a simple example of the mixing that may occur, any motion that leads to a change in the circulating light power in a cavity is likely to couple, at some level, to every signal that depends on the intra-cavity light, unless the signal is precisely zero.

In most cases such mixing is undesirable as it is easier to design control systems to deal with one degree of freedom in isolation. In the worst case, if the mixing, or cross-coupling is strong, it can lead to the formation of unintended feedback paths. If the transfer function of such loops has a magnitude exceeding unity, there is a chance that the loop may be unstable. A common cause of such instability is a resonance in the unintended or ‘parasitic’ loop. At such a resonance high gain is typically accompanied by a phase lag of $$-\pi
								$$ which will tend to be unstable unless some compensation is included, e.g., in the form of a notch filter to cancel the resonance.

Unwanted mixing of signals can also occur at the point of actuation. For example, a mirror may be common to two degrees of freedom of an interferometer. In an interferometer with arm cavities, the cavity mirrors closest to the beam splitter behave in this way. Moving such a mirror must then affect at least two length degrees of freedom. This can be seen in Fig. 51 where motion of either of the two mirrors labelled ITMX and ITMY affects the phase of the light in the respective arm cavity and also the interference condition of the Michelson interferometer. In contrast, the end mirrors (ETMX, ETMY) each affect only one longitudinal degree of freedom.

A further possible source of mixing between degrees of freedom arises at the point of actuation. Feedback to control a mirror is often carried out in practice using an array of actuators, such as coil-magnet pairs, that push on the mirror at various points on its surface. For example, it is common to employ a square-array of four magnets attached to the rear surface of the mirror, as these allow longitudinal, pitch and yaw adjustment. If they are mounted close to the perimeter of the rear surface they may be out of the way of a transmitted light beam. With such an arrangement, each individual actuator causes changes to a mixture of angular and longitudinal degrees of freedom. If the actuators are not of precisely uniform strength and alignment, this leads to unintended components in the resultant force produced by the array. An *actuation matrix*, with frequency-dependent elements where necessary, can be employed to orthogonalise the response of the system to commands from the controller, at least to some degree of precision.

The elements of actuation and sensing matrices are typically determined as a result of simulation and measurement. Modelling may yield a set of starting values that suffice to allow the interferometer to operate. When operational residual mixing is normally determined by carrying out all possible transfer function measurements. The measurements allow coupling matrices to be determined, and inverting the coupling matrix provides the appropriate matrix necessary to remove unwanted mixing. This process is somewhat involved and benefits from automation.

### Modern control methods in gravitational-wave detectors

During the past few decades new methods of designing sophisticated controllers based on digital signal processing have emerged. A major benefit of the resulting ‘digital controls’ is that the response of a control filter can be adjusted by changing filter coefficients, this can even be achieved while the controller is operating, if that is required.

Digital control facilitates the application of so-called modern control methods in which optimisation methods are employed. As an indication of the possible advantages that may arise from this, we briefly mention two approaches to modern control of application in interferometry. For a relevant description of these see, e.g., Franklin et al. (1998).

In the first approach, we consider the generation of an optimal filter with fixed coefficients (gain, poles and zeros). In such a case, the plant to be controlled is characterised by some means, and the results are used in the design of an optimal filter. For example, if it can be assumed that a measurement produces an estimate of the system contaminated by noise, and a model of the system with the correct number of degrees of freedom exists, a Wiener filter may be formed as a result of least-squares fitting the model to the data. If the result is to be inverted to provide a compensating filter in a control system, then the fit must be constrained produce a causal filter (with all poles and zeros having negative real parts, in an analogue model). The are standard methods by which this may be accomplished.

The next step up in sophistication is to find a controller that remains optimal even if the underlying plant changes (or if its parameters cannot be measured accurately before the controller is put into operation). Such an *adaptive* controller, employs a Kalman filter—also called a Linear Quadratic Estimator. This is implemented as an algorithm that operates on a series of measurements taken over time. These measurements are assumed to be contaminated with noise. The algorithm operates recursively to produce an optimal estimate of the state of the physical system. During this process a model of the system, i.e., a representation of the equations of motion, with relevant coefficients available to be adjusted, is iteratively updated. The model is assumed to have errors either as a result of poor starting estimates or due to drifting of parameters over time. A weighting function, also called a *cost function*, is applied to the measured data to allow less noisy or otherwise more important aspects of the data to have a stronger influence on the outcome. At each iteration the model is employed to predict the current state, this is then compared with the actual state and the results of the comparison are used to refine and update the model. When this method is made to operate, the model of the underlying system converges to an optimal solution for the given weighting function.

### Fabry–Perot length sensing

In Fig. 27 we have plotted the circulating power in a Fabry–Perot cavity as a function of the laser frequency. The steep features in this plot indicate that such a cavity can be used to measure changes in the laser frequency. From the equation for the circulating power [see Eq. (5.1)],

8.8 $$\begin{aligned}
								P_1/P_0=\frac{T_1}{1+R_1R_2-2r_1r_2\cos \left( 2 k L\right)
								}=\frac{T_1}{d}, \end{aligned}$$

we can see that the actual frequency dependence is given by the $$\cos (2 k
								L)$$ term. Writing this term as

8.9 $$\begin{aligned}
								\cos (2 k L)=\cos \left( 2\pi \frac{Lf}{c}\right) ,
								\end{aligned}$$

we can highlight the fact that the cavity is in fact a reference for the laser frequency in relation to the cavity length. If we know the cavity length very well, a cavity should be a good instrument to measure the frequency of a laser beam. However, if we know the laser frequency very accurately, we can use an optical cavity to measure a length. In the following we will detail the optical setup and behaviour of a cavity used for a length measurement. The same reasoning applies for frequency measurements. If we make use of the resonant power enhancement of the cavity to measure the cavity length, we can derive the sensitivity of the cavity from the differentiation of Eq. (5.1), which gives the slope of the trace shown in Fig. 27,

8.10 $$\begin{aligned}
								\frac{d\,P_1/P_0}{d\,L}=\frac{-4 T_1 r_1 r_2 k \sin (2 k L)}{d^2},
								\end{aligned}$$

with *d* as defined in Eq. (8.8). This is plotted in Fig. 65 together with the cavity power as a function of the cavity tuning. From Fig. 65 we can deduce a few key features of the cavity:

- The cavity must be held as near as possible to the resonance for maximum sensitivity. This is the reason that active servo control systems play an important role in modern laser interferometers.
- If we want to use the power directly as an error signal for the length, we cannot use the cavity directly on resonance because there the optical gain is zero. A suitable error signal (i.e., a bipolar signal) can be constructed by adding an offset to the light power signal. A control system utilising this method is often called *DC-lock* or *offset-lock*. However, we show below that more elegant alternative methods for generating error signals exist.
- The differentiation of the cavity power looks like a perfect error signal for holding the cavity on resonance. A signal proportional to such differentiation can be achieved with a modulation-demodulation technique.

**Fig. 65:**
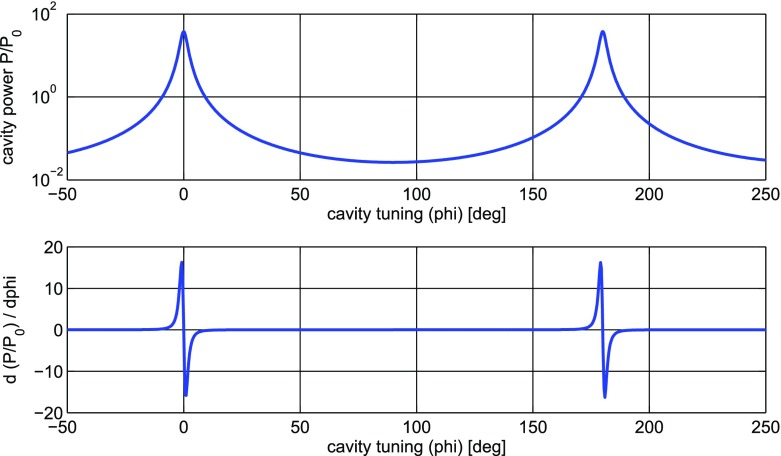
The *top plot* shows the cavity power as a function of the cavity tuning. A tuning of 360° refers to a change in the cavity length by one laser wavelength. The *bottom plot* shows the differentiation of the upper trace. This illustrates that near resonance the cavity power changes very rapidly when the cavity length changes. However, for most tunings the cavity seems not sensitive at all

**Fig. 66:**
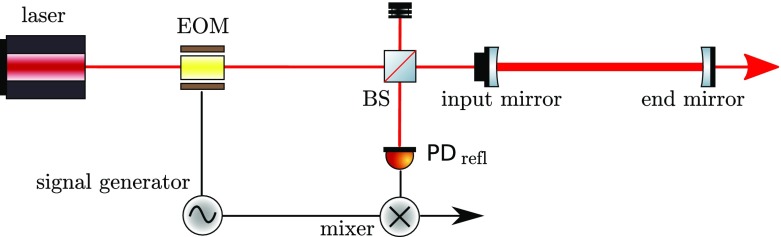
Typical setup for using the Pound–Drever–Hall scheme for length sensing and with a two-mirror cavity: the laser beam is phase modulated with an electro-optical modulator (EOM). The modulation frequency is often in the radio frequency range. The photodiode signal in reflection is then electrically demodulated at the same frequency

### The Pound–Drever–Hall length sensing scheme

**Fig. 67:**
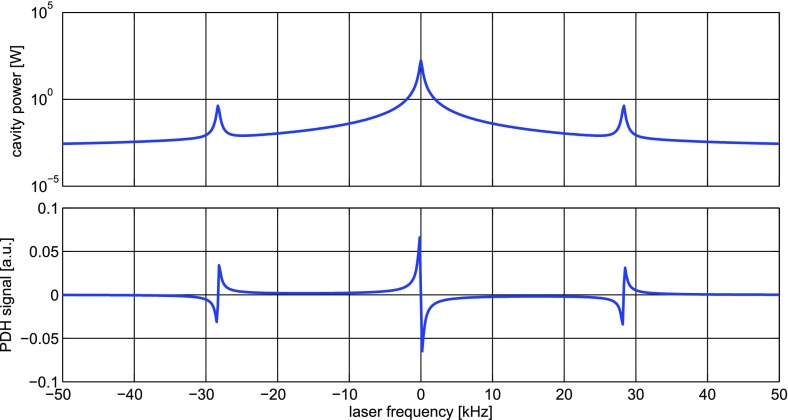
This figure shows an example of a Pound–Drever–Hall (PDH) signal of a two-mirror cavity. The *plots* refer to a setup in which the cavity mirrors are stationary and the frequency of the input laser is tuned linearly. The *upper trace* shows the light power circulating in the cavity. The three peaks correspond to the frequency tunings for which the carrier (main central peak) or the modulation sidebands (smaller side peaks) are resonant in the cavity. The *lower trace* shows the PDH signal for the same frequency tuning. Coincident with the peaks in the *upper trace* are bipolar structures in the *lower trace*. Each of the bipolar structures would be suitable as a length-sensing signal. In most cases the central structure is used, as experimentally it can be easily identified because its slope has a different sign compared to the sideband structures

This scheme for stabilising the frequency of a light field to the length of a cavity, or vice versa, is based on much older techniques for performing very similar actions with microwaves and microwave resonators (Pound 1946). Drever and Hall have adapted such techniques for use in the optical regime (Drever 1983) and today what is now called the *Pound–Drever–Hall* technique can be found in a great number of different types of optical setups. An example layout of this scheme is shown in Fig. 66, in this case for generating a length (or frequency) signal of a two-mirror cavity. The laser is passed through an electro-optical modulator, which applies a periodic phase modulation at a fixed frequency. In many cases the modulation frequency is chosen such that it resides in the radio frequency band for which low-cost, low-noise electronic components are available. The phase modulated light is then injected into the cavity. However, from the frequency domain analysis introduced in Sect. 5, we know that in most cases not all the light can be injected into the cavity. Let’s consider the example of an over-coupled cavity with the reflectivity of the end mirror $R_2<1$. Such a cavity would have a frequency response as shown in the top traces of Fig. 28 (recall that the origin of the frequency axis refers to an arbitrarily chosen default frequency, which for this figure has been selected to be a resonance frequency of the cavity). If the cavity is held on resonance for the unmodulated carrier field, this field enters the cavity, gets resonantly enhanced and a substantial fraction is transmitted. If the frequency offset of the modulation sidebands is chosen such that it does not coincide with (or is near to) an integer multiple of the cavity’s free spectral range, the modulation sidebands are mostly reflected by the cavity and will not be influenced as much by the resonance condition of the cavity as the carrier. The photodiode measuring the reflected light will see the optical beat between the carrier field and the modulation sidebands. This includes a component at the modulation frequency which is a measure of the phase difference between the carrier field and the sidebands (given the setup as described above). Any slight change of the cavity length would introduce a proportional change in the phase of the carrier field and no change in the sideband fields. Thus the photodiode signal can be used to measure the length changes of the cavity. One of the advantages of this method is the fact that the so-generated signal is bipolar with a zero crossing and steep slope exactly at the cavity’s resonance, see Fig. 67.

### Michelson length sensing

Similarly to the two-mirror cavity, we can start to understand the length-sensing capabilities of the Michelson interferometer by looking at the output light power as a function of a mirror movement, as shown in Fig. 31. The power changes as sine squared with the maximum slope at the point when the output power (in what we call the South port) is half the input power. The slope of the output power, which is the *optical gain* of the instrument for detecting a differential arm-length change $$\varDelta
								L$$ with a photo detector in the South port can be written as

8.11 $$\begin{aligned}
								\frac{dS}{d\varDelta L}=\frac{2\pi P_0}{\lambda }\sin \left(
								\frac{4\pi }{\lambda }\varDelta L\right)
								\end{aligned}$$

and is shown in Fig. 68. The most notable difference of the optical gain of the Michelson interferometer with respect to the Fabry–Perot interferometer (see Fig. 65) is the wider, more smooth distribution of the gain. This is due to the fact that the cavity example is based on a high-finesse cavity in which the optical resonance effect is dominant. In a basic Michelson interferometer such resonance enhancement is not present.

**Fig. 68:**
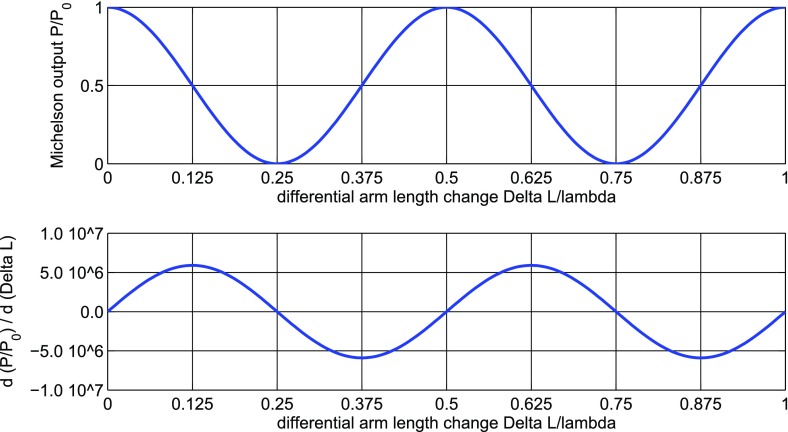
Power and slope of a Michelson interferometer. The *upper plot* shows the output power of a Michelson interferometer as detected in the South port (as already shown in Fig. 31). The *lower plot* shows the optical gain of the instrument as given by the slope of the *upper plot*

However, the main difference is that the measurement is made differentially by comparing two lengths. This allows one to separate a larger number of possible noise contributions, for example noise in the laser light source, such as amplitude or frequency noise. This is why the main instrument for gravitational-wave measurements is a Michelson interferometer. However, the resonant enhancement of light power can be added to the Michelson, for example, by using Fabry–Perot cavities within the Michelson as introduced in Sect. 7.2. This construction of new topologies by combining Michelson and Fabry–Perot interferometers has culminated in the dual-recycled Fabry–Perot–Michelson configuration that is the subject of the following section.

The Michelson interferometer has two longitudinal degrees of freedom (setting aside the optical wavelength as a third degree of freedom). These can be represented by the positions (along the optical axes) of the end mirrors. However, it is more efficient to use proper linear combinations of these and describe the Michelson interferometer length or position information by the *common* and *differential arm length*, as introduced in Eq. (5.10):

$$\begin{aligned}
								\begin{array}{l} \bar{L}=\frac{L_N+L_E}{2}\\ \varDelta L=L_N-L_E.\\
								\end{array} \end{aligned}$$

The Michelson interferometer is intrinsically insensitive to the common arm length $\bar{L}$.

### Advanced LIGO: an example of a complex interferometer

In this section we present a simplified overview of the dual-recycled Fabry–Perot–Michelson interferometer (*DRFPMI*) topology, as exemplified by the Advanced LIGO detectors (Harry and the LIGO Scientific Collaboration 2010). At this level of detail, the description applies equally to Advanced Virgo (Acernese 2015).

**Fig. 69:**
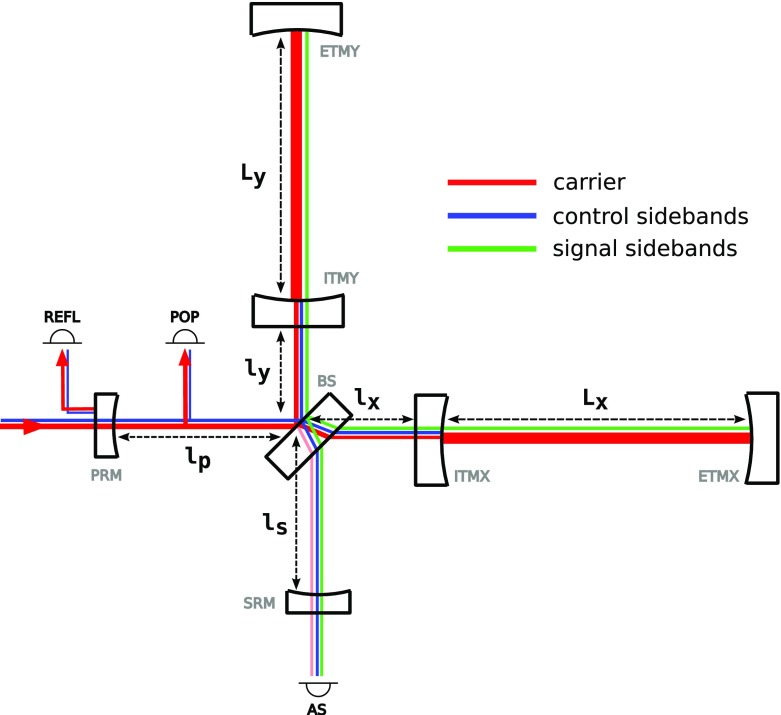
Schematic illustration of the dual-recycled Fabry–Perot–Michelson configuration showing the main optical components (i.e., 6 mirrors and the beam splitter), components of the light field in different regions of the interferometer, photo-detectors and one possible representation of the degrees of freedom. The system is controlled by signals obtained from three photo-detectors: REFL, short for reflected port, and POP, short for pick-off-port detect aspects of the light reflected by the Michelson, while the transmitted light is detected at the anti-symmetric port (AS). The degrees of freedom are indicated by the various lengths ‘L’ and ‘l’ with subscripts described in the text of the current section. Note that the lengths marked with capital ‘L’s involve the long arms of the interferometer, while the others involve the short distances from the beam splitter to the nearby components. Further detail of the sensing and control is discussed in “Appendix B”

Our description builds on the ideas presented in Sect. 7. The DRFPMI configuration is built around a Michelson interferometer, with 4 cavities added to modify the behaviour of the system. As shown in Fig. 69, there are two Fabry–Perot arm cavities that extend the light path in the arms of the interferometer to enhance the signal due to gravitational waves. The Michelson is operated at, or very close to, a dark fringe so that, apart from losses, most of the light is reflected back in the direction towards laser and injection optics, hence this input port is also called the ‘bright’ port of the interferometer. A partially transmitting power-recycling mirror, placed at the bright port and adjusted to resonate the light, allows the power circulating within the interferometer to build up (ideally by a factor of 1/loss), reducing the requirement for input light power.

The final cavity is formed by placing a partially transmitting mirror between the output or ‘dark’ port of the Michelson and the detection optics (consisting of a photo-detector, and perhaps some other components). This mirror recycles light that carries signal information to the photo-detector, and is called the signal recycling mirror—see Sect. 7.3 for an introduction to this aspect of the interferometer configuration.

The idea of a bright fringe or port and dark fringe or port can be extended to form one of the central concepts in the control of complex interferometers. In the condition described, with the input or power recycling port maintained in the bright state, and the output or signal recycling port held in the dark state, there is a separation of light-field components to one or other port according to their relative state in the interferometer arms. Here ‘component’ means light at a single frequency, i.e., a carrier or a sideband, and in a single optical mode (for a discussion of spatial modes, see Sect. 9). Such light-field components, which have spatial and temporal coherence, can interfere. If they have the same phase in the two arms they interfere constructively at the bright port. If they have the opposite phase in the two arms they interfere constructively at the dark port. Note that this arises because of the choice of interference of the carrier light to create the bright and dark ports.

In the same way that the carrier light which has a common phase in the two arms appears at the bright port, any perturbation of the interferometer that is common to the two arms generates higher order modes and/or sidebands that have the same phase in the two arms and thus causes an effect on the optical field at the bright port. Examples of this would be in-phase arm-length changes, or the addition of the same amount of optical loss in the two arms. On the other hand, perturbations that are exactly out-of-phase between the two arms have an effect on the light field at the dark port. An example would be that gravitational waves produce differential phase modulation sidebands that have opposite phase in the two arms, and these interfere constructively at the output port.

The distinction between effects that are either in-phase or have opposing phases is frequently important in the control of interferometers. As noted in the previous section for the case of the simple Michelson, it has become standard to consider the two physical degrees of freedom associated with the arms of an interferometer in logical-combination as the *common mode* and the *differential mode*. For the same reasons, the bright port is also called the *symmetric port* and the dark port is called the *anti-symmetric port*.

The advantage of the choice of common and differential modes may be seen in consideration of control loops to deal with laser-frequency fluctuations and to keep the interferometer locked at the dark fringe, to give but two examples. In a nearly-symmetrical interferometer a fluctuation of the frequency of the in-going light will lead to a primarily common-mode effect, and it makes sense to stabilise the laser frequency with respect to the common mode of the two arms. Similarly the gravitational wave signal may be read-out as part of the error signal of a control loop for the dark fringe. Such a loop should act on the differential mode, rather than on the length of one arm cavity, or the other.

Referring to Fig. 69, the physical optical path-lengths shown on the diagram may be related to the logical degrees of freedom applied in interferometer control in the following way. The solution presented here is not unique, but is intended as an example of one way to approach the problem. We deal with the degrees of freedom in turn, take the beam splitter as a point of reference for the small ‘l’ lengths (as suggested on the figure) and assume the laser frequency is fixed. This leaves 5 degrees of freedom to be controlled:

- **CARM** Common-mode arm length, CARM $$=L_x+L_y$$. This corresponds to the average length of the arm cavities and is adjusted to keep both arm-cavities on resonance.
- **DARM** Differential arm length, DARM $$=L_x-L_y$$. This corresponds to the difference in length of the two arm cavities and is used to maximise the constructive interference, at the output port, of sidebands resulting from differential arm-length changes (this degree of freedom is therefore the source of the gravitational wave channel).
- **MICH** Michelson arm length difference, MICH $$=l_x-l_y$$. MICH corresponds to the difference in length of the short arms of the Michelson, between the ITMs and the beam splitter, and determines the state of interference at the output port. In Advanced LIGO, the Michelson is operated close to the dark fringe.
- **PRCL** Power recycling cavity length, PRCL $$=L_p
											+\frac{l_x+l_y}{2}$$. The power recycling cavity is operated on resonance to maximise the power coupled into the central interferometer.
- **SRCL** Signal recycling cavity length, SRCL $$=L_s+\frac{l_x+l_y}{2}$$. This corresponds to the resonance condition of the signal recycling cavity. The operating point of SRCL depends on the mode of operation of the interferometer. It can be tuned for a particular frequency of gravitational wave or for broadband operation.

As a reminder, we restate that in a gravitational-wave detector, we are concerned with microscopic variations of path lengths that may be up to several km.

In the following sections we discuss, in general terms, how the error signals can be extracted from the optical system, combined and processed to provide signals representing the degrees of freedom to be controlled, and how the resulting signals can be fed-back to force the optical system into the desired condition.

### The Schnupp modulation scheme

In this and the following Sects. 8.14, 8.15 and 8.16, we introduce techniques for reading out signals from interferometers. These approaches complement and extend the Pound–Drever–Hall method for readout from Fabry–Perot cavities presented in Sect. 8.10.

Similar to the Fabry–Perot cavity, the Michelson interferometer is also often used to set an operating point where the optical gain of a direct light power detection is zero. This operating point, given by $$\varDelta
								L/\lambda =(2N+1)\cdot 0.25$$ with *N* a non-negative integer, is called *dark fringe*. This operating point has several advantages, the most important being the low (ideally zero) light power on the diode. Highly efficient and low-noise photodiodes usually use a small detector area and thus are typically not able to detect large power levels. By using the dark fringe operating point, the Michelson interferometer can be used as a *null instrument* or *null measurement*, which generally is a good method to reduce systematic errors (Saulson 1994).

One approach to make use of the advantages of the dark fringe operating point is to use an operating point very close to the dark fringe at which the optical gain is not yet zero. In such a scenario a careful trade-off calculation can be done by computing the signal-to-noise with noises that must be suppressed, such as the laser amplitude noise. This type of operation is usually referred to as *DC control* or *offset control* and is very similar to the similarly-named mechanism used with Fabry–Perot cavities.

**Fig. 70:**
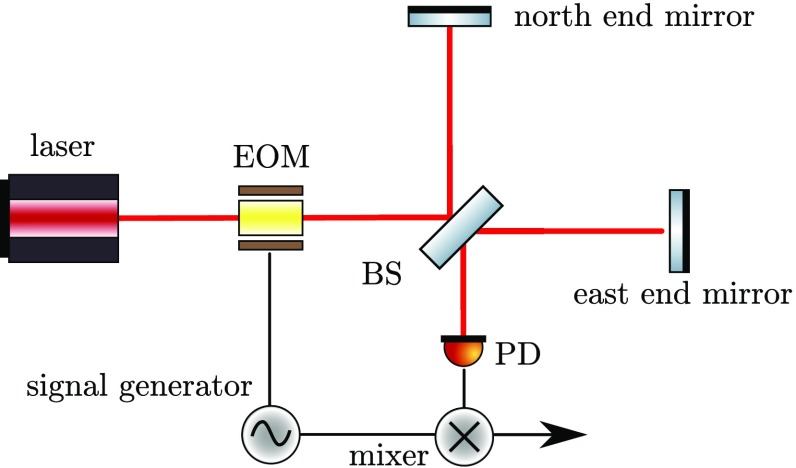
This length sensing scheme is often referred to as *frontal* or *Schnupp modulation*: an EOM is used to phase modulate the laser beam before entering the Michelson interferometer. The signal of the photodiode in the South port is then demodulated at the same frequency used for the modulation

Another option is to employ phase modulated light, similar to the Pound–Drever–Hall scheme described in Sect. 8.10. The optical layout of such a scheme is depicted in Fig. 70: an electro-optical modulator is used to apply a phase modulation at a fixed frequency, usually in the RF range, to the monochromatic laser light before it enters the interferometer. The photodiode signal from the interferometer output is then demodulated at the same frequency. This scheme allows one to operate the interferometer precisely on the dark fringe. The method originally proposed by Lise Schnupp is also sometimes referred to as *frontal modulation*.

The optical gain of a Michelson interferometer with Schnupp modulation is shown in Fig. 75 in Sect. 8.17.

### Extending the Pound–Drever–Hall technique to more complicated optical systems

To recap Sect. 8.10, in the Pound–Drever–Hall (PDH) or RF-reflection locking technique sinusoidal radio-frequency phase modulation is applied to the light to produce phase modulation sidebands. With phase modulation, higher order sidebands are imposed on the light, though the beats due to these are generally not employed in the normal implementation of the Pound–Drever–Hall technique. The light is then incident on the cavity that is to be controlled. The signal is obtained by detecting the reflected light on a photo-detector which has a square-law response to the light amplitude, and analysing the resulting beats. The important beats are between the carrier and the first order RF sidebands. The electronic signal from the photodiode is filtered to pass the beats in a frequency range around the modulation frequency, and multiplied or ‘mixed’ with an electronic signal at the modulation frequency: an electronic local oscillator. The output from the mixer is then low-pass filtered to remove oscillations at harmonics of the modulation frequency. The useful signal is in one quadrature of the output from the photo-detector at the modulation frequency. The phase of the local oscillator is chosen to select the required quadrature.

During the 1980s and 1990s, the question arose of how to obtain control signals for systems of coupled cavities and systems with combination of cavities in a Michelson interferometer. A good example is the power-recycled Fabry–Perot–Michelson interferometer configuration as employed in initial LIGO and Virgo. In such a system, one possibility is to add pick-offs (low-reflectivity beam splitters) to remove some of the light reflected from each arm cavity for detection. This approach introduces a conflict between efficient power recycling that requires low loss, and the generation of a low-noise control signal, which argues for more highly reflecting beam splitters. It is of interest to identify other approaches that do not require additional detection ports. With this restriction, the problem becomes one of sensing all internal degrees of freedom by analysing light fields reflected from or transmitted by the entire interferometer. This has been accomplished for the dual-recycled Michelson topology of GEO 600 (Grote 2003), for the dual-recycled Fabry–Perot–Michelson configuration, e.g., Advanced LIGO—see “Appendix B” for a short description of the sensing scheme.

The scope of this section permits discussion only of design principles. It is worth noting, however, some practical matters that constrain the acceptable solutions. For example, the choice of modulation frequencies is usually restricted. One limit is the speed of photo-detectors, and in particular quadrant photo-detectors, for alignment sensing. This restriction makes the use of modulation frequencies above of order 100 MHz highly challenging. Another limitation results from the presence of mode-cleaning cavities in the path from the laser to the interferometer. Due to practical difficulties in the design of in-vacuum modulators, modulation is usually applied prior to the light passing the mode-cleaner. In this case the only available modulation frequencies are whole-multiples of the free-spectral-range of the mode-cleaner. Note however that in-vacuum modulation is possible, and has been applied in GEO 600 allowing a relatively free choice of modulation frequency which is important for the method of locking the dual recycled system (Grote 2003).

The essence of the Pound–Drever–Hall method is that the light field is divided, according to frequency, into a component that suffers a phase change in response to variation of the target degree of freedom for measurement, and a component that does not. Therefore, a starting point in the discovery of alternatives is to create circumstances in which different light components, distinguished by frequency, resonate in different locations. Secondly, to produce a useful error signal, the output from the detection process should contain a dominant linear component in terms of its magnitude as a function of the target degree of freedom. Although it is desirable that the signal crosses zero at the operating point, it may be necessary and acceptable to subtract a (hopefully steady) offset to obtain the required result. These aspects are dealt with in turn.

First we consider how zero-crossing signals may be obtained from beats. The desired zero-crossing linear slope is achieved most directly if the components of the light are in quadrature, as is the case in Pound–Drever–Hall sensing: see Sects. 3.2 and 8.10. This ensures that the measurement depends on the relative phase of the optical field components, rather than their amplitudes. As an example of an alternative, quadrature is also achieved in the case of beating amplitude modulation sidebands against phase modulation sidebands.

In cases like this, where beats are obtained between various sidebands, rather than by beating with the carrier, the demodulated signal may either be obtained directly by mixing the electronic signal with a local oscillator at the beat frequency, or by employing double demodulation. A description of this process is shown in Sect. 4.2.

The condition for quadrature requires pairs of sidebands to be symmetrical so that they represent either pure phase modulation or pure amplitude modulation. In either of these cases, their resultant sum maintains a constant phase over time. If there is an imbalance of the amplitude of the lower and upper sidebands, the phase of the resultant must oscillate. This is equivalent to saying that the sidebands represent a mixture of amplitude and phase modulation, or equivalently, that there is an unbalanced single-sideband component. Extraction of useful error signals is still possible, but it is to be expected that there will be an offset in the demodulated signal, rather than a zero-crossing at the desired resonance condition.

Such sideband imbalance arises naturally in interferometers with detuned signal recycling, see Sect. 7.3. In these interferometers, the resonance of the signal recycling cavity is not centered on the carrier and so the response to upper and lower modulation sidebands can be expected to be asymmetrical. The beats produced on detection of the unbalanced sidebands may still produce a useful linear component, corresponding to the part of the amplitude that is in the appropriate quadrature.

As an example of obtaining signals from beats between sidebands, we cite the important method called *third-harmonic demodulation*, introduced and explained in detail in Arai et al. (2000). In brief summary, this technique exploits the natural presence of higher harmonics in phase modulation for moderate to large modulation indices, e.g., 0.8 rad in the cited work. As noted above, such harmonics are passed by a mode-cleaner that is resonant at the first harmonic, and depending on the design of the interferometer, at least some can be expected to be resonant in the power recycling cavity (the odd members of the series in the scheme described by Arai et al. (2000). By combining this method of demodulation with the introduction of asymmetry in the geometry of the interferometer, as described in the following section, it is possible to construct a sensing system that provides well separated readout of the various degrees of freedom. In the cited scheme, neither the first or third order sidebands are strongly affected by the phase of the arm cavities (when the carrier is on resonance), and the method allows relatively independent control of the other degrees of freedom.

The third harmonic demodulation approach has been extended, with results proven in a series of investigations on prototype interferometers, including a 4 m interferometer with resonant sideband enhancement (Kawazoe et al. 2006), and experiments on the CalTech 40 m apparatus (Miyakawa et al. 2006) as part of the development of control systems for Advanced LIGO, in which third-harmonic demodulation is employed—see “Appendix B”.

Next we return to the question of how sideband fields may be separated by breaking the symmetry of the interferometer. To reduce noise couplings, interferometers are usually designed and built to be as symmetrical as possible. For instance, an interferometer with perfectly matched arms is insensitive to the frequency of the light. In the design process it is usually assumed that the long arms of the interferometer must be kept as symmetrical as can be arranged in practice, but that controlled amounts of asymmetry can be introduced in the paths from the beam splitter to the arm cavities or recycling mirrors as appropriate to facilitate the design of sensing schemes.

The methods discussed in this section stem from the Schnupp modulation technique described in Sect. 8.13. In the unmodified Michelson interferometer, shown in Fig. 70, the asymmetry required to maximise the strength of the sidebands at the output, with modulation frequencies in the usual range (typically 10–100 MHz) is one quarter of the RF wavelength. The addition of power-recycling lowers the required asymmetry because in this case optimum transfer of sideband power occurs when the asymmetry leads to an out-coupling of equal strength to the transmission of the power recycling mirror. This is in direct analogy with the transmission of light through an equal-mirror Fabry–Perot cavity.

An example of this ‘classical’ application of Schnupp modulation is found in GEO 600. Here the approximately 1200 m (optical path) arms are adjusted to differ in length by about 10 cm, and this provides efficient transfer of $${\approx }
								15$$ MHz sidebands to the output port. The approach is described in Grote (2003).

The idea of Schnupp modulation influenced the development of Advanced LIGO see, for example, Strain et al. (2003). It had been decided that phase modulation would be applied prior to the in-vacuum mode-cleaner, thus constraining the modulation sidebands to fall in a harmonic series. A detailed description of these methods is beyond the scope of this review, but some important features are described below.

The objective is always to cause distinct modulation sidebands to resonate in different physical regions within the interferometer. In a dual-recycling Fabry–Perot–Michelson configuration, it is necessary to control the (inner) Michelson, the power recycling cavity and the signal recycling cavity. Controlling the arm cavities may be achieved by beating the carrier with suitable sidebands, the hard part of the problem is to remove the influence of arm cavities on signals for the other degrees of freedom. For control of the signal recycling cavity, for example, at least one sideband must be directed towards the signal recycling mirror. This can be accomplished by choosing a difference in the lengths of the two arms of the Michelson to arrange that one sideband is on a bright fringe, and therefore strongly directed towards the signal recycling mirror. For further detail of this aspect of interferometer sensing, see Strain et al. (2003) and “Appendix B”.

One last design ingredient is that, in a ‘closed’ configuration like the dual-recycling Fabry–Perot–Michelson, light travelling back from one of the arms ‘sees’ another (effective) Michelson interferometer formed by the beam splitter and the two recycling mirrors. A variation of the Schnupp technique can also be applied in that case, by adjusting the optical paths from the beam splitter to the recycling mirrors to be unequal. This provides further control over sideband resonance conditions in the various parts of the interferometer.

It can be appreciated that the design problem rapidly becomes too complex for a full description in this review, but all of the main principles are included, and numerical calculation allows these principles to be developed into a complete sensing scheme.

### Complementary techniques: internal modulation, external modulation and dithering

**Fig. 71:**
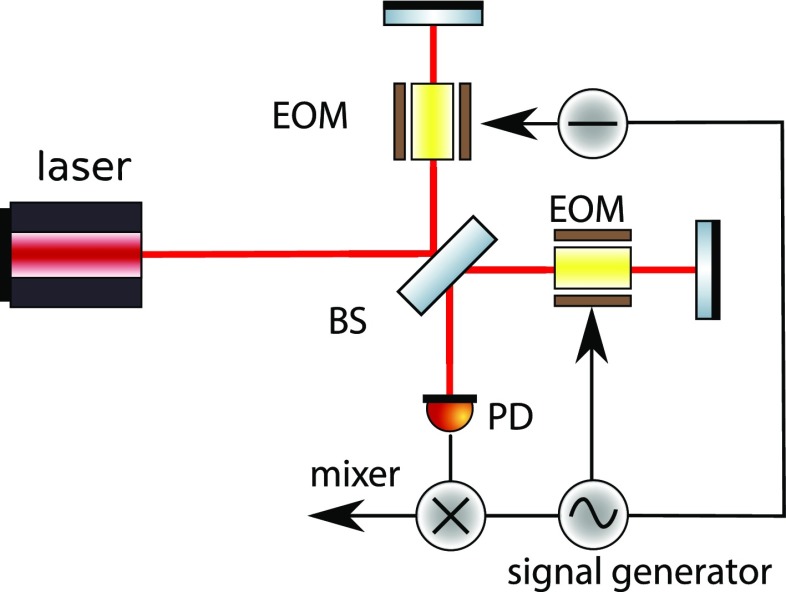
Michelson interferometer with internal modulation. Phase modulators are placed in the arms of the interferometer and driven sinusoidally in opposing phase at a radio-frequency. The strength of the modulation is chosen such that the light field at the output of the interferometer, at the *dark fringe* is strongly dominated by the modulation sidebands. Since the sidebands are applied differentially, they appear predominantly at the anti-symmetric port when the interferometer output is at the *dark fringe* for the carrier light. If, as shown here, the light passes the modulators in both directions, the position of the modulators and the frequency chosen must be taken into account to avoid unwanted cancellation or enhancement of the effect

For completeness we review a range of methods that have been applied in interferometry for gravitational wave detection. The ideas follow on from the basic RF heterodyne methods introduced in Sect. 5.4. The first RF-modulation based signal readout scheme for a Michelson interferometer involved generating the RF sidebands in phase modulators placed into the arms of the interferometer, as shown in Fig. 71.

Although this technique, called *internal modulation* was shown to be successful in interferometry up to the late 1980s (Shoemaker et al. 1988), it has not been possible to devise an implementation that operates with the low noise levels required for modern detectors. A related concept, dithering of interferometer mirrors to phase modulate the light within the interferometer, is described below. See also Sect. 3.8.2.

**Fig. 72:**
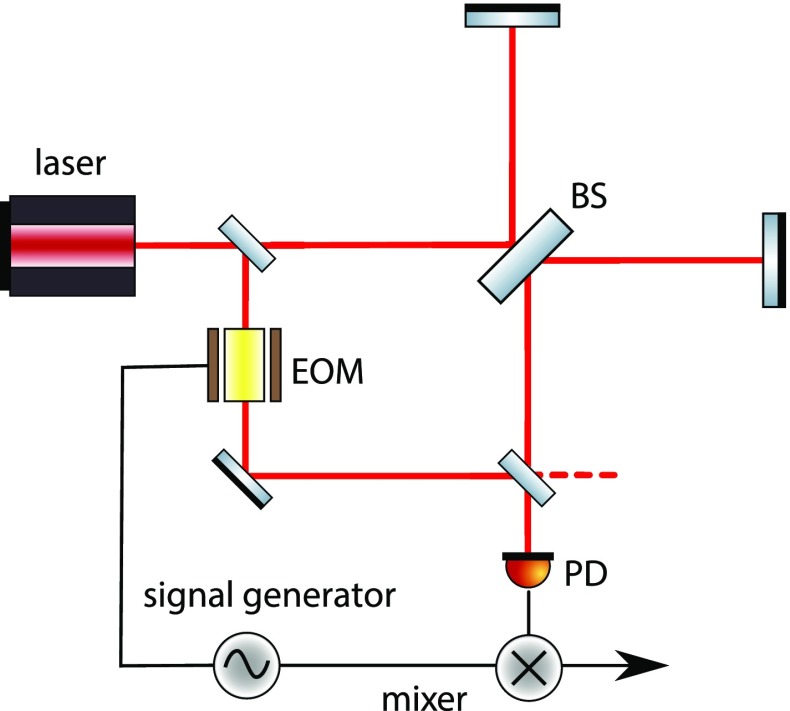
Michelson interferometer with external modulation. In this version of external modulation, a sample of the in-going light is picked off, phase modulated and recombined with the light emerging from the anti-symmetric port in a Mach–Zehnder arrangement. In an interferometer with power recycling, the light to be modulated may be extracted from within the power-recycling cavity, where the filtering action of the cavity may render it more stable. As with internal modulation, the sidebands should dominate the detected light. In that case to improve efficiency and minimise the amount of light that is extracted from the power recycling cavity, detectors may be placed at both ports of the Mach–Zehnder interferometer, and the resulting signals subtracted prior to demodulation. In the case of external modulation, the path-lengths involved are normally small compared to the RF wavelength

In the technique of *external modulation* (Man et al. 1990), a phase modulated field is derived from the common mode light within the interferometer as shown in Fig. 72. Light picked-off from a convenient location, usually close to the beam splitter or even at its imperfectly anti-reflection coated rear surface, is phase modulated and recombined with the main output field, by means of a second beam splitter. This Mach–Zehnder interferometer geometry is distinguished from general heterodyne methods in that, when power recycling is present, the modulated field is obtained from within the power recycling cavity, where the light field may be more stable than the ingoing light, due to the passive filtering provided by the power recycling cavity. External modulation adds significant complexity to the output optics of an interferometer, and is disfavoured in advanced interferometers where the application of squeezed light is considered.

Another approach to the generation of suitable signals is *dithering*, this is, effectively, the application of phase modulation sidebands by modulating parameters of the system, usually the positions or angles of mirrors, rather than modulating the ingoing light. In principle, dithering could be applied at distinct frequencies to as many components of the system as there are degrees of freedom requiring to be controlled.

There are practical limitations that restrict the application of dithering, and it is normally applied to lock auxiliary degrees of freedom where the signal to noise requirements are less severe. The limitations arise because dithering is commonly applied by mechanical means, resulting in restricted actuation force (to avoid either causing damage or adding noise due from powerful actuators). This imposes a limit to the product of imposed displacement and (dither-) frequency-squared, resulting in typical dither frequencies that do not exceed a few kHz. Dithering is, therefore, typically employed to monitor and control slowly varying aspects of the interferometer. A relatively recent application of dithering is in locking an output mode-cleaner for use with DC readout. This is discussed in the following section and in Ward et al. (2008).

### Circumstances in which offset locking is favoured over modulationbased techniques

As mentioned in Sects. 5.4 and 8.13, the idea of offset-locking of Michelson interferometers to produce a zero-crossing error signal for the differential displacement arises naturally. There are, however, disadvantages associated with this method of readout, and it has only become favoured over heterodyne methods due to particular circumstances that associated with recently developed interferometer designs, as explained below.

In a simple Michelson interferometer, the steepest gradient in the length to intensity transfer function occurs half-way-up the fringe. However, operating in this condition has two disadvantages: half of the light is directed back towards the laser and sensitivity to laser power fluctuations is maximised. The latter problem can be ameliorated by symmetrising the readout through the addition a photo-detector for the reflected light. On subtracting the signals from the detectors at the two ports of the interferometer, the displacement signals add while laser power fluctuations cancel, to the extent that balance is achieved. In this case, however, all the light is detected and there is no possibility to take advantage of low-loss optics by adding power recycling.

A further problem when a simple Michelson is offset-locked is that the optical local oscillator for the measurement is a relatively noisy component of the light field. Indeed this last concern led to the choice of radio frequency modulation in the Pound–Drever–Hall and other techniques described above. In those techniques modulation frequencies are chosen to fall at Fourier frequencies where technical laser noise is less than shot noise in the detected light power. This is typically true above about 10 MHz for detection of the tens of mW of light from the argon-ion or Nd:YAG lasers typically employed.

During the design of Enhanced and Advanced LIGO, Advanced Virgo and GEO-HF, three motivations emerged to prompt reconsideration of offset-locking methods. As noted in Sect. 3.1 it had been shown that modulation generally worsens shot-noise limited performance, and these arguments were extended to show that it is impractical to benefit from squeezed light in modulation based readout (Buonanno et al. 2003). Secondly, it was realised that, for the interferometer to achieve the planned sensitivity, the light within the power recycling cavity in a system such as Advanced LIGO, must be more stable than the best available RF oscillators, at Fourier frequencies of interest, and so the arguments against employing this light for signal readout scheme become moot. Finally, whether the detected light amplitude is shot noise limited or not depends on the power that is detected, because the shot noise in the detection of small light power can make technical noise unimportant. It was realised that, by adding a mode-cleaner on the output of the interferometer, to pass the signal, which would predominantly be in the $$\mathrm
								{TEM}_{00}$$ mode of the arms, but exclude other light resulting from imperfect interference, mainly in other modes, it would suffice to detect relatively low light power, at which level the measurement should be shot noise limited. See Sect. 10 for a description of modes resulting from imperfect interference.

In modern detectors this scheme, where signals are read out directly in the base-band i.e., near zero frequency or ‘DC’, is often called *DC readout*. As an example of its application, the details of the DC readout scheme developed for Advanced LIGO are described in Ward et al. (2008). The technique has also been tested on GEO 600, where the method has been shown to be compatible with squeezing (LIGO Scientific Collaboration 2011).

It should be noted that offset locking applies to the control of one length degree of freedom per interferometer, and the remaining degrees of freedom are typically sensed using the modulation methods described above.

### Finesse examples

#### Michelson modulation

**Fig. 73:**
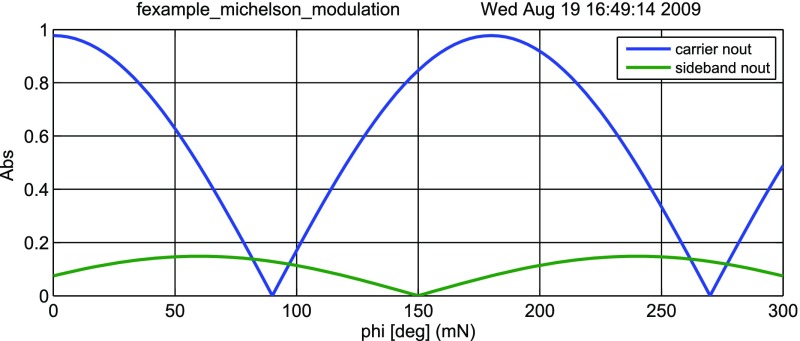
Finesse example: Michelson modulation

This example demonstrates how a macroscopic arm length difference can cause different ‘dark fringe’ tuning for injected fields with different frequencies. In this case, some of the 10 MHz modulation sidebands are transmitted when the interferometer is tuned to a dark fringe for the carrier light. This effect can be used to separate light fields of different frequencies. It is also the cause for transmission of laser noise (especially frequency noise) into the Michelson output port when the interferometer is not perfectly symmetric (Fig. 73).

**Finesse input file for ‘Michelson modulation’** 
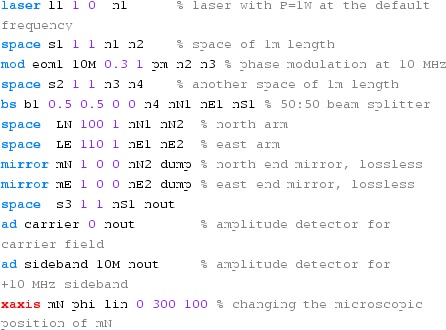


#### Cavity power and slope

**Fig. 74:**
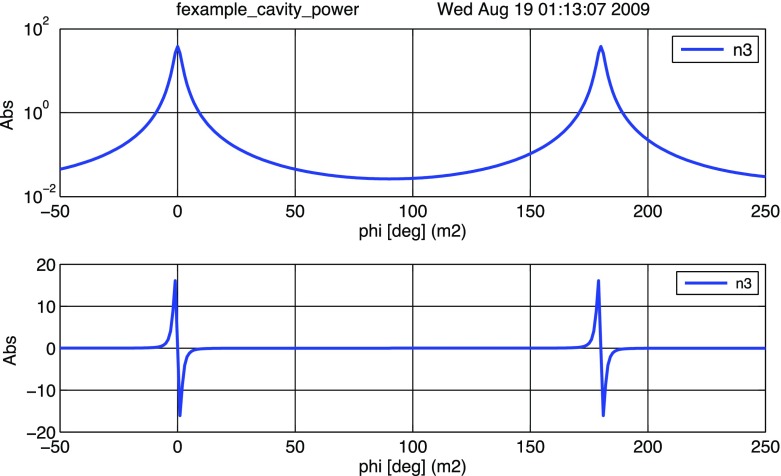
Finesse example: cavity power and slope

Figure 74 (same as Fig. 65) shows a plot of the analytical functions describing the power inside a cavity and its differentiation by the cavity tuning. This example recreates the plot using a numerical model in Finesse.

**Finesse input file for ‘Cavity power and slope’** 
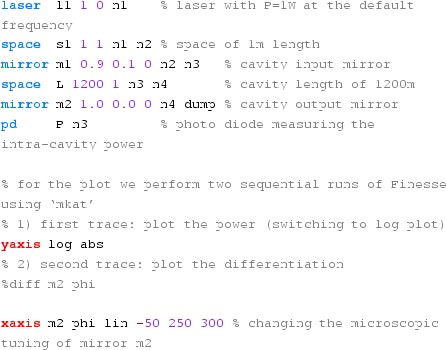


#### Michelson with Schnupp modulation

**Fig. 75:**
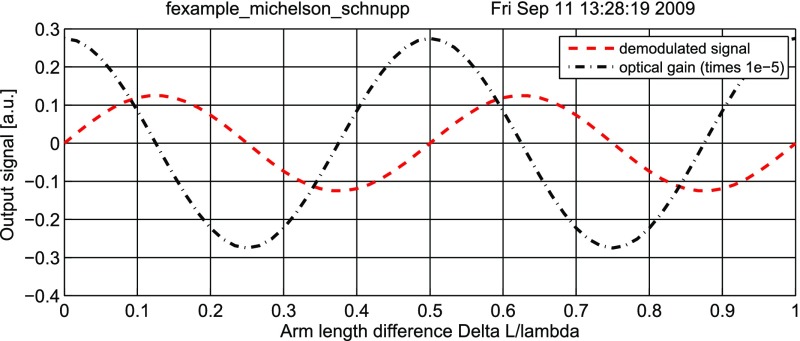
Finesse example: Michelson with Schnupp modulation

Figure 75 shows the demodulated photodiode signal of a Michelson interferometer with Schnupp modulation, as well as its differentiation, the latter being the optical gain of the system. Comparing this figure to Fig. 68, it can be seen that with Schnupp modulation, the optical gain at the dark fringe operating points is maximised and a suitable error signal for these points is obtained.

**Finesse input file for ‘Michelson with Schnupp modulation’** 
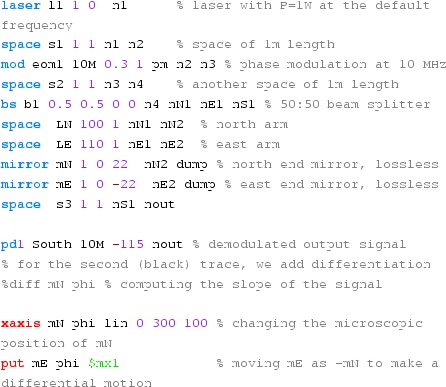


## Beam shapes: beyond the plane wave approximation

In previous sections we have introduced a notation for describing the on-axis properties of electric fields. Specifically, we have described the electric fields along an optical axis as functions of frequency (or time) and the location *z*. Models of optical systems may often use this approach for a basic analysis even though the respective experiments will always include fields with distinct off-axis beam shapes. A more detailed description of such optical systems needs to take the geometrical shape of the light field into account. One method of treating the transverse beam geometry is to describe the spatial properties as a sum of ‘spatial components’ or ‘spatial modes’ so that the electric field can be written as a sum of the different frequency components and of the different spatial modes. Of course, the concept of modes is directly related to the use of a sort of oscillator, in this case the optical cavity. Most of the work presented here is based on the research on laser resonators reviewed originally by Kogelnik and Li (1966). Siegman has written a very interesting historic review of the development of Gaussian optics (Siegman 2000a, b) and we use whenever possible the same notation as used in his textbook ‘Lasers’ (Siegman 1986).

This section introduces the use of Gaussian modes for describing the spatial properties along the transverse orthogonal *x* and *y* directions of an optical beam. We can write

9.1 $$\begin{aligned}
							E(t,x,y,z)=\sum _{j}~\sum _{n,m}~a_{jnm}~u_{nm}(x,y,z)~\exp {\left(
							\mathrm {i}\,(\omega _j \,t-k_j z)\right) },
							\end{aligned}$$

with $$u_{nm}$$ as special functions describing the spatial properties of the beam and $$a_{jnm}$$ as complex amplitude factors ($$\omega
							_j$$ is again the angular frequency and $$k_j=\omega
							_j/c$$). For simplicity we restrict the following description to a single frequency component at one moment in time ($$t=0$$), so

9.2 $$\begin{aligned}
							E(x,y,z)=\exp {\left( -\mathrm {i}\,k z\right) }~\sum
							_{n,m}~a_{nm}~u_{nm}(x,y,z). \end{aligned}$$

In general, different types of spatial modes $u_{nm}$ can be used in this context. Of particular interest are the *Gaussian modes*, which will be used throughout this document. Many lasers emit light that closely resembles a *Gaussian beam*: the light mainly propagates along one axis, is well collimated around that axis and the cross section of the intensity perpendicular to the optical axis shows a Gaussian distribution. The following sections provide the basic mathematical framework for using Gaussian modes for analysing optical systems.

### A typical laser beam: the fundamental Gaussian mode

The beam produced from a real laser is not a plane wave, but has some intensity distribution. This is typically a roughly circular beam with a peak brightness near the centre. The intensity pattern of a beam generated by an ideal laser based on a stable optical cavity with spherical mirrors would resemble a Gaussian beam. Figure 76 shows the intensity and amplitude distribution of a typical Gaussian beam, often characterised by the beam spot size, *w*, the radius within which $${\sim
								}86\,\%$$ ($$\frac{1}{e^2}$$) of the light power is contained. As the beam propagates the beam spot size changes slowly, which produces a narrow beam of light with a small diffraction angle.

The use of cavities in interferometry provides the basis for the mathematical description of laser beam shapes as Gaussian modes. A well designed cavity is a perfect optical resonator for a particular Gaussian mode. As discussed above, the intensity distribution can be characterised by the beam spot size, which determines the width of the beam. In the case of Gaussian modes the wavefront, or phase, of the light field is curved and can be expressed with a radius of curvature, $R_C$. As the beam propagates the curvature of the wavefront changes. To achieve perfect resonance in an optical cavity the curvature of the wavefront must match the curvature of the mirrors at their positions on the optical axis. The Gaussian beam whose curvatures match the mirrors of a cavity is known as the cavity eigenmode, see Fig. 77.

**Fig. 76:**
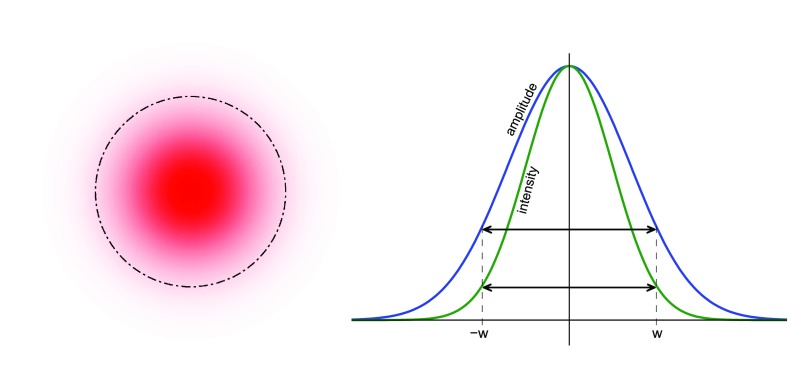
A typical laser beam intensity pattern (*left*) and the intensity and amplitude distributions of a normalised Gaussian beam (*right*). A Gaussian beam is characterised by its spot size, *w*, the radius at which the intensity falls to $$\frac{1}{e^2}$$ ($${\sim }
											14\,\%$$) of the peak intensity

**Fig. 77:**
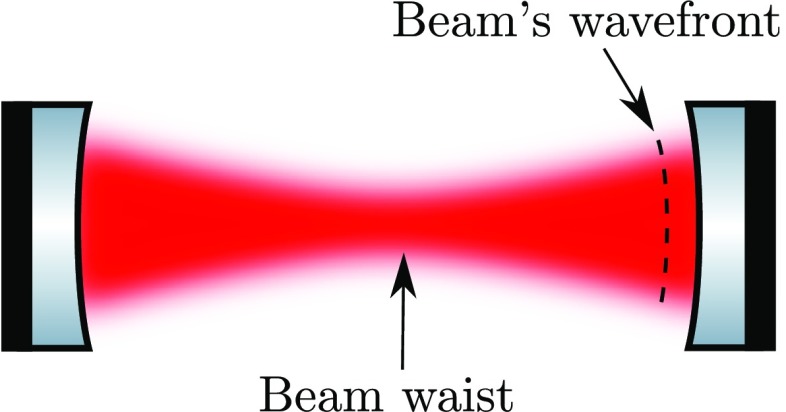
Simple depiction of a cavity eigenmode. The position and curvature of the mirrors determine the cavity eigenmode, which is defined by the beam waist size and position relative to the mirrors

### Describing beam distortions with higher-order modes

In an ideal interferometer the laser beam would be a perfect Gaussian beam, with wavefronts exactly matched to the shape of the mirrors. However, in a real interferometer mismatches between the beam and mirror curvatures, misalignments from the optical axis and deviations of the mirror surfaces from a perfect sphere all contribute to distort the beam from the ideal Gaussian beam.

Small distortions of the fundamental beam can be described by the addition of *higher-order modes*. Higher-order modes have the same basic properties of the fundamental Gaussian beam, with two exceptions: higher-order modes have different intensity patterns from the simple spot of the fundamental mode and modes of different order pick up an extra phase upon propagation (the Gouy phase, see Sect. 9.10).

**Fig. 78:**
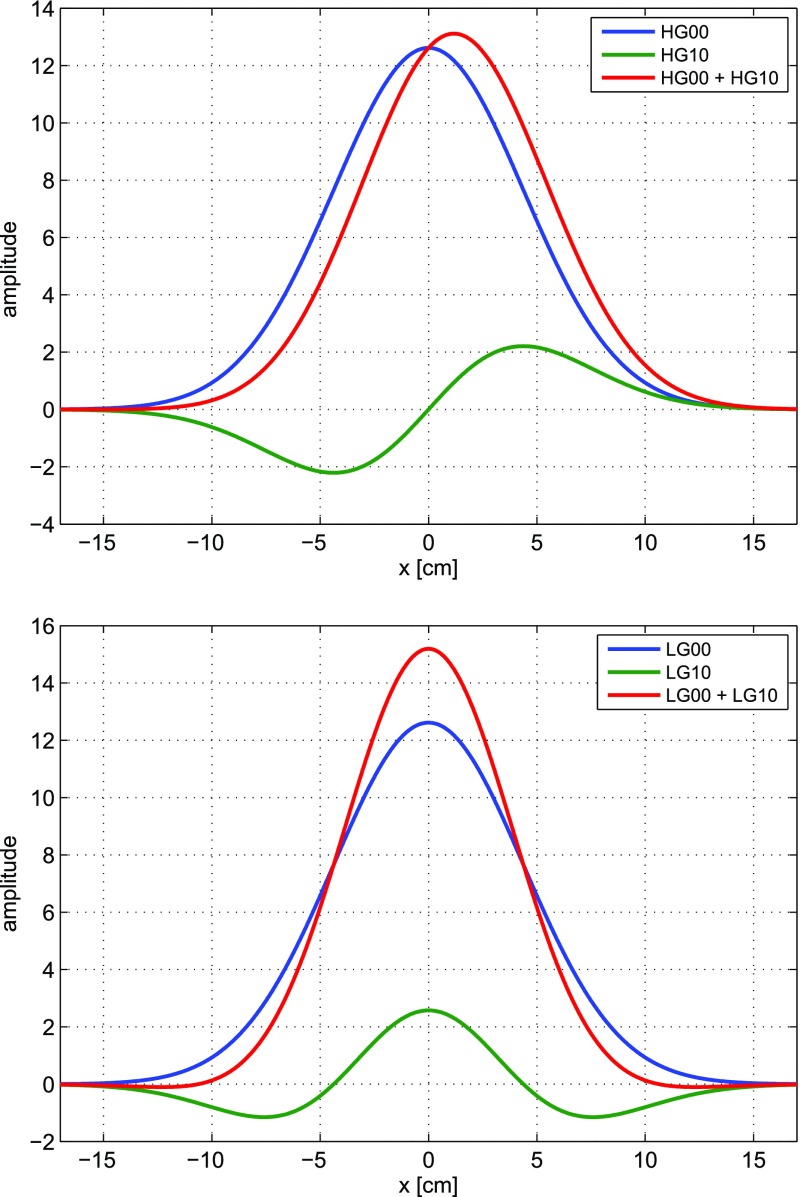
*Left* Amplitude distributions of a fundamental gaussian beam (HG$_{00}$), order 1 Hermite–Gauss beam (HG$_{10}$) and the sum of the two modes. The resulting sum is a good description of a misaligned fundamental beam. The total power is 1 W with 4 % power in the order 1 mode. *Right* Amplitude distributions of a fundamental gaussian beam (LG$_{00}$), order 2 Laguerre–Gauss beam (LG$_{10}$) and the sum of the two modes. The resulting sum is a good description of a fundamental gaussian beam with a smaller beam spot size. The power in the order 2 mode is 4 % of the total 1 W power

One simple example is a misaligned beam, whose centre has been shifted from the optical axis. This can be described by the addition of an order ‘1’ Hermite–Gauss mode, HG$_{10}$ (Sect. 9.7), as illustrated in the left panel of Fig. 78. Such a distortion is a first order effect and, as long as the misalignment is small, can be described with just this one additional mode. In a similar way the second order effect such as a mismatch in beam size can be described by the addition of a single order ‘2’ mode, in this case the Laguerre–Gauss mode LG$_{10}$ (Sect. 9.11). A mismatch in beam size is illustrated in the right panel of Fig. 78.

The following sections describe details of Gaussian modes and how any paraxial laser beam with distortions can be described by a sum of Gaussian modes.

### The paraxial approximation

All electromagnetic waves are solutions to the general wave equation (Helmholtz equation), which in vacuum can be given as:

9.3 $$\begin{aligned}
								\varDelta \mathbf {E}-\frac{1}{c^2}\ddot{\mathbf {E}}=0.
								\end{aligned}$$

Mathematically, Gaussian modes represent solutions to the *paraxial approximation* of this equation. Laser light fields are a special class of electromagnetic waves. A laser beam will have a characteristic size *w* describing the ‘width’ (the dimension of the field transverse to the main propagation axis), and a characteristic length *l* defining some local length along the propagation over which the beam characteristics do not vary much. By definition, for what we call a *beam* *w* is typically small and *l* large in comparison, so that *w* / *l* can be considered small. In fact, the paraxial wave equation (and its solutions) can be derived as the first-order terms of a series expansion of Eq. (9.3) into orders of *w* / *l* (Lax et al. 1975).

A simpler approach to the paraxial-wave equation goes as follows: a particular beam shape shall be described by a function *u*(*x*, *y*, *z*) so that we can write the electric field as

9.4 $$\begin{aligned}
								E(x,y,z)=u(x,y,z)~\exp {\left( -\mathrm {i}\,kz\right) }.
								\end{aligned}$$

Substituting this into the standard wave equation yields a differential equation for *u*:

9.5 $$\begin{aligned}
								\left( \partial _x^2+\partial _y^2+\partial _z^2\right)
								u(x,y,z)-2\mathrm {i}\,k\partial _z u(x,y,z)=0.
								\end{aligned}$$

Now we put the fact that *u*(*x*, *y*, *z*) should be slowly varying with *z* in mathematical terms. The variation of *u*(*x*, *y*, *z*) with *z* should be small compared to its variation with *x* or *y*. Also the second partial derivative in *z* should be small. This can be expressed as

9.6 $$\begin{aligned}
								\left| \partial _z^2 u(x,y,z)\right| \ll \left| 2k\partial
								_zu(x,y,z)\right| ,\left| \partial _x^2 u(x,y,z)\right| ,\left|
								\partial _y^2 u(x,y,z)\right| .
								\end{aligned}$$

With this approximation, Eq. (9.5) can be simplified to the *paraxial wave equation*,

9.7 $$\begin{aligned}
								\left( \partial _x^2+\partial _y^2\right) u(x,y,z)-2\mathrm
								{i}\,k\partial _z u(x,y,z)=0.
								\end{aligned}$$

Any field *u* that solves this equation represents a paraxial beam shape when used in the form given in Eq. (9.4).

### Transverse electromagnetic modes

In general, any solution *u*(*x*, *y*, *z*) of the paraxial wave equation, Eq. (9.7), can be employed to represent the transverse properties of a scalar electric field representing a beam-like electro-magnetic wave. Especially useful in this respect are special *families* or *sets* of functions that are solutions of the paraxial wave equation. When such a set of functions is complete and countable, it is called a set of *transverse electromagnetic modes* (TEM). For instance, the set of *Hermite–Gauss modes* are exact solutions of the paraxial wave equation. These modes are represented by an infinite, countable and complete set of functions. The term *complete* means they can be understood as a base system of the function space defined by *all* solutions of the paraxial wave equation. In other words, we can describe any solution of the paraxial wave equation $u'$ by a linear superposition of Hermite–Gauss modes:

9.8 $$\begin{aligned}
								u'(x,y,z)=\sum _{n,m}~a_{jnm}~u_{nm}(x,y,z),
								\end{aligned}$$

which in turn allows us to describe any laser beam using a sum of these modes:

9.9 $$\begin{aligned}
								E(t,x,y,z)=\sum _{j}~\sum _{n,m}~a_{jnm}~u_{nm}(x,y,z)~\exp {\left(
								\mathrm {i}\,(\omega _j \,t-k_j z)\right) }.
								\end{aligned}$$

The Hermite–Gauss modes as given in this document (see Sect. 9.7) are orthonormal so that

9.10 $$\begin{aligned}
								\int \!\!\!\int \!dx\,dy~u_{n m}u^*_{n' m'}=\delta _{n n'}\delta _{m
								m'}=\left\{ \begin{array}{l@{\quad }l} 1&{}\mathrm {if}\quad
								n=n'\quad \mathrm {and}\quad m=m'\\ 0&{}\mathrm {otherwise}
								\end{array}\right\} . \end{aligned}$$

This means that, in the function space defined by the paraxial wave equation, the Hermite–Gauss functions can be understood as a complete set of *unit-length* basis vectors. This fact can be utilised for the computation of coupling factors, as shown in Sect. 11.3. Furthermore, the power of a beam, as given by Eq. (9.2), being detected on a single-element photodetector (provided that the area of the detector is large with respect to the beam) can be computed as

9.11 $$\begin{aligned}
								EE^*=\sum _{n,m} a_{nm}a_{nm}^*,
								\end{aligned}$$

or for a beam with several frequency components [compare with Eq. (4.9)] as

9.12 $$\begin{aligned}
								EE^*=\sum _{n,m}\sum \limits _i\sum \limits _j a_{inm}a_{jnm}^*\quad
								\mathrm {with}\quad \{i,j~|~i,j\in \{0,\ldots ,N\}~\wedge ~\omega
								_i=\omega _j\}.\quad \end{aligned}$$

### Properties of Gaussian beams

The basic or ‘lowest-order’ Hermite–Gauss mode is equivalent to what is usually called a *Gaussian beam* and is given by

9.13 $$\begin{aligned}
								u(x,y,z)=\sqrt{\frac{2}{\pi }}~\frac{1}{w(z)}~\exp {\left( \mathrm
								{i}\,\varPsi (z)\right) }~ \exp {\left( -\mathrm
								{i}\,k\frac{x^2+y^2}{2R_C(z)}-\frac{x^2+y^2}{w^2(z)}\right) }.
								\end{aligned}$$

The parameters of this equation are explained in detail below. The shape of a Gaussian beam is quite simple: the beam has a circular cross section, and the radial intensity profile of a beam with total power *P* is given by

9.14 $$\begin{aligned}
								I(r)=\frac{2P}{\pi w^2(z)}\exp {\left( -2r^2/w^2\right) },
								\end{aligned}$$

with *w* the *spot size*, defined as the *radius* at which the intensity is $1/e^2$ times the maximum intensity *I*(0). This is a Gaussian distribution, see Fig. 79, hence the name *Gaussian beam*.

**Fig. 79:**
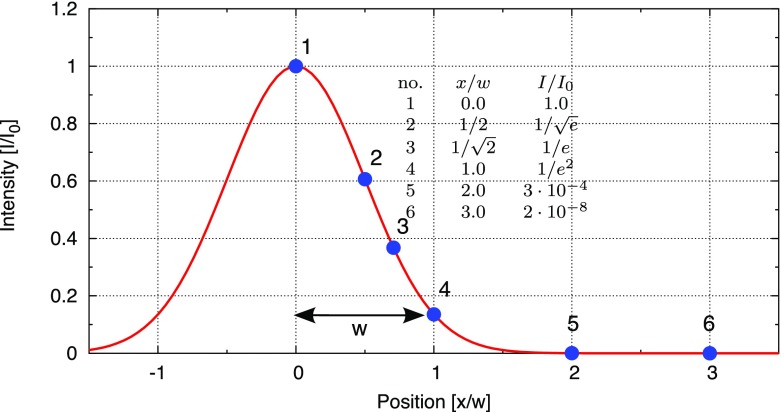
One dimensional cross-section of a Gaussian beam. The width of the beam is given by the radius *w* at which the intensity is $1/e^2$ of the maximum intensity

Figure 80 shows a different cross section through a Gaussian beam: it plots the beam size as a function of the position on the optical axis. Such a beam profile (for a beam with a given wavelength $$\lambda
								$$) can be completely determined by two parameters: the size of the minimum spot size $$w_0$$ (called the *beam waist*) and the position $$z_0$$ of the beam waist along the *z*-axis.

**Fig. 80:**
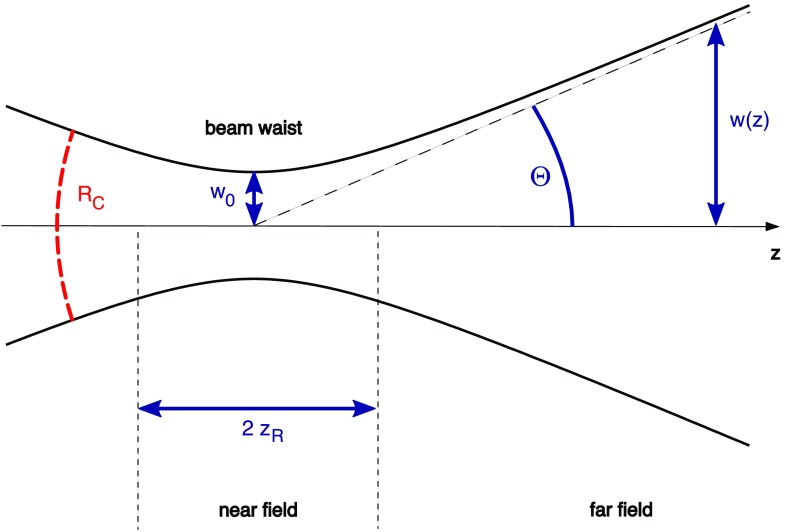
Gaussian beam profile along z: this cross section along the *x*–*z*-plane illustrates how the beam size *w*(*z*) of the Gaussian beam changes along the optical axis. The position of minimum beam size $w_0$ is called *beam waist*. See text for a description of the parameters $\Theta$, $z_R$ and $R_c$

To characterise a Gaussian beam, some useful parameters can be derived from $$w_0$$ and $$z_0$$. A Gaussian beam can be divided into two different sections along the *z*-axis: a *near field*—a region around the beam waist, and a *far field*—far away from the waist. The length of the near-field region is approximately given by the *Rayleigh range* $$z_{\mathrm
								{R}}$$. The Rayleigh range and the spot size are related by

9.15 $$\begin{aligned}
								z_{\mathrm {R}}=\frac{\pi w_0^2}{\lambda }.
								\end{aligned}$$

With the Rayleigh range and the location of the beam waist, we can usefully write

9.16 $$\begin{aligned}
								w(z)=w_0\sqrt{1+\left( \frac{z-z_0}{z_{\mathrm {R}}}\right) ^2}.
								\end{aligned}$$

This equation gives the size of the beam along the *z*-axis. In the far-field regime ($$z\gg z_{\mathrm
								{R}},z_0$$), it can be approximated by a linear equation, when

9.17 $$\begin{aligned}
								w(z)\approx w_0\frac{z}{z_{\mathrm {R}}}=\frac{z \lambda }{\pi w_0}.
								\end{aligned}$$

The angle $$\Theta
								$$ between the *z*-axis and *w*(*z*) in the far field is called the *diffraction angle* 13 and is defined by

9.18 $$\begin{aligned}
								\Theta =\arctan \left( \frac{w_0}{z_{\mathrm {R}}}\right) =\arctan
								\left( \frac{\lambda }{\pi w_0}\right) \,\,\approx
								\frac{w_0}{z_{\mathrm {R}}}.
								\end{aligned}$$

Another useful parameter is the *radius of curvature* of the wavefront at a given point *z*. The radius of curvature describes the curvature of the ‘phase front’ of the electromagnetic wave—a surface across the beam with equal phase—intersecting the optical axis at the position *z*. We obtain the radius of curvature as a function of *z*:

9.19 $$\begin{aligned}
								R_C(z)=z-z_0+\frac{z_{\mathrm {R}}^2}{z-z_0}.
								\end{aligned}$$

We also find:

9.20 $$\begin{aligned}
								{ \begin{array}{l@{\quad }l@{\quad }l} R_C\approx \infty
								,&{} z-z_0\ll z_{\mathrm {R}}\ &{} \text {(beam
								waist)}\\ R_C\approx z, &{} z\gg z_{\mathrm {R}},~ z_0
								&{} \text {(far field)}\\ R_C=2z_{\mathrm {R}}, &{}
								z-z_0=z_{\mathrm {R}}&{} \text {(maximum curvature)}.\\
								\end{array} } \end{aligned}$$

### Astigmatic beams: the tangential and sagittal plane

If the interferometer is confined to a plane (here the *x*–*z* plane), it is convenient to use projections of the three-dimensional description into two planes (Rigrod 1965): the *tangential plane*, defined as the *x*–*z* plane and the *sagittal plane* as given by *y* and *z*.

The beam parameters can then be split into two respective parameters: $z_{0,s}$, $w_{0,s}$ for the sagittal plane and $z_{0,t}$ and $w_{0,t}$ for the tangential plane so that the Hermite–Gauss modes can be written as

9.21 $$\begin{aligned}
								u_{nm}(x,y)=u_n(x,z_{0,t},w_{0,t})~u_m(y,z_{0,s},w_{0,s}).
								\end{aligned}$$

Beams with different beam waist parameters for the sagittal and tangential plane are *astigmatic*.

Remember that these Hermite–Gauss modes form a base system. This means one can use the separation into sagittal and tangential planes even if the actual optical system does not show this special type of symmetry. This separation is very useful in simplifying the mathematics. In the following, the term *beam parameter* generally refers to a simple case where $w_{0,x}=w_{0,y}$ and $z_{0,x}=z_{0,y}$ but all the results can also be applied directly to a pair of parameters.

### Higher-order Hermite–Gauss modes

The complete set of Hermite–Gauss modes is given by an infinite discrete set of modes $$u_{\mathrm
								{nm}}(x,y,z)$$ with the indices n and m as *mode numbers*. The sum $$\hbox {n}+\hbox
								{m}$$ is called the *order* of the mode. The term *higher-order modes* usually refers to modes with an order $$n+m>0$$. The general expression for Hermite–Gauss modes can be given as (Kogelnik and Li 1966)

9.22 $$\begin{aligned}
								u_{\mathrm {nm}}(x,y,z)=u_{\mathrm {n}}(x,z)u_{\mathrm {m}}(y,z),
								\end{aligned}$$

with

9.23 $$\begin{aligned}
								u_{\mathrm {n}}(x,z)= & {} \left( \frac{2}{\pi }\right)
								^{1/4}\left( \frac{\exp {\left( \mathrm {i}\,(2n+1)\varPsi
								(z)\right) }}{2^n n! w(z)}\right) ^{1/2}\nonumber \\&\times
								\, H_n\left( \frac{\sqrt{2}x}{w(z)}\right) \exp {\left( -\mathrm
								{i}\,\frac{kx^2}{2R_C(z)}-\frac{x^2}{w^2(z)}\right) },
								\end{aligned}$$

and $H_n(x)$ the Hermite polynomials of order n. The first Hermite polynomials, without normalisation, can be written

9.24 $$\begin{aligned}
								H_0(x)= & {} 1, \quad H_1(x)=2x\nonumber \\ H_2(x)=
								& {} 4x^2-2,\quad H_3(x)=8x^3-12x.
								\end{aligned}$$

Further orders can be computed recursively since

9.25 $$\begin{aligned}
								H_{n+1}(x)=2xH_n(x)-2nH_{n-1}(x).
								\end{aligned}$$

For both transverse directions we can also rewrite the above to

9.26 $$\begin{aligned}
								u_{\mathrm {nm}}(x,y,z)= & {} \left( 2^{n+m-1}n!m!\pi
								\right) ^{-1/2} \frac{1}{w(z)}~\exp {\left( \mathrm
								{i}\,(n+m+1)\varPsi (z)\right) }\nonumber \\&\times
								\,H_n\left( \frac{\sqrt{2}x}{w(z)}\right) H_m\left(
								\frac{\sqrt{2}y}{w(z)}\right) \exp {\left( -\mathrm
								{i}\,\frac{k(x^2+y^2)}{2R_C(z)}-\frac{x^2+y^2}{w^2(z)}\right)
								}.\nonumber \\ \end{aligned}$$

The latter form has the advantage of clearly showing the extra phase shift along the *z*-axis of $$(n+m+1)\varPsi
								(z)$$, called the *Gouy phase*; see Sect. 9.10.

### The Gaussian beam parameter

For a more compact description of the interaction of Gaussian modes with optical components we will make use of the *Gaussian beam parameter* *q* (Kogelnik 1965). The beam parameter is a complex quantity defined as

9.27 $$\begin{aligned}
								\frac{1}{q(z)}=\frac{1}{R_C(z)}-\mathrm {i}\,\frac{\lambda }{\pi
								w^2(z)}. \end{aligned}$$

It can also be written as

9.28 $$\begin{aligned}
								q(z)=\mathrm {i}\,z_{\mathrm {R}}+z-z_0=q_0+z-z_0\quad \mathrm
								{and}\quad q_0=\mathrm {i}\,z_{\mathrm {R}}.
								\end{aligned}$$

Using this parameter, Eq. (9.13) can be rewritten as

9.29 $$\begin{aligned}
								u(x,y,z)=\sqrt{\frac{2}{\pi }}\frac{q_0}{w_0q(z)}\exp {\left(
								-\mathrm {i}\,k\frac{x^2+y^2}{2q(z)}\right) }.
								\end{aligned}$$

Other parameters, like the beam size and radius of curvature, can also be written in terms of the beam parameter *q*:

9.30 $$\begin{aligned}
								w^2(z)= & {} \frac{\lambda }{\pi }\frac{|q|^2}{{\mathfrak
								{I}}\left\{ {q}\right\} }, \end{aligned}$$

9.31 $$\begin{aligned}
								w_0^2= & {} \frac{{\mathfrak {I}}\left\{ {q}\right\} \lambda
								}{\pi }, \end{aligned}$$

9.32 $$\begin{aligned}
								z_{\mathrm {R}}= & {} {\mathfrak {I}}\left\{ {q}\right\}
								\end{aligned}$$

and

9.33 $$\begin{aligned}
								R_C(z)=\frac{|q|^2}{{\mathfrak {R}}\left\{ q \right\} }.
								\end{aligned}$$

The Hermite–Gauss modes can also be written using the Gaussian beam parameter as^14^

9.34 $$\begin{aligned}
								u_{\mathrm {nm}}(x,y,z)= & {} u_{\mathrm {n}}(x,z)u_{\mathrm
								{m}}(y,z)\quad \text {with}\nonumber \\ u_{\mathrm {n}}(x,z)=
								& {} \left( \frac{2}{\pi }\right) ^{1/4}\left(
								\frac{1}{2^nn!w_0}\right) ^{1/2} \left( \frac{q_0}{q(z)}\right)
								^{1/2}\left( \frac{q_0~q^*(z)}{q_0^*~q(z)}\right) ^{n/2}\nonumber
								\\&\times H_n\left( \frac{\sqrt{2}x}{w(z)}\right) \exp
								{\left( -\mathrm {i}\,\frac{kx^2}{2q(z)}\right) }.
								\end{aligned}$$

### Properties of higher-order Hermite–Gauss modes

**Fig. 81:**
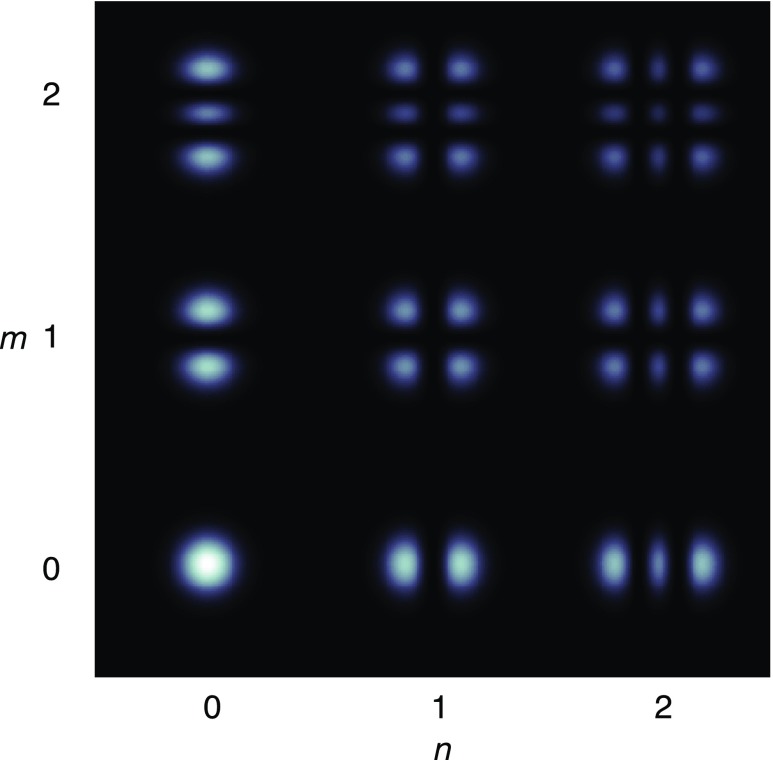
This *plot* shows the intensity distribution of Hermite–Gauss modes $u_{nm}$. One can see that the intensity distribution becomes wider for larger mode indices and the peak intensity decreases. The mode index defines the number of dark stripes in the respective direction

Some of the properties of Hermite–Gauss modes can easily be described using cross sections of the field intensity or field amplitude. Figure 81 shows such cross sections, i.e., the intensity in the *x*–*y* plane, for a number of higher-order modes. This shows a *x*–*y* symmetry for mode indices *n* and *m*. We can also see how the size of the intensity distribution increases with the mode index, while the peak intensity decreases. Similarly, Fig. 83 shows the amplitude and phase distribution of several higher-order Hermite–Gauss modes. Some further features of Hermite–Gauss modes:

- The size of the intensity profile of any sum of Hermite–Gauss modes depends on *z* while its shape remains constant over propagation along the optical axis.
- The phase distribution of Hermite–Gauss modes shows the curvature (or radius of curvature) of the beam. The curvature depends on *z* but is equal for all higher-order modes.

**Fig. 82:**
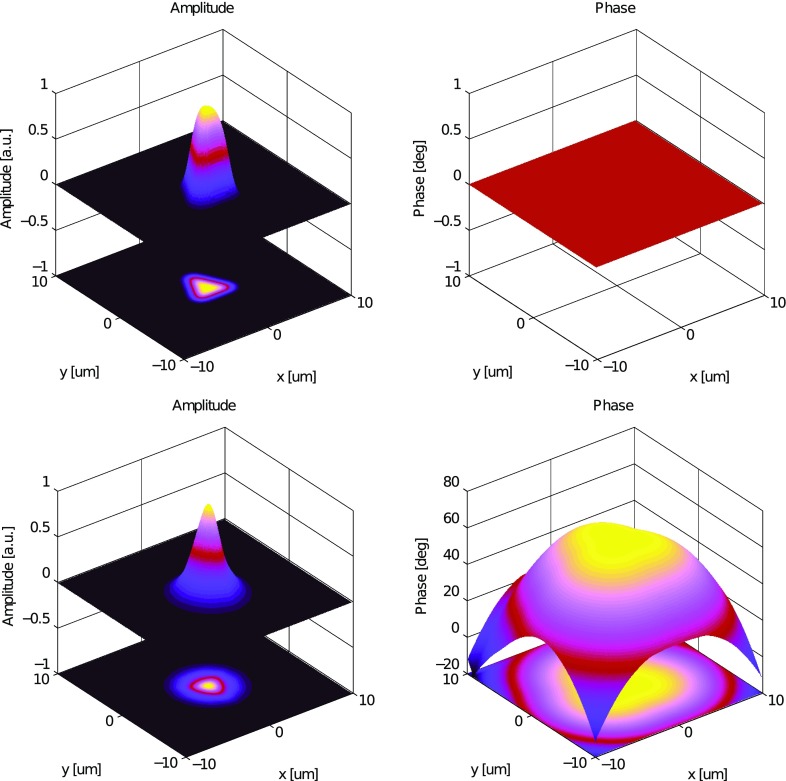
These *top plots* show a triangular beam shape and phase distribution and the *bottom plots* the diffraction pattern of this beam after a propagation of *z*  = 5 m. It can be seen that the shape of the triangular beam is not conserved and that the phase front is not spherical

Note that these are special features of Gaussian beams and not generally true for arbitrary beam shapes. Figure 82, for example, shows the amplitude and phase distribution of a triangular beam at the point where it is (mathematically) created and after a 10 m propagation. Neither the shape is preserved nor does it show a spherical phase distribution.

### Gouy phase

The equation for Hermite–Gauss modes shows an extra longitudinal phase lag. This *Gouy phase* (Boyd 1980; Gouy 1890a, b) describes the fact that, compared to a plane wave, the Hermite–Gauss modes have a slightly slower phase velocity, especially close to the waist. The Gouy phase can be written as

9.35 $$\begin{aligned}
								\varPsi (z)=\arctan \left( \frac{z-z_0}{z_{\mathrm {R}}}\right) ,
								\end{aligned}$$

or, using the Gaussian beam parameter,

9.36 $$\begin{aligned}
								\varPsi (z)=\arctan \left( \frac{{\mathfrak {R}}\left\{ q \right\}
								}{{\mathfrak {I}}\left\{ {q}\right\} }\right) .
								\end{aligned}$$

Compared to a plane wave, the phase lag $$\varphi
								$$ of a Hermite–Gauss mode is

9.37 $$\begin{aligned}
								\varphi =(n+m+1)\varPsi (z).
								\end{aligned}$$

With an astigmatic beam, i.e., different beam parameters in the tangential and sagittal planes, this becomes

9.38 $$\begin{aligned}
								\varphi =\left( n+\frac{1}{2}\right) \varPsi _t(z)+\left(
								m+\frac{1}{2}\right) \varPsi _s(z),
								\end{aligned}$$

with

9.39 $$\begin{aligned}
								\varPsi _t(z)=\arctan \left( \frac{{\mathfrak {R}}\left\{ q_t
								\right\} }{{\mathfrak {I}}\left\{ {q_t}\right\} }\right) ,
								\end{aligned}$$

as the Gouy phase in the tangential plane (and $$\varPsi
								_s$$ is similarly defined in the sagittal plane).

**Fig. 83:**
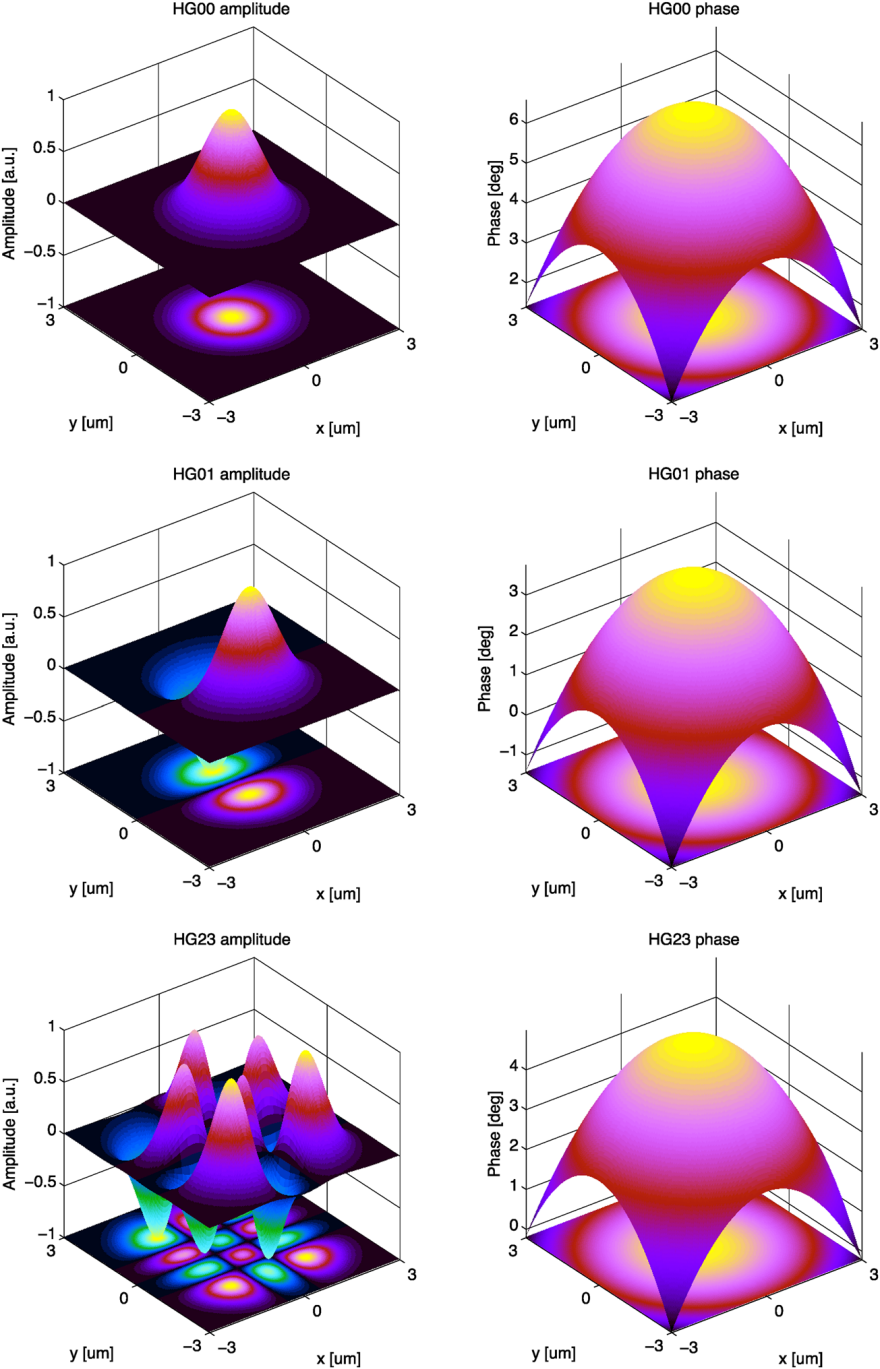
These *plots* show the amplitude distribution and wave front (phase distribution) of Hermite–Gaussian modes $$u_{nm}$$ (labeled as HG*nm* in the plot). All plots refer to a beam with $$\lambda = 1 \upmu \hbox
											{m}$$, *w* = 1 mm and distance to waist *z*  = 1 m. The mode index (in one direction) defines the number of zero crossings (along that axis) in the amplitude distribution. One can also see that the phase distribution is the same spherical distribution, regardless of the mode indices

### Laguerre–Gauss modes

Laguerre–Gauss modes are another complete set of functions, which solve the paraxial wave equation. They are defined in cylindrical coordinates and can have advantages over Hermite–Gauss modes in the presence of cylindrical symmetry. More recently, Laguerre–Gauss modes are being investigated in a different context: using a pure higher-order Laguerre–Gauss mode instead of the fundamental Gaussian beam can significantly reduce the impact of mirror thermal noise on the sensitivity of gravitational-wave detectors (Vinet 2009; Chelkowski et al. 2009). Laguerre–Gauss modes are commonly given as (Siegman 1986)

9.40 $$\begin{aligned}
								u_{p,l}(r,\phi ,z)= & {} \frac{1}{w(z)}\sqrt{\frac{2p!}{\pi
								(|l|+p)!}}\exp (\mathrm {i}\,(2p+|l|+1)\varPsi (z))\nonumber
								\\&\times \left( \frac{\sqrt{2}r}{w(z)}\right)
								^{|l|}L_p^{|l|}\left( \frac{2r^2}{w(z)^2}\right) \exp \left(
								-\mathrm {i}\,k\frac{r^2}{2q(z)}+\mathrm {i}\,l \phi \right) ,
								\end{aligned}$$

with $$r, \phi
								$$ and *z* as the cylindrical coordinates around the optical axis. The letter *p* is the radial mode index, *l* the azimuthal mode index^15^ and $$L_p^{|l|}(x)$$ are the associated Laguerre polynomials:

9.41 $$\begin{aligned}
								L_p^{|l|}(x)=\frac{1}{p!}\sum _{j=0}^p\frac{p!}{j!} \left(
								\begin{array}{c} |l|+p\\ p-j \end{array}\right) (-x)^j .
								\end{aligned}$$

All other parameters $$(w(z), q(z),
								\ldots )$$ are defined as above for the Hermite–Gauss modes.

The dependence of the Laguerre modes on $$\phi
								$$ as given in Eq. (9.40) results in a spiralling phase front, while the intensity pattern will always show unbroken concentric rings; see Fig. 84. These modes are also called *helical* Laguerre–Gauss modes because of the their special phase structure.

**Fig. 84:**
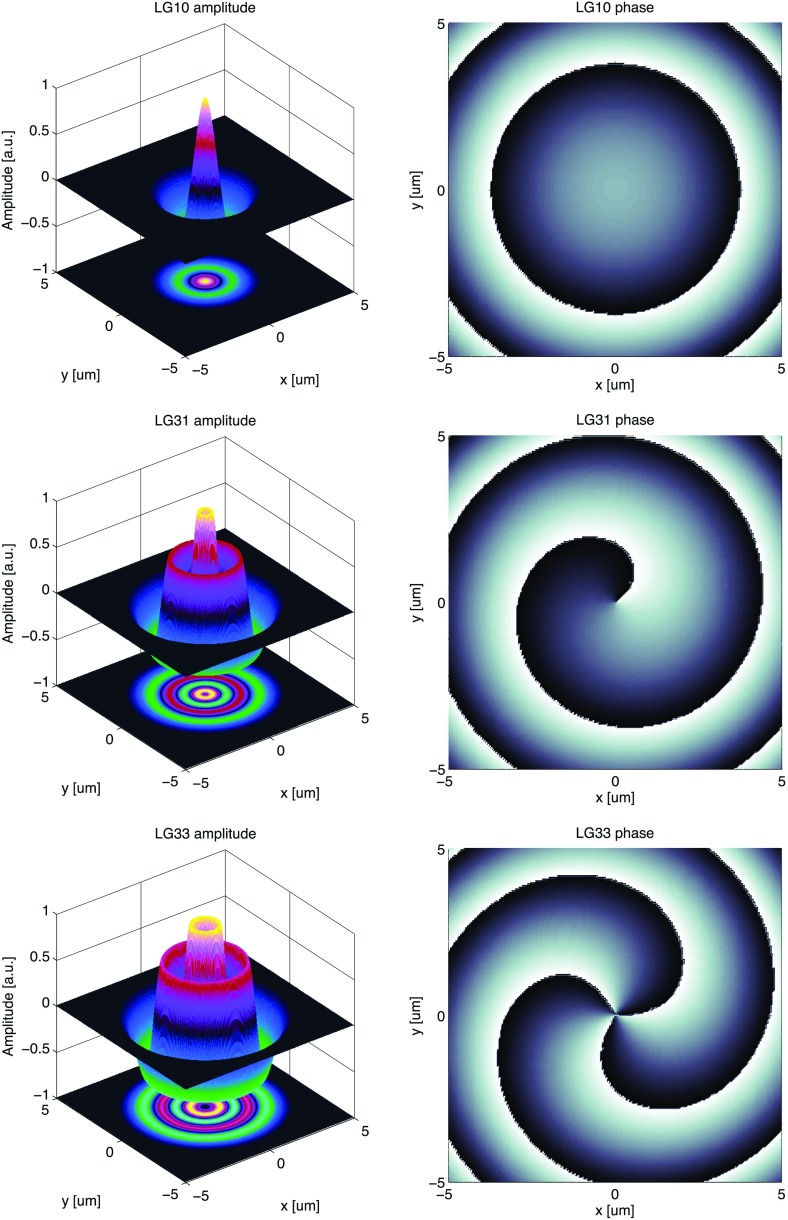
These plots show the amplitude distribution and wave front (phase distribution) of helical Laguerre–Gauss modes $$u_{pl}$$. All plots refer to a beam with $$\lambda =1\upmu \hbox
											{m}$$, *w* = 1 mm and distance to waist *z*  = 1 m

**Fig. 85:**
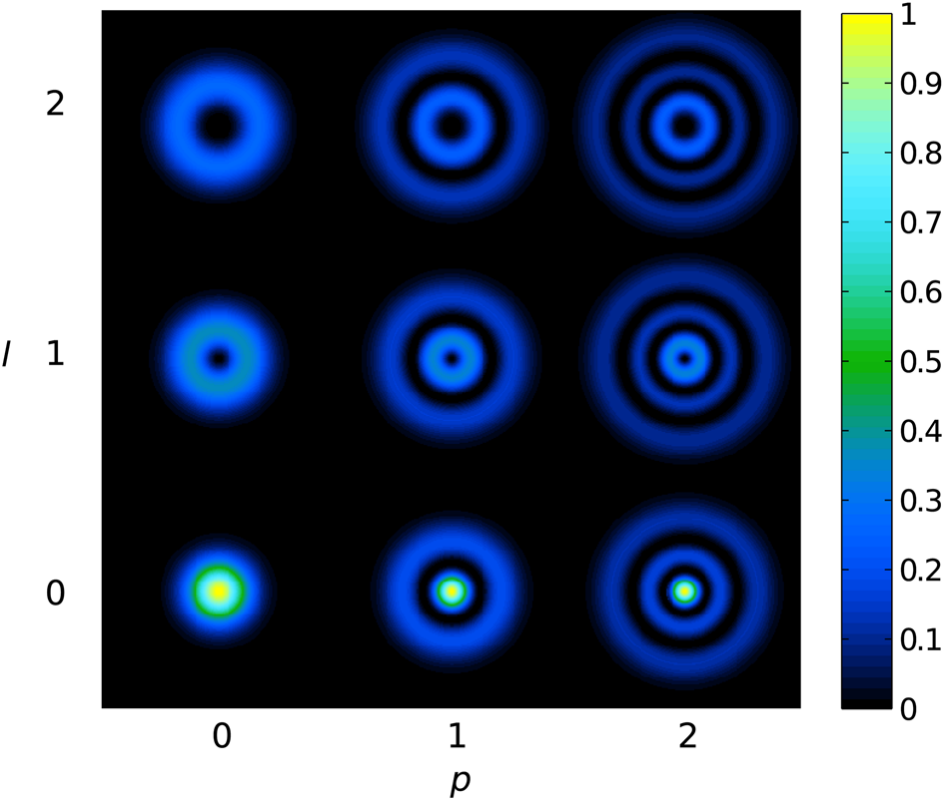
Intensity profiles for helical Laguerre–Gauss modes $u_{pl}$. The $u_{00}$ mode is identical to the Hermite–Gauss mode of order 0. Higher-order modes show a widening of the intensity and decreasing peak intensity. The number of concentric dark rings is given by the radial mode index *p*

**Fig. 86:**
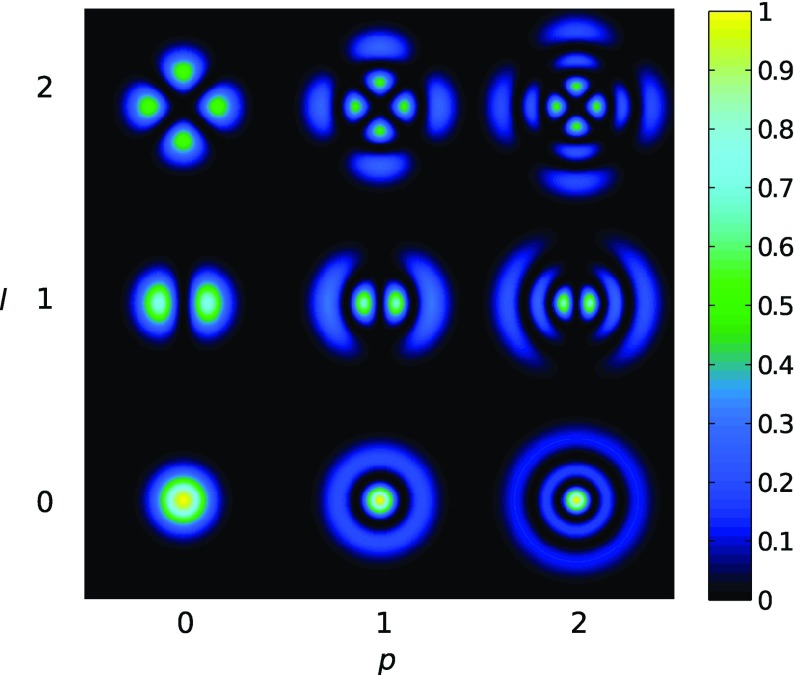
Intensity profiles for sinusoidal Laguerre–Gauss modes $$u^{\mathrm
											{alt}}_{pl}$$. The $$u_{p0}$$ modes are identical to the helical modes. However, for azimuthal mode indices $$l>0$$ the pattern shows *l* dark radial lines in addition to the *p* dark concentric rings

The reader might be more familiar with a slightly different type of Laguerre modes (compare Figs. 85 and 86) that features dark radial lines as well as dark concentric rings. Mathematically, these can be described simply by replacing the phase factor $$\exp (\mathrm
								{i}\,l \phi )$$ in Eq. (9.40) by a sine or cosine function. For example, an alternative set of Laguerre–Gauss modes is given by Vinet and the Virgo Collaboration (2001)

9.42 $$\begin{aligned}
								u^{\mathrm {alt}}_{p,l}(r,\phi ,z)= & {}
								\frac{2}{w(z)}\sqrt{\frac{p!}{(1+\delta _{0l}\pi (|l|+p)!}}\exp
								(\mathrm {i}\,(2p+|l|+1)\varPsi (z))\nonumber \\&\times
								\,\left( \frac{\sqrt{2}r}{w(z)}\right) ^{|l|}L_p^{|l|}\left(
								\frac{2r^2}{w(z)^2}\right) \exp \left( -\mathrm
								{i}\,k\frac{r^2}{2q(z)}\right) \cos (l \phi ).
								\end{aligned}$$

This type of mode has a spherical phase front, just as the Hermite–Gauss modes. We will refer to this set as *sinusoidal* Laguerre–Gauss modes throughout this document.

For the purposes of simulation it can be sometimes useful to decompose Laguerre–Gauss modes into Hermite–Gauss modes. The mathematical conversion for helical modes is given as (Beijersbergen et al. 1993; Abramochkin and Volostnikov 1991)

9.43 $$\begin{aligned}
								u^{LG}_{p,l}(x,y,z)=\sum _{k=0}^N (-1)^p (\mp \mathrm {i}\,)^k
								b(|l|+p,p,k) u^{HG}_{N-k,k}(x,y,z),
								\end{aligned}$$

where $${\mp
								}$$ is negative for positive *l* and positive for negative *l* and with real coefficients

9.44 $$\begin{aligned}
								b(n,m,k)=\sqrt{\frac{(N-k)!k!}{2^N n!m!}} \frac{1}{k!}(\partial
								_t)^k[(1-t)^n(1+t)^m]_{t=0},
								\end{aligned}$$

if $N=2p+|l|$. The coefficients *b*(*n*, *m*, *k*) can be computed numerically by using Jacobi polynomials. Jacobi polynomials can be written in various forms:

9.45 $$\begin{aligned}
								P_n^{\alpha , \beta }(x)=\frac{(-1)^n}{2^n n!}(1-x)^{-\alpha
								}(1+x)^{-\beta }(\partial _x)^n(1-x)^{\alpha +n}(1+x)^{\beta +n},
								\end{aligned}$$

or

9.46 $$\begin{aligned}
								P_n^{\alpha , \beta }(x)=\frac{1}{2^n}\sum _{j=0}^n \left(
								\begin{array}{c} n+\alpha \\ j \end{array}\right) \left(
								\begin{array}{c} n+\beta \\ n-j \end{array}\right)
								(x-1)^{n-j}(x+1)^j , \end{aligned}$$

which leads to

9.47 $$\begin{aligned}
								b(n,m,k)=\sqrt{\frac{(N-k)!k!}{2^N n!m!}}~(-2)^k P_k^{n-k,m-k}(0).
								\end{aligned}$$

### Tracing a Gaussian beam through an optical system

Whenever Gauss modes are used to analyse an optical system, the Gaussian beam parameters (or equivalent waist sizes and locations) must be defined for each location at which field amplitudes are to be computed (or at which coupling equations are to be defined). In our experience the quality of a computation or simulation and the correctness of the results depend critically on the choice of these beam parameters. One might argue that the choice of a basis should not alter the result. This is correct, but there is a practical limitation: the number of modes having non-negligible power might become very large if the beam parameters are not optimised, so that in practice a good set of beam parameters is usually required.

In general, the Gaussian beam parameter of a mode is changed at every optical surface in a well-defined way (see Sect. 9.13). Thus, a possible method of finding reasonable beam parameters for every location in the interferometer is to first set only some specific beam parameters at selected locations and then to derive the remaining beam parameters from these initial ones: usually it is sensible to assume that the beam at the laser source can be properly described by the (hopefully known) beam parameter of the laser’s output mode. In addition, in most stable cavities the light fields should be described by using the respective cavity eigenmodes. Then, the remaining beam parameters can be computed by *tracing* the beam through the optical system. ‘Trace’ in this context means that a beam starting at a location with an already-known beam parameter is propagated mathematically through the optical system. At every optical element along the path the beam parameter is transformed according to the ABCD matrix of the element (see below).

### ABCD matrices

The transformation of the beam parameter can be performed by the ABCD matrix-formalism (Kogelnik 1965; Siegman 1986). When a beam passes an optical element or freely propagates though space, the initial beam parameter $q_1$ is transformed into $q_2$. This transformation can be described by four real coefficients as follows:

9.48 $$\begin{aligned}
								\frac{q_2}{n_2}=\frac{A\frac{q_1}{n_1}+B}{C\frac{q_1}{n_1}+D},
								\end{aligned}$$

with the coefficient matrix

9.49 $$\begin{aligned}
								M=\left( \begin{array}{c@{\quad }c} A&{} B \\ C&{} D
								\end{array} \right) , \end{aligned}$$

$n_1$ being the index of refraction at the beam segment defined by $q_1$, and $n_2$ the index of refraction at the beam segment described by $q_2$. ABCD matrices for some common optical components are given below, for the sagittal and tangential plane.

**Fig. 87:**
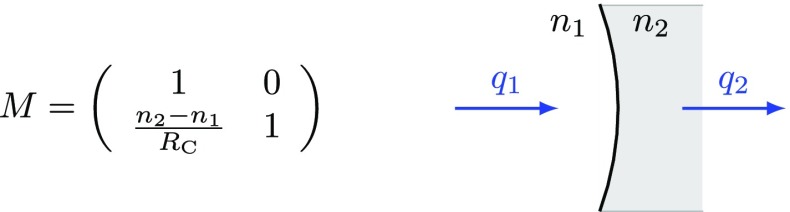
ABCD matrix for the transmission through a surface at normal incidence

*Transmission through a mirror*

A mirror in this context is a single, partly-reflecting surface with an angle of incidence of 90°. The transmission is described by (Fig. 87)

with $$R_{\mathrm
								{C}}$$ being the radius of curvature of the spherical surface. The sign of the radius is defined such that $$R_{\mathrm
								{C}}$$ is negative if the centre of the sphere is located in the direction of propagation. The curvature shown above (in Fig. 87), for example, is described by a positive radius. The matrix for the transmission in the opposite direction of propagation is identical.

*Reflection at a mirror*

**Fig. 88:**
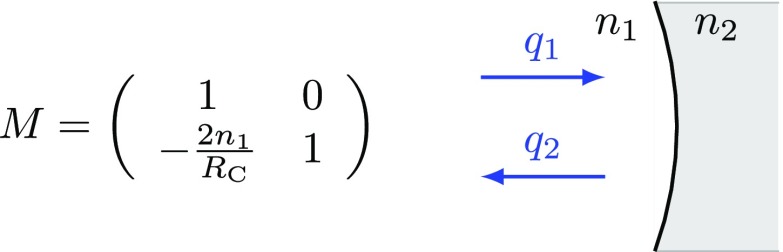
ABCD matrix for the reflection at a surface at normal incidence

The matrix for reflection is given by (Fig. 88)

The reflection at the back surface can be described by the same type of matrix by setting $C=2n_2/R_{\mathrm {C}}$.

*Transmission through a beam splitter*

A beam splitter is understood as a single surface with an arbitrary angle of incidence $$\alpha
								_1$$. The matrices for transmission and reflection are different for the sagittal and tangential planes ($$M_{\mathrm
								{s}}$$ and $$M_{\mathrm
								{t}}$$) (Fig. 89):

**Fig. 89:**
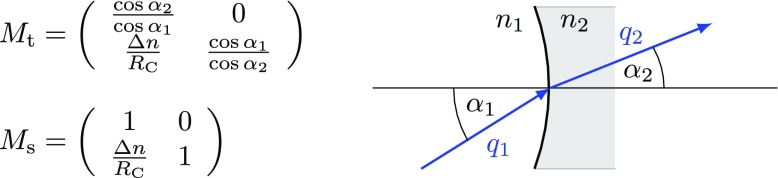
ABCD matrix for the transmission through a surface with an arbitrary angle of incidence

with $$\alpha
								_2$$ given by Snell’s law:

9.50 $$\begin{aligned}
								n_1\sin {\left( \alpha _1\right) }=n_2\sin {\left( \alpha _2\right)
								}, \end{aligned}$$

and $$\varDelta
								n$$ by

9.51 $$\begin{aligned}
								\varDelta n=\frac{n_2\cos {\left( \alpha _2\right) }-n_1\cos {\left(
								\alpha _1\right) }}{\cos {\left( \alpha _1\right) }\cos {\left(
								\alpha _2\right) }}. \end{aligned}$$

If the direction of propagation is reversed, the matrix for the sagittal plane is identical and the matrix for the tangential plane can be obtained by changing the coefficients A and D as follows:

9.52 $$\begin{aligned}&A\longrightarrow
								1/A,\nonumber \\&D\longrightarrow 1/D.
								\end{aligned}$$

*Reflection at a beam splitter*

**Fig. 90:**
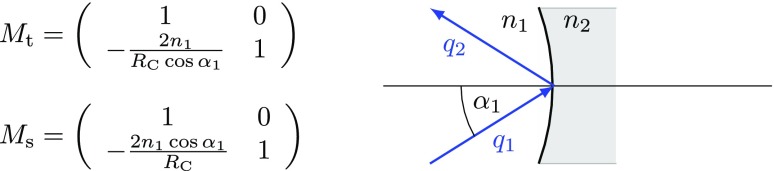
ABCD matrix for the reflection at a surface with an arbitrary angle of incidence

The reflection at the front surface of a beam splitter is given by (Fig. 90):

To describe a reflection at the back surface the matrices have to be changed as follows:

9.53 $$\begin{aligned}&R_{\mathrm
								{C}}\longrightarrow -R_{\mathrm {C}},\nonumber
								\\&n_1\longrightarrow n_2,\nonumber \\&\alpha
								_1\longrightarrow -\alpha _2.
								\end{aligned}$$

*Transmission through a thin lens*

**Fig. 91:**
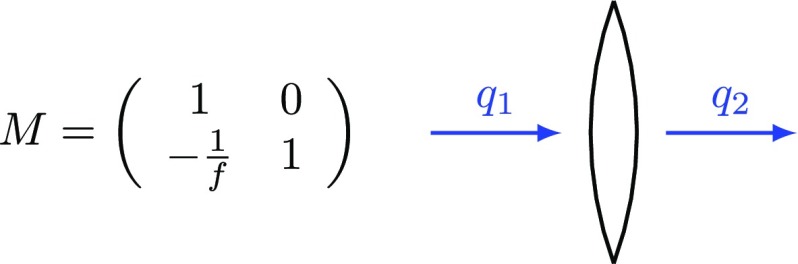
ABCD matrix for a thin lens

A thin lens transforms the beam parameter as follows (Fig. 91):

where *f* is the focal length. The matrix for the opposite direction of propagation is identical. Here it is assumed that the thin lens is surrounded by ‘spaces’ with index of refraction $n=1$.

*Transmission through a free space*

As mentioned above, the beam in free space can be described by one base parameter $$q_0$$. In some cases it is convenient to use a matrix similar to that used for the other components to describe the *z*-dependency of $$q(z)=
								q_0+z$$. On propagation through a free space of the length *L* and index of refraction *n*, the beam parameter is transformed as follows (Fig. 92).

**Fig. 92:**
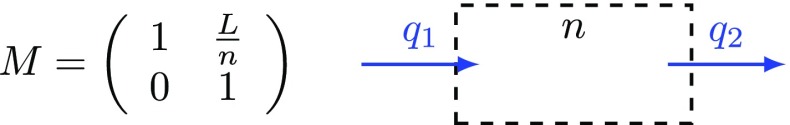
ABCD matrix for free propagation (a ‘space’ component)

The matrix for the opposite direction of propagation is identical.

### Computing a cavity eigenmode and stability

A cavity eigenmode is defined as the optical field whose spatial properties are such that the field after one round-trip through the cavity will be exactly the same as the injected field. In the case of resonators with spherical mirrors, the eigenmode will be a Gaussian mode, defined by the Gaussian beam parameter $q_\mathrm{{cav}}$. For a generic cavity (an arbitrary number of spherical mirrors or lenses) a round-trip ABCD matrix $M_\mathrm{{rt}}$ can be defined and used to compute the cavity’s eigenmode. Chapter 21 of Siegman (1986) provides a comprehensive description of different optical resonators including a derivation and discussion of stability criteria. Here we provide a brief introduction focussing on the specific case of closed and stable resonators with spherical mirrors.

The change in the *q* parameter after one round-trip through a cavity is given by:

9.54 $$\begin{aligned}
								\frac{A q_{1} + B}{C q_{1} + D} = q_{2} = q_{1}
								\end{aligned}$$

where *A*, *B*, *C* and *D* are the elements of a matrix $$M_\mathrm{{rt}}$$. If $$q_1 =
								q_2$$ then the spatial profile of the beam is recreated after each round-trip and we have identified the cavity eigenmode. We can compute the parameter $$q_\mathrm{{cav}}
								\equiv q_1 = q_2$$ by solving:

9.55 $$\begin{aligned}
								C q_\mathrm{{cav}}^2+(D-A)q_\mathrm{{cav}} - B = 0,
								\end{aligned}$$

**Fig. 93:**
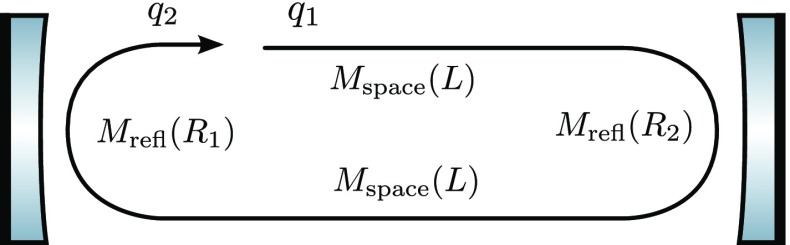
Cavity round trip ABCD matrices for a 2-mirror cavity

For example, in the case of the two-mirror cavity shown in Fig. 93 the matrix is given by:

9.56 $$\begin{aligned}
								M_\mathrm{{rt}}= & {} M_{space}(L) \times
								M_\mathrm{{refl}}(R_2) \times M_\mathrm{{space}}(L) \times
								M_\mathrm{{refl}}(R_1), \end{aligned}$$

with *L* the length of the cavity, and $R_{1/2}$ the radii of curvature of the mirrors. Now we can compute the A, B, C and D coefficients for the round-trip matrix $M_\mathrm{{rt}}$ to solve Eq. (9.55). This quadratic equation generally has two solutions, one being the complex conjugate of the other.

When the polynomial above has a suitable solution the optical resonator is said to be ‘stable’. The stability requirement can be formulated using the Gaussian beam parameter: a cavity is stable only when the cavity’s eigenmode, $$q_\mathrm{{cav}}$$, has a real waist size. The value for the beam waist is a real number whenever $$q_\mathrm{{cav}}$$ has a positive non-zero imaginary part, as this defines the Rayleigh range of the beam and therefore the beam waist, $${\text
								{Im}}(q_\mathrm{{cav}}) = \pi w_0^2/\lambda
								$$. A complex $$q_\mathrm{{cav}}$$ is ensured if the determinant of Eq. (9.55) is negative. This requirement can be formulated in a compact way by defining the parameter *m* as:

9.57 $$\begin{aligned}
								m \equiv \frac{A+D}{2}, \end{aligned}$$

where *A* and *B* are the coefficients of the round-trip matrix $M_\mathrm{rt}$. The stability criterion then simply becomes:

9.58 $$\begin{aligned}
								m^2 < 1. \end{aligned}$$

The stability of simple cavities are often described using *g-factors*. These factors are simply rescaled versions of the more generic *m* value:

9.59 $$\begin{aligned}
								g \equiv \dfrac{m+1}{2} = \dfrac{A+D+2}{4},
								\end{aligned}$$

For the cavity to be stable the g-factor must fulfil the requirement:

9.60 $$\begin{aligned}
								0 \le g \le 1 \end{aligned}$$

The closer *g* is to the 0 or 1, the smaller the tolerances are for any change in the geometry before the cavity becomes unstable.

For a simple two-mirror cavity of length *L* and mirror radii of curvature $R_{1,2}$, its *g-factor* is

9.61 $$\begin{aligned}
								g_1= & {} 1 - \frac{L}{R_1},
								\end{aligned}$$

9.62 $$\begin{aligned}
								g_2= & {} 1 - \frac{L}{R_2},
								\end{aligned}$$

9.63 $$\begin{aligned}
								g= & {} g_1 g_2. \end{aligned}$$

where $g_{1,2}$ are the individual *g-factors* of the cavity mirrors and *g* is the g-factor of the entire cavity.

### Round-trip Gouy phase and higher-order-mode separation

As discussed in Sect. 9.10, as a higher order optical mode propagates it accumulates an additional phase, the Gouy phase, proportional to its mode order. To determine how such a mode resonantes within an optical cavity the accumulated Gouy phase on one round-trip through the cavity must be included. The round-trip Gouy phase will determine which order of optical modes are resonant within a cavity. As the resonance condition of a mode is dependent on its order, this allows an optical setup to select particular orders of optical modes from an incident field. This behaviour is the basis of *mode-cleaner* cavities; such as those used for the input and output light of gravitational-wave detectors.

To compute the round-trip Gouy phase the evolution of the beam shape through the cavity must first be computed. This involves computing the round-trip ABCD matrix, $M_\mathrm{{RT}}$, as outlined in Sect. 9.14. With this matrix the round-trip Gouy phase is computed using its elements (Arai 2013):

9.64 $$\begin{aligned}
								\psi _\mathrm{{RT}}= & {} 2 \mathrm {arccos} \left( \mathrm
								{sign}(B) \sqrt{ \dfrac{A+D+2}{4} } \right) ,
								\end{aligned}$$

9.65 $$\begin{aligned}
								\psi _\mathrm{{RT}}(g)= & {} 2 \mathrm {arccos} \left(
								\mathrm {sign}(B) \sqrt{g} \right) .
								\end{aligned}$$

As can be seen, the round-trip Gouy phase is linked to the cavity’s g-factor, (9.59). As the cavity approaches instability, $$g\rightarrow
								0$$ or 1, the phase accumulated by a mode TEM$$_\mathrm{nm}$$ is then $$(n+m)\psi
								_\mathrm{{RT}}(0) = (n+m)\pi /2$$ or $$(n+m)\psi
								_\mathrm{{RT}}(1) = 0$$. In the later case all higher order optical modes—regardless of their mode order—are resonant in the cavity at the same time. In the former case either the odd or even mode orders are resonant at once.

The effect of the round-trip Gouy phase is often referred to as the *higher order mode separation frequency*. This states how far the resonance of the next optical order is in frequency:

9.66 $$\begin{aligned}
								\delta f = \dfrac{\psi _{\mathrm {RT}}}{2\pi } \mathrm {FSR}.
								\end{aligned}$$

For example, the Advanced LIGO arm cavities have $$\delta f \approx
								5$$ kHz.

### Coupling of higher-order-modes

Now that we are able to compute the eigenmode of a particular cavity, what happens if a beam with a slightly different eigenmode is injected into it? The aim of this section is to outline the problem. In reality producing a perfect Gaussian laser which matches exactly the eigenmode of a cavity is essentially impossible, there will always be a minor difference. However we are still able to inject lasers into a cavity and produce a resonance. This is because as long as the eigenmode of the incoming laser is nearly the same, the majority of the laser light will ‘fit’ into the cavity and resonate, the rest will be reflected from it.

Let us consider a cavity with eigenmode $q_2$ and an incident Gaussian beam with eigenmode $q_1$. The incident beam has all of its power in the fundamental 00 mode. For a cavity with perfectly spherical mirrors there are two possible ‘misconfigurations’ that can take place:

- If the optical axes of the beam and the cavity do not overlap perfectly, the setup is called *misaligned*,
- If the beam size or shape at cavity input does not match the beam shape and size of the (resonant) fundamental eigenmode ($$q_1(z_{\mathrm {cav}})\ne
											q_2(z_{\mathrm {cav}})$$), the beam is then not *mode-matched* to the second cavity, i.e., there is a *mode mismatch*.

The coupling of a mode refers to how a spatial mode in one basis is represented in another; e.g., which sum of modes in the cavity basis $q_2$ produces the HG$_{00}$ mode in the $q_1$ basis. Hermite–Gauss modes are coupled whenever a beam is not matched or aligned to a cavity or beam segment. This coupling is sometimes referred to as *scattering* into higher-order modes because in most cases the laser beam is a considered as a pure HG$_{00}$ mode and any mode coupling would transfer power from the fundamental into higher-order modes. However, in general every mode with non-zero power will transfer energy into other modes whenever mismatch or misalignment occur, and this effect also includes the transfer from higher orders into a low order.

To compute the amount of coupling the beam must be projected into the base system of the cavity or beam segment it is being injected into. This is always possible, provided that the paraxial approximation holds, because each set of Hermite–Gauss modes, defined by the beam parameter at a position *z*, forms a complete set. Such a change of the basis system results in a different distribution of light power in the new Hermite–Gauss modes and can be expressed by coupling coefficients that yield the change in the light amplitude and phase with respect to mode number.

Let us assume that a beam described by the beam parameter $$q_1$$ is injected into a segment described by the parameter $$q_2$$. Let the optical axis of the beam be misaligned: the coordinate system of the beam is given by (*x*, *y*, *z*) and the beam travels along the *z*-axis. The beam segment is parallel to the $$z'$$-axis and the coordinate system ($$x', y',
								z'$$) is given by rotating the (*x*, *y*, *z*) system around the *y*-axis by the *misalignment angle* $$\gamma
								$$. The amplitude of a particular mode TEM$$_\mathrm{nm}$$ in the beam segment is then defined as:

9.67 $$\begin{aligned}
								u_{n m}(x,y;\,q_2)\exp {\Bigl (\mathrm {i}\,(\omega t -k z)\Bigr
								)}=\sum _{n',m'}k_{n,m,n',m'}u_{n' m'}(x,y;\,q_1)\exp {\Bigl
								(\mathrm {i}\,(\omega t -k z')\Bigr )},
								\end{aligned}$$

where $$u_{n'
								m'}(x,y;\,q_1)$$ are the Hermite–Gauss modes used to describe the injected beam, $$u_{n
								m}(x,y;\,q_2)$$ are the ‘new’ modes that are used to describe the light in the beam segment and $$k_{n,m,n',m'}$$ is the *coupling coefficient* from each TEM$$_\mathrm{n'm'}$$ into TEM$$_\mathrm{nm}$$. Note that including the plane wave phase propagation within the definition of coupling coefficients is important because it results in coupling coefficients that are independent of the position on the optical axis for which the coupling coefficients are computed.

Using the fact that the Hermite–Gauss modes $$u_{n
								m}$$ are orthonormal, we can compute the coupling coefficients by the overlap integral (Bayer-Helms 1984):

9.68 $$\begin{aligned}
								k_{n,m,n',m'}=\exp {\left( \mathrm {i}\,2 k z' \sin ^2\left(
								\frac{\gamma }{2}\right) \right) }\int \!\!\!\int \!dx'\,dy'~ u_{n'
								m'}\exp {\left( \mathrm {i}\,k x' \sin {\gamma }\right) }~u^*_{n m}.
								\end{aligned}$$

Since the Hermite–Gauss modes can be separated with respect to *x* and *y*, the coupling coefficients can also be split into $$k_{n m n'
								m'}=k_{n n'}k_{m m'}$$. These equations are very useful in the paraxial approximation as the coupling coefficients decrease with large mode numbers. In order to be described as paraxial, the angle $$\gamma
								$$ must not be larger than the diffraction angle. In addition, to obtain correct results with a finite number of modes the beam parameters $$q_1$$ and $$q_2$$ must not differ too much.

The integral 9.68 can be computed directly using numerical integration methods. However, this can potentially be computationally very expensive depending on how difficult the integrand is to evaluate and complex it. The following part of this section is based on the work of Bayer-Helms (1984) and provides an analytic solution to the integral. Another description of coupling coefficients and their derivation can be found in the work of Vinet and the Virgo Collaboration (2001). In Bayer-Helms (1984) the above integral is partly solved and the coupling coefficients are given by multiple sums as functions of $$\gamma
								$$ and the mode mismatch parameter *K*, which is defined by

9.69 $$\begin{aligned}
								K=\frac{1}{2}(K_0+\mathrm {i}\,K_2),
								\end{aligned}$$

where $$K_0=(z_R-z_R')/z_R'$$ and $$K_2=((z-z_0)-(z'-z_0'))/z_R'$$. This can also be written using $$q=\mathrm
								{i}\,z_{\mathrm {R}}+z-z_0$$, as

9.70 $$\begin{aligned}
								K=\frac{\mathrm {i}\,(q-q')^*}{2 {\mathfrak {I}}\left\{ {q'}\right\}
								}. \end{aligned}$$

The coupling coefficients for misalignment and mismatch (but no lateral displacement) can then be written as

9.71 $$\begin{aligned}
								k_{n n'}=(-1)^{n'} E^{(x)} (n!n'!)^{1/2} (1+K_0)^{n/2+1/4}
								(1+K^*)^{-(n+n'+1)/2}\left\{ S_g-S_u\right\} ,\quad
								\end{aligned}$$

where

9.72 $$\begin{aligned}
								{\begin{array}{l} S_g=\sum \limits _{\mu =0}^{[n/2]}\sum \limits
								_{\mu '=0}^{[n'/2]} \frac{(-1)^\mu \bar{X}^{n-2\mu }X^{n'-2\mu
								'}}{(n-2\mu )!(n'-2\mu ')!} \sum \limits _{\sigma =0}^{\min (\mu
								,\mu ')}\frac{(-1)^\sigma \bar{F}^{\mu -\sigma } F^{\mu '-\sigma
								}}{(2\sigma )! (\mu -\sigma )! (\mu '-\sigma )!},\\ S_u=\sum \limits
								_{\mu =0}^{[(n-1)/2]}\sum \limits _{\mu '=0}^{[(n'-1)/2]}
								\frac{(-1)^\mu \bar{X}^{n-2\mu -1}X^{n'-2\mu '-1}}{(n-2\mu
								-1)!(n'-2\mu '-1)!} \sum \limits _{\sigma =0}^{\min (\mu ,\mu
								')}\frac{(-1)^\sigma \bar{F}^{\mu -\sigma } F^{\mu '-\sigma
								}}{(2\sigma +1)! (\mu -\sigma )! (\mu '-\sigma )!}. \end{array}}
								\end{aligned}$$

The corresponding formula for $$k_{m
								m'}$$ can be obtained by replacing the following parameters: $$n\rightarrow
								m$$, $$n'\rightarrow
								m'$$, $$X,\bar{X}\rightarrow 0$$ and $$E^{(x)}\rightarrow 1$$ (see below). The notation [*n* / 2] means

9.73 $$\begin{aligned}
								\left[ \frac{m}{2}\right] =\left\{ \begin{array}{l@{\quad }l} m/2
								&{} \text {if}\ m\ \text {is even,}\\ (m-1)/2 &{}
								\text {if}\ m\ \text {is odd.} \end{array}\right.
								\end{aligned}$$

The other abbreviations used in the above definition are

9.74 $$\begin{aligned}
								{ \begin{array}{l} \bar{X}={(\mathrm {i}\,z_{\mathrm {R}}'-z')\sin
								{(\gamma )}}/({\sqrt{1+K^*}w_0}),\\ X={(\mathrm {i}\,z_{\mathrm
								{R}}+z')\sin {(\gamma )}}/({\sqrt{1+K^*}w_0}),\\
								F={K}/({2(1+K_0)}),\\ \bar{F}={K^*}/{2},\\ E^{(x)}=\exp {\left(
								-\frac{X\bar{X}}{2}\right) }. \end{array}}
								\end{aligned}$$

In general, the Gaussian beam parameter might be different for the sagittal and tangential planes and a misalignment can be given for both possible axes (around the *y*-axis and around the *x*-axis), in this case the coupling coefficients are given by

9.75 $$\begin{aligned}
								k_{nm m'n'}=k_{n n'} k_{m m'},
								\end{aligned}$$

where $$k_{n
								n'}$$ is given above with

9.76 $$\begin{aligned}
								\begin{array}{l} q \rightarrow q_t\\ \text {and}\\ w_0\rightarrow
								w_{t,0} \text {, etc.} \end{array}
								\end{aligned}$$

and $$\gamma
								\rightarrow \gamma _y$$ is a rotation about the *y*-axis. The $$k_{m
								m'}$$ can be obtained with the same formula, with the following substitutions:

9.77 $$\begin{aligned}
								\begin{array}{l} n \rightarrow m,\\ n' \rightarrow m', \\ q
								\rightarrow q_s,\\ \text {thus}\\ w_0\rightarrow w_{s,0} \text {,
								etc.} \end{array} \end{aligned}$$

and $$\gamma
								\rightarrow \gamma _x$$ is a rotation about the *x*-axis. At each component a matrix of coupling coefficients has to be computed for every time a beam transfers from one eigenmode to another for transmission and reflection as depicted in Fig. 94.

In this section we have outlined how an incoming higher-order-mode will be coupled into an outgoing beam basis when taking into account a difference in the eigenmode of two sections of the interferometer or misalignments. This coupling of higher-order-modes is a very powerful tool that is used throughout this article, as it allows us to model interferometers with realistic defects; like imperfect mirror surfaces or misaligned optics. This enables us to better understand the reasons why complex interferometers behave in certain ways and provide solutions to combat particular problems that might arise. The next section details how misalignments and mode-mismatching affect the dual-recycled Fabry–Perot–Michelson interferometers that were described in Sect. 7. Then Sect. 11 lays out the theory behind a more general form of scattering HOMs undergo when they interact with surface defects present on mirrors or beamsplitters.

**Fig. 94:**
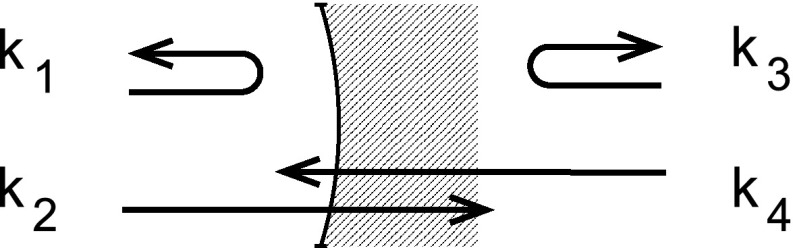
Coupling coefficients for Hermite–Gauss modes: for each optical element and each direction of propagation complex coefficients *k* for transmission and reflection have to be computed. In this figure $$k_1$$, $$k_2$$, $$k_3$$, $$k_4$$ each represent a matrix of coefficients $$k_{n m n'
											m'}$$ describing the coupling of $$u_{n,m}$$ into $$u_{n',m'}$$

### Finesse examples

#### Beam parameter tracing

**Fig. 95:**
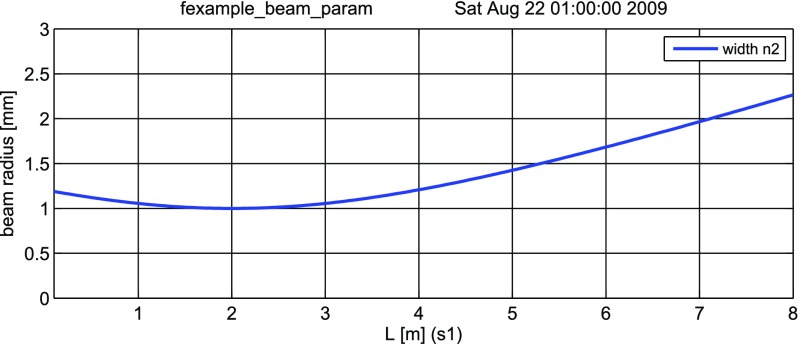
Finesse example: beam parameter tracing

This example illustrates a possible use of the beam parameter detector ‘bp’: the beam radius of the laser beam is plotted as a function of distance to the laser. For this simulation, the interferometer matrix does not need to be solved. ‘bp’ merely returns the results from the beam tracing algorithm of Finesse (Fig. 95).

**Finesse input file for ‘Beam parameter tracing’** 
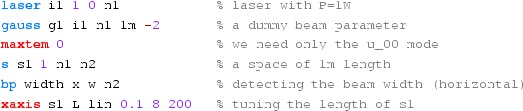

**Fig. 96:**
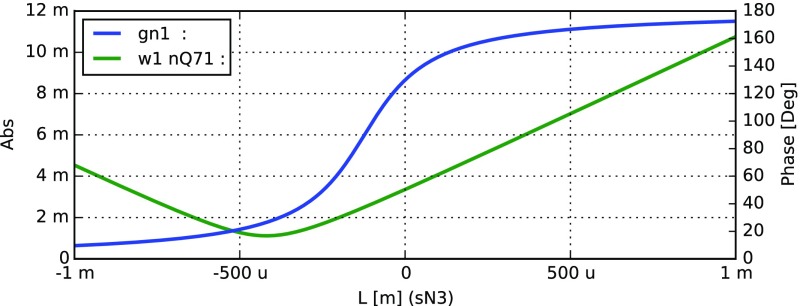
Finesse example: telescope and Gouy phase. The *blue trace* shows the Gouy phase accumulated in the telescope, the *green trace* the beam spot size at the end of the telescope. The change on the x-axis represents a position tuning of the lens ‘L2’

#### Telescope and Gouy phase

This example shows the fine tuning of a telescope. The optical setup is similar to the optical layout on the Virgo North-end detection bench, resembling the telescope for the beam transmitted by the end mirror of one arm. The telescope consists of four sequential lenses with the purpose of reducing the beam size and provide a user defined Gouy phase for a split photo detector which is used for the alignment sensing system (Fig. 96).

**Finesse input file for ‘Telescope and Gouy phase’** 
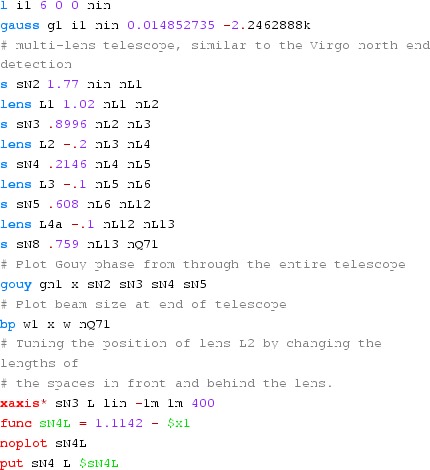


#### LG33 mode

**Fig. 97:**
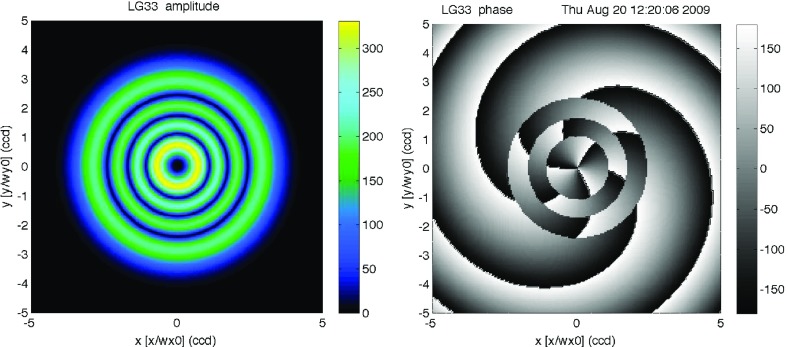
Finesse example: LG33 mode. The ring structure in the phase plot is due to phase jumps, which could be removed by applying a phase ‘unwrap’

Finesse uses the Hermite–Gauss modes as a base system for describing the spatial properties of laser beams. However, Laguerre–Gauss modes can be created using the coefficients given in Eq. (9.43). This example demonstrates this and the use of a ‘beam’ detector to plot amplitude and phase of a beam cross section (Fig. 97).

**Finesse input file for ‘LG33 mode’** 
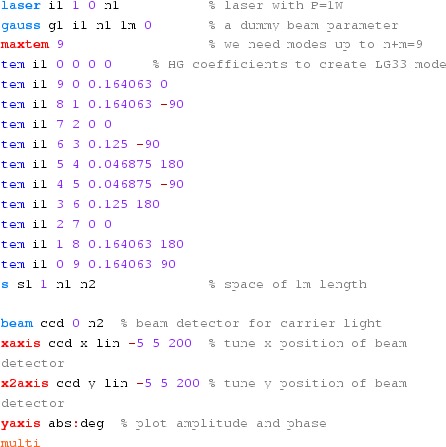


## Imperfect interferometers

Imperfections in a Michelson interferometer can refer to any of the differences between a real interferometer and the perfect design. These include, but are not limited to: deviations of the optical properties of the mirrors from the design; the limits of longitudinal and alignment control of the mirrors; additional noise sources not included in our models (i.e., electronic noise); and effects which distort the shape of the beam. To estimate the impact of such imperfections on the Michelson’s performance is complicated and requires substantial modelling. The greatest impact on the sensitivity arises from asymmetries between the two arms. For accurate differential measurements, such as those made in gravitational wave interferometers, the mirrors are very carefully manufactured to make the arms as similar as possible. Differences between the two arms, for example, imbalances in the finesse of the two arm cavities, will couple extra light into the anti-symmetric port of the interferometer where it adds additional noise to the detection photodiode.

It is important to understand how imperfections in an interferometer affect the resonating beams and impact the sensitivity of the instrument. For this we need accurate models which can simulate complex interferometers in the presence of such imperfections. This is crucial for the design of interferometers, such as gravitational-wave detectors, and the commissioning process, in which deviations of the interferometer behaviour from the expected design must be diagnosed. In this review we will consider imperfections in the form of distortions of the beam and we discuss these effects for gravitational wave interferometers; firstly in terms of the behaviour of distorted optics and how this effects the performance of different optical configurations; and secondly in terms of solutions to these distortion problems and implications for the design process.

### Spatial modes in optical cavities

In the previous chapter the idea of representing distortions of a beam as higher-order Gaussian modes was introduced. Here we use this description to investigate the behaviour of interferometers with distorted beams.

A well designed optical cavity can act as a resonator for a particular order of Gaussian modes, depending on its longitudinal tuning. In modern interferometers such cavities are operated as resonators for the fundamental mode, filtering out unwanted spatial components of the beam. This is achieved as each Gauss mode is subject to an additional phase term as it propagates. This additional phase depends on the mode order:

10.1 $$\begin{aligned}
								\varphi (z) = (n+m+1) \psi (z) = (n+m+1) \tan ^{-1}\left(
								\frac{z}{z_R}\right) \end{aligned}$$

where $$n+m$$ is the mode order, $$\psi
								$$ is the Gouy phase and $$z_R$$ is the Rayleigh range (see Sect. 9.5). This additional phase ensures different modes are resonant in a cavity at different longitudinal tunings. The Gouy phase accumulated in one round trip of a cavity is

10.2 $$\begin{aligned}
								\varPsi = 2(\psi _2-\psi _1) = 2\left( \tan ^{-1}\left(
								\frac{z_2}{z_R}\right) - \tan ^{-1}\left( \frac{z_1}{z_R}\right)
								\right) \end{aligned}$$

where $$\psi
								_{1/2}$$ is the Gouy phase at the input/end mirror, $$z_{1/2}$$ is the distance from the waist of the input/end mirror and $$z_{R}$$ is the Rayleigh range, all in terms of the cavity eigenmode. The different resonant tunings for various HOM in a cavity is illustrated in Fig. 98, where an Advanced LIGO cavity is simulated with an input beam made up of equal parts of 6 different order modes, orders 0–5. Each of the higher-order modes is resonant at a different microscopic tuning; a cavity operated on resonance for the fundamental mode (order 0) will suppress the power in the higher-order modes circulating in the cavity and transmitted by the cavity, as these are not resonant at the same tuning. In consequence the higher-order modes are reflected by the cavity. The parameters for the Gaussian eigenmode of an Advanced LIGO arm cavity are summarised in Table 1. This includes half the round-trip Gouy phase, in this case 24.3$$^{\circ
								}$$, which gives the spacing between subsequent resonance peaks in terms of cavity tuning (as seen in Fig. 98).

**Fig. 98:**
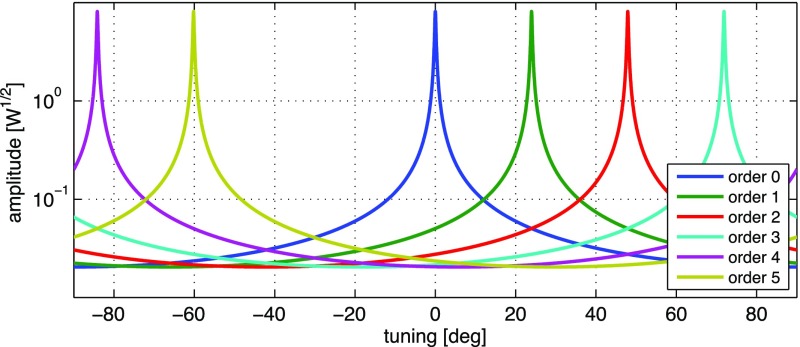
Amplitude of 6 higher-order modes (orders 0–5) circulating in an optical cavity as the microscopic length is tuned. The fundamental mode (order 0) is resonant at 0$^{\circ }$ tuning and the mode separation tuning, 24$^{\circ }$ is defined by the length of the cavity and the mirror curvatures

This property of an optical cavity to act as a filter of spatial modes is utilised in gravitational-wave detectors. Firstly, the input laser beam is ‘cleaned’ of spatial modes by passing through an *input mode cleaner*, an optical cavity carefully designed to transmit the fundamental mode and filter out most higher-order modes before the beam enters the main interferometer. Within the multiple cavities of the central interferometer careful design can take advantage of these resonant properties to suppress distortions of the beam. Finally, the output beam containing the gravitational wave signal is cleaned of spatial modes and control sidebands using an *output mode cleaner*. These design features are discussed in grater detail in Sect. 10.7.

### Cavity alignment in the mode picture

In the previous example the injected beam contains several different order modes. This is an exaggeration of the effect of a mode cleaner, where a distorted beam is cleaned of unwanted spatial modes. After the mode cleaner the input beam is well described by the fundamental mode and higher-order modes present in interferometers can be the result of defects in the optics and mismatches between the incoming beam and eigenmode of the interferometer. The simplest example of this is a misaligned 2-mirror cavity, where the optical axis of the incoming beam is not aligned to the optical axis of the cavity.

**Table 1:** Summary of the parameters defining the Gaussian eigenmode of an Advanced LIGO arm cavity

$R_{C,1}$ (m)	$R_{C,2}$ (m)	$w_0$ (cm)	$w_1$ (cm)	$w_2$ (cm)	$z_1$ (m)	$z_2$	$z_R$ (m)	$\frac{\varPsi }{2}$ ($^{\circ }$)
1934	2245	1.2	5.3	6.2	$-$1834	2160	425	24.3

This includes the radius of curvature, $$R_C$$, at the input (1) and end (2) mirrors; the beam spot size, *w*, at the input mirror, end mirror and at the beam waist (0); the distance from the waist, *z*, of the input and end mirrors; the Rayleigh range, $$z_R$$; and half the round-trip Gouy phase, $$\frac{\varPsi
											}{2}$$

**Fig. 99:**
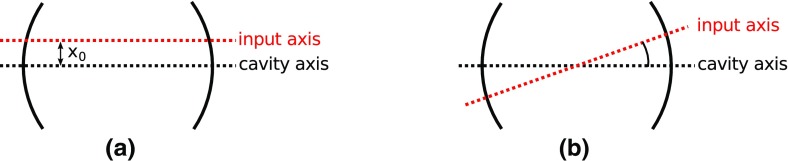
Different possible misalignments of an optical cavity. **a** Constant *x* displacement of the input beam optical axis with respect to the cavity axis. **b** Relative tilt between the input optical axis and the cavity axis

As discussed by Anderson (1984) and illustrated in Fig. 99 we can consider different possible misalignments. Any misalignment can be split into a displacement in *x* (or *y*) of the input beam axis with respect to the cavity axis (a) and a relative tilt between the input beam and cavity axes (b). For mathematical simplicity we consider a fundamental Gaussian beam at the waist. As the Hermite–Gauss modes are separable in *x* and *y* we just consider the *x* component. The results are equivalent for a displacement in *y*. The fundamental mode ($n=0$) and first order mode ($n=1$) of the cavity can be written

10.3 $$\begin{aligned}
								\begin{array}{cccc} u_0(x) = \left( \frac{2}{\pi w_0^2}\right)
								^{1/4} \exp {\left( -\frac{x^2}{w_0^2}\right)
								}&\,&u_1(x) = \left( \frac{2}{\pi w_0^2}\right)
								^{1/4} \frac{2 x}{w_0} \exp {\left( -\frac{x^2}{w_0^2}\right) }
								\end{array} \end{aligned}$$

where $w_0$ is the beam waist size. Assuming the input beam matches the cavity eigenmode, with the exception of the misalignment, a displacement of the input beam (a) is translated onto the cavity axis as

10.4 $$\begin{aligned}
								u_{\mathrm {disp.}}(x) = u_0(x-x_0) = \left( \frac{2}{\pi
								w_0^2}\right) ^{1/4} \exp {\left( -\frac{(x-x_0)^2}{w_0^2}\right) }.
								\end{aligned}$$

As long as the displacement, $$x_0$$, is small compared to the beam size ($$\frac{x_0}{w_0}
								\ll 1$$) any second order terms and higher in $$\frac{x_0}{w_0}$$ can be ignored and the input field is approximated as

10.5 $$\begin{aligned}
								u_{\mathrm {disp.}}(x) \approx \left( \frac{2}{\pi w_0^2}\right)
								^{1/4} \left( 1 + \frac{2xx_0}{w_0^2}\right) \exp {\left(
								-\frac{x^2}{w_0^2}\right) } = u_0(x) + \frac{x_0}{w_0} u_1(x).
								\end{aligned}$$

Thus this displacement of the input beam, with respect to the cavity, is equivalent to the addition of a first order Hermite–Gauss mode.

Similarly a misalignment in terms of a tilt of the input axis with respect to the cavity axis (b) can be described by the addition of a first order mode (Anderson 1984). In this case the amplitude of the input field as projected onto the cavity axis is un-altered, for small tilts, and the relative misalignment only effects the phase of the beam

10.6 $$\begin{aligned}
								u_{\mathrm {tilt.}}(x) = u_0(x)\exp {\left( \mathrm {i}\,k \sin
								{(\alpha ) x}\right) } \approx u_0(x)\exp {\left( \mathrm {i}\,k
								\alpha x \right) }. \end{aligned}$$

For a small misalignment the higher-order terms of the exponential are ignored:

10.7 $$\begin{aligned}
								u_{\mathrm {tilt.}}(x) \approx u_0(x) \left( 1 +\mathrm {i}\,k
								\alpha x\right) = u_0(x) + \mathrm {i}\,\frac{k \alpha w_0}{2}
								u_1(x). \end{aligned}$$

The relative tilt of the input beam is expressed with the addition of an order 1 mode, 90$$^{\circ
								}$$ out of phase with the fundamental mode. This 90$$^{\circ
								}$$ phase factor is a useful feature which can be used to separate the order 1 modes caused by a displacement of the optical axis and those caused by a tilt of the optical axis. A combination of these two types of misalignment can describe any misaligned cavity (providing the misalignment is small).

The consequence of a misalignment of an optical cavity is the creation of first order modes. If we chose to describe the problem using the Gaussian beam parameters and axis of the incoming beam as our basis, then the incoming beam is a pure fundamental beam and the first order modes are created when the light enters (and leaves) the cavity. Alternatively we can use the cavity eigenmode and cavity axis as our basis. In this case the higher-order modes are already present in the incoming beam. Either of these approaches is valid for such a simple distortion.

In more realistic cases the circulating field in a cavity is not completely described by a fundamental Gaussian beam, due to deviations of real mirrors from an ideal sphere. This can be modelled using the closest Gaussian eigenmode (from now on referred to as the eigenmode of the cavity) superimposed with higher-order modes. One can say that the higher-order modes are created when the fundamental mode interacts with the distorted mirrors.

To describe the input–output relations of a cavity for higher-order modes it is important to know at which location they have been created, in other words where they enter the cavity. For higher-order modes present in the input beam (not created inside the cavity) the amplitude of these modes in the circulating field is given by:

10.8 $$\begin{aligned}
								a_{n,m}^{\mathrm {circ.}} = \frac{\mathrm {i}\,r_2 t_1 \exp {\left(
								-\mathrm {i}\,2kL + \mathrm {i}\,(1+n+m) \varPsi \right) }}{
								1-r_1r_2\exp {\left( -\mathrm {i}\,2kL +\mathrm {i}\,(1+n+m)\varPsi
								\right) }} a_{n,m}^{\mathrm {in}},
								\end{aligned}$$

where $$a_{n,m}^{\mathrm
								{in}}$$ is the amplitude of the HOM in the incoming field. The equation is very similar to that for a plane wave [Eq. (2.3)] with the addition of the Gouy phase picked up for different modes. On the usual operating point of resonance for the fundamental mode this simplifies to:

10.9 $$\begin{aligned}
								a_{n,m}^{\mathrm {circ.}} = \frac{\mathrm {i}\,r_2 t_1 \exp {\left(
								\mathrm {i}\,(n+m) \varPsi \right) }}{ 1-r_1r_2\exp {\left( \mathrm
								{i}\,(n+m)\varPsi \right) }} a_{n,m}^{\mathrm {in}}.
								\end{aligned}$$

A well designed cavity will have $$(n+m)\varPsi \ne
								N 2\pi $$ up to a high mode order to prevent other modes resonating. In the case where we consider higher-order modes created at individual mirrors, the equations are different. If the modes are created at the end mirror, with no higher-order modes present in the input beam, the circulating field is approximated as:

10.10 $$\begin{aligned}
								a_{n,m}^{\mathrm {circ.}} \approx \frac{\mathrm {i}\,r_2 t_1 \exp
								{\left( \mathrm {i}\,(n+m)\frac{\varPsi }{2}\right)
								}}{(1-r_1r_2)(1-r_1r_2\exp {\left( \mathrm {i}\,(n+m)\varPsi \right)
								)}} k_{0,0,n,m} \ a_{0,0}^{\mathrm {in}},
								\end{aligned}$$

where $k_{0,0,n,m}$ is a coupling coefficient describing the phase and amplitude of the mode HG$_{nm}$ created at the end mirror, due to an incident HG$_{00}$ mode. The approximation here assumes the coupling is small and so does not include the loss of power from the HG$_{00}$ mode and coupling from the HG$_{nm}$ mode back into HG$_{00}$. This approximation is valid for small distortions, but including distortions on all the optics will quickly become very complicated analytically, and it is for such problems that simulation tools such as Finesse are valuable.

**Fig. 100:**
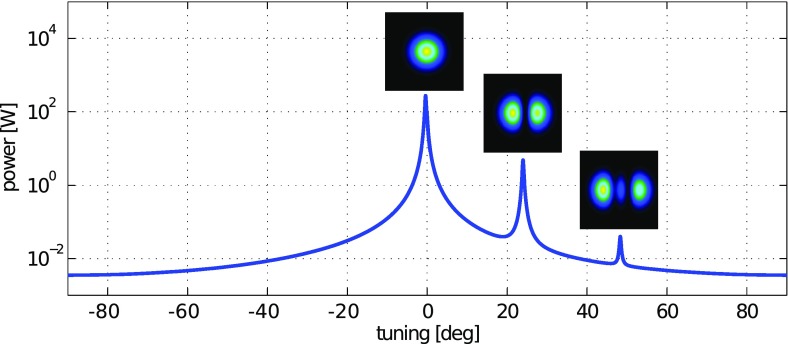
Circulating power in an Advanced LIGO cavity with a 0.3 $$\upmu $$rad misalignment applied to the end mirror. The cavity is tuned and the circulating beam is detected for each peak in intra-cavity power. Most of the power remains in the fundamental mode, resonant at $${\sim } 0^{\circ
											}$$, with some coupling into HG$$_{10}$$, resonant at $${\sim } 24^{\circ
											}$$, and HG$$_{20}$$, resonant at $${\sim } 50^{\circ
											}$$

In Fig. 100 the effects of misalignment on intra-cavity power is illustrated. In this example an Advanced LIGO cavity has been modelled with a misaligned end mirror. The circulating power exhibits several peaks, corresponding to the resonances of the different higher-order modes created due to the misalignment. Most of the power remains in the fundamental mode (peak at $${\sim } 0^{\circ
								}$$). The misalignment of the cavity has induced higher-order modes, mostly the first order HG$$_{10}$$ mode, whose resonance is observed at $${\sim }
								24^{\circ }$$. In this case the extent of the misalignment also results in the creation of the order 2 mode HG$$_{20}$$, resonant at $${\sim }
								50^{\circ }$$. During operation, where the cavity is on resonance for the HG$$_{00}$$ mode, the relative power in higher-order modes is suppressed. There will still be some higher-order modes in the beam at this tuning, which degrade the purity of the beam transmitted and reflected from the cavity.

### Mode mismatch

**Fig. 101:**
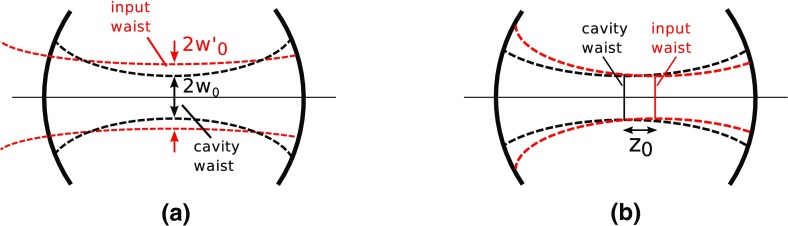
Different possible mode mismatch between an the eigenmode of an optical cavity and an injected laser beam. **a** Beam size mismatch. **b** Mismatch of the position of the beam waists

Another common defect of optical cavities with respect to an input laser beam is known as *mode mismatch* (Mueller et al. 2000). Whilst (a small) misalignment is a first order effect described by first order modes, a (small) mode mismatch is a second order effect, where the wavefront curvature or beam size of the incoming beam does not match that of the cavity eigenmode. Such effects are described primarily by second order modes. As with misalignment this defect can be split into two different effects: beam size mismatch and waist position mismatch, as shown in Fig. 101.

In the case of a pure beam size mismatch, with the cavity and input beam waists located at the same point along the optical axis the input beam can be described in the cavity basis as (Anderson 1984):

10.11 $$\begin{aligned}
								u_{\mathrm {size.}}(r) = u_0(r) + \epsilon \ u_2(r),
								\end{aligned}$$

where $$u_0$$ is the fundamental cavity mode, $$\epsilon
								$$ is the fractional difference in the input beam size to the beam size of the cavity eigenmode, $$w_0' =
								(1+\epsilon ) w_0$$, and $$u_2$$ is the second order Laguerre–Gauss mode of the cavity, with no angular dependence (LG$$_{10}$$)

10.12 $$\begin{aligned}
								u_2(r) = \sqrt{\frac{2}{\pi }} \frac{1}{w_0} \left(
								1-\frac{2r^2}{w^2_0}\right) . \exp {\left( -\frac{r^2}{w_0^2}\right)
								} \end{aligned}$$

In this case the calculation is performed at the waist for simplicity. Similarly, for a purely waist position mismatch we have

10.13 $$\begin{aligned}
								u_{\mathrm {posit.}}(r,z) = u_0(r,z) + \mathrm
								{i}\,\frac{kz_0}{w_0^2} u_2(r,z),
								\end{aligned}$$

where $$z_0$$ is the displacement of the input beam waist with respect to the cavity waist, and the fundamental and second order modes are now in their more general form, taking into account a finite radius of curvature. As with misalignment we find that the two types of mode mismatch result in the creation of the same mode, with one in phase (beam size) and one with a 90$$^{\circ
								}$$ phase shift (waist position) with respect to the fundamental mode.

In Fig. 102 the circulating power in a mismatched cavity is shown. In this case the mismatch is a 25 % mismatch purely in beam size. Most of the power remains in the fundamental mode (resonant at 0$$^{\circ
								}$$), with most of the mismatch described by the order 2 mode LG$$_{10}$$ (resonant at $${\sim }
								50^{\circ }$$). Such a large mismatch results in additional modes with even mode orders: the order 4 mode LG$$_{20}$$ and order 6 mode LG$$_{30}$$.

**Fig. 102:**
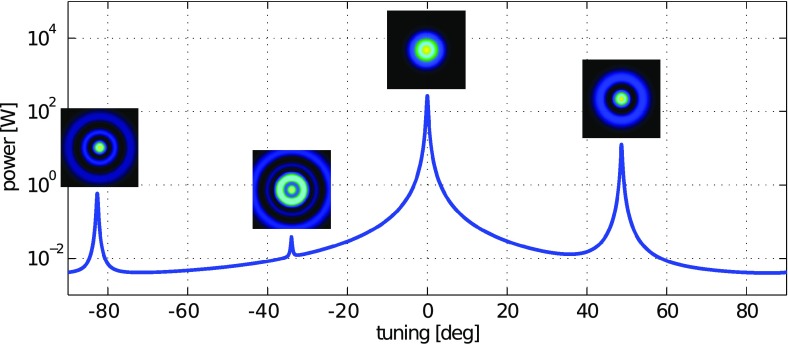
Circulating power in a Advanced LIGO cavity with a 25 % mismatch in beam size between the injected beam and the cavity eigenmode. The intra-cavity power is detected as the longitudinal length is scanned, with the beam detected at each local resonance. Most of the power is in the fundamental mode, resonant at $${\sim } 0^{\circ
											}$$, with the mismatch represented by power in the order 2 mode LG$$_{10}$$ ($${\sim } 50^{\circ
											}$$), the order 4 mode LG$$_{20}$$ ($${\sim } -80^{\circ
											}$$) and the order 6 mode LG$$_{30}$$ ($${\sim } -35$$)

### Spatial defects

Misalignment and mode mismatch are the lowest order distortions of the beam and are well described analytically. These low order distortions are carefully controlled in an interferometer, using alignment control schemes and using lenses and curved optics to mode match beams between different cavities. Higher-order distortions produced from more complex processes, i.e., interaction with distorted mirror surfaces or finite sized optics, cannot currently be controlled. There are many different spatial defects which are likely to be present in real interferometers.

For the design and commission of real detectors we want to represent these more arbitrary defects, in particular the deviation of the mirror surfaces from a perfect sphere. In the case of interferometer design this will help set requirements on the polishing and coating of the mirrors. For the commissioning process this will aide in identifying the output beam shape and other effects associated with distortions of the beam. In this article we will focus on mirror surface errors and thermal effects. The detailed mathematics of these higher-order effects are discussed in Sect. 11. For now we just consider that higher-order modes are created when beams are distorted. The advantage of describing distortions of the beam as higher-order modes is that these spatial modes are easy to trace through the interferometer, to predict the behaviour of a distorted interferometer.

### Operating cavities at high power

Advanced gravitational-wave detectors will operate with very high light power in the arm cavities, to increase the signal compared to the shot noise. In Advanced LIGO, for example, the power in the arms will approach 1 MW (Advanced LIGO Team 2007). Even the state of the art optics used in advanced gravitational-wave detectors will absorb a proportion of the incident power. The mirrors produced for Advanced LIGO have requirements of $${<}0.5$$ parts-per-million (ppm) power absorption. With the expected power in the arms this means $${\sim }
								0.5$$ W will be absorbed in the mirror coatings and substrates. During operation this absorption of power will lead to a temperature gradient evolving in the optics, starting from a *cold state* defined by the temperature of the environment and gradually developing a temperature field across the optic, with a hot point at the centre where the beam is most intense. Finally the temperature field of the mirrors will reach a steady state, where the optic is in thermal equilibrium. The development of such temperature gradients in the mirrors will result in two types of mirror aberration:

1. A thermal lens forms within the mirror due to the temperature dependent nature of the index of refraction of the substrate material (fused silica). This distortion can be described mostly as a spherical lens, with some higher-order components (Hello and Vinet 1990; Vinet and the Virgo Collaboration 2001).
2. The mirror expands thermally, with the expansion greatest where the mirror is hottest, giving a non-uniform expansion over the mirror surface and effectively distorting the surface from the cold case. This thermal distortion is primarily a change in the radius of curvature of the mirror (Vinet and the Virgo Collaboration 2001; Hello and Vinet 1990).

Both these effects will impact the shape of the beam in the arms, the thermal lens in the input mirror distorting the beam injected into the cavities and the surface distortions of the cavity mirrors changing the shape of the beam resonating in the arms. These effects will be primarily second order effects, impacting the mode matching of the beam into the arm cavities and the resonating eigenmode. Crucially these effects are not constant: the temperature fields and thermal aberrations will evolve from the cold state to thermal equilibrium, where this equilibrium state, or *hot state*, is dependent on the interferometer input power. For example, Advanced LIGO is expected to operate within a range of input powers up to $${\sim
								}$$100 W.^16^ The transitory nature of these thermal aberrations will be one of the key challenges for advanced interferometers. Effectively the input mode and cavity eigenmodes are constantly developing and require additional systems to control the resonating mode of the interferometer. For a more detailed description of the evolution of these thermal effects please refer to the Living Reviews article by Vinet (2009). Here we will attempt to quantify this problem and motivate the need for thermal compensation systems to correct the lensing and change in curvatures of the mirrors at high power.

Firstly, we consider a single arm cavity of the second generation gravitational-wave detector Advanced LIGO (Arain et al. 2012). The two mirrors which make up this cavity, the input test mass (ITM) and end test mass (ETM) are separated by $${\sim }
								4$$ km. The radii of curvature and optical parameters are given in Table 2. These numbers refer to the curvature of the mirrors in the cold state, before heating of the mirror from the laser beam. During operation the mirrors will heat up as they absorb power from the laser beam, creating a thermal lens in the ITM and distorting the reflective surfaces of both mirrors. Advanced LIGO is expected to operate within a range of input powers. Here we will refer to the cold case (0 W input power), low power (12.5 W input power) and high power (125 W input power). This gives a maximum of 800 kW in the arm cavities at high power. The thermal lensing and distortions for each case are summarised in Table 3, calculated using the Hello–Vinet method (Vinet and the Virgo Collaboration 2001; Hello and Vinet 1990).

**Table 2:** The design geometric and optical parameters of an Advanced LIGO arm cavity

	$R_C$ (m)	Transmittance	Loss (ppm)
ITM	1934	1.4 %	37.5
ETM	2245	5 ppm	37.5

The radius of curvature ($R_C$), proportion of power transmitted and proportion of power lost for the input test mass (ITM) and end test mass (ETM) are given. ppm refer to parts-per-million (Arain et al. 2012)

**Table 3:** The expected thermal aberration of the test masses in an Advanced LIGO arm cavity, as calculated using the Hello–Vinet method (Vinet and the Virgo Collaboration 2001; Hello and Vinet 1990), for 3 states of operation corresponding to different input powers

	$f_\mathrm{{ITM}}$ (km)	$\delta R_{C,\mathrm {ITM}}$ (km)	$\delta R_{C,\mathrm {ETM}}$ (km)
Cold case (0 W)	$\infty$	$\infty$	$\infty$
Low power (12.5 W)	50	1100	1600
High power (125 W)	5	110	160

The aberrations are well described by second order effects: a spherical lens in the ITM characterised by focal length $$f_\mathrm{{ITM}}$$ and distortions of the reflective surfaces of the mirrors characterised by a change in curvature $$\delta R_{C,\mathrm
											{ITM}}$$/$$\delta R_{C,\mathrm
											{ETM}}$$. In these cases of relatively low power absorption the distortions scale linearly with power

The thermal lens is the dominant aberration and will have a large impact on the beam injected into the cavity. However, it will not impact the eigenmode of the cavity, this is determined purely by the curvature of the highly reflective mirror surfaces.

**Fig. 103:**
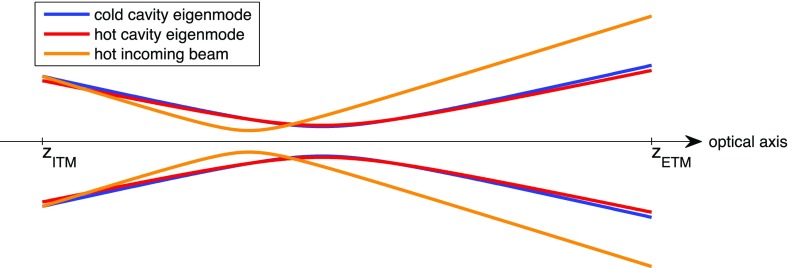
Plots of the predicted beam sizes for different states (hot and cold) of an Advanced LIGO arm cavity. In the cold case the cavity eigenmode (*blue curve*) is defined by the radius of curvature of the cold optics (0 W input power). We assume the incoming beam is mode matched to the cold cavity. The hot case refers to high power operation (125 W input power) where the mirrors absorb a proportion of the laser power. The hot cavity eigenmode (*red curve*) is determined by the curvature of the reflective surface of the hot optics: the mirror expands elastically, reducing the curvature. This changes the eigenmode of the cavity. In the hot case the input beam (*orange curve*) will no longer be matched to the cavity eigenmode, as it passes through the strong thermal lens ($f=5$ km) in the ITM. During high power operation *thermal compensation systems* will be used to correct the curvatures of the mirrors back to their cold state and compensate the lens in the ITM, with the aim of keeping the interferometer modes well matched and consistent for a range of input powers

Consider an individual arm cavity with an incoming laser mode matched to the cold optics (the design curvatures). The size of the beam corresponding to this cold eigenmode is plotted in blue in Fig. 103 along the length of the cavity. Next we consider the hot eigenmode of the cavity, in this case corresponding to the curvatures of the cavity during high power operation (125 W). This is plotted in red. The mirrors are less curved and the eigenmode differs slightly from the cold case, with a slightly smaller beam size at the input and end mirror and a larger waist. The mismatch between the two eigenmodes is relatively small. The incoming beam, however, is strongly mismatched between the possible cavity eigenmodes (shown plotted in orange). During high power operation the injected beam will experience a strong 5 km lens in the ITM, focussing the beam and shifting the waist closer to the ITM, resulting in a larger beam at the ETM.

**Table 4:** Beam parameter, *q*, beam size, *w*, wavefront curvature, $R_C$ and distance from the wasit, *z* of 3 different Gaussian beams, the eigenmode of an advanced LIGO cavity during cold operation (0 W input power) and during hot operation (125 W input power) and the input beam during hot operation

	*q* (m)	*w* (cm)	$R_C$ (m)	*z* (m)
Cold eigenmode	$-1834.2 + 427.8\mathrm {i}\,$	5.30	$-1934$	$-1834.2$
Hot eigenmode	$-1832.7 + 499.0\mathrm {i}\,$	4.95	$-1968.6$	$-1832.7$
Hot input beam	$-1356.2 + 228.1\mathrm {i}\,$	5.30	$-1394.6$	$-1356.2$

In reality the hot eigenmode and input beam will develop over time as the mirrors heat up and the aberrations evolve. This takes us from the cold case, where the incoming beam is well matched to the cavity, to the hot case where there is a strong mode mismatch. This will have a strong impact on the power injected into the cavity. During operation the arm cavities are ‘locked’ to the resonance of the fundamental cavity mode. In this state the components of the injected beam which do not overlap with the cavity eigenmode will be reflected. As was discussed in Sect. 10.3 these will be primarily order 2 modes.

Table 4 lists the beam parameters for the 3 different Gaussian beams: the cold and hot cavity eigenmodes, calculated from the radii of curvature of the hot and cold optics, and the hot input beam, calculated using an ABCD matrix for a 5 km lens (see Sect. 9.13). To estimate how much power will be injected into the hot cavity we calculate the overlap between the hot cavity eigenmode and hot input beam, i.e., we want to know how much power in the input beam is in the 00 mode of the cavity eigenmode. This takes the form

10.14 $$\begin{aligned}
								c = \int _A u_{\mathrm {in}} u^*_{\mathrm {cav}} \, \mathrm {d}A,
								\end{aligned}$$

where $$u_{\mathrm
								{in}}$$ is the input field, $$u_{\mathrm
								{cav}}$$ is the cavity eigenmode, *S* is an infinite surface perpendicular to the optical axis and the percentage of input power in the cavity eigenmode is given by $$|c|^2$$. In this case both beams are cylindrically symmetric fundamental Gaussian beams (not astigmatic) and the overlap can be calculated as

10.15 $$\begin{aligned}
								c = \frac{2}{\pi }\frac{1}{w_{\mathrm {in}}w_{\mathrm {cav}}} \exp
								{\left( \mathrm {i}\,\varPsi _{\mathrm {in}}-\mathrm {i}\,\varPsi
								_{\mathrm {cav}}\right) } \int _0^{2\pi } \int _0^{\infty } \exp
								{\left( -\frac{\mathrm {i}\,kr^2}{2}\left( \frac{1}{q_{\mathrm
								{in}}}-\frac{1}{q_{\mathrm {cav}}^*}\right) \right) } r \, \mathrm
								{d}\phi \, \mathrm {d}r . \end{aligned}$$

Integrating with respect to $$\phi
								$$ and changing variables to $$R=r^2$$ we have

10.16 $$\begin{aligned}
								c = \frac{2}{w_{\mathrm {in}}w_{\mathrm {cav}}} \exp {(\mathrm
								{i}\,\varPsi _{\mathrm {in}} - \mathrm {i}\,\varPsi _{\mathrm
								{cav}})} \int _0^{\infty } \exp {\left( -\frac{\mathrm
								{i}\,kR}{2}\left( \frac{1}{q_{\mathrm {in}}} - \frac{1}{q_{\mathrm
								{cav}}^*}\right) \right) } \, \mathrm {d}R .
								\end{aligned}$$

**Fig. 104:**
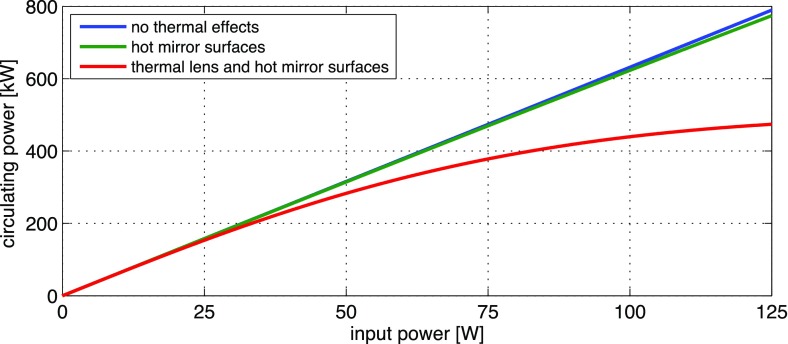
The simulated circulating power in an Advanced LIGO arm cavity versus input laser power into the power recycled interferometer with linear arm cavities. Three cases are simulated: no thermal effects, where the response is linear; including the change in curvature of the mirror surfaces due to thermal effects; and including both the change in curvature of the mirrors and the thermal lens induced in the input mirror due to power absorbed in the mirrors. The addition of the thermal lens has the greatest impact, reducing the power coupled into the arm cavities

As $${\mathfrak
								{R}}\left\{ \frac{\mathrm {i}\,k}{2}\left( \frac{1}{q_{\mathrm
								{in}}}-\frac{1}{q_{\mathrm {cav}}^*}\right) \right\} >
								0$$ the solution of this equation can be written as (Gradshteyn and Ryzhik 1994)

10.17 $$\begin{aligned}
								c = -\frac{4 \mathrm {i}\,}{w_{\mathrm {in}}w_{\mathrm {cav}} k}
								\exp {(\mathrm {i}\,\varPsi _{\mathrm {in}}-\mathrm {i}\,\varPsi
								_{\mathrm {cav}})} \frac{1}{\frac{1}{q_{\mathrm
								{in}}}-\frac{1}{q_{\mathrm {cav}}^*}} .
								\end{aligned}$$

Using this formula we calculate the overlap between the hot cavity eigenmode and hot input beam as $|c|^2=52.5\,\%$. Such a large mismatch between the incoming beam and cavity would therefore result in around half the circulating power expected from a plane wave model or with a perfectly mode-matched beam. This is illustrated in Fig. 104, which shows the power circulating in a single arm cavity as the input power is increased from 0 to 125 W, taking into account the 50:50 beam splitter and assuming a power recycling gain of 45 (in reality this will also be impacted by thermal effects). When no thermal effects are included the increase in circulating power is linear. Including the thermally induced reduction in curvature of the mirror surfaces, with the input beam remaining mode matched to the cold cavity, causes a small mismatch, which, at high powers can be noted in a reduction in the gain of the cavity. The largest effect of the internal heating is in the creation of the thermal lens in the ITM, which induces a large mismatch between the beam injected into the cavity and the cavity eigenmode. This is reflected in a large reduction in the circulating power at high input powers, around the 50 % reduction predicted by the overlap between the hot input beam and cavity eigenmode. In this simple model we assume a linear scaling of the thermal distortions and lenses with input power. As these aberrations are determined by the circulating power, which no longer scales linearly with input power, this is a slight over estimation of the power loss. In reality this process is more complicated: as the mirrors heat and their aberrations evolve the circulating power will decrease, but this will then reduce the thermal lensing and distortions. The expected stable cavity will therefore be slightly different to the cases shown here. However, these plots illustrate the magnitude of the problem: such extreme mode mismatches are unacceptably high. We require some method to compensate these effects, especially the thermal lensing. Such compensation will need to be adaptive, to be applicable for a range of input powers and for the transition from cold to hot, controlling the mode resonating in the interferometer as the power is increased and the mirrors reach thermal equilibrium. In reality the thermal aberrations will differ slightly from our models and more importantly the aberrations in each arm will differ from each other, due to differences in absorption for the individual mirrors. The *thermal compensation systems* need to act on individual mirrors, incorporating sensors which monitor the current state of the thermal aberrations in each arm and then feed back to systems which can correct the curvature of the mirror surfaces and the lenses in the ITMs. These compensation systems are discussed in more detail in Sect. 10.7 and in the comprehensive review article by Vinet (2009).

### The Michelson: differential imperfections

Previously we motivated operating a Michelson interferometer on the dark fringe in order to maximise the differential gravitational wave signal and minimise the noise at the dark port (see Sect. 5.3). The differential degrees of freedom, the Michelson (MICH) and differential arm length (DARM), are carefully controlled to maintain the interferometer on the dark fringe, as discussed in Sect. 8.12. A well defined dark fringe relies on the fact that the two arms are very similar and essentially the carrier and any common mode effects cancel at the dark port. Simply we can express the field reflected back towards the laser at the symmetric port as

10.18 $$\begin{aligned}
								E_\mathrm{{sym.}} = \frac{1}{\sqrt{2}} \left( E_x+E_y\right) ,
								\end{aligned}$$

where $E_x$ and $E_y$ refer to the fields coming from the individual arms. The field in the asymmetric port, or output port is

10.19 $$\begin{aligned}
								E_\mathrm{{asym.}} = \frac{1}{\sqrt{2}}\left( E_x-E_y\right) .
								\end{aligned}$$

In the case where each arm is identical the symmetric field is $$E_\mathrm{{sym.}} = \sqrt{2}
								E_\mathrm{{arm}}$$, where $$E_\mathrm{{arm}}$$ is the field reflected from an individual arm, and the asymmetric field is $$E_\mathrm{{asym.}} = 0$$. For a generic interferometer any field components common to both arms cancel at the asymmetric port whilst any differential components cancel in the symmetric port: the field reflected from the interferometer is the common mode; the field exiting the interferometer is the differential mode. Previously we have considered the carrier and any noise coming from the laser to be common mode (reflected back towards the laser) whilst the asymmetric port is dominated by the differential gravitational wave signal, any differential noise and potentially a small proportion of leaked carrier light for DC readout, see Sect. 5.4. However, a complex realistic interferometer, such as gravitational wave detectors, contains imperfections and deviations from specifications that lead to additional fields at the asymmetric port. For example:

- Differences in the loss and finesse of each arm result in different carrier amplitudes in each arm, degrading the interference of the two beams at the dark fringe and leading to additional carrier light at the output port.
- Different resonant or interference conditions for the carrier and control sidebands. Advanced gravitational-wave detectors such as Advanced LIGO employ a Schnupp modulation scheme, see Sect. 8.13, to control the interferometer, where by an asymmetric length applied to the short Michelson arms ensures that the dark fringe of the carrier is not equivalent to the dark fringe of the control sidebands, resulting in a proportion of these radio frequency sideband fields at the dark port.
- Spatial differences between the beams coming from each arm. An imperfect overlap of the spatial distribution of these beams will degrade their interference and cause light in higher-order modes to leak into the dark port.

In the specific examples discussed here we will focus on the impact of higher-order modes at the detector output. These fields do not contain the gravitational wave signal but will carry noise to the output port. This will not only increase the contributions from expected differential mode noises but can couple common mode noise, such as laser noise, into the output channel. Such additional light fields can be refereed to as excess light, defined as light fields exiting the interferometer which do not contribute to the signal readout. This should not be confused with the local oscillator fields, such as the leaked local carrier light in the DC readout scheme.

A figure of merit for the excess light leaving a Michelson interferometer is the *contrast defect*. This is the ratio of the excess light exiting through the dark port to the light circulating in the interferometer and, for an interferometer on the dark fringe, is calculated as

10.20 $$\begin{aligned}
								C = \dfrac{ \displaystyle \int _A | E_x-E_y|^2 \ \mathrm {d}A
								}{\displaystyle \int _A |E_x +E_y|^2 \ \mathrm {d}A} .
								\end{aligned}$$

In a Michelson with no differential spatial effects the contrast defect is determined by the differential losses in the arms and at the beam splitter. In reality slight differences in mirror curvatures, distortions of the mirror surfaces and limits to alignment control will result in different spatial features in each arm, appearing as higher-order modes in the output port and increasing the contrast defect. The mirrors for each arm of the detector are manufactured to be very similar in terms of their optical properties, which will determine differential arm losses; and their geometric and thermal properties, which will determine the higher-order mode content in each arm. In Advanced LIGO the contrast defect should be lower than a few hundred ppm ($${\sim } 2 \times
								10^{-4}$$W/W) (Aasi 2015). In addition the higher-order modes can be suppressed using an output mode cleaner.

An example of the impact of higher order modes on contrast defect in a dual recycled Michelson have been observed at GEO 600, the German–British gravitational-wave detector in Hannover (Danzmann et al. 1994; Abbott 2004). Unlike other gravitational-wave detectors GEO 600 does not include arm cavities, but instead has folded arms to increase the effective arm length of the Michelson. As described in Lück et al. (2004), during the operation of GEO 600 it was discovered that a difference in the radii of curvature of the folding mirrors in the *x* and *y* arms (687 m in the *x* arm, 666 m in the *y* arm) was causing a significant difference in the wavefront curvatures of the beams returning from each arm. This mismatch between the two beams resulted in a significant amount of power at the interferometer dark fringe: the degrade in overlap between the two beams reduced the effective destructive interference and consequently the output beam on the dark fringe was dominated by the order 2 mode typical of a mode mismatch, LG$$_{10}$$. The resulting loss of power into the anti symmetric port increased the effective loss in the power recycling cavity, limiting the power build up to $${\sim }
								200$$ W/W, a significant reduction from the 300 W/W predicted for this configuration. The mismatch of the two arms also had a negative impact on the longitudinal error signal of the Michelson, reducing the magnitude of the error signal and increasing the susceptibility to misalignments.

To reduce this mismatch and recover the power recycling gain the curvature of one of the folding mirrors required correcting, to match that of the other arm. In this case the thermal properties of the mirrors were exploited, namely the dependence of the radius of curvature of the mirrors to a temperature gradient, as was discussed in Sect. 10.5. In advanced detectors the temperature gradient which develops from high powered beams incident on the mirrors is an unwanted effect which results in the distortions of the mirror surfaces (primarily a change in curvature) and thermal lensing. In GEO 600 this thermal behaviour was manipulated to alter the curvature of the folding mirror in the East arm (*x* arm) using a ring heater placed behind the mirror substrate to produce an appropriate temperature gradient in the East mirror (Lück et al. 2004). The extent of the change in mirror curvature is dependent on the ring heater power, which can be gradually altered to find the power which corresponds to the optimum curvature (i.e., $${\sim }
								666$$ m to match the North mirror).

**Fig. 105:**
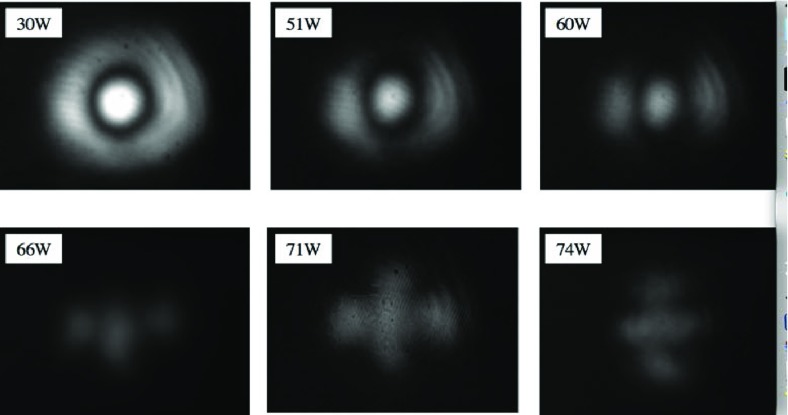
Interference pattern at the dark port of GEO 600, for different thermal compensation of the East arm folding mirrors, indicated by the different ring heater powers. The brightness of the beam images in the *bottom row* are slightly enhanced for better visibility. Image reproduced with permission from Lück et al. (2004), copyright by IOP

In Fig. 105 the interference pattern at the dark fringe is shown for different ring heater powers. For relatively low powers (30 W) the two arms are still not well matched and the dark port is dominated by the typical bullseye shape of the mismatch mode, LG$_{10}$. As the ring heater power is increased from 30 to 66 W the mode matching between the two arms increases and the power at the dark fringe is reduced, improving the contrast defect by an order of magnitude. Increasing the ring heater power to 71 W further optimised the dark fringe, in terms of minimum power at the dark port. At this point the limitations of this curvature compensation are observed: the compensation is applied as a spherical curvature correction and does not take into account differences in curvature in the horizontal and vertical directions, i.e. astigmatism of the mirrors or beam splitter. For ring heater powers of 66–74 W the output mode is still dominated by order two modes but in this case these are the Hermite–Gauss modes consistent with astigmatic mismatches. At 66 W the mismatch between the two arms is compensated in the vertical direction whereas optimum compensation in the horizontal direction requires a ring heater power of 74 W.

**Fig. 106:**
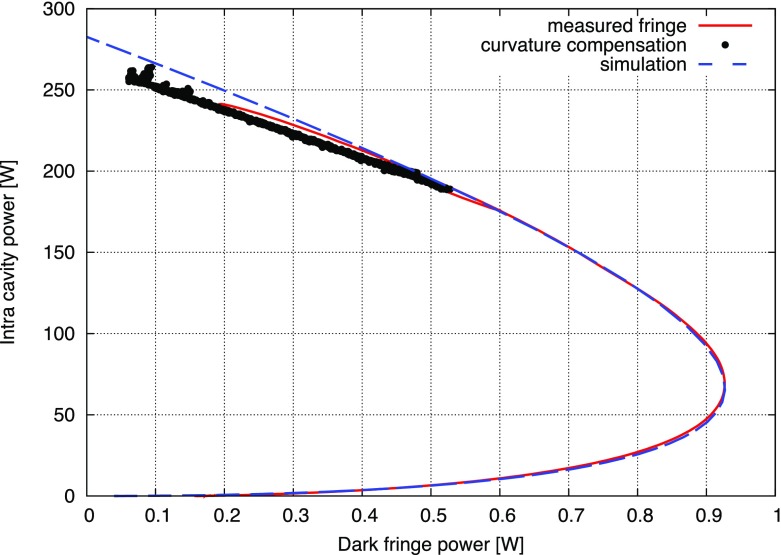
Comparison of measurements and simulations of the circulating power and dark fringe power in GEO 600. The measured fringe (*red trace*) and simulation (*dashed blue trace*) refer to the measured and simulated powers for GEO 600 as the interferometer is moved through the dark fringe with the optimal curvature compensation applied to the East folding mirror (that which best matches the two arms). The curvature compensation (*black markers*) refers to measurements made whilst the interferometer is locked to the dark fringe and the curvature compensation of the East mirror is varied. Image reproduced with permission from Lück et al. (2004), copyright by IOP

To fully diagnose and understand the nature of this problem these measurements were compared with Finesse simulations of GEO 600. In Fig. 106 the power circulating in the power recycling cavity is plotted against the power at the dark fringe, showing both simulation and experimental results. Two different experimental results are shown. The first result has the optimum curvature compensation applied with the powers measured as the interferometer passes through the dark fringe (solid red trace). The second result is the case where the interferometer is locked to the dark fringe and the curvature compensation is varied (black markers). The experimental and simulation results are sufficiently similar to suggest that our understanding of this problem is correct and that the low intra cavity power/high contrast defect is dominated by a differential mode mismatch. The slight differences between the experimental results and the simulation observed at high intra cavity power can be explained by the limits of the model: no astigmatic or higher-order spatial effects were included in this model and hence the model represents a more simplified system than reality. The experiment and simulation are well matched for low intra-cavity power where the effects of the spherical mode mismatch dominate.

This experience at GEO 600 illustrates the need to have well matched arms. This can be in terms of mode matching, as shown here, or in terms of mirror surface distortions and other defects. While low-order aberrations such as misalignment and mode mismatch can be corrected during operation by means of additional control systems, higher-order effects are typically not actively controlled. It is crucial that the impact of higher-order modes is considered during the design of an interferometer to avoid a large buildup of unwanted modes in the detector.

### Advanced LIGO: implications for design and commissioning

The correct modelling of the impact of beam distortions in interferometers is crucial, not only to our understanding of the physics of real interferometers, but because it will have implications for real experiments, in particular during the design and commissioning of detectors. There are many defects in an interferometer which will effect the shape of the resonating beams. In complex advanced interferometers, such as Advanced LIGO, additional systems help control the shape of the beam, mitigating some higher-order mode effects. The main sources of higher-order modes are:

- *Misalignment* Any tilt or lateral shift between the beam axis and a cavity axis, or between the axes of the multiple interdependent cavities in advanced interferometers, will produce higher-order modes, for small misalignments these are dominated by first-order modes. In modern gravitational-wave detectors these effects are carefully controlled using alignment systems to maintain consistent optical axes within the interferometer and avoid a large amount of power in first order modes on the detection photodiode.
- *Mismatch* Second-order modes arise from a mismatch in beam size or wavefront curvature between the cavity eigenmode and incoming beam, or the multiple cavity eigenmodes in complex interferometers. In gravitational wave detectors mismatches are the result of second-order mirror aberrations from the manufacturing process or environmental processes such as thermal lensing. In Advanced interferometers *thermal compensation systems* will be in place to correct the curvature of the arm cavity mirrors, to compensate any thermal lensing and to avoid large mode mismatches.
- *Surface distortions* Higher-order distortions of the beam are generally the result of higher-order mirror distortions on the highly reflective mirror surfaces. These defects can arise during the manufacturing process (so-called *mirror figure error*) or through environmental processes like the thermal distortion of the mirror surfaces. Whilst first and second order distortions of the beam can be corrected it is more difficult to actively correct modes of a higher order. A crucial part of the design process is to determine the tolerances and requirements for the polishing and coating of the interferometer mirrors, to ensure a low higher-order modes content. This is discussed in more detail in Sect. 11.
- *Apertures* Higher-order modes are also generated when the circulating beams encounter the effective aperture caused by the finite size of optical components. The ‘clipping’ of the beam results in a sharp cut-off, equivalent to the addition of high order modes. The design of a well behaved optical setup will ensure the size of the optics, compared to the beam, is sufficiently large such that these higher-order effects are small and we can simple consider the effect of the aperture as a small power loss.

In this section we consider the impact higher-order mode effects have on the final design of an advanced interferometer. The impact of beam distortions are carefully considered during the design process and here we review the choices motivated by beam shape and size for the particular case of Advanced LIGO.

*The input mode cleaner*

Gravitational-wave detectors use an optical cavity, called the *input mode cleaner* (IMC), between the laser and the main interferometer. The purpose of the IMC is to produce a very pure fundamental $$\mathrm
								{TEM}_{00}$$ Gaussian beam for the detector input, filtering out higher-order spatial modes. It is also used as part of the laser frequency stabilisation system, producing a very stable carrier frequency. This is motivated by the desire to avoid injecting light fields into the interferometer which may couple additional noise to the output photodiode. The interferometer is tuned to the operating point of one specific mode, the carrier $$\mathrm
								{TEM}_{00}$$ mode. Any other fields will propagate differently through the interferometer, for example, most higher-order modes do not enter the arm cavities and therefore carry different phase information than the $$\mathrm
								{TEM}_{00}$$ mode.

**Fig. 107:**
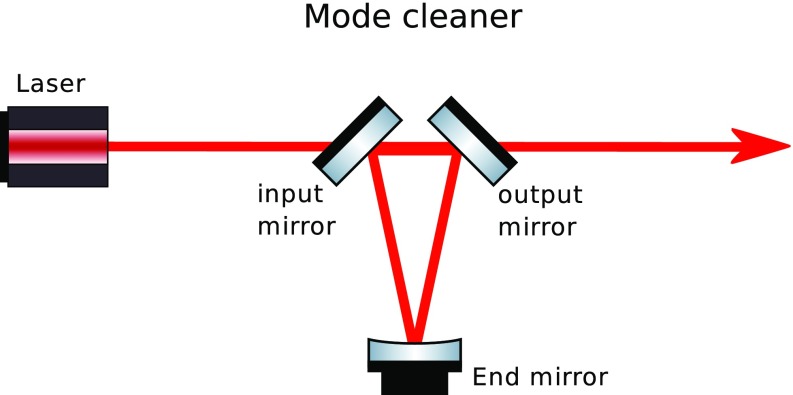
Diagram of a 3-mirror input mode cleaner, similar to that used in advanced interferometers. The cavity is impedance matched to ensure maximum transmission of the carrier

Another requirement of the IMC is to maximise transmission of the fundamental carrier mode, whilst also transmitting the radio frequency control sidebands applied to the beam. For the case of Advanced LIGO the corresponding modulation frequencies are 9 and 45 MHz. The requirement of high transmission for the carrier and certain sidebands sets very specific specifications on the length of the IMC (see Sect. 5.1), whereas the suppression of higher-order spatial modes requires a choice of mirror curvatures which provide a round-trip Gouy phase sufficient to effectively separate the resonance of the spatial modes (see Sect. 10.1). The Advanced LIGO IMC is a 3-mirror impedance matched cavity, as shown in Fig. 107. It consists of two identical flat mirrors (input and output mirrors) and one curved mirror with a very high reflectivity (the end mirror). The final design parameters of the IMC are described in Martin et al. (2013), and Table 5 summarises the key parameters. The free spectral range (FSR) is chosen to allow transmission of the two control sidebands at $$f_1 = 1\times
								\mathrm {FSR}$$ and $$f_2 = 5\times
								\mathrm {FSR}$$.

**Table 5:** Summary of key design parameters of an Advanced LIGO input mode cleaner (Martin et al. 2013)

Parameter	Value
Length	16.473 m
Free spectral range	9, 099, 471 Hz
Input/end mirror $R_C$	${>}10{,}000$ m
Input/end mirror *T*	0.6 %
Input/end mirror *R*	99.4 %
Input/end mirror $\alpha$	$44.59^{\circ }$
Curved mirror $R_C$	$27.24 \pm 0.14$ m
Curved mirror *R*	${>}0.9999$
Curved mirror $\alpha$	$0.82^{\circ }$
Finesse	522

The cavity length, radii of curvature ($R_C$), reflectance (*R*), transmittance (*T*) and angle of incidence ($\alpha$) are all given, as well as derived parameters such as the free spectral range and finesse. The cavity consists of 3 mirrors of which the input and end mirrors are nominally flat

*Recycling cavities*

As discussed in Sects. 10.3 and 10.5, it is important that any beam injected into a cavity is well mode matched to ensure optimum coupling of the laser beam into the cavity. In a Michelson it is important that the two arms are well mode matched to avoid a large amount of power exiting the interferometer through the anti-symmetric port (see Sect. 10.6). Advanced interferometers are highly complex, incorporating a series of cavities within the general Michelson layout. The addition of a recycling mirror at the symmetric port (power recycling) and anti-symmetric port (signal recycling) form recycling cavities between these mirrors and the rest of the interferometer. The parameters of these cavities must be carefully chosen to ensure a good mode match between the eigenmodes of the recycling cavities and the arm cavities. The following discussion of the design of the recycling cavities refers to the most common design based on arguments presented in Arain and Mueller (2008). Note that the Advanced Virgo project has chosen a different design approach (Acernese 2015).

**Fig. 108:**
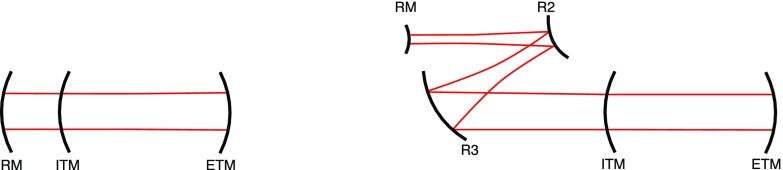
Two examples of a coupled cavity formed between a recycling mirror (RM) and an Advanced LIGO arm cavity, made of the input and end test masses (ITM and ETM). The diagram on the *left* has a single recycling mirror forming a cavity with the ITM. This is illustrative of the setup of Initial LIGO (Adhikari et al. 2006). The diagram on the *right* illustrates a folded recycling cavity, where two additional mirrors in the recycling cavity reshape the beam between the recycling mirror and ITM. This is the setup used in Advanced LIGO (Arain and Mueller 2008). Illustrations are not to scale and in the case of LIGO the distances between the recycling optics is much smaller (of the order 10 m) than the distance between the test masses (4 km)

For the design stage we first assume perfect matching of the arm cavities. We can then consider each recycling cavity acting with the arms as a simple coupled cavity. Two examples of a possible coupled cavity setup are shown in Fig. 108. The eigenmode of the arm cavities is selected to produce large beams at the ITM (5.3 cm) and ETM (6.2 cm) to reduce thermal noise, with slightly smaller beams at the ITM as the thermal noise is lower here (fewer coating layers) and to prevent scattering into the recycling cavities. The curvatures are also carefully selected for a specific Gouy phase to avoid higher-order modes easily ringing up in the arms: $R_C=1934$ m (ITM) and $R_C=2245$ m (ETM). The beam parameter of the arms is therefore a fixed parameter, and the properties of the recycling cavities should be chosen to mode match the recycling cavity to the arms.

The simplest design for the recycling cavities uses a single mirror coupled with the arm cavities, as shown in the left diagram of Fig. 108, where the curvature of the recycling mirror is matched to the wavefront curvature of the arm cavity eigenmode. This was the layout chosen for power recycling in Initial LIGO. In this layout the eigenmode of the arm and recycling cavity can be matched. However, there is another consideration for the design of the recycling cavities: the separation of higher-order mode resonances. This is determined by the Gouy phase accumulated in the recycling cavity (between RM and ITM). In Fig. 109, the Gouy phase of the eigenmode for the Advanced LIGO arms is shown at different positions along the optical axis. The ITM and ETM are both far from the waist but the difference in Gouy phase (155.7$$^{\circ
								}$$, equivalent to $$-24.3^{\circ
								}$$) is far outside the linewidth of the cavity. With a single recycling mirror the only possible positions do not allow for a large change in Gouy phase, as the ITM is already in the far field. In reality there are additional limitations on the position of the recycling mirror, such as the physical location of the vacuum chambers.

In Initial LIGO, this configuration resulted in a power recycling cavity formed in the far field where the higher-order mode resonances were not sufficiently separated: they fell within the linewidth of the cavity. The individual recycling cavity (power recycling mirror and ITM) was only marginally stable in this setup. When operated as a coupled cavity the carrier $$\mathrm
								{TEM}_{00}$$ mode enters the arm cavity, whilst all higher-order modes are directly reflected, meaning the $$\mathrm
								{TEM}_{00}$$ mode acquires 180$$^{\circ
								}$$ of phase on reflection from the arm compared with the higher-order modes. This allowed stable operation of the power recycling cavity for the carrier in Initial LIGO, as in the coupled system the HOMs are effectively anti-resonant in the recycling cavity when the carrier is resonant. However, as observed in LIGO (Adhikari et al. 2006), this configuration is only marginally stable for the control sidebands, which do not enter the arm cavities, resulting in a near-degenerate cavity for the sidebands with all spatial modes near resonance. HOMs of the sidebands are easily excited through misalignment and mode mismatch and it was only the use of thermal compensation systems which allowed the design sensitivity of LIGO to be achieved.

**Fig. 109:**
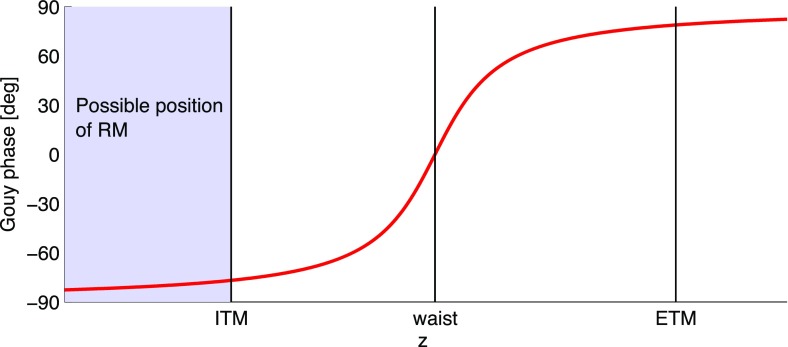
Gouy phase as a function of position on the optical axis for the Advanced LIGO arm cavity eigenmode. A single power/signal recycling mirror (RM) would be placed before the ITM in this representation (Arain and Mueller 2008)

In Advanced LIGO, the issue of unstable recycling cavities becomes more complex due to larger beam sizes, large thermal lensing effects and the addition of signal recycling. Unlike the power recycling cavity the signal recycling cavity coupled with the arm cavities will operate on anti-resonance for the carrier, for resonant sideband extraction. Any HOMs will be nearly resonant in an SRC designed with a single recycling mirror. To avoid these problems in Advanced LIGO an alternative recycling geometry was designed. This is shown in the right diagram of Fig. 108, adding 2 folding mirrors to the recycling cavities to alter the beam parameter and gain significant Gouy phase between the ITM and recycling mirror. The curvatures of these mirrors are carefully chosen to gain this required Gouy phase, whilst maintaining a mode matched system. The design parameters for the power and signal recycling cavities for Advanced LIGO are summarised in “Appendix B”.

Such stable recycling cavities are now installed in Advanced LIGO. Each recycling cavity is characterised by 3 mirrors: the primary mirrors, PRM and SRM, and two additional folding mirrors which shape and direct the beam, PR2/3 and SR2/3. The greatest change in the beam occurs between PR2/3 (and SR2/3) where the beam size increases by around a factor of 10 over a short distance ($${\sim }
								16$$ m). Any small changes in the curvatures of the folding mirrors or the distance between them can lead to substantially larger or smaller beams and degrade the mode matching to the arm cavities. This is illustrated in Fig. 110 where the mode matching between the power recycling cavity, arm cavity and input beam (input mode cleaner eigenmode) is plotted against the distance between PR2 and PR3.^17^ Two sets of results are shown, those for the nominal values of recycling optics, and those for a slight error in the curvature of PR3. The mode matching between the arm and the recycling cavity is relatively insensitive to the PR2–PR3 distance over a 100 mm range, whilst the mode matching between the recycling mode and input beam falls more sharply away from the nominal value. An error in the curvature of the recycling optics can significantly degrade the mode matching, even pushing the recycling cavity to instability (regions of no data). However, the mode matching can be recovered from any such errors by adjusting the distance between the two folding mirrors, R2 and R3.

**Fig. 110:**
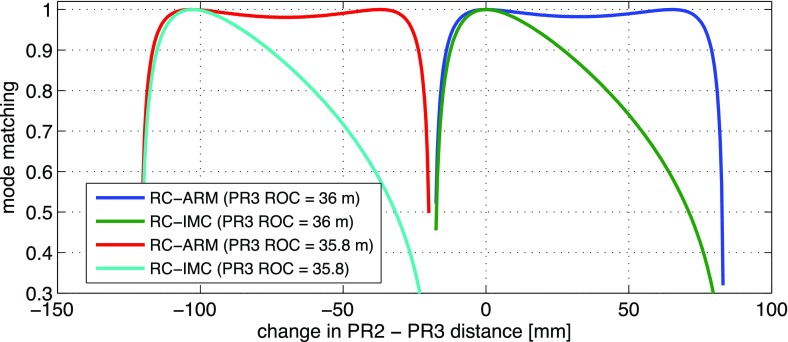
*Plots* showing the mode matching between the recycling cavity eigenmode, the arm cavity eigenmode and the incoming beam (input mode cleaner, IMC, eigenmode) for the Advanced LIGO design. The mode matching is shown for the power recycling cavity as the distance between the two telescope mirrors, PR2 and PR3 (see Fig. 108), is adjusted from the nominal design value. Two sets of results are shown, those for the design curvature of PR3 (36 m) and a small error on this curvature (35.8 m). Adjusting the PR2–PR3 distance recovers the mode matching from errors in the curvatures of the recycling optics (Arain and Mueller 2008)

*Thermal distortions*

**Fig. 111:**
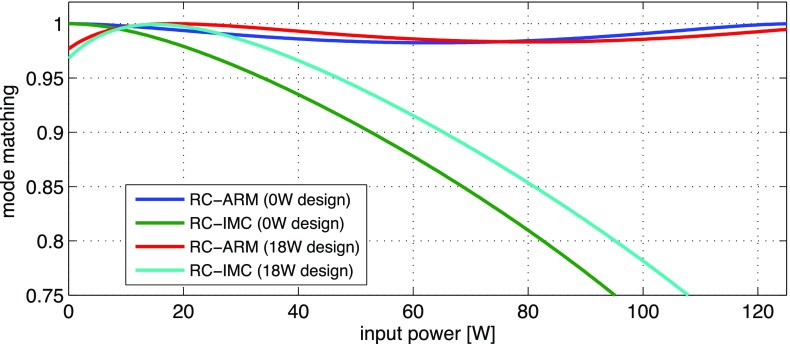
Plots showing mode matching at different input powers between the recycling cavities and the arms (RC-ARM) and recycling cavities and the input mode cleaner (RC-IMC) for the Advanced LIGO design. The mode overlap is calculated considering the thermal lens formed in the input test masses from predicted absorptions in the ITMs. Two different designs are considered, one optimised for mode matching at an input power of 0 W (cold optics) and one optimised for 18 W, the final Advanced LIGO design

The mode matching of the beams between the recycling cavities and arms is complicated by thermal effects, specifically thermal lensing and the change in mirror curvatures. Previously, the need for some thermal compensation was motivated by the behaviour of a single cavity at high power (see Sect. 10.5). For Advanced LIGO the implications for the coupled systems of the arm and recycling cavities were considered during the design phase (Arain et al. 2012). In Fig. 111, the mode matching between the recycling and arm cavities, and the recycling cavities and the input mode is shown, as the interferometer input power is increased. As the thermal lens in the ITM is by far the dominant effect (10.5) this is the only thermal aberration included, modelled as a simple spherical lens. The first two traces in Fig. 111 (0 W design) show the mode matching for the original design of the recycling cavities, where the parameters were chosen to match the cold optics of the arm cavities. A second design (18 W design) is also shown. In this case the mode matching between the recycling cavities, the arm cavities and the input mode was optimised for the expected thermal lensing of 34.5 km^18^ at 18 W input power. The advantages of this design is that it gives a larger range of input power at which the interferometer is well mode matched, without the need for thermal compensation systems.

The plots shown in Fig. 111 show that the mode matching between the recycling cavities and arm cavities is relatively independent of the expected thermal lensing. Whilst the eigenmode of the arm cavity is fixed, the recycling cavity eigenmode is affected by the thermal lens. The recycling eigenmode curvature is fixed at the reflective ITM surface, and the beam size at this point only varies a small amount, maintaining the mode matching between the arm and recycling cavity. However, the effect of the lens on the mode parameters is exaggerated during the large divergence between the recycling mirrors R2 and R3 (see Fig. 108) and this has a large impact on the beam size at the recycling mirror, and hence the mode matching between the input beam and recycling cavity is significantly degraded. As we saw previously for a single cavity, during high power operation the power coupled into the interferometer will be significantly reduced.

In Advanced LIGO *thermal compensation systems* (TCS) will be employed at high power, not only to ensure a large power buildup within the interferometer but to balance the lensing and eigenmodes of the two arms to prevent a high contrast defect (Willems 2009). The first is a ring-heater positioned near the anti-reflective surface of each test mass (Arain et al. 2012). These are used to heat the outer edge of the mirror to produce a curvature in the opposite direction to that from heating by the beam. The ring heater also corrects some of the thermal lens in the ITM substrate. An additional system is required to complete the correction of the thermal lens. This involves a compensation plate, placed in front of the ITMs, made of the same material (fused silica). A heating pattern is projected onto this plate via a CO$_2$ laser. This pattern is designed to heat the compensation plate in such a way as to correct any thermal lensing in the ITM (Brooks et al. 2012).

*The output mode cleaner*

Even with state of the art optics, alignment systems to correct any misalignments and thermal compensation systems to correct for differential mismatches some light at the Michelson anti-symmetric port will be in higher-order modes. There will also be some power in the control sidebands exiting the interferometer, as the dark fringe for the carrier is not the dark fringe for the sidebands due to the applied Schnupp asymmetry (see Sect. 8.13 and “Advanced LIGO configuration” section in “Appendix B”). The only fields which should be present on the detection photodiode are the gravitational wave signal sidebands and the local oscillator field, in the case of Advanced LIGO this is the leaked carrier light for DC readout (see Sect. 6.2). If the power in the higher-order modes and control sidebands is sufficiently low they can be effectively stripped from the beam using an *output mode cleaner* (OMC), an optical cavity between the Michelson interferometer and the main photodiode.

The parameters of the output mode cleaner are carefully chosen to maximise the transmission of the gravitational wave signal and local oscillator field, whilst sufficiently filtering out the unwanted spatial modes and control sidebands. The OMC design for Advanced LIGO is a Fabry–Perot cavity in a 4-mirror bow tie configuration (Arai et al. 2013), consisting of two flat mirrors (the input and output mirrors) and two curved mirrors (high reflectors). To maximise the transmission of the desired fields the cavity is impedance matched between the input mirror and output mirror. Ignoring any losses, the power in an individual field transmitted by an impedance matched cavity is

10.21 $$\begin{aligned}
								P_{\mathrm {trans.}} = \frac{T^2}{1+R^2-2R\cos {(kL_{rt} - \varPsi
								(n+m+1))}} P_0 , \end{aligned}$$

where *T* and *R* are the transmittance and reflectance properties of the input and output coupler, $$P_0$$ is the power in the incoming field, *k* is the wavenumber of the field, $$L_{rt}$$ is the round-trip length of the cavity, $$\varPsi
								$$ is the round-trip Gouy phase and *n* and *m* are the higher-order mode indices of the field. In a lossless cavity all the power in a field is transmitted on resonance. For an Advanced LIGO OMC with realistic losses the transmission of the carrier $$\mathrm
								{TEM}_{00}$$ mode and signal sidebands is expected to be $${\sim }
								98\,\%$$. On anti-resonance the transmitted power can be approximated as

10.22 $$\begin{aligned}
								\mathrm {min}(P_{\mathrm {trans.}}) = \frac{T^2}{1+R^2+2R}P_0
								\approx \frac{T^2}{4} P_0 , \end{aligned}$$

as in the case of a high finesse cavity, $$R\approx
								1$$. In order to avoid transmitting the unwanted fields the length and curvatures of the cavity mirrors are very carefully chosen. The length of the cavity must not be resonant for the 9 MHz and 45 MHz control sidebands. The curvatures of the mirrors are chosen to ensure sufficient Gouy phase to avoid the resonance of any higher-order modes entering the cavity. This requires careful modelling and a knowledge of which higher-order modes are expected to exit the main interferometer. In Arai and Korth (2015) the HOM content is modelled using a power law derived from the spectrum of higher-order modes observed in Enhanced LIGO. This predicts the total power in each order of modes exiting the interferometer as

10.23 $$\begin{aligned}
								P_{\mathrm {HOM}} = 1.8 \times 10^{-3} \times 10^{-\frac{N}{4.8}} ,
								\end{aligned}$$

where *N* is the mode order and $$N \le
								2$$ (1st order modes are reduced via alignment control). Using this power law different mirror curvatures and lengths were modelled to find the optimum design for minimum transmission of the expected undesired fields. This design is presented in Arai et al. (2013) and the key parameters are summarised in Table 6. With a finesse of $${\sim }
								400$$ and an expected round-trip loss of 140 ppm the transmission of undesirable fields is expected to be $$10^{-5}$$ W/W, relative to the power injected into the interferometer. This is equivalent to $${\sim }
								1$$ mW at high power, compared to $${\sim }
								100$$ mW of reference carrier light for DC readout.

**Table 6:** Summary of key design parameters for the Advanced LIGO output mode cleaner (Arai et al. 2013)

Parameter	Value
Length	1.132 m
Free spectral range	264.8 MHz
Input/output mirror $R_C$	$\infty$
High reflectors $R_C$	2.5 m
Input/output mirror *T*	8300 ppm
High reflectors *T*	50 ppm
Finesse	390
Angle of incidence	4$^{\circ }$

The length, mirror radii of curvature ($R_C$) and transmittance (*T*) are given, as well as derived parameters the free spectral range and finesse

### Commissioning

Commissioning describes the process of tuning and improving a gravitational-wave detector after its subsystems have been installed and before the full system is operational. This process typically takes several years because the interferometer couples all the subsystems in a unique and complex way, which cannot be tested in advance. This is particularly important for advanced detectors which employ many cutting edge technologies which, although having been tested in the laboratory and at prototype facilities, have never been implemented together in interferometers of this scale. The efficiency of the commissioning process is crucial to achieving the expected sensitivity and providing an instrument for scientific data taking in a timely manner.

Through the commissioning process we observe effects never seen before, the interferometer will be operated in a new regime, namely a full scale, high power, dual recycled interferometer with arm cavities. In this extremely sensitive configuration previously negligible effects could have a strong impact on interferometer performance. For example, parametric instabilities, where higher-order mode and radiation pressure effects couple together with the potential of ringing up high order sidebands, will likely be a factor in this high powered regime (Braginsky et al. 2001; Evans et al. 2010; Gras et al. 2010). Subsystems of the interferometer also use cutting edge techniques which have yet to be tested within the full framework of our advanced detectors.

During commissioning the interferometers are assembled in increments, building towards the full dual recycled configuration. As the optics are installed many measurements are taken to test the behaviour of various subsystems and finally to test the response and noise budgets of the full interferometer. During this process it is crucial that we have accurate models of the interferometers. These must include possible defects and higher-order mode effects, typically going beyond the more simplified models used in the design phase. For example, in Advanced LIGO measurements of the surfaces of the mirrors were taken prior to installation. This surface data can be used in simulations to model the expected distortion of the beams within the real interferometers. During the commissioning process these models are used to check against experimental measurements. In the case where a measurement is not as expected models are used to investigate the possible causes, adding in more realistic measurements and tuning parameters to recreate the observed behaviour. From such models we can then suggest solutions in the case of underperformance.

The interested reader is directed to the following documents, which give details on specific modelling tasks to support the commissioning of Advanced LIGO, particularly those which are concerned with higher-order mode effects^19^:

- Comparisons of alignment signals calculated for Fabry–Perot cavities using three methods: Finesse, an analytic calculation and the FFT propagation simulation OSCAR (Ballmer et al. 2014).
- Comparisons of the control signals and sideband build up in Advanced LIGO, as modelled in Finesse and Optickle (Bond et al. 2014a; Bond 2014).
- Investigation into the effect of mode-mismatch in the control signals of the Advanced LIGO interferometer (Bond et al. 2014b).
- A dedicated commissioning investigation into power loss at the central beam splitter in Advanced LIGO using Finesse (Bond et al. 2013).
- Finesse simulations of the alignment control signal of the Advanced LIGO input mode cleaner (Kokeyama et al. 2013).

### Finesse examples

#### Higher-order mode resonances

**Fig. 112:**
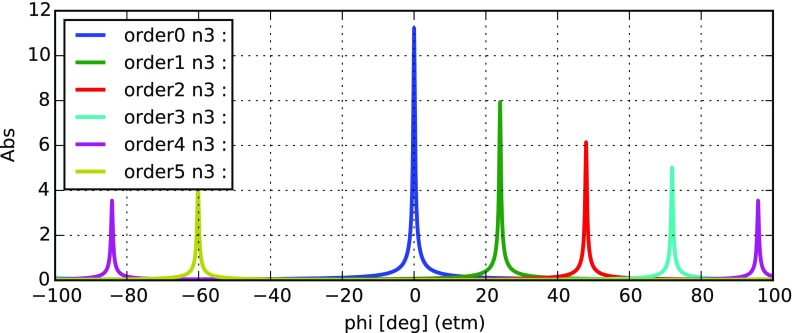
Finesse example: higher-order mode resonances

This example illustrates the different resonance conditions of the higher-order modes in an optical cavity. In the simulation an input beam made up of 6 different order modes is generated using the ‘tem’ commands. This beam is injected into an Advanced LIGO style cavity and the cavity length is tuned. Using amplitude detectors, ‘ad’, the different order modes are individually detected (Fig. 112).

**Finesse input file for ‘Higher-order mode resonances’** 
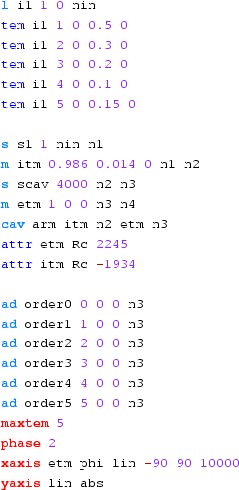


#### Mode cleaner

**Fig. 113:**
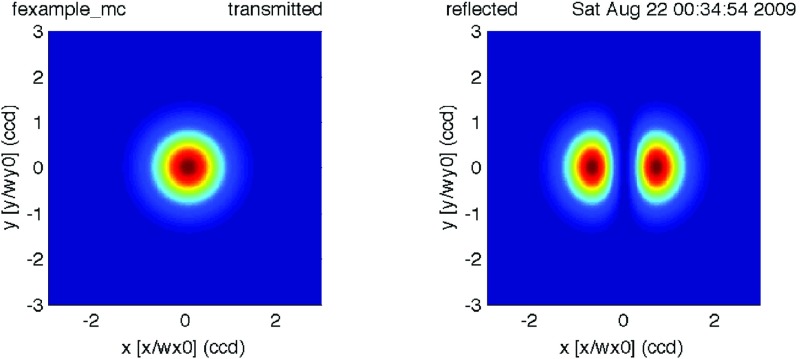
Finesse example: mode cleaner

This example uses the ‘tem’ command to create a laser beam which is a sum of equal parts in $u_{00}$ and $u_{10}$ modes. This beam is passed through a triangular cavity, which acts as a mode cleaner. Being resonant for the $u_{00}$, the cavity transmits this mode and reflects the $u_{10}$ mode as can be seen in the resulting plots (Fig. 113).

**Finesse input file for ‘Mode cleaner’** 
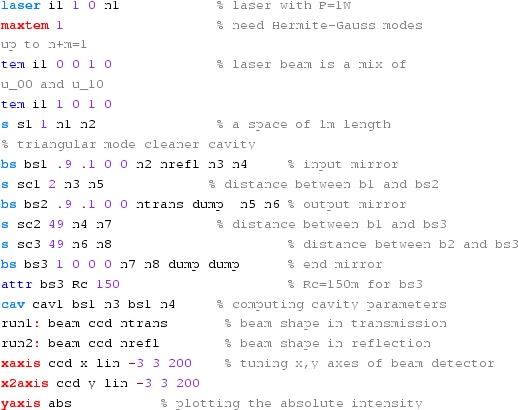


#### Misaligned cavity

**Fig. 114:**
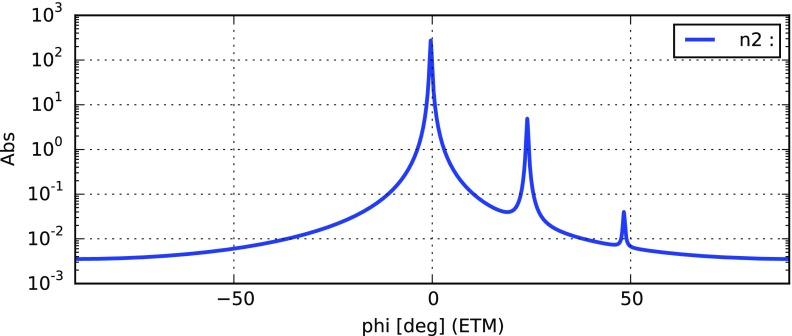
Finesse example: misaligned cavity

In this example a misaligned cavity is scanned and the circulating power is detected. Additional spikes in the cavity scan indicate the higher-order modes (order one and two are visible) created by the misalignment (Fig. 114).

**Finesse input file for ‘Misaligned cavity’** 
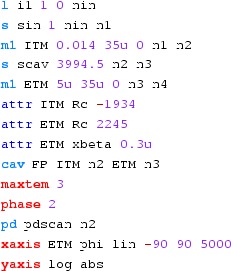


#### Impact of thermal aberrations

**Fig. 115:**
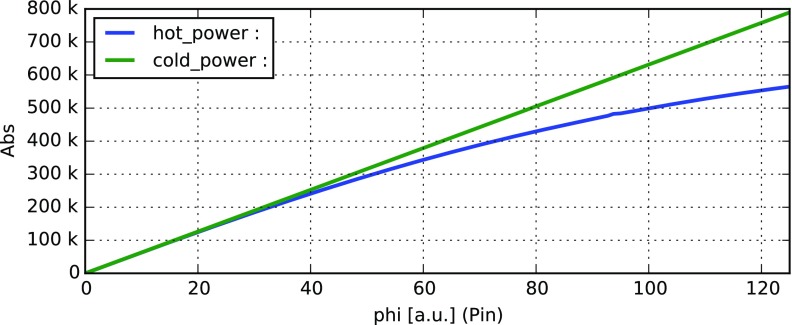
Finesse example: thermal cavity

This example shows the power circulating in an Advanced LIGO style arm cavity versus input laser power when we consider the impact of thermal effects (lensing of the input mirror and change in curvature of the mirror surfaces). The mode mismatches these aberrations cause results in less power coupled into the cavity (Fig. 115).

We assume here that the thermal aberrations scale linearly with power (Vinet 2009). As we tune the incident laser power we also tune the thermal changes in curvature (dRc1 and dRc2). Here we use the change in Rc calculated for an Advanced LIGO cavity operating at high power (125 W) and then scale the Rcs accordingly. The curvatures are combined with the cold state curvatures to give the final state of the cavity mirrors at a given laser power. Similarly for the thermal lens in the ITM we scale the focal length, calculated for high power, with input power.

Finally we scale the power circulating in an individual arm cavity ($Pc) by the gain afforded by the power recycling cavity (45 W/W) and the beam splitter (0.5) to represent the power in an arm of the full power recycled Michelson configuration. We also plot the theoretical linear circulating power, when thermal effects are not considered. Here the 280.7 W is the circulating power in a cavity simulated with no thermal or higher order mode defects.

**Finesse input file for ‘Thermal cavity’** 
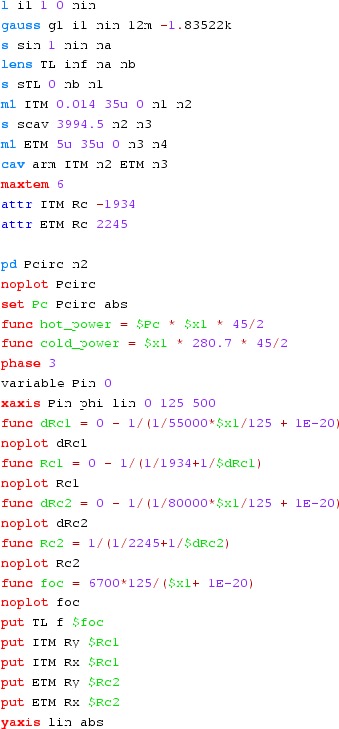


## Scattering into higher-order modes

Spatial variations in the optics that compose a laser interferometer, such as distortions of the mirror surfaces, will change the shape of the circulating beams. Methods for quantifying such optical imperfections and their effects are required during the design of an interferometer and for modelling efforts to characterise the instrument during operation. In particularly, this is crucial during the design phase in order to produce, for example, polishing requirements for the mirror surfaces. At first glance it is not obvious how such optical defects should be characterised and we will show that the nature of the problem determines which approach to use.

Previously we introduced the idea that these distortions can be described as higher-order Gaussian modes and considered the impact of such modes on the interferometer performance. In this section we consider the mechanisms and mathematics of this scattering into higher-order modes, with particular emphasis on this process for mirror surface distortions. We will explore how different types of surface distortions impact the beam shape and quantify which mirror shapes produce which higher order modes. Throughout this section we use measured data from the Advanced LIGO mirrors, kindly provided by GariLynn Billingsley of the LIGO Laboratory (Billingsley 2015).

### Light scattering in interferometers

The term ‘scattering’ in interferometers can refer to several different processes. Most commonly it refers to imperfections of high spatial frequency that scatter light at large angles away from the optical axis, effectively scattering light out of the path of the beam. This is a different problem to scattering into higher order modes, which occurs when the light is scattered back into the path of the beam (i.e., small angle scattering). Light scattered at large angles has the potential to be re-scattered back into the path of the beam by interactions with, for example, the walls of the beam tube. This will couple new noises into the interferometer, from the beam tubes into the circulating light field. Low angle scattering into higher order modes can introduce noise in other ways, as was discussed in Sect. 10. The effects of scattered light and mitigation solutions are an ongoing research topic in the gravitational wave community (Vinet et al. 1997; Yamamoto 2007; Accadia 2010; Vander-Hyde et al. 2015).

The different scattering processes require different methods for efficient, accurate modelling. Whereas low-angle scattering can be modelled using a paraxial approach, either via a description of higher-order modes or using a Fourier propagation model, high-angle scattering is outside the paraxial approximation and can require computationally heavy numerical algorithms for accurate results.

In this review we focus on low angle scattering which manifests itself as changes in the beam shape. Of course low and high angle scattering are not two separate phenomena, and we see the paraxial method fail at scattering angles greater than $${\sim }20^{\circ
								}$$. This region between high and low-angle scattering can be difficult to model, falling between the two regimes. In addition, the finite size of the mirrors in real interferometers prevents the buildup of very high-order modes as these are wider than the mirrors and experience significant larger losses. In this way the finite size of the cavity mirrors can set a limit for high angle scattering.

The two regimes of scattering correspond to some extent to two commonly used categories for describing spatial surface defects:

1. Flatness, describing the overall shape of a mirror and its large scale, low spatial frequency features. These defects impact the shape of the beam within the path, as can be described with higher spatial modes.
2. Roughness, the high spatial frequency distortions of the mirror which scatter light out of the path of the beam.

### Mirror surface defects

**Fig. 116:**
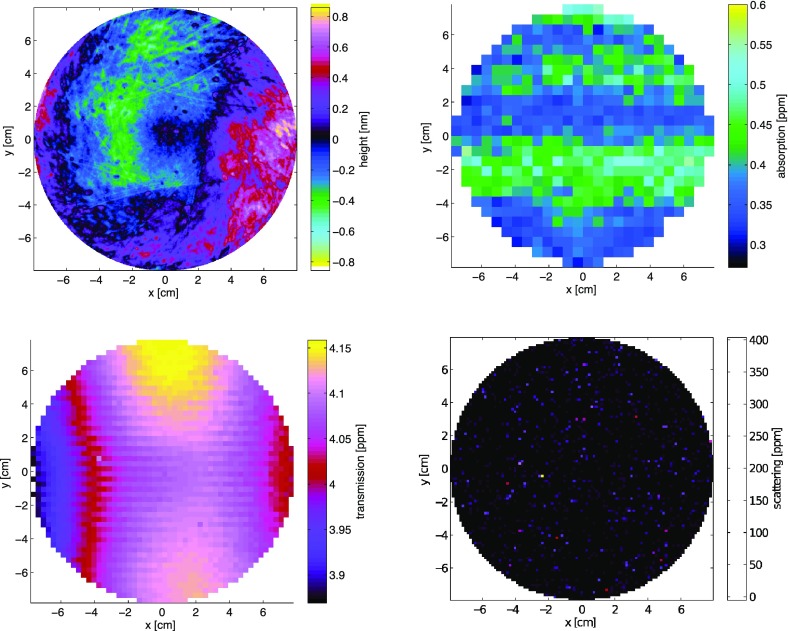
*Maps* showing different properties of an Advanced LIGO mirror, measured across the optic (end test mass ETM08) (Billingsley 2015). *Top left* The surface height of the polished, uncoated substrate of the optic. *Top right* The absorption of the coated mirror at 1064 nm. *Bottom left* Transmission of light through the coated optic at 1064 nm. *Bottom right* Average scatter from the coated mirror surface at 1064 nm. The surface height map was measured by Zygo, with the absorption, transmission and scatter maps provided by the vendor, Laboratoire des Matériaux Avancés (LMA)

Realistic mirrors differ from their ideal form in that their optical and geometric properties are not uniform over the optic. A possible categorisation of mirror imperfections in interferometers are:

- misalignment and curvature mismatch, i.e., a mismatch between the position, orientation and shape of the optics with respect to the laser beam
- non-uniform mirror phase effects, distorted surfaces and substrates will change the phase distribution of a reflected or transmitted beam
- non-uniform amplitude effects: dirty or distorted optics can cause non-uniform absorption and reflection
- apertures created by the finite size of the optics.

The mirrors can be characterised in detail before installation, see for example the presentation (Billingsley and Zhang 2012). Figure 116 shows plots detailing some measured properties from an Advanced LIGO mirror (Billingsley 2015). These *mirror maps* (see Sect. 11.5) can be used in simulations of detectors for a more accurate comparison with experimental results or for the purposes of producing design requirements for the mirrors. In the following we discuss the effects of mirror surface distortions, as these are expected to be the dominant source of spatial beam distortions. Similar methods can be used to model non-uniform absorption or reflection properties.

### Coupling between higher-order modes

Any paraxial beam can be expressed as a sum of Hermite or Laguerre–Gauss modes. An expansion, in terms of Hermite–Gauss modes, of the arbitrary field *u*(*x*, *y*, *z*), can be written as (Siegman 1986):

11.1 $$\begin{aligned}
								u(x,y,z) = \sum _{n,m} k_{nm} u_{nm}(x,y,z),
								\end{aligned}$$

where $k_{nm}$ refer to coefficients which describe the amplitude and phase of each Gaussian mode in the field *u*(*x*, *y*, *z*). The Hermite–Gauss (and Laguerre–Gauss) modes are orthonormal and the coefficients can be calculated from an inner product with the relevant HG (or LG) mode:

11.2 $$\begin{aligned}
								k_{nm} = \int _{-\infty }^{\infty } \int _{-\infty }^{\infty }
								u(x,y,z) \ u_{nm}^*(x,y,z) \, \mathrm {d}x \, \mathrm {d}y.
								\end{aligned}$$

The integral for a generic distortion from an input mode $u_{n'm'}$ to an output mode $u_{nm}$ due to some distortion to the input beam described by the complex function *A*(*x*, *y*) is:

11.3 $$\begin{aligned}
								k_{n,m,n',m'}(q, q', A) = \iint _{-\infty }^{\infty
								}{u_{nm}(x,y;\,q) \, A(x,y)\, u^{*}_{n'm'}(x,y;\,q')} \, \mathrm
								{d}x \, \mathrm {d}y. \end{aligned}$$

In the special case when *A*(*x*, *y*) is exclusively misalignment

11.4 $$\begin{aligned}
								A(x,y) \Rightarrow A(x,y,\gamma _x, \gamma _y) = e^{\mathrm
								{i}\,2kz' (\sin ^2(\gamma _x/2)+\sin ^2(\gamma _y/2))e^{\mathrm
								{i}\,k}}, \end{aligned}$$

the integral can be simplified to the Bayer-Helms coupling equation (9.68) as described in Sect. 9.16. In general, however, the integral cannot be solved analytically. To model realistic mirror surfaces and how they couple higher-order-modes, numerical metrology data is used directly in the coupling coefficient integral. In the generic case *A*(*x*, *y*) represents a complex valued function that interpolates the measured data. For example, for the coupling in reflection from a mirror surface we have

11.5 $$\begin{aligned}
								k^{\mathrm {refl.}}_{nm,n'm'} = \int _{-\infty }^{\infty } \int
								_{-\infty }^{\infty } u_{nm}(x,y,z) \exp {(2\mathrm {i}\,k n_1
								z(x,y))} u^*_{n'm'}(x,y,z) \, \mathrm {d}x \, \mathrm {d}y ,
								\end{aligned}$$

where *z*(*x*, *y*) describes the distorted surface height and $n_1$ is the index of refraction for the incident and reflected fields. Similarly, for transmission through a distorted surface we have

11.6 $$\begin{aligned}
								k^{\mathrm {trans.}}_{nm,n'm'} = \int _{-\infty }^{\infty } \int
								_{-\infty }^{\infty } u_{nm}(x,y,z) \exp {(\mathrm
								{i}\,k(n_2-n_1)z(x,y))} u^*_{n'm'}(x,y,z) \, \mathrm {d}x \, \mathrm
								{d}y , \end{aligned}$$

where $n_1$ is the index of refraction for the incident beam and $n_2$ is the index for the transmitted beam. This process of distorting the beam is refereed to as coupling into other modes, as the action of reflection from a distorted surface creates modes other than those contained in the incoming beam.

In interferometer simulations such as Finesse that use modes to describe the beam shape, a maximum order of the modes included $O_\mathrm{max}$ must be defined for each model. The coupling between all modes with an order less than the maximum order of modes is calculated in reflection and transmission of a distorted optic. This is represented as a coupling coefficient matrix, as described in Sect. 9.16, which computes the transformation of the incident light field as it interacts with the distorted optic. These coupling matrices are inserted into the matrix describing the interferometer behaviour, as described in Sect. 2.3, giving the higher-order mode content at any position within the simulated setup. For well behaved optics, such as those installed in gravitational-wave detectors, we can accurately model realistic distortions of the beam with a finite number of modes, as long as we chose a good Gaussian basis (eigenmode) to work in. The further from the ideal eigenmode the more modes you will require to converge to the correct result. It has been our experience that the best eigenmode is most often that of the optical cavity, as given by the mirror curvatures and positions.

### Simulation methods

Optical simulation tools inherently involve approximations in order to provide meaningful results within practical computation times. The most common approximation when modelling laser interferometers for gravitational-wave detectors is assuming a paraxial beam, allowing the use of a small angle approximation. There are two distinct simulation methods based on the paraxial approximation:

1. Modal decomposition with light fields expressed as linear combinations of Gaussian modes (solutions to the paraxial wave equation).
2. Fast Fourier Transform (FFT) methods where the light fields are represented as finite numerical grids which are propagated through an optical system in the Fourier domain.

The most important aspects of performing simulations with modal models are: (1) to use the correct Gaussian basis for the higher-order mode expansion; and (2) to use enough higher-order modes to recreate distortions of the wavefront. A good choice of Gaussian basis means a small number of modes should be sufficient to reproduce the distortions we expect in gravitational wave interferometers. In this review we make extensive use of the modal simulation Finesse, see “Appendix A”. Other modelling tools for laser interferometers are based on, or are using, Gaussian modes (Evans 2004; Vajente 2013). The FFT method formed the beginning of optical modelling in the gravitational-wave community and has been used extensively since (Vinet et al. 1992; Bochner and Hefetz 2003; Day et al. 2014; Jerome 2008). Both methods contain further approximation, in the addition to assuming paraxial behaviour. In the case of modal models this arises from the finite number of modes. In FFT codes the finite grid size and resolution restricts the accuracy. A balance between accuracy and efficiency often determines the number of modes and grid dimensions used in these simulations. Some powerful tools have been developed which can use modal and FFT based methods internally (Caron et al. 1999; Yamamoto et al. 2015).

**Fig. 117:**
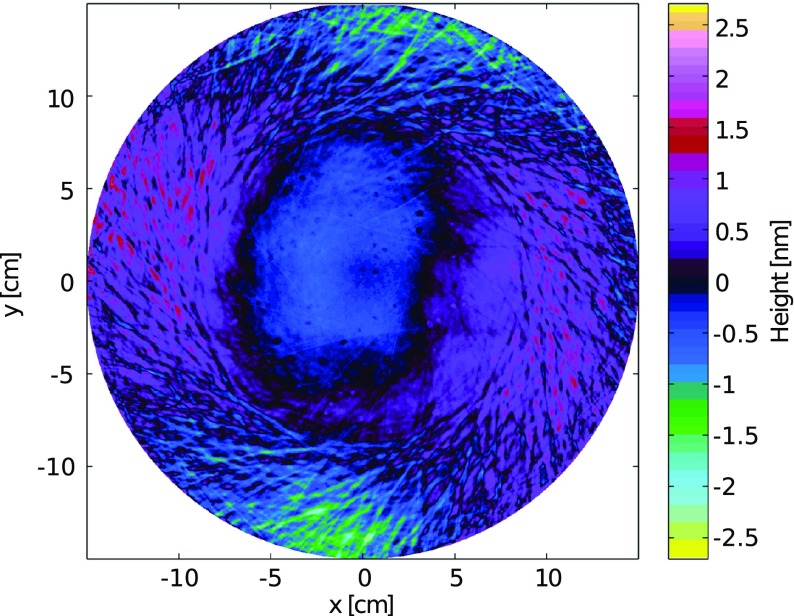
Mirror map describing the measured surface height of an Advanced LIGO end test mass, before coating. The curvature, tilt and average offset of the surface are removed from the data, to clearly show the higher-order distortions of the mirror surface

### Mirror surface maps

In order to analyse the effects of mirror surface distortions we require numerical descriptions of actual mirrors. In this section we discuss several methods for representing mirror surface data, with some methods more suited to use in numerical simulations, whilst others allow an analytic analysis.

A powerful way to implement mirror surface distortions in modal models is by using a numerical grid representing the surface height of the real mirrors, known as *mirror maps*. This is how mirror surface effects are implemented in the interferometer simulation Finesse (Freise 2015; Freise et al. 2004). The surface data is given as a function over the *x*–*y* surface of the optic and can either be measured data from a real mirror or data generated from mathematical functions, for example, describing the expected thermal distortion of a mirror. Mirror map data can be produced for surface height, reflectivity, transmissivity or absorption over the surface of the optic. Unless otherwise noted we use the generic term mirror map referring to surface height. Figure 117 shows an example of a mirror map depicting the surface of an end test mass produced for Advanced LIGO (shown here with any curvature, tilt and offset removed) to illustrate the kinds of distortions of the mirrors we can expect. Note the nanometer scale of the graph, which is typical for mirrors in such high-precision interferometers. We can also see that the central region of the mirror exhibits less surface height variation. Again this is expected, as the requirements on the polishing of the mirror are much more stringent in the centre of the optic where the beam is most intense.

Essentially mirror maps characterise the surfaces by their deviation from a perfect sphere. The terms for any piston, tilt and curvature are then expressed by individual numbers (amplitude, angle and radius of curvature respectively). This raises the problem of how to optimally define these low-order features for a distorted surface. For example, measureing the curvature of a real mirror is done by fitting a spherical function to the measured surface data, minimising the difference between our reference function, the spherical surface $Z_\mathrm{sphere}$, and our data, the mirror map $Z_\mathrm{map}$. This is represented by minimising the function

11.7 $$\begin{aligned}
								f = \int _0^{2\pi } \int _0^R [Z_\mathrm{map} - Z_\mathrm{sphere}]^2
								\, r \, \mathrm {d}r \, \mathrm {d}\theta ,
								\end{aligned}$$

where *R* is the radius over which we are measuring. For a typical distorted surface the result can vary greatly with *R*. We could chose *R* to be the radius of the mirror, taking a measure of the curvature over the whole surface. However, if we consider the part of the mirror over which the Gaussian beams interact we can measure the curvature the beam ‘sees’ more effectively. Therefore, it makes sense to weight our fitting routine using a Gaussian function:

11.8 $$\begin{aligned}
								f = \int _0^{2\pi } \int _0^R W(r,\theta ) [Z_\mathrm{map} -
								Z_\mathrm{sphere}]^2 \, r \, \mathrm {d}r \, \mathrm {d}\theta ,
								\end{aligned}$$

where $$W(r,\theta
								)$$ is the weighting function, in most cases given by the intensity distribution of the fundamental Gaussian beam and *R* is the radius of the mirror. The plots in Fig. 118 show different estimates for the curvature of a mirror surface measured over different regions and using a weighted fitting function. There is a significant difference in curvature depending on the area or weighting used and we must take care to use the correct measurement for accurate models. It is especially important for modal simulations that the correct curvature is measured, as this will determine the cavity eigenmodes, and the basis of our calculations. In most cases working in the cavity eigenmodes ensures efficient simulations: accurate results using the least higher order modes. The offset and tilt can be measured using similar methods, specifying the area or weighting with which to measure the defect.

**Fig. 118:**
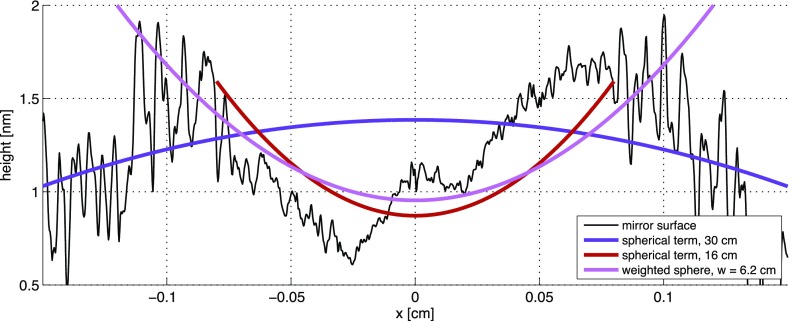
Estimates of mirror curvature over different radii and with different weightings for a distorted mirror surface. Three different estimates for the curvature of the surface are shown, (1) the spherical term over the whole 30 cm; (2) the spherical term over the central 16 cm region; and (3) the Gaussian weighted curvature of the surface, using a weighting beam size of $w=6.2$ cm

### Spectrum of surface distortions

It is desirable to have an analytic description of a mirror surface, not just numeric data, for example, to categorise specific types of distortion. A commonly used method for describing surfaces is to use a spectrum over spatial frequencies or wavelengths. The distortion of a surface along the *x*-axis at a specific spatial frequency, *F*, can be written as:

11.9 $$\begin{aligned}
								Z(x) = A\cos {\left( 2\pi F x + \phi \right) } ,
								\end{aligned}$$

where *A* is the amplitude of the distortion and $$\phi
								$$ is the initial phase of the distortion. For a purely cosine distortion $$\phi
								=0$$ and for purely sine $$\phi =\frac{\pi
								}{2}$$. A generic distortion can be described by a sum of sines and cosines at different frequencies, $$F_n$$:

11.10 $$\begin{aligned}
								Z(x) = \sum _n A_n \cos (2\pi F_n + \phi _n) .
								\end{aligned}$$

The coefficients and phases can be extracted from a discrete Fourier transform of measured surface data *z*(*x*), calculated using a Fast Fourier Transform (FFT) algorithm:

11.11 $$\begin{aligned}
								Z(k) = \sum _{n=0}^{N-1} z(n) \exp {\left( -\frac{\mathrm {i}\,2 \pi
								(k-1)(n-1)}{N}\right) } , \end{aligned}$$

where *N* is the number of elements in *z*, and *n* and *k* are integer indices related to the spatial coordinate *x* and *k* and spatial frequency *F* respectively. This method can be adapted to a 2 dimensional surface, for example, by averaging a 2D Fourier transform into a single 1D amplitude spectrum, similar to the root mean squared (*rms*) for each spatial frequency. In Fig. 119 this analysis is shown for an Advanced LIGO mirror. From such an analysis we can identify what spatial frequencies are present or dominant in the mirror surfaces distortions.

**Fig. 119:**
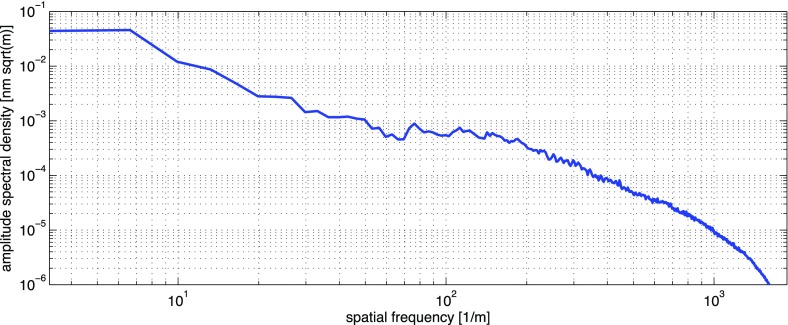
Amplitude spectral density of the different spatial frequencies present in the Advanced LIGO mirror map ETM08, calculated using a 2D FFT and computing a radial average. The offset, tilt and common curvature terms have been removed prior to this analysis

Low spatial frequency distortions correspond to the overall mirror shape, higher spatial frequencies refer to the *roughness* of the mirror. The amplitude of the lower spatial frequencies is significantly higher, as expected. Higher spatial frequencies occur naturally with smaller amplitudes but are also required to be very small in gravitational wave mirrors to reduce wide angle scattering out of the beam path.

### Surface description with Zernike polynomials

A convenient model for describing the overall shape and low spatial frequency distortion of a mirror surface are Zernike polynomials. Zernike polynomials are a complete set of functions which are orthogonal over the unit disc and defined by radial index, *n*, and azimuthal index, *m*, with $$m \le
								n$$. For any index *m* we have

11.12 $$\begin{aligned}
								\begin{aligned} Z_{n}^{+m}(\rho ,\phi )= {}&\cos (m\phi
								)R_{n}^{m}(\rho ) \quad \quad \text{ the } \text{ even } \text{
								polynomial } \\ Z_{n}^{-m}(\rho ,\phi )={}&\sin (m\phi
								)R_{n}^{m}(\rho ) \quad \quad \ \text{ the } \text{ odd } \text{
								polynomial }\\ \end{aligned}
								\end{aligned}$$

with $$\rho
								$$ the normalised radius, $$\phi
								$$ the azimuthal angle and $$R_{n}^{m}(\rho
								)$$ the radial function

11.13 $$\begin{aligned}
								R_{n}^{m}(\rho )= \left\{ \begin{array}{ll} \sum
								_{h=0}^{\frac{1}{2}(n-m)} \frac{(-1)^{h}(n-h)!}{h! \left(
								\frac{1}{2}(n+m)-h\right) ! \left( \frac{1}{2}(n-m)-h\right) !} \rho
								^{n-2h} &{} \quad \text{ for } \text{ even } n-m\\ 0
								&{} \quad \text{ for } \text{ odd } n-m\\ \end{array}
								\right. \ \end{aligned}$$

**Fig. 120:**
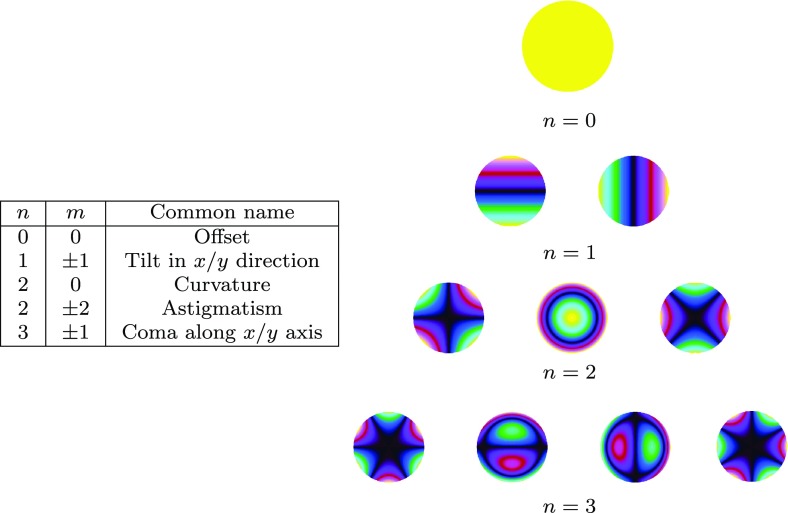
Plots of all the non-zero Zernike polynomials from $n=0$ to $n=3$. They go from odd polynomials with $m=-n$ on the far left to even polynomials with $m=n$ on the far right, in steps of 2. The *colour scale* represents negative surface heights with *greens* and *blues*, zero with *black* and positive surface heights with *reds* and *purples*

This gives $n+1$ non-zero Zernike polynomials for each value of *n* (for $m=0$ the odd polynomial is zero). Some common optical features are described by the low order Zernike polynomials, as shown in Fig. 120. The simplest polynomials represent effects we are familiar with: offset (longitudinal tuning), tilt (misalignment) and curvature (mode mismatch). The higher *n* polynomials represent higher spatial frequencies.

Odd and even Zernike polynomials describe the same shape for given *n* and *m*, with a rotation of $$\frac{90^{\circ
								}}{m}$$ with respect to each other. A combination of the odd and even polynomials result in the same shape rotated by a given angle with an amplitude:

11.14 $$\begin{aligned}
								a_n^m = \sqrt{(a_n^{-m})^2 +(a_n^{+m})^2} .
								\end{aligned}$$

Any surface defined over a disc can be described as a sum of Zernike polynomials, in the same way any beam shape can be described as a sum of Gaussian modes, making these function suitable for the purposes of describing mirror surface distortions. Mirror surface data, $Z_\mathrm{map}$, can be expressed as

11.15 $$\begin{aligned}
								Z_\mathrm{map} = \sum _{n,m} a_n^m Z_n^m ,
								\end{aligned}$$

where $a_n^m$ is the amplitude of the relevant Zernike polynomial in the surface. In the approach taken here this amplitude has the same units as the map data. We can analyse the surface data contained in mirror maps by decomposing the surface into Zernike polynomials, calculating the Zernike coefficients using an inner product and exploiting the orthogonal nature of the polynomials

11.16 $$\begin{aligned}
								\int _A Z_\mathrm{map}\, \left( N_n^m \right) ^2 Z_n^m \mathrm{d} A
								= a_n^m \left( N_n^m\right) ^2 \int _A Z_n^m \, Z_n^m \mathrm{d}A =
								a_n^m . \end{aligned}$$

Here $$N_n^m$$ is a normalisation factor which gives $$\int _A (N_n^m
								Z_n^m) (N_n^m Z_n^m) \mathrm{d}A = 1$$ and has the form

11.17 $$\begin{aligned}
								N_n^m = \sqrt{\frac{2(n+1)}{(1+\delta _{m,0})\pi }} .
								\end{aligned}$$

**Fig. 121:**
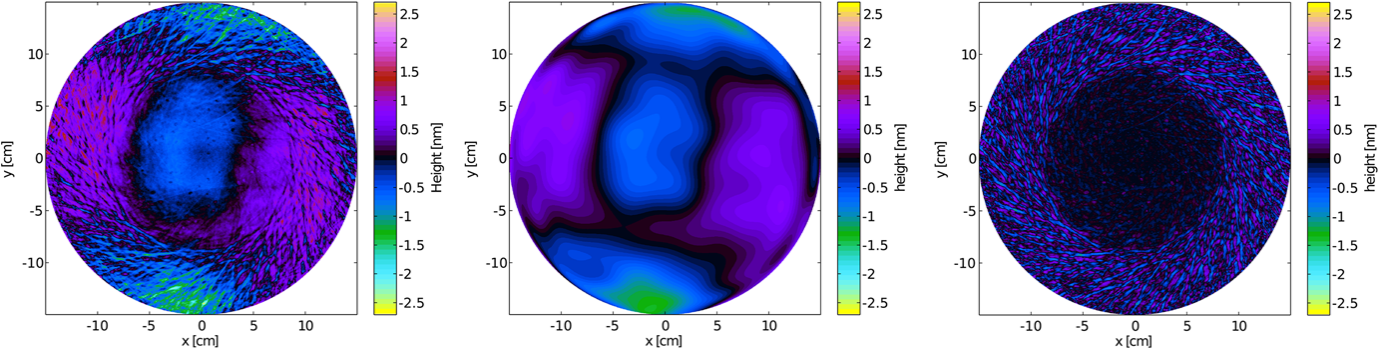
Representations of an Advanced LIGO mirror surface. *Left* Original surface map over 30 cm region, with offset, tilt and curvature (Z$_2^0$) removed. *Center* Map recreated from Zernike polynomials with $n\le 20$, representing the overall shape of the mirror. *Right* Residual surface after the Zernike map is removed, showing the higher spatial frequencies

**Fig. 122:**
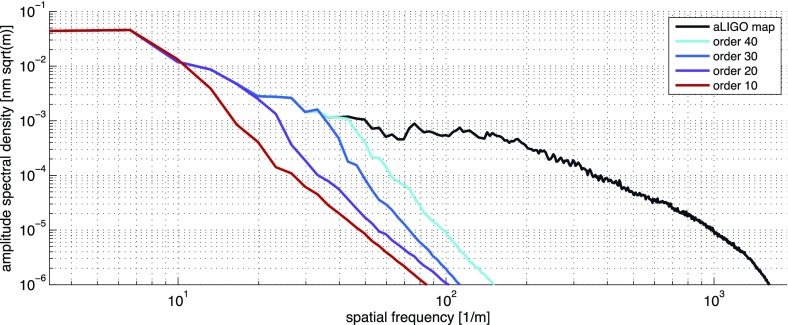
Spectra of spatial frequencies in different ETM08 maps. The spectrum for the original map is shown, as well as those for maps created from Zernike polynomials up to a given order. As more polynomials are added to the model the spectra tend to the original result

Using numerical integration routines real surface data can be represented as a sum of Zernike polynomials. This is illustrated in Fig. 121, where an Advanced LIGO mirror map is recreated using low order Zernike polynomials ($$n\le
								20$$). The overall shape of the Zernike surface looks very similar to the original map, but lacks the high spatial frequencies. These are shown in the residual map which also illustrates the high polishing requirements for the central 16 cm region. Although high spatial frequencies can be represented by Zernike polynomials it is often convenient for mirror surface analysis to consider only the low order Zernike polynomials, with the rest of the mirror description contained in spectra of spatial frequencies. In Fig. 122 the spectrum of an Advanced LIGO mirror map is shown, as well as the spectra for Zernike maps recreated using polynomials up to a given order, illustrating how low order polynomials correspond to low spatial frequencies. Including more polynomials in our model tends towards the original map.

### Mode coupling due to mirror surfaces defects

In Sect. 11.3 the method for calculating coupling coefficients numerically, for a generic surface distortion, was discussed. For design of new laser interferometers we want tools to predict which types of distortions will couple light into which higher-order Gaussian modes. Such a tool would allows us to compute specific requirements for the distortions in optics for future detectors. For example, in Bond et al. (2011) the proposal of a new input laser mode, LG$_{33}$, is analysed in terms of the performance of such a high-order mode with the current mirrors. This involves an analysis of the mirror shapes which will couple between LG$_{33}$ and other modes of the same order, as these modes have the potential to seriously degrade the performance. In such a case an analytic approach to coupling, where the distortions are described by functions such as the Zernike polynomials, is highly desirable.

*Scattering into HOMs*

Firstly we consider coupling from specific spatial frequencies within a mirror surface. Such an approach was also considered by Winkler et al. (1994), for an incident HG$_{00}$ mode. Here we expand on this work to present an analytic approach to scattering of light in the modal picture from an arbitrary incident mode.

In this case the mirror surface is described using spatial frequencies and considering the *x* and *y* spatial components separately. For example, for the *x* distortion we have

11.18 $$\begin{aligned}
								Z(x) = h_0 \cos {\left( \frac{2\pi }{\Lambda }x + \phi \right) } ,
								\end{aligned}$$

where $$h_0$$ is the amplitude of the distortion and $$\Lambda
								$$ is the spatial wavelength. For symmetric distortions (around $$x=0$$) $$\phi =
								0$$, for anti-symmetric $$\phi = \frac{\pi
								}{2}$$. The coupling from a particular spatial wavelength is calculated using the Hermite–Gauss modes, separating the *x* and *y* components. As previously discussed, the coupling between Hermite–Gauss modes can be separated into the *x* and *y* components (see Eq. 9.75)

11.19 $$\begin{aligned}
								k_{nmn'm'} = k_{nn'}k_{mm'}.
								\end{aligned}$$

For the coupling from mode *n* to $n'$ we have

11.20 $$\begin{aligned}
								k_{n,n'} = \int _{-\infty }^{+\infty } U_n \exp {(2\mathrm {i}\,k Z)
								} U_{n'}^* \, \mathrm {d}x .
								\end{aligned}$$

where $U_n$ is the *x* component of a Hermite–Gauss mode in the incident beam and $U_{n'}$ is the *x* component of a mode in the reflected beam. The product of the two modes is

11.21 $$\begin{aligned}
								U_n U_{n'}^* = \frac{1}{w} \sqrt{\frac{2}{\pi }} \frac{\exp
								{(\mathrm {i}\,(n-n') \varPsi )}}{\sqrt{2^{n+n'} n! n'!}} H_n\left(
								\frac{\sqrt{2}x}{w}\right) H_{n'}\left( \frac{\sqrt{2}x}{w}\right)
								\exp {\left( -\frac{2x^2}{w^2}\right) } .
								\end{aligned}$$

Assuming the distortion of the surface is small compared to the laser wavelength, a valid assumption when considering the mirrors of gravitational-wave detectors, we use the approximation

11.22 $$\begin{aligned}
								\exp {(2 \mathrm {i}\,k Z)} \approx 1 + 2\mathrm {i}\,kZ .
								\end{aligned}$$

The coupling equation can be furthered simplified due to the orthogonal nature of the Hermite–Gauss modes.

11.23 $$\begin{aligned}
								\begin{aligned} k_{n,n'} {}&= \int _{-\infty }^{\infty } U_n
								U_{n'}^* \, \mathrm {d}x + 2\mathrm {i}\,k \int _{-\infty }^{\infty
								} Z U_n U_{n'}^* \, \mathrm {d}x \\ {}&= \delta _{n,n'} +
								2\mathrm {i}\,k \int _{-\infty }^{\infty } Z U_n U_{n'}^* \, \mathrm
								{d}x, \end{aligned} \end{aligned}$$

where $$\delta
								_{n,n'}$$ is the Kronecker delta, equal to 1 for $$n=n'$$ and 0 otherwise. This represents the coupling back into the incident mode. Expanding the equation and implementing a change of variable we have

11.24 $$\begin{aligned}
								k_{n,n'}= & {} \delta _{n,n'} + C \int _{-\infty }^{\infty }
								H_n(v) H_{n'}(v) \exp {(-v^2)} \cos {\left( \frac{\sqrt{2}\pi
								w}{\Lambda }v + \phi \right) } \, \mathrm {d} v \nonumber \\=
								& {} \delta _{n,n'} + C \cos {(\phi )} \int _{-\infty
								}^{\infty } H_n(v) H_{n'}(v) \exp {(-v^2)} \cos {\left(
								\frac{\sqrt{2}\pi w}{\Lambda }v\right) } \, \mathrm {d}v \nonumber
								\\&- \,C \sin {(\phi )} \int _{-\infty }^{\infty } H_n(v)
								H_{n'}(v) \exp {(-v^2)} \sin {\left( \frac{\sqrt{2}\pi w}{\Lambda
								}v\right) } \, \mathrm {d}v,
								\end{aligned}$$

where

11.25 $$\begin{aligned}
								\begin{array}{ccc} C = \frac{2\mathrm {i}\,k h_0 }{\sqrt{\pi }}
								\frac{\exp {(\mathrm {i}\,(n-n')\varPsi
								)}}{\sqrt{2^{n+n'}n!n'!}}&\text{ and }&v =
								\frac{\sqrt{2}x}{w} . \end{array}
								\end{aligned}$$

These two integrals can be solved using the identities (Gradshteyn and Ryzhik 1994)

11.26 $$\begin{aligned}
								\int _0^{\infty } e^{-x^2} \sin {(bx)} H_p(x) H_{p+2m+1}(x) \,
								\mathrm {d}x= & {} 2^{p-1} (-1)^m \sqrt{\pi } \ p! b^{2m+1}
								\nonumber \\&\exp {\left( -\frac{b^2}{4}\right) }
								L_p^{2m+1}\left( \frac{b^2}{2}\right) \nonumber \\ \int _0^{\infty }
								e^{-x^2} \cos {(bx)} H_p(x) H_{p+2m}(x) \, \mathrm {d}x= &
								{} 2^{p-1}(-1)^m\sqrt{\pi } \ p! b^{2m} \nonumber \\&\exp
								{\left( -\frac{b^2}{4}\right) } L_p^{2m}\left( \frac{b^2}{2}\right)
								.\qquad \end{aligned}$$

for $$b>0$$, where *L*(*x*) refer to the Laguerre polynomials. In our case $$b =
								\frac{\sqrt{2}\pi w}{\Lambda }$$. The first integral refers to coupling from asymmetric distortions (sine terms) where $$n-n'$$ is odd and the second refers to coupling from symmetric distortions (cosine terms) where $$n-n'$$ is even. The solutions to these equations look very similar to the amplitude of the Laguerre–Gauss modes:

11.27 $$\begin{aligned}
								|U_{p,l}| = \frac{1}{W} \sqrt{\frac{2p!}{\pi (|l|+p)!}} \exp {\left(
								-\frac{r^2}{W^2}\right) } \left( \frac{\sqrt{2}r}{W}\right) ^{|l|}
								\left| L_p^{|l|}\left( \frac{2r^2}{W^2}\right) \right| .
								\end{aligned}$$

Note that *r* and *W* are not the radial coordinate and beam spot size as in the definition of an LG mode, but related to the ratio of the beam size to the spatial wavelength, $$\frac{w}{\Lambda
								}$$. For a complete solution with the correct phase the sign of the Laguerre-polynomial should be included, as this disappears when taking the amplitude of the LG mode. Using these identities we have

11.28 $$\begin{aligned}
								\begin{aligned} k_{n,n'} {}&= \delta _{n,n'} + C 2^p \pi W
								\sqrt{\frac{p!(|l|+p)!}{2}} (\sqrt{2})^{|l|} \text{ sign }\left(
								L_p^{|l|}(\pi ^2 r^2)\right) \left| U_{p,l}\right| \\&\quad
								\ \ [\cos {(\phi )} \cos {\left( |l|\tfrac{\pi }{2}\right) } - \sin
								{(\phi )}\sin {\left( |l|\tfrac{\pi }{2}\right) }] \\ {}&=
								\delta _{n,n'} + C 2^p \pi W \sqrt{\frac{p!(|l|+p)!}{2}}
								(\sqrt{2})^{|l|} \text{ sign }\left( L_p^{|l|}(\pi ^2 r^2)\right)
								\left| U_{p,l}\right| \cos {\left( \phi + |l|\tfrac{\pi }{2}\right)
								} , \end{aligned} \end{aligned}$$

where

11.29 $$\begin{aligned}
								\begin{array}{ccc} p = \min {(n,n')}&l =
								n-n'&\frac{r}{W} = \frac{\pi }{\sqrt{2}}\frac{w}{\Lambda } .
								\end{array} \end{aligned}$$

The factors $$\sin
								{(|l|\frac{\pi }{2})}$$ and $$\cos
								{(|l|\frac{\pi }{2})}$$ come from the combination of factors $$(-1)^{|l|/2}$$ and $$(-1)^{(|l|-1)/2}$$ with the fact that the integral including the sine term is 0 for even $$n-n'$$ and the integral including the cosine term is 0 for odd $$n-n'$$. For simplicity we set $$W=\frac{\sqrt{2}}{\pi }$$, which gives $$r=\frac{w}{\Lambda }$$, the ratio of the beam spot size to the wavelength of the spatial distortion. Finally substituting in the values for *C* and using $$p+|l|=\max
								{(n,n')}$$ and $$n+n'=2p+|l|$$ the equation becomes

11.30 $$\begin{aligned}
								k^1_{n,n'} = \delta _{n,n'} + \text{ sign }\left( L_p^{|l|}(\pi ^2
								r^2)\right) \frac{2 \mathrm {i}\,k \ h_0}{\sqrt{\pi }} \exp
								{(il\varPsi )} |U_{p,l}(W=\tfrac{\sqrt{2}}{\pi })| \cos {\left( \phi
								+ |l|\tfrac{\pi }{2}\right) } ,
								\end{aligned}$$

**Fig. 123:**
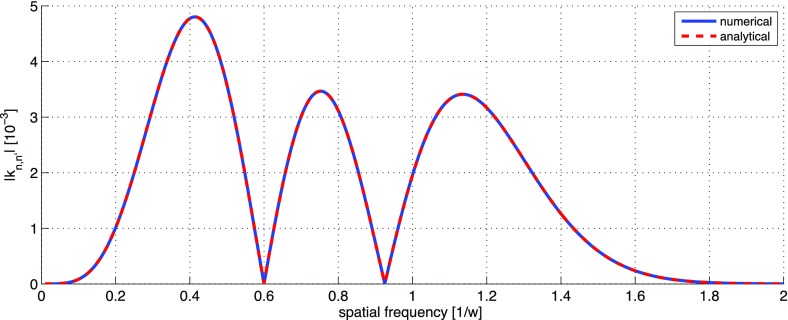
Comparison of the first order coupling approximation (analytical) with the numerical solution of the coupling between the *x* components of the Hermite–Gauss modes, $U_2$ and $U_6$

The coupling between Hermite–Gauss modes of different orders is well expressed by this first order approximation, where the coupling is described with Laguerre–Gauss modes of order $n+n'$. This is illustrated in Fig. 123 where the first order analytical coupling is compared with the numerical solution of the coupling integral for $k_{2,6}$ over a range of spatial frequencies.

For coupling back into the same order ($n=n'$) we require up to second order, derived using the same method as described above. We have

11.31 $$\begin{aligned}
								k_{n,n'} \approx k^1_{n,n'} - k^2h_0^2\delta _{n,n'} -\text{ sign
								}\left( L_p^{|l|}( 4\pi ^2r^2)\right) \frac{k^2h_0^2}{\sqrt{\pi }}
								|U_{p,l}(W=\tfrac{1}{\sqrt{2}\pi })| \cos {\left( 2\phi +
								|l|\tfrac{\pi }{2}\right) } ,
								\end{aligned}$$

where the second order corrections are also described by Laguerre–Gauss modes of order $n+n'$ but with a beam spot parameter half the size of the that of the first order coupling.

**Fig. 124:**
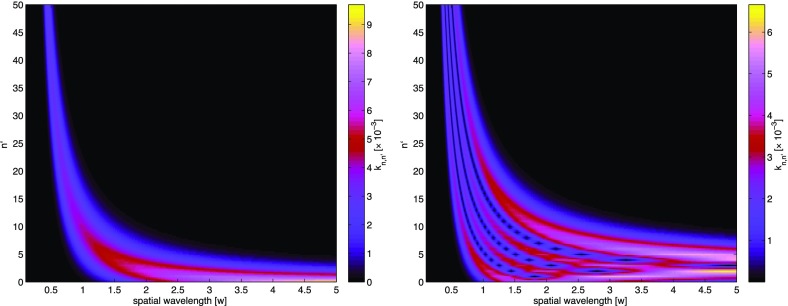
The scattering into higher order modes across a range of spatial distortions (frequencies) for an incident $U_0$ mode (*left*) and an incident $U_3$ mode (*right*)

Figure 124 illustrates the scattering into a range of higher order modes for different spatial frequency mirror distortions. Two examples are given, an incident mode with $n=0$ and an incident mode with $n=3$. For low frequency spatial distortions the coupling occurs mostly into low orders. For higher spatial frequencies, where the wavelength of the spatial distortion is smaller than the beam spot size, coupling occurs into a vast number of higher order modes. In practice the amplitude of spatial distortions is not constant across the spectrum of spatial wavelengths, as is illustrated here ($h_0=1$ nm), but decreases with spatial frequency. In realistic simulations and in experiments the low order modes dominate, so much so that we can model gravitational wave interferometers well with a finite number of modes.

Typically individual $$k_{n.n'}$$ (where $$n\ne
								n'$$) are of the order $$10^{-3}$$ for 1 nm distortions. For coupling back into the same mode $$k_{n,n}\approx
								1$$ for small distortions. We would therefore expect coupling from HG$$_{n,m}$$ to HG$$_{n,m'}$$ or HG$$_{n',m}$$ would be significantly larger than coupling where both indices change, as these are of the order $$10^{-3}$$ rather than $$10^{-6}$$.

The analytical coupling approximation described here is discussed in detail in Bond (2014). This provides a quick analytical tool to predict the modes produced on interaction with distorted mirrors and can be used to provide mirror surface requirements and during the commissioning stage to predict the modal content of the resonating laser beams.

*Zernike coupling*

Common analysis using spatial frequencies involves taking a statistical approach: performing numerous simulations using randomly generated realisations of mirror surfaces to determine the higher order mode behaviour for a mirror conforming to a particular spectrum of spatial frequencies. Such an approach for an LG$_{33}$ mode investigation is detailed in Hong et al. (2011). Here we present an analytic approach which aims to identify the exact shapes which couple between different modes.

An analysis of mirror surface distortions in terms of Zernike polynomials is complementary to the approach of describing the shape of the beam in terms of Gaussian modes. Both methods deal with the overall shape of the beam/mirror by expressing them as sums of orthonormal functions. Now we have an analytic representation of mirror surface distortions we can try and formulate a relation between particular mirror shapes and their impact on the beam. The coupling on reflection from a mirror described by an individual Zernike polynomial, $Z_n^m$, is

11.32 $$\begin{aligned}
								k^{n,m}_{p,l,p',l'}=\int _{A}U_{p,l}\exp {\left( 2\mathrm
								{i}\,kZ_{n}^{m}\right) }U^{*}_{p',l'} \mathrm{d}A ,
								\end{aligned}$$

where $U_{p,l}$ is a mode in the incident beam and $U_{p',l'}$ is a mode in the reflected beam. The Laguerre–Gauss modes are most suited for this analysis as they, like the Zernike polynomials, are naturally described in cylindrical coordinates. Assuming the distortions are small enough that the beam parameter remains unchanged, the product of the two LG fields is

11.33 $$\begin{aligned}
								\begin{aligned} U_{p,l} U^{*}_{p',l'} =
								{}&\frac{1}{w^{2}}\frac{2}{\pi
								}\sqrt{\frac{p!p'!}{(|l|+p)!(|l'|+p')!}} \exp {\left( \mathrm
								{i}\,\left( 2p+|l|-2p'-|l'|\right) \varPsi \right) } \\
								{}&\times \left( \frac{\sqrt{2}r}{w}\right) ^{|l|+|l'|}
								L^{|l|}_{p}\left( \frac{2r^{2}}{w^{2}}\right) L^{|l'|}_{p'}\left(
								\frac{2r^{2}}{w^{2}}\right) \exp {\left(
								-\frac{2r^{2}}{w^{2}}\right) } \exp {\left( \mathrm {i}\,\phi \left(
								l-l'\right) \right) } . \\ \end{aligned}
								\end{aligned}$$

To simplify the integral we can simplify the expansion of $$\exp {(2\mathrm
								{i}\,kZ)}$$. Assuming 2*kZ* is small we can use the approximation

11.34 $$\begin{aligned}
								\exp {(2\mathrm {i}\,kZ)} \approx 1+2\mathrm {i}\,kZ .
								\end{aligned}$$

This is a valid approximation for advanced gravitational wave interferometers, where the scale of distortions is not expected to exceed the order of 1 nm. $$Z=10$$ nm and a wavelength of 1064 nm gives $$2kZ\approx
								0.1$$. Making this approximation the coupling coefficients are simplified

11.35 $$\begin{aligned}
								\begin{aligned} k^{n,m}_{p,l,p',l'} {}&= \int
								_{A}U_{p,l}U^{*}_{p',l'}(1+2\mathrm {i}\,kZ_{n}^{m}) \, \mathrm
								{d}A\\ {}&= \delta _{p,p'} \delta _{l,l'} + \int _{0}^{2\pi
								} \int _{0}^{R} U_{p,l}U^*_{p',l'}(2\mathrm {i}\,kZ_n^m) \, r \,
								\mathrm {d}r \, \mathrm {d}\phi , \end{aligned}
								\end{aligned}$$

where $$\delta
								_{p,p'}\delta _{l,l'}$$ refers to coupling back into the same mode. The integral to calculate is over the surface with $$r\rightarrow
								\infty $$, as Laguerre–Gauss modes are orthogonal over this range. But since the integrand is proportional to the Zernike polynomial, *S* becomes the Zernike surface as $$Z_{n}^{m}(\frac{r}{R} > 1)=
								0$$, with *R* the Zernike radius.

Both Zernike polynomials and Laguerre–Gauss modes can be separated into there angular and radial parts. The angular integrand is

11.36 $$\begin{aligned}
								\exp {(\mathrm {i}\,\phi (l-l'))}\times \left\{ \begin{aligned} \cos
								{(m\phi )}&\quad \text{ for } \text{ even } Z_n^m \\ \sin
								{(m\phi )}&\quad \text{ for } \text{ odd } Z_n^m
								\end{aligned} \right. \end{aligned}$$

Considering the even Zernike polynomial the angular integrant becomes

11.37 $$\begin{aligned}
								I_{\phi } = \int _{0}^{2\pi }e^{\mathrm {i}\,\phi (l-l')}
								\frac{e^{\mathrm {i}\,m\phi }+e^{-\mathrm {i}\,m\phi }}{2} d\phi =
								\left[ \frac{e^{\mathrm {i}\,\phi (l-l'+m)}}{2\mathrm
								{i}\,(l-l'+m)}+\frac{e^{\mathrm {i}\,\phi (l-l'-m)}}{2\mathrm
								{i}\,(l-l'-m)} \right] ^{2\pi }_{0} .
								\end{aligned}$$

As $$e^{\mathrm
								{i}\,0}=e^{\mathrm {i}\,N\times 2\pi }=1$$, for integer *N*, the integral is equal to 0. The only combination of Zernike polynomials and Laguerre–Gauss modes to give a non-zero result occurs when one of the exponentials disappears before the integration takes place. This occurs for $$l-l'+m=0$$ or $$l-l'-m=0$$. These same conditions also give the only non-zero results for the odd Zernike polynomials. This forms a *coupling condition* between the azimuthal indices of the shape of the mirror, the Zernike polynomial (*m*), and the LG modes such a mirror couples between (*l*/$$l'$$). This is summarised as

11.38 $$\begin{aligned}
								m=|l-l'| . \end{aligned}$$

Unless this condition is satisfied the coupling between modes *l* and *l*’ is 0, to first order. This condition allows quick identification of the modes which are created from certain mirror shapes. It also agrees with previous work on misalignment and mode-mismatch. For example, the Zernike polynomial Z$$_1^1$$ corresponds to misalignment and couples from the fundamental mode into LG$$_{0,\pm
								1}$$, the order 1 Laguerre–Gauss modes. Similarly the curvature polynomial, Z$$_{2}^{0}$$, couples from LG$$_{0,0}$$ to the order 2 mode LG$$_{0,1}$$.

Using this condition we can integrate with respect to $$\phi
								$$:

11.39 $$\begin{aligned}
								I_{\phi } =\left\{ \begin{array}{ll} 0 &{}\quad m \ne |l-l'|
								\\ \pi &{}\quad \text{ for } \text{ even } Z_n^m \\ \pm
								\mathrm {i}\,\pi &{}\quad \text{ for } \text{ odd } Z_n^m \\
								2\pi &{}\quad m = |l-l'| = 0 \end{array} \right.
								\end{aligned}$$

The next step is to solve the radial integration. Making the variable substitution $$x =
								\frac{2r^2}{w^2}$$ the coefficient becomes

11.40 $$\begin{aligned}
								k^{n,m}_{p,l,p',l'}= & {} \delta _{p,p'} \delta _{l,l'} +
								\mathrm {i}\,k \frac{I_{\phi }}{\pi }
								\sqrt{\frac{p!p'!}{(|l|+p)!(|l'|+p')!}} \exp {(i \varDelta o \varPsi
								)} \nonumber \\&\times \int _0^X x^{\frac{|l|+|l'|}{2}}
								L_p^{|l|}(x) L_{p'}^{|l'|}(x) \ \exp {(-x)} Z_n^m \left(
								\sqrt{\frac{x}{2}}w \right) \, \mathrm {d}x ,\quad
								\end{aligned}$$

with $$X =
								\frac{2R^2}{w^2}$$ and $$\varDelta o = 2p
								+ |l| - 2p' - |l'|$$, the difference in order between the incident and reflected modes. The integrand is in the form of *f*(*x*)*g*(*x*), where *f*(*x*) is a polynomial of *x* whose order depends on the mode and Zernike indices, and $$g(x)=\exp
								(-x)$$. This integration is solved using the incomplete gamma function, $$\gamma
								(n,x)=\int _{0}^{x} t^{n-1}e^{-t} \, \mathrm
								{d}t$$ (Gradshteyn and Ryzhik 1994), which for integer $$n = 1, 2, \ldots
								$$ is

11.41 $$\begin{aligned}
								\gamma (n,x) = (n-1)! \left[ 1 - e^{-x} \sum
								_{m=0}^{n-1}\frac{x^m}{m!}\right]
								\end{aligned}$$

Substituting in this solution to the integral we have the final solution to this coupling approximation as

11.42 $$\begin{aligned}
								k_{p,l,p',l'}^{n,m}= & {} \delta _{p,p'} \delta _{l,l'} +
								A_n^m \mathrm {i}\,k \frac{I_{\phi }}{\pi }
								\sqrt{p!p'!(p+|l|)!(p'+|l'|)!}\nonumber \\&\times \sum
								_{i=0}^{p} \sum _{j=0}^{p'} \sum _{h=0}^{\frac{1}{2}(n-m)}
								\frac{(-1)^{i+j+h}}{(p-i)!(p'-j)!(|l|+i)!(|l'|+j)! i! j!}
								\frac{1}{X^{\frac{1}{2}(n-2h)}} \nonumber \\&\times
								\frac{(n-h)!}{\left( \frac{1}{2}(n+m)-h\right) ! \left(
								\frac{1}{2}(n-m)-h\right) ! h!}\nonumber \\&\times \, \gamma
								(i+j-h+\frac{1}{2}(|l|+|l'|+n)+1,X).
								\end{aligned}$$

It is worth noting that the first order direct coupling described here is proportional to the amplitude of the Zernike polynomial, $A_n^m$.

As with the Winkler scattering approximation detailed above we require up to second order in the exponential expansion to accurately calculate the coupling back into the incident mode. We have:

11.43 $$\begin{aligned}
								k_{p,l,p',l'}^{n,m} {}= & {} \int _A u_{p,l} \exp {(2\mathrm
								{i}\,k Z_n^m)} u^*_{p',l'} \, \mathrm {d}A \nonumber \\\approx
								& {} \delta _{p,p'} \delta _{l,l'} + k^{n,m,1}_{p,l,p',l'} +
								k^{n,m,2}_{p,l,p',l'}, \end{aligned}$$

where $k^{n,m,1}_{p,l,p',l'}$ is the first order coupling as given by Eq. (11.42) and $k^{n,m,2}_{p,l,p',l'}$ is the second order coupling, given by:

11.44 $$\begin{aligned}
								k^{n,m,2}_{p,l,p',l'} = \int _{A} u_{p,l} \ u^*_{p',l'}
								(-2k^2(Z_n^m)^2) \, \mathrm {d}A .
								\end{aligned}$$

As with the first order coupling we can split the integration into the radial and angular parts. The angular integration is:

11.45 $$\begin{aligned}
								I_{\phi } = \left\{ \begin{array}{ll} \int _0^{2\pi } \cos ^2{(m\phi
								)} \exp {(\mathrm {i}\,\phi (l-l'))} \, \mathrm {d}\phi &{}
								\quad \text{ for } \text{ even } Z_n^m \\ \int _0^{2\pi } \sin
								^2{(m\phi )} \exp {(\mathrm {i}\,\phi (l-l'))} \, \mathrm {d}\phi
								&{} \quad \text{ for } \text{ odd } Z_n^m \\ \end{array}
								\right. \end{aligned}$$

Taking the even Zernike polynomial we have:

11.46 $$\begin{aligned}&\int _0^{2\pi }
								\frac{1}{4}\left( e^{\mathrm {i}\,m\phi } + e^{-\mathrm {i}\,m\phi
								}\right) ^2 e^{\mathrm {i}\,\phi (l-l')} \, \mathrm {d}\phi
								\nonumber \\&\quad = \int _0^{2\pi }\frac{1}{4} \left[
								e^{\mathrm {i}\,\phi (l-l'+2m)}+e^{\mathrm {i}\,\phi (l-l'-2m)} +
								2e^{\mathrm {i}\,\phi (l-l')}\right] \, \mathrm {d}\phi .
								\end{aligned}$$

As with the angular integration for the first order coupling, a non-zero value is only achieved when the exponentials disappear before the integration. We therefore have conditions for non-zero second order coupling:

11.47 $$\begin{aligned}
								\begin{aligned} {}&2m = |l-l'| \\ {}&\mathrm {or} \\
								{}&l = l' . \end{aligned}
								\end{aligned}$$

Integrating with respect to $$\phi
								$$ we have:

11.48 $$\begin{aligned}
								I_{\phi } = \left\{ \begin{array}{ll} 0 &{}\quad 2m \ne
								|l-l'|, \ \ l\ne l' \\ \frac{\displaystyle \pi }{2} &{}\quad
								2m=|l-l'|, \ \ \mathrm {even}\ Z_n^m \\ - \frac{\displaystyle \pi
								}{2} &{} \quad 2m=|l-l'|, \ \ \mathrm {odd}\ Z_n^m \\ \pi
								&{}\quad l=l', \ \ m\ne 0 \\ 2\pi &{}\quad l=l', \ \
								m=0 . \\ \end{array} \right.
								\end{aligned}$$

For the radial integration we make the variable substitution $$x =
								\frac{2r^2}{w^2}$$ which gives:

11.49 $$\begin{aligned}
								\begin{aligned} k_{p,l,p',l'}^{n,m,2} = {}&-2 k^2
								\frac{1}{w^2} \frac{2 I_{\phi }}{\pi } \sqrt{\frac{p!
								p'!}{(|l|+p)!(|l'|+p')!}} \exp {(\mathrm {i}\,\varDelta o \ \psi )}
								\\ {}&\times \int _0^X x^{\frac{|l|+|l'|}{2}}
								L_p^{|l|}(x)L_{p'}^{|l'|}(x)\exp {(-x)} \left[ R_n^m\left(
								\sqrt{\frac{x}{2}} \frac{w}{R}\right) \right] ^2 \sqrt{\frac{x}{2}}w
								\ \mathrm {d}x , \end{aligned}
								\end{aligned}$$

where $$\varDelta
								o$$ is the difference in order between the incident and coupled mode and $$X=\frac{2R^2}{w^2}$$ is the limit of the exponential. As with the first order coupling we use the lower incomplete gamma function, $$\gamma
								(a,x)=\int _0^x t^{a-1}e^{-t}\mathrm {d}t$$ to get the final solution:

11.50 $$\begin{aligned}
								\begin{aligned} k_{p,l,p',l'}^{n,m,2} = {}&-\frac{I_{\phi
								}}{\pi } k^2 A^2 \sqrt{p! p'!(p+|l|)!(p'+|l'|)!} \exp {(\mathrm
								{i}\,\varDelta o \ \psi )} \\ {}&\times \sum _{i=0}^p \sum
								_{j=0}^{p'} \sum _{h=0}^{\frac{1}{2}(n-m)} \sum
								_{g=0}^{\frac{1}{2}(n-m)} \frac{(-1)^{i+j+h+g} (n-h)! (n-g)!
								X^{h+g-n}}{(p-i)!(p'-j)!(|l|+i)!(|l'|+j)!i!j!h!g!} \\
								{}&\times \frac{\gamma
								(i+j+n-h-g+\frac{1}{2}(|l|+|l'|)+1,X)}{(\frac{1}{2}(n+m)-h)!(\frac{1}{2}(n+m)-g)!(\frac{1}{2}(n-m)-h)!(\frac{1}{2}(n-m)-g)!}
								. \end{aligned} \end{aligned}$$

**Fig. 125:**
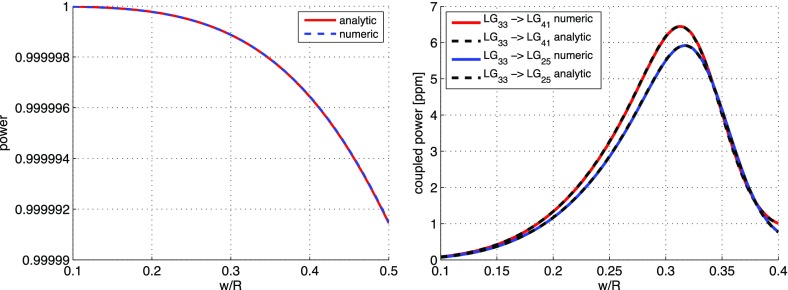
*Plots* illustrating relationship between power scattered into higher order modes by a curvature mismatched mirror and the relative beam size. *Left* Power scattered out of an incident 00 mode. *Right* Power scattered from LG$_{33}$ into 2 other order 9 modes, LG$_{41}$ and LG$_{25}$. For *both plots* both the analytical coupling approximation and numerical results are shown

Using this derivation of the second order term, combined with our previous derivation of the first order coupling, the amplitude/power coupled back into the incident mode can be calculated. In the left panel of Fig. 125 the power scattered out of an LG$_{00}$ mode incident on an mode-mismatched mirror is plotted against the relative beam size. The larger the beam size the more of the distortion the beam ‘see’ and hence the more power is scattered into higher order modes. In the right panel shows the power coupled from an LG$_{33}$ mode incident on an astigmatic mirror into 2 other order 9 modes, LG$_{41}$ and LG$_{25}$. In both cases the analytic coupling approximation and numerical results agree.

This coupling approximation has proved particularly useful as it allows for quick identification of the sources of coupled modes. For example, this approximation was used in the case of an investigation into the compatibility of the LG$_{33}$ mode with the current advanced detector mirrors (Bond et al. 2011). The main cause of worse performance, compared to the fundamental mode, is coupling into modes of the same order (in the case of LG$_{33}$ order 9). This approximation means we can quickly identify which mirror distortions will couple between LG$_{33}$ and other order 9 modes, and hence which mirror shapes need stricter requirements to produce LG$_{33}$ compatible mirrors. Table 7 summarise the shapes which couple (to first order) into each order 9 mode from LG$_{33}$. Only shapes with specific azimuthal structures cause coupling between specific modes, and here we see only even azimuthal indices (*m*) will cause problems for the LG$_{33}$ mode. Using this approximation mirror requirements were derived to produce an equivalent performance between an injected LG$_{33}$ laser mode and a fundamental mode (Bond et al. 2011).

**Table 7:** Azimuthal index (*m*) of the Zernike shapes required to cause first order coupling from an incident LG$_{33}$ mode into each of the other order 9 modes

mode (*p*, *l*)	4, 1	2, 5	4, $-$1	1, 7	3, $-$3	0, 9	2, $-$5	1, $-$7	0, $-$9
*m*	2	2	4	4	6	6	8	10	12

### Efficient coupling matrix computations with multiple distortions

Evaluating coupling coefficients numerically is a computationally expensive task if an analytic solution is not known for a particular distortion to the beam shape. Analytic solutions such as those from Bayer-Helms (1984) for mode-mismatches and misalignments (see Sect. 9.16) provide a fast way to compute the matrices for such effects. However, if a surface defect or some other distortion is also applied to a mirror this can require full numerical integration which is very slow, especially if the simulation varies the mode-mismatch or alignments. Different distortions can mathematically be separated into multiple coupling coefficient matrices, allowing a fast method to solve one which varies often, like mode-mismatch, and a slow numerical integration which often need only be performed once.

Consider two general distortions to the beam *A* and *B*, these could be tilts, apertures, surface defects, etc.

11.51 $$\begin{aligned}
								k_{MN} = \iint _{-\infty }^{\infty }{U_N(x,y,q_1) A(x,y) B(x,y)
								U^{*}_M(x,y,q_2)} \, \mathrm {d}x \, \mathrm {d}y.
								\end{aligned}$$

What we want to be able to do is separate the effects as the coupling caused by the distortion *A* might be analytically solveable and variable and whereas *B* may take along time to recompute and is constant, therefore we only want to compute it once. This is the typical scenario when considering simulating varying mode-mismatches and static surface distortions or apertures on a mirror for example.

Such a coupling coefficient computation can be represented as vectors in a Hermite–Gaussian polynomial basis—for convenience we write for shorthand $$|U_N(x,y,q_1)\rangle \rightarrow |N,q_1\rangle
								$$.

11.52 $$\begin{aligned}
								k_{MN}= & {} \langle N,q_1| A(x,y) B(x,y) |M,q_2\rangle ,
								\end{aligned}$$

11.53 $$\begin{aligned}
								| N,q \rangle= & {} \begin{bmatrix} 0 \\ \vdots \\
								U^*_N(x,y,q) \\ \vdots \\ 0 \end{bmatrix}, \,\, \langle N,q| =
								\begin{bmatrix}
								0&\ldots&U_N(x,y,q)&\ldots&0
								\end{bmatrix}, \qquad \quad \end{aligned}$$

11.54 $$\begin{aligned}
								\langle N,q_1 | M,q_2 \rangle= & {} \iint _{-\infty
								}^{\infty } U_N(x,y,q_1)U^*_M(x,y,q_2) \, \mathrm {d}x \, \mathrm
								{d}y \qquad \end{aligned}$$

11.55 $$\begin{aligned}
								\hat{I}_{MN}= & {} \sum ^\infty _{M}\sum ^\infty _{N} | N,q
								\rangle \langle M,q | \nonumber \\= & {} \sum ^\infty
								_{N,M=N} \left( \iint _{-\infty }^{\infty } U_N(x,y,q)U^*_N(x,y,q)
								\, \mathrm {d}x \, \mathrm {d}y \right) = 1 .\qquad \quad
								\end{aligned}$$

We then define two new vectors and the inner product between them

11.56 $$\begin{aligned}
								k_{MN}= & {} \langle v_N | v_{M} \rangle
								\end{aligned}$$

11.57 $$\begin{aligned}
								|v_M,q_2 \rangle \!= & {} \! \begin{bmatrix} 0 \\ \vdots \\
								U^*_M(x,y,q_2)B(x,y) \\ \vdots \\ 0 \end{bmatrix}, \,\, \langle
								v_N,q_1| \!=\! \begin{bmatrix} 0&\ldots&A(x,y)
								U^*_N(x,y,q_1)&\ldots&0 \end{bmatrix},\nonumber \\
								\end{aligned}$$

We then insert the identity matrix, formed by a complete orthonormal basis set of modes,

11.58 $$\begin{aligned}
								k_{MN}= & {} \langle v_N, q_1 | \hat{I} | v_{M},q_2 \rangle
								\end{aligned}$$

11.59 $$\begin{aligned}=
								& {} \langle v_N q_1 | \left( \sum ^\infty _{L} | L,q_l
								\rangle \langle L,q_l | \right) | v_{M},q_2 \rangle
								\end{aligned}$$

11.60 $$\begin{aligned}=
								& {} \sum ^\infty _{L} \langle v_N, q_1 | L,q_l \rangle
								\langle L,q_l | v_{M}, q_2 \rangle
								\end{aligned}$$

11.61 $$\begin{aligned}=
								& {} \sum ^\infty _{L} \langle N, q_1 | A(x,y) | L,q_l
								\rangle \langle L,q_l | B(x,y) | M,q_2 \rangle
								\end{aligned}$$

From this we can see that we now have two separate inner products and a sum over the infinite number of Hermite basis functions. In practice this is limited to a certain number of modes of interest. The last line is identical to a matrix multiplication where each inner product represents the element of a matrix,

11.62 $$\begin{aligned}
								k_{MN}= & {} \sum _{L} \langle N | A(x,y) | L \rangle
								\langle L | B(x,y) | M \rangle
								\end{aligned}$$

11.63 $$\begin{aligned}=
								& {} \sum _{L} \hat{A}_{NL} \hat{B}_{LM} =
								(\hat{A}\hat{B})_{MN} , \end{aligned}$$

where each matrix is now coupling coefficients for each distortion.

The expansion beam parameter $$q_l$$ can in theory be set to any value; however the computational requirements can be reduced if it is chosen sensibly. Remember that a mode-mismatch is present if $$q_1 \ne
								q_2$$, so if $$q_l$$ is chosen to be either $$q_1$$ or $$q_2$$ the mode-mismatch is present in only one of the matrices. This is beneficial as coupling coefficient matrices are Hermitian if there is no mode-mismatch. Thus only one half of the matrix elements need to be computed—when solving via numerical integration this can save a great deal of time. There is also the issue of matrix commutation, *A*(*x*, *y*) and *B*(*x*, *y*) are interchangeable in the derivation thus it appears $$[\hat{A},
								\hat{B}] = 0$$, which is a surprising result seeing as the functions can be any arbitrary values. In practice it is found that commutation errors are only present if the functions are not described using enough higher-order-modes. If significant amount of information is lost in modes that are not considered, commutation errors are likely to occur.

### Clipping by finite apertures

Another spatial effect present in real interferometers is the finite size of the optics. Often in simulations with Gaussian modes or plane waves there is some intrinsic assumption that the optics are infinite. In reality the size of the optics is carefully chosen, optimising between large optics to contain the power of the incident beams and smaller optics to reduce the impact of thermal noise.

A finite aperture in the path of a laser beam will produce higher-order modes. However, in the case of well designed interferometers, such as gravitational wave detectors, the effect can often be modelled as just a loss of power in the fundamental mode, so called *clipping loss*. In such interferometers the size of the optics are chosen such that they are large enough, compared to the beam size, that very little power is lost over the edges. For an LG mode this loss is given by:

11.64 $$\begin{aligned}
								l_{clip} = 1 - \int _{A}|u_{p,l}|^2 \, \mathrm {d}A .
								\end{aligned}$$

The integral represents the normalised power reflected by a mirror with a finite aperture. For a large mirror the loss is effectively 0. The loss for LG modes is derived as:

11.65 $$\begin{aligned}
								\begin{aligned} l_{clip} ={}&1-p!(p+|l|)! \sum _{m=0}^p \sum
								_{n=0}^p \frac{(-1)^{n+m}}{(p-n)!(p-m)!} \\ {}&\times
								\frac{1}{(|l|+n)!(|l|+m)!n!m!} \gamma (|l|+n+m+1,X) , \end{aligned}
								\end{aligned}$$

where $$X=\frac{2R^2}{w^2}$$ and $$\gamma
								$$ is the lower incomplete gamma function. Generally a clipping loss of the order 1 ppm ($$10^{-6}$$) is desirable.

Generic analytic coupling coefficients describing clipping at a circular aperture is available in Vinet and the Virgo Collaboration (2001).

### Cavity modes of many shapes

A realistic cavity has a resonate mode which deviates slightly from that of a pure Gaussian eigenmode: i.e., it is a perfect resonator for a slightly distorted Gaussian beam, as described by the distorted cavity mirrors. As an example we consider the case of an astigmatic cavity and the Laguerre–Gauss modes. An astigmatic cavity has differing curvatures along the *x* and *y* axes. The Laguerre–Gauss modes, with their cylindrically symmetric properties, are not eigenmodes of such a system. In cases with a large astigmatism *frequency splitting* can be observed, where an injected Laguerre–Gauss mode is broken down into the eigenmodes of the cavity. For an astigmatic cavity these eigenmodes are astigmatic Hermite–Gauss modes, which can be separated in *x* and *y*

11.66 $$\begin{aligned}
								u_{n,m}(x,y,z) = u_n(x,z,R_{C,x})u_m(y,z,R_{C,y}) ,
								\end{aligned}$$

with different curvature associated with the *x* and *y*. The frequency splitting phenomenon has its cause in the difference in Gouy phase accumulated in *x* and *y* for the different modes. The total Gouy phase is:

11.67 $$\begin{aligned}
								\varphi _{n,m} = (n+\tfrac{1}{2}) \psi _x(z) + (m+\tfrac{1}{2})\psi
								_y(z) . \end{aligned}$$

**Fig. 126:**
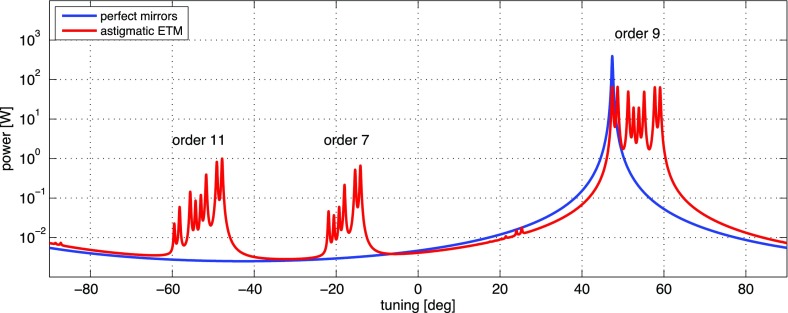
Scan of a cavity injected with an LG$_{33}$ mode. The results for a cavity with perfectly spherical mirrors show a single resonance for the order 9 mode. For an astigmatic cavity the high order resonances are split into the resonances of the astigmatic Hermite–Gauss modes, the eigenmodes of the cavity. Coupling into orders 7 and 11 caused by the astigmatism also display this frequency splitting

**Fig. 127:**
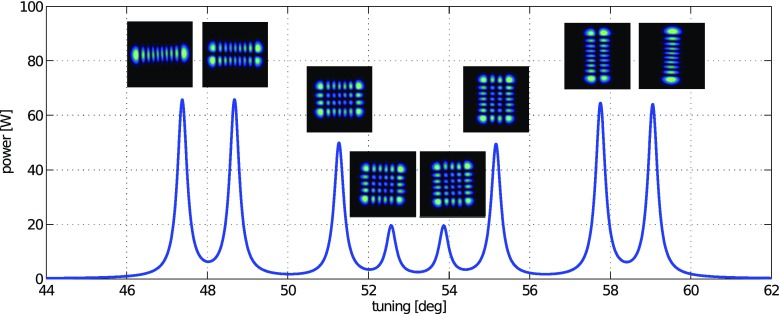
Scan over the order 9 peak in an astigmatic cavity injected with an LG$_{33}$ mode. The peak is split into the resonances of the order 9 Hermite–Gauss modes, the eigenmodes of the cavity

**Fig. 130:**
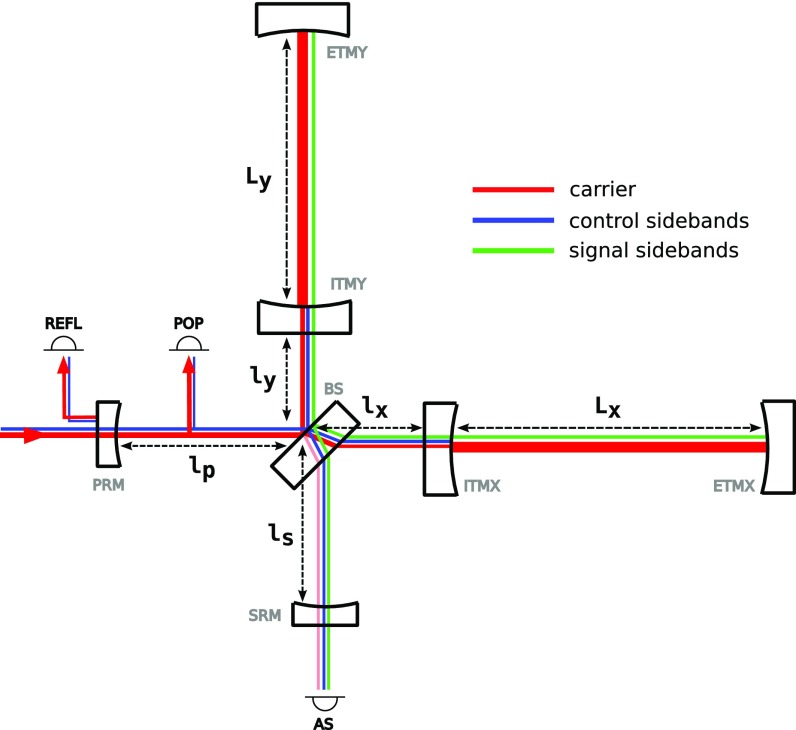
Simplified layout of the dual recycled Fabry–Perot–Michelson of Advanced LIGO, illustrating the fields inside the interferometer. All main mirrors are shown: power and signal recycling mirrors (PRM and SRM); input test masses (ITMX and ITMY); end test masses (ETMX and ETMY); and the central beam splitter. Also shown are the locations of various photodiodes: REFL (reflected port), POP (power recycling pick off port) and AS (anti-symmetric port). The paths of the different light fields in the interferometer are shown. The carrier resonates inside the arm cavities and power recycling cavity, with the Fabry–Perot–Michelson tuned close to the carrier dark fringe. A small amount of carrier light leaks into the anti-symmetric port due to a small DC offset. The RF sidebands used for control resonate in the power recycling cavity but are reflected from the arms. The Michelson is not at the dark fringe for the control sidebands and they appear in the signal recycling cavity and at the Michelson output. The signal sidebands generated by a gravitational wave originate in the arm cavities and then exit the Michelson through the anti-symmetric port

This results in slightly different resonance frequencies for different Hermite–Gauss modes of the same order. Consider for instance the Laguerre–Gauss mode LG$_{33}$, an order 9 mode. As described in Sect. 9.11, this mode can be described as a sum of order 9 Hermite–Gauss modes. In the case where this mode is injected into an astigmatic cavity, instead of a single clean resonance peak at the order 9 resonance, we see a spread of resonances corresponding to the different Hermite–Gauss modes. This is illustrated in Figs. 126 and 127 where the individual higher-order resonances are split across the resonances of the astigmatic Hermite–Gauss modes: the eigenmodes of such a cavity.

This example is illustrative of a more general effect: the fact that the resonant modes of a distorted cavity will differ from a perfect Gaussian mode. We also note that the finite size of the cavity mirrors makes the situation more complex, for example it affects the orthogonality of the cavity eigenmodes (Siegman 1979).

## Figures and Tables

**Fig. 128:**
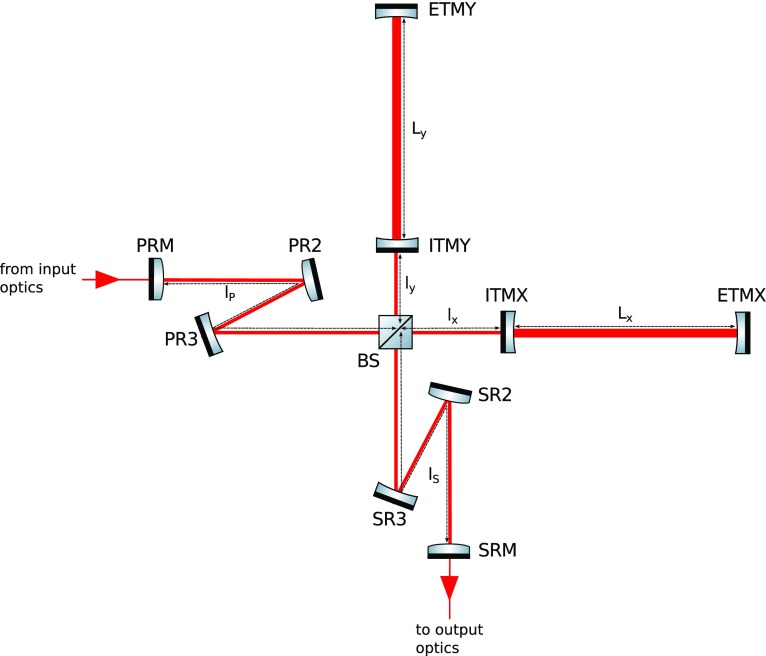
Optical layout of the central interferometer of Advanced LIGO, a dual recycled Michelson interferometer with Fabry–Perot arm cavities. The input beam comes from an input mode cleaner, which filters the beam of unwanted spatial and frequency components. The output beam is sent to an output mode cleaner before it reaches the final detection photodiode. The arm cavities are characterised by the input and end test masses (ITMs and ETMs). Each recycling cavity consists of 3 mirrors, the power/signal recycling mirror (PRM/SRM) and two folding mirrors (PR2/SR2 and PR3/SR3). The distances between the key optics are labelled: the distance between the recycling mirrors and the beam splitter ($l_P$ and $l_S$), the short Michelson arms ($l_x$ and $l_y$) and the arm cavity lengths ($L_x$ and $L_y$)

**Fig. 129:**
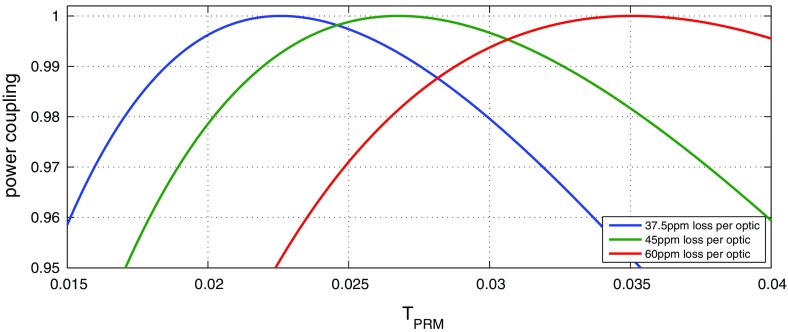
Power coupled into an Advanced LIGO interferometer versus power recycling mirror transmission. The power coupled is simulated for 3 different values of scattering loss per optic, 37.5 ppm (nominal value), 45 and 60 ppm

## Footnotes

1 This equation only considers shot-noise, which is one aspect of quantum noise, see Sect. 6 for a more detailed description of quantum noise.

2 In many implementations of numerical matrix solvers the input vector is also called the *right-hand side* vector.

3 Note that in other publications the tuning or equivalent microscopic displacements are sometimes defined via an optical path-length difference. In that case, a tuning of $$2\pi
								$$ is used to refer to the change of the optical path length of one wavelength, which, for example, if the reflection at a mirror is described, corresponds to a change of the mirror’s position of $$\lambda
								_0/2$$.

4 The signal sidebands are sometimes also called *audio sidebands* because of their frequency range.

5 The term *effective* refers to that amount of incident light, which is converted into photo-electrons that are then usefully extracted from the junction (i.e., do not recombine within the device). This fraction is usually referred to as *quantum efficiency* $$\eta
								$$ of the photodiode.

6 Please note that in the presence of losses the coupling is defined with respect to the transmission and losses. In particular, the impedance-matched case is defined as $$T_1=T_2\times
								\mathrm {Loss}$$, so that the input power transmission exactly matches the light power lost in one round-trip.

7 The term ‘main signals’ refers to the optical signal providing the readout of the interferometric measurement, for example, of a position or length change. In addition, other output signals exist: for example, the light power reflected back into the West port can be recorded for monitoring the interferometer status.

8 Derivations of the accumulated phase can be found in many works, a simple example is presented in Bond (2014).

9 It is proportional to a factor $$\chi
								$$, the photodiodes quantum efficiency, which states how many Amps per Watt of incident power is output by the photodiode. We will assume here the efficiency is perfect, $$\chi
								=1$$, for simplicity.

10 The tuned case is slightly special, because the integrated area is half compared to the others, because the plot shows only the positive half of the total linewidth seen by signal sidebands.

11 The typical light wavelength is $${\sim }
								10^{-6}\,$$m while all ground-based interferometers built or planned have target displacement noise spectral densities below $${\sim }
								10^{-19}\,{\mathrm m}/\sqrt{\mathrm Hz}$$ in a frequency band of order 100 Hz wide.

12 To minimise thermal noise in suspensions, low-loss materials and techniques are employed to avoid dissipation. The resonant modes of these suspensions are seen in the frequency domain as narrow spectral features, or lines. The violin modes are transverse oscillations of the stretched suspension fibres that support the mirrors, which vibrate much like a violin string. The frequencies of these modes typically lie between 300 and 800 Hz, and they are often conspicuous in the spectra of signals from gravitational-wave detectors.

13 Also known as the *far-field angle* or the *divergence* of the beam.

14 Please note that this formula from Siegman (1986) is very compact. Since the parameter *q* is a complex number, the expression contains at least two complex square roots. The complex square root requires a different algebra than the standard square root for real numbers. Especially the third and fourth factors can *not* be simplified in any obvious way: $$\left(
								\frac{q_0}{q(z)}\right) ^{1/2} \left(
								\frac{q_0q^*(z)}{q_0^*q(z)}\right) ^{n/2} \ne \left(
								\frac{q_0^{n+1}{q^*}^n(z)}{q^{n+1}(z){q_0^*}^n}\right)
								^{1/2}$$!

15 Siegman (1986) states that the indices must obey the following relations: $$0\le |l|\le
								p$$. However, that is not the case.

16 Here we refer to the input power as the power injected into the central Michelson interferometer, after the input mode cleaner and other input optics.

17 This shows results which are slightly different than those reported in Arain and Mueller (2008) because we have used the final design values for this plot, not the preliminary values used in Arain and Mueller (2008).

18 34.5 km is the focal length when modelled as an individual lens in a vacuum, the approach in this document. Sometimes quoted is 50 km corresponding to the lens when modelled inside the fused silica substrate of the ITM.

19 Many more such documents exist for Advanced LIGO and other gravitational wave detectors. This selection is based on our familiarity with the described work.

## Appendix A: The interferometer simulation Finesse

Throughout this document we have provided a number examples using the interferometer simulation Finesse (Freise 2015; Freise et al. 2004). We encourage the reader to obtain Finesse and to learn its basic usage by running the included example files (and by making use of its extensive manual). The program has been designed to allow the analysis of arbitrary, user-defined optical setups. In addition, it is easy to install and use. Therefore, Finesse is well suited to study basic optical properties. The Finesse input files provided in this article are in most cases very simple and illustrate single concepts in interferometry. We believe that even a Finesse novice should be able to use them as starting points to play and explore freely, for example, by changing parameters, or by adding further optical components. This type of ‘numerical experimentation’ can provide insights similar to real experiments, supplementing the understanding through a mathematical analysis with experience and intuition.

**Free software**

Finesse is a free software package developed and maintained by some of us (AF and DB): we provide free downloads of binaries for Linux, Windows and Macintosh computers online at: http://www.gwoptics.org/finesse/. The code is available under the GPLv2 license and does not require any commercial software (such as *Matlab*) to compile and run.

**Development**

Finesse is a numerical simulation written in the C language; it is actively developed to fix bugs as they are found and to add new features required to simulate new interferometric systems. The recent updates allow modelling of suspended optics that are effected by radiation pressure of the laser light as well as quantum effects such as squeezing and vacuum noise. We are further developing Pykat (Brown and Freise 2015), a Python based toolbox to extend the use of Finesse, in particular for automating tasks and data post-processing.

**Gravitational-wave research**

The Finesse development started in 1997 to support the design and commissioning of the gravitational-wave detector GEO 600 and Finesse has remained the standard simulation software of that project. Since then Finesse has been developed continuously to meet the new challenges of the interferometric gravitational-wave detectors worldwide such as LIGO, Virgo and KAGRA.^20^ Finesse provides a fast and versatile tool that is optimised for the type of low-noise, steady state laser interferometry used by gravitational-wave detectors.

## Appendix B: Advanced LIGO optical layout

In this review, the specific example of Advanced LIGO has been used to illustrate the operation of an advanced gravitational wave detector. In this section, the layout and parameters of Advanced LIGO are presented, with a brief description of the motivation for some of the key parameters.

**Advanced LIGO configuration**

Figure 128 shows the configuration of the central interferometer of Advanced LIGO, a dual recycled Michelson interferometer with Fabry–Perot arm cavities. The interferometer is characterised by the parameters and performance of the different subsystems of the interferometer: the arm cavities, power recycling cavity, signal recycling cavity and the Michelson.

Table 8 summarises the design optical parameters of the key components of Advanced LIGO: the input and end test masses (ITM and ETM) and the power and signal recycling mirrors (PRM and SRM). A 50:50 beam splitter is used and the design assumes a loss per optic of 37.5 ppm. The additional mirrors which make up the recycling cavities are highly reflective with a transmittance on the order of 1 ppm.

**Table 8:** Summary of the key optical parameters of the Advanced LIGO design

Parameter	Value
ITM *T*	1.4 %
ETM *T*	5 ppm
PRM *T*	3.0 %
SRM *T*	20 %
BS *T*	50 %
Arm cavity finesse	450
Loss per optic	37.5 ppm

The transmittance, *T*, of the main mirrors is quoted (in % or parts-per-million, ppm), as well as the expected loss per optic and the finesse of the arm cavities. The reflectivity of the mirrors is simple calculated as $$R=1-T-L$$, where *T* is the transmittance and *L* is the loss for each mirror (Arain and Mueller 2009). Note that these parameters represent the design at a certain period in time and not always the exact parameters of the currently operating detectors. For example at the time of writing the transmittance of the installed SRM mirror is $${\approx }
												35$$ %

As well as the optical properties the response of an interferometer is also characterised by its geometric parameters, such as the lengths of the arm cavities. Table 9 summarises the key lengths of the Advanced LIGO design: the lengths of the arm and recycling cavities. Also included is the Schnupp asymmetry length, a small difference in the short Michelson length of the *x* and *y* arms.

**Table 9:** Key lengths of the Advanced LIGO design

Length	Symbol	Value (m)
Power recycling cavity	$L_\mathrm{{PRC}} = l_P + \frac{l_x+l_y}{2}$	57.656
Signal recycling cavity	$L_\mathrm{{SRC}} = l_S + \frac{l_x+l_y}{2}$	56.008
Arm cavity	$L_x=L_y$	3994.5
Schnupp asymmetry	$l_\mathrm{{Sch.}}=l_x-l_y$	0.050

The length of the recycling cavities ($L_\mathrm{{PRC}}$/$L_\mathrm{{SRC}}$) is the average distance between the recycling mirror (PRM or SRM) and the ITMs (using the average of the short Michelson arms for the distance from the BS and ITM, $\frac{l_x+l_y}{2}$). The arm cavity length is the distance between the highly reflective surfaces of the ITM and ETM ($L_x$/$L_y$). Finally, the Schnupp asymmetry, $l_\mathrm{{Sch.}}$, is the difference between the short Michelson arms $l_x-l_y$ (Arain and Mueller 2009). Figure 128 shows a diagram of the Advanced LIGO layout which illustrates these lengths

**Choice of parameters**

The parameters of Advanced LIGO, as used in models throughout this review, are summarised above. In this section we provide a brief description of the motivation behind the choice of specific parameters. The majority of the parameters and arguments presented in this section are taken from Abbott et al. (2010).

*Arm cavity finesse*

The first consideration is the arm cavities. The length of the arms is set to ${\sim } 4$ km, from the existing LIGO infrastructure. This gives a free spectral range of 37.5 kHz. The choice of the reflectivity of the mirrors, or the finesse of the arm cavities, will impact other aspects of the design, so is chosen with care. The decision to have ${\sim }800$ kW circulating in the arms (during high power operation) was made early on, in order to reduce shot noise in the interferometer, the limiting noise source at most frequencies for initial LIGO. A combination of parameters determine this intra-cavity power: input laser power, power recycling gain and arm cavity finesse; the finesse was designed to be 450 (Fritschel et al. 2007).

*PRM transmission*

The transmission of the power recycling mirror (PRM) is carefully chosen to be close to impedance matched with the Fabry–Perot–Michelson, ensuring maximum power coupled into the interferometer and close to zero reflected. This requires some knowledge or estimate of the loss per mirror, which the design states should not be greater than 37.5 ppm per optic. In Fig. 129 the power coupled into the interferometer for different PRM transmissions is modelled, considering different mirror losses (Abbott et al. 2010). A PRM transmission of 3 % was chosen, giving slight over-coupling for 37.5 ppm losses but providing robustness against greater losses and the potential to detect error signals in reflection. It should be noted that most of the power is ‘transmitted’ through the losses in the arms, rather than through the end mirrors.

*Recycling cavity lengths*

The choice of lengths for the power and signal recycling cavities are closely linked to the control scheme of Advanced LIGO. This involves injecting 2 pairs of control sidebands into the interferometer, at two different frequencies. The 2 sidebands have (design) frequencies of $$f_1 = 9099471$$ Hz and $$f_2 = 5\times f_1=
									45497355$$ Hz, which are chosen to be anti-resonant in the arm cavities when the carrier is resonant (i.e., they are reflected by the arm cavities) and to avoid coinciding with any higher order mode resonances in the arms.

In the case of Advanced LIGO both sidebands must be resonant in the power recycling cavity when it is locked to the carrier. This puts a strict condition on the PRC length:

13.1 $$\begin{aligned} L_\mathrm{PRC} = \left(
									N+\frac{1}{2}\right) \frac{c}{2f_1} .
									\end{aligned}$$

The factor of $\frac{1}{2}$ is included as the sidebands are $180^{\circ }$ out of phase with the carrier, as the carrier enters the arm cavities whilst the sidebands do not. A power recycling cavity length of 57.6557 m ($N=3$) was chosen to be compatible with the optomechanical layout for a stable recycling cavity (Abbott et al. 2010). The signal recycling cavity length was chosen to be resonant for $f_2$ but not for $f_1$, i.e., (Abbott et al. 2010)

13.2 $$\begin{aligned} L_\mathrm{SRC} =
									M\frac{c}{2f_2} \ne Q\frac{c}{2f_1} ,
									\end{aligned}$$

where *M* and *Q* are integers. An SRC length of 56.0084 m was chosen (Abbott et al. 2010).

For control of Advanced LIGO one of the sidebands should exit the Michelson into the signal recycling cavity and eventually the output port. However, in a Michelson where the short arms between the beam splitter and ITMs ($$l_x$$/$$l_y$$) are the same length the dark fringe for the carrier will also be the dark fringe for the sidebands. In order for one of the sidebands ($$f_2$$) to leak into the signal recycling cavity we need some asymmetry between the two arms, the so-called *Schnupp asymmetry*, $$l_{Sch} = l_x-l_y\ne
									0$$ (Hild et al. 2009). In the case of Advanced LIGO a Schnupp asymmetry of 5 cm was a compromise between maximising the coupling of $$f_2$$ into the signal recycling cavity for two different cases: broadband signal recycling and slightly detuned signal recycling (optimised for neutron star–neutron star in-spiral (NS–NS) signals).

*Mode-matching*

The curvatures of the highly reflective mirror surfaces and distances between individual mirrors determine the size and shape of the resonating beams. The individual cavities in the detector need to be well mode matched to maximise the power build up and avoid high contrast defects.

The eigenmode of the arm cavities is selected to produce large beams at the ITM (5.3 cm) and ETM (6.2 cm) to reduce thermal noise, with slightly smaller beams at the ITM due to lower thermal noise (fewer coating layers) and to prevent scattering into the recycling cavities. The curvatures are also carefully selected for a specific Gouy phase to avoid higher order modes easily ringing up in the arms: $R_C=1934$ m (ITM) and $R_C=2245$ m (ETM).

In initial LIGO the power recycling cavity was marginally unstable, enhancing the power in higher order modes in the control sidebands and causing problems for control (Gretarsson et al. 2007). To avoid this in Advanced LIGO the interferometer was designed with stable recycling cavities: folded cavities which do not share the arm cavity eigenmode (Arain and Mueller 2008), see Sect. 10.7. These cavities consist of 3 mirrors, the primary recycling mirrors (PRM/SRM) and two additional mirrors to shape and direct the beam (PR2/SR2 and PR3/SR3). The curvatures and positions of the mirrors are chosen to ensure good mode matching into the arm cavities and to achieve a good spacing between higher order resonances (see Sect. 10.7).

The mode matching of the beams between the recycling cavities and arms is complicated by thermal effects, specifically thermal lensing and the change in mirror curvatures. Although this will be corrected by thermal compensation systems (Willems 2009) it was decided to match the recycling cavities to the arms in the presence of 50 km lenses in the ITMs, as expected for an input power of ${\sim }12.5$ W with coating absorptions of 0.5 ppm (Arain and Mueller 2009). This will potentially mitigate the use of TCS at low power. In the end the cavities were designed for a 50 km lens inside the substrate (effective 34.5 km lens) corresponding to 18 W input power.

**Degrees of freedom**

In Fig. 130 a simplified diagram of the Advanced LIGO dual recycled configuration is shown, with the lengths which require control labelled. The microscopic degrees of freedom associated with these lengths are:

- **CARM** Common-mode arm motion, CARM $$=L_x+L_y$$. This tunes the average length of the arm cavities and is used to keep the arms on resonance.
- **PRCL** Power recycling cavity length, PRCL $$=l_p
  												+\frac{l_x+l_y}{2}$$. The power recycling cavity is operated on resonance to maximise the power coupled into the central interferometer.
- **MICH** Michelson arm length, MICH $$=l_x-l_y$$. MICH controls the short arms of the Michelson (between the ITMs and beam splitter) and determines the fringe at the output port. Generally the Michelson is operated on the dark fringe.
- **DARM** Differential arm motion, DARM $$=L_x-L_y$$. This controls the difference in length of the two arm cavities and is used to get the best interference between the two arms at the dark port.
- **SRCL** Signal recycling cavity length, SRCL $$=l_s+\frac{l_x+l_y}{2}$$. Used to control the operation of the signal recycling cavity. The operating point of SRCL depends on the mode of operation of the interferometer. It can be tuned for a particular frequency of gravitational wave or for broadband operation.
