# Publisher Correction: Interferometer techniques for gravitational-wave detection

**DOI:** 10.1007/s41114-017-0005-0

**Published:** 2017-08-15

**Authors:** Charlotte Bond, Daniel Brown, Andreas Freise, Kenneth A. Strain

**Affiliations:** 1 School of Physics and Astronomy, University of Birmingham, Birmingham, B15 2TT UK; 2 School of Physics and Astronomy, University of Glasgow, Glasgow, G12 8QQ UK

## Publisher’s Correction to: Living Rev Relativ (2016) 19:3 DOI 10.1007/s41114-016-0002-8

Due to a technical error during the production process the article was originally published with incorrect bibliographical information. The article has been updated with the following correct bibliographical information:

Living Rev Relativ (2016) 19:3

Received: 4 December 2015

Accepted: 21 July 2016

Published online: 17 February 2017

The earlier version incorrectly identifying the article as Living Rev Relativ (2016) 19:1 should be disregarded.

